# Crystal Engineering of Reticular Materials for Gas‐ and Liquid‐Phase Hydrocarbon Separation

**DOI:** 10.1002/adma.202512551

**Published:** 2026-02-11

**Authors:** Xia Li, Soumya Mukherjee, Michael J. Zaworotko

**Affiliations:** ^1^ College of Chemistry Nankai University Tianjin P. R. China; ^2^ Department of Chemical Sciences Bernal Institute and Research Ireland Centre for Pharmaceuticals (SSPC) University of Limerick Limerick Ireland

**Keywords:** adsorptive separation, binding sites, coordination networks, crystal engineering, hydrocarbons, reticular materials

## Abstract

Crystal engineering focuses upon the design, properties, and applications of crystals, whereas reticular chemistry involves linking molecular building blocks to create network structures. The intersection of these areas is evident in the number of systematic studies of structure/function relationships concerning porous coordination networks (PCNs) and covalent organic frameworks (COFs). PCNs and COFs are inherently modular in nature and therefore amenable to systematic fine‐tuning of both pore size and chemistry in a manner that is infeasible for other classes of porous solid. This review highlights how this exquisite control over pore size and chemistry has enabled the development of a new generation of physisorbents that are effective in the context of industrially relevant hydrocarbon (HC) separations. The motivation behind such reticular sorbents is the need to replace today's energy‐intensive HC separation methods with more sustainable alternatives. Physisorbents are attractive in this context as they can offer the high selectivity needed for trace removal of impurities along with relatively low energy of recycling. This review details how crystal engineering strategies offer precise control of pore size and chemistry to enable HC selectivity to reach hitherto unprecedented levels. Nevertheless, despite these property advances, challenges remain to be addressed before commercial adoption becomes feasible.

## Graphical abstract

Crystal engineering enables systematic study of structure/function relationships as exemplified by pore engineering of reticular sorbents, including porous coordination networks and covalent organic frameworks. This review assesses such studies applied across the full scope of industrially relevant hydrocarbon separations to provide insight into how reticular sorbents offer low energy alternatives to conventional separation processes like cryogenic distillation and solvent extraction.

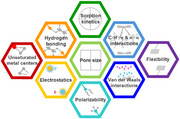

**Abbreviations**
AUM: azolate ultramicroporous material
BET: Brunauer–Emmett–Teller
CNs: coordination networks
COFs: covalent organic frameworks
CSD: Cambridge Structural Database
DCB: dynamic column breakthrough
DFT: dispersion‐corrected density functional theory
ELM: elastic layer‐structured MOF
EU: European Union
FT‐IR: fourier transform‐infrared spectroscopy
GC: gas chromatography
HCs: hydrocarbons
HUM: hybrid ultramicroporous material
IAST: ideal adsorbed solution theory
LP: large pore
MAF: metal azolate framework
MBB: molecular building block
MCM: mobil composition of matter
MD: molecular dynamics
MIL: Matériaux de l′Institut Lavoisier
MIP: Materials of the Institute of Porous Materials of Paris
MOFs: metal–organic frameworks
MOMs: metal–organic materials
MOP: metal–organic polyhedra
MTO: methanol‐to‐olefins
MUF: Massey University Framework
NG: natural gas
NP: narrow pore
PCCs: porous coordination cages
PCNs: porous coordination networks
PCP: porous coordination polymer
PET: polyethylene terephthalate
PIET: photoinduced electron transfer
*Q*_st_
: isosteric enthalpy of adsorption
RBBs: rod building blocks
RE: rare‐earth
RH: relative humidity
RON: research octane number
SBB: supermolecular building block
SCXRD: single‐crystal X‐ray diffraction
SIFSIX: hexafluorosilicate
SMB: simulated moving bed
SSST: synergistic sorbent separation technology
TGA: thermogravimetric analysis
TIFSIX: hexafluorotitanate
UMCs: unsaturated metal centeres
VOC: volatile organic compounds
VT‐PXRD: variable temperature‐powder X‐ray diffraction
ZIFs: zeolitic imidazolate frameworks

## 1 Introduction

The chemical industry, with an annual turnover of approximately $6.2 trillion in 2024, excluding pharmaceutical chemicals (≈$1.6 trillion in 2024), accounts for around 5.4% of global GDP (≈ $113.8 trillion) [1, 2]. It is also one of the most energy‐intensive industries, with around 40% of its energy consumption dedicated to separation and purification processes [3]. Overall, the energy footprint of chemical separations and purifications has been estimated to consume nearly 15% of global energy consumption [4]. A key driver of this high energy footprint is that the current state‐of‐the‐art relies upon utilization of energy‐intensive purification methods like cryogenic separation, fractional distillation, catalytic cracking, solvent extraction, and combustion [5, 6]. With the demand for chemical commodities expected to triple by 2050 [7], this energy footprint must be addressed. Energy‐efficient approaches to commodity separation and purification are therefore urgently needed, including for hydrocarbons (HCs), the highest volume products of the chemical industry.

Efforts towards decarbonization and energy efficiency are gaining momentum, with European Union (EU) initiatives to reduce greenhouse gas emissions and improve energy efficiency being part of broader climate goals [8]. Further, innovations in chemical processing, such as increased reliance on emission reduction methods like carbon capture, process electrification, and improved recycling, are being developed to help the sector meet its ambitious sustainability targets that are aligned with the EU's zero pollution ambition [9, 10]. For example, let us consider HC production, in particular ethylene (C_2_H_4_) purification, a crucial step in producing polyethylene, the World's most widely used plastic. C_2_H_4_ serves as a key feedstock for not only plastics but also detergents and coatings. Global production is already nearing 250 million tons per year, with projections suggesting that capacity will double by 2050 [11, 12]. However, producing C_2_H_4_ is energy‐intensive, particularly during the downstream purification stages where impurities like acetylene (C_2_H_2_), CO_2_, and ethane (C_2_H_6_) must be removed. C_2_H_4_’s environmental impact is significant, contributing nearly 13% of the petrochemical industry's total CO_2_ emissions. The primary culprit is the steam cracking process, which releases around 1.2 tons of CO_2_ for every ton of C_2_H_4_ produced [12, 13]. The industry is under pressure to reconcile the expected growth in HC production with global net‐zero carbon goals. Advanced technologies and machine learning are now being employed to identify more energy‐efficient separation processes, but large‐scale adoption is essential to achieve energy and emissions reduction targets. The widespread implementation of low‐cost, energy‐efficient separation methods will be crucial for the sector's sustainability goals. In the current “age of gas”—where gases and vapors have largely replaced liquids as fuels and feedstocks in the chemical industry—the strategic adoption of alternative separation technologies is vital [3]. Replacing energy‐intensive methods like fractionation, cryogenic separation, and solvent extraction with more energy‐efficient solutions, such as physisorption and its low‐energy recycling footprint, is key to this transition [3, 6].

Several classes of solid adsorbents, such as zeolites, activated carbon, and hierarchical inorganic solids, including mesoporous silica materials like Santa Barbara Amorphous‐15 (**SBA‐15**) and Mobil Composition of Matter No. 41 (**MCM‐41**), have been investigated for HC separation. However, the performance of such physisorbents has been suboptimal due to the difficulty in precisely fine‐tuning pore structure (size, shape) and environment (pore chemistry) at the molecular level. HC separations are particularly challenging for these conventional physisorbents, as they tend to suffer from weak binding affinities, especially when dealing with HCs that exhibit similar physicochemical properties.

In this context, the potential utility of advanced physisorbents in HC production from chemical feedstocks is evident (Table 1). Researchers in crystal engineering, materials science, and process engineering are actively addressing the development of innovative, energy‐efficient HC separation processes, and developing the materials that will enable these innovative processes. These efforts are increasingly driven by physisorbents that are designed from first principles and fine‐tuned by exploiting their modular compositions. This article delves into the key advances behind this new generation of crystalline reticular physisorbents, highlighting the significant progress that has been made in performance metrics relevant to HC separation and purification processes and the underlying reasons for these enhancements in performance. Amorphous porous materials, inorganic sorbents, and membranes are excluded from the scope of this review.

**TABLE 1:** A comparative analysis of conventional industrial separation processes versus reticular sorbents‐based physisorption.

Separation task	Conventional industrial route	Typical energy consumption and key constraints	Reticular sorbents‐based physisorption	Potential impact (displace/complement) and advantages
C_2_H_4_/C_2_H_6_	Cryogenic distillation	Energy: Very High (∼200–400 MJ/ton **C_2_H_4_**) Constraints: High capital cost, operates at cryogenic temperatures (−25°C to −50°C) and high pressure (∼20–30 bar), complex and energy‐intensive due to similar boiling points.	Adsorptive Separation: Equilibrium‐based: Using PCNs (e.g., **Mg‐MOF‐74**, **Fe‐MOF‐74**) with higher affinity for **C_2_H_4_**. Kinetics‐based: Using PCNs/COFs with precise pore sizes to selectively diffuse **C_2_H_4_**.	Complement/Displace in niche cases. Advantages: Can operate at near‐ambient temperatures, potentially significantly lowering energy use. Suitable for small‐scale and/or purifier units.
C_3_H_6_/C_3_H_8_ (Propylene/Propane)	Cryogenic distillation	Energy: Extremely High (∼300–500 MJ/ton **C_3_H_6_**) Constraints: Even more demanding than C2 separation due to closer relative volatility. Requires very tall distillation columns (over 200 trays).	Adsorptive Separation: Using rigid PCNs/COFs with pore chemistry/size for one‐step, high‐purity **C_3_H_6_** capture.	Potential for Displacement (Long‐term). Advantages: Promises a one‐step, low‐energy alternative. Recent materials exhibit record selectivity and capacity, potentially replacing this energy‐intensive process with low‐energy physisorbent alternatives.
CO_2_ Capture (Post‐Combustion)	Amine scrubbing	Energy: High (∼3.5–4.5 GJ/ton CO_2_ for regeneration) Constraints: High energy penalty for solvent regeneration (120‐140°C), solvent degradation (oxidation, corrosion), environmental concerns with waste streams.	Physisorption on Solid Sorbents: Using PCNs/COFs (e.g., **Mg‐MOF‐74**, **SIFSIX‐3‐Cu**, **COF‐102**) with high CO_2_ capacity at flue gas conditions (∼40–60°C, 1 bar).	Complement, aiming for Displacement. Advantages: Much lower regeneration energy (80‐100°C via Temperature Swing Adsorption – TSA), faster adsorption kinetics, no corrosive liquids.
CO_2_ Capture (Pre‐Combustion)	Physical solvents (e.g., Selexol)	Energy: Moderate‐High (for solvent regeneration and refrigeration) Constraints: Operates at high pressure (∼20–70 bar), co‐absorption of other gases (H_2_S).	Pressure Swing Adsorption (PSA): Using PCNs/COFs (e.g., **Ni‐MOF‐74**, **COF‐105**) with high CO_2_ capacity at elevated pressures.	Strong Potential to Displace. Advantages: PSA cycles are highly compatible with high‐pressure streams. PCNs/COFs can offer higher selectivity and capacity over physical solvents, simplifying the process.
CH_4_/N_2_ (Natural Gas Purification)	Cryogenic distillation	Energy: High Constraints: Technically challenging and expensive due to the very low boiling points of both components. Requires significant compression and cooling.	Adsorptive Separation: Using PCNs (e.g., **SIFSIX‐3‐Cu**, **Ni‐MOF‐74**) with tailored pores for strong N_2_ binding over CH_4_.	Displace (Primary target for new tech). Advantages: Cryogenic distillation is rarely used due to cost; current methods (e.g., PSA with zeolites) are inefficient. PCNs/COFs with high N_2_ uptake offer a potentially transformative, low‐energy solution.
Hydrocarbon Separations (Xylene Isomers)	Crystallization/simulated moving bed (SMB) chromatography	Energy: High (for refrigeration in crystallization; complex operation for SMB) Constraints: Crystallization is batch‐wise; SMB is complex with continuous counter‐current flow of solid and liquid.	Adsorptive Separation: Using rigid PCNs/COFs (e.g., **MIL‐125**, **COF‐5**) with shape‐selective pores to differentiate between isomers (e.g., p‐xylene vs. o‐xylene/m‐xylene).	Displace (Demonstrated success). Advantages: PCN‐based benchmark separations of xylene isomers is a landmark success. It offers a continuous, more efficient, and lower‐energy process than crystallization.

## 2 Crystal Engineering of Reticular Materials

**SCHEME 1:**
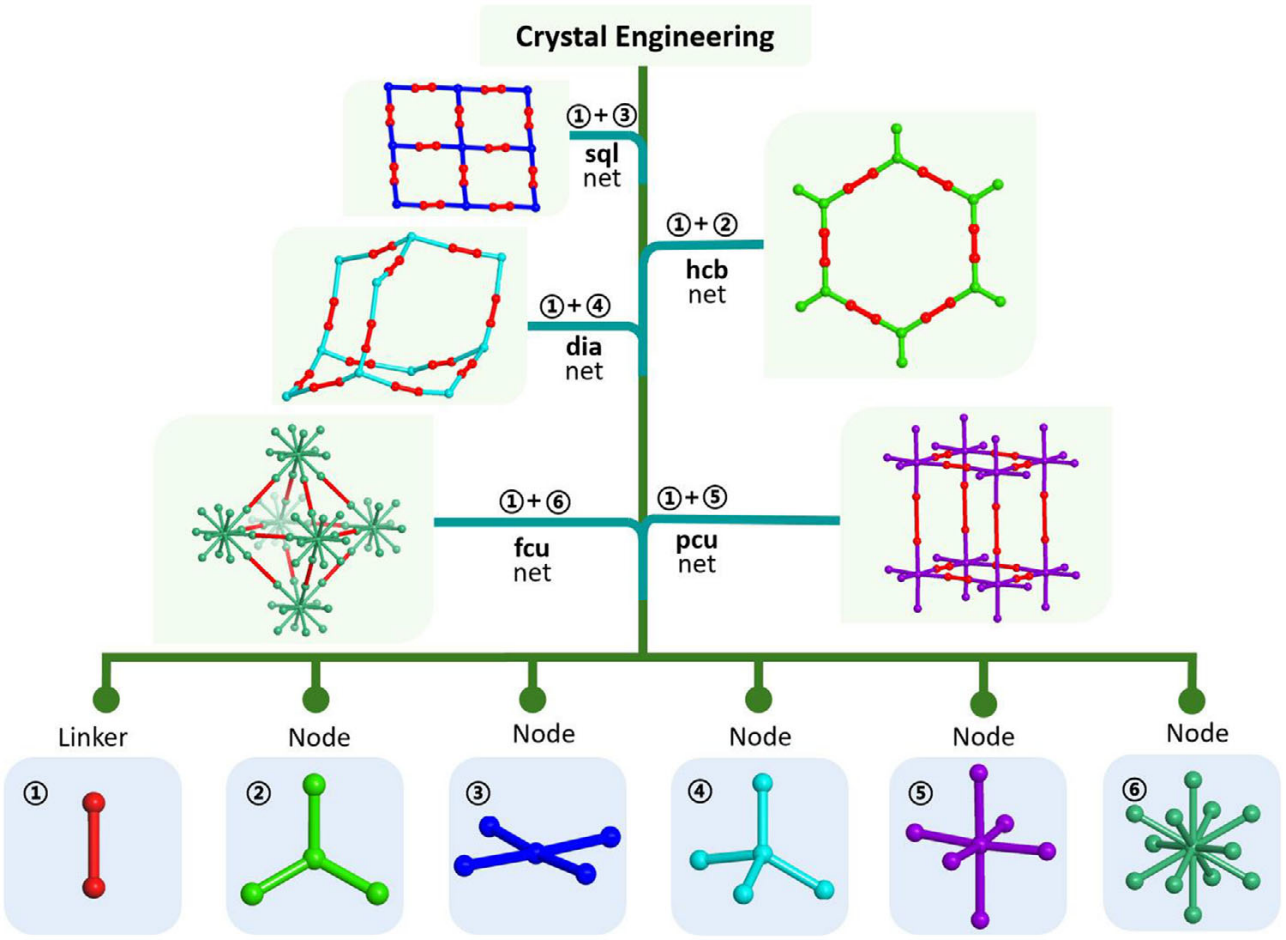
A schematic illustration of the “node and linker” approach to the crystal engineering of reticular networks exemplified by self‐assembly of linkers ① and commonly used nodes ②‐⑥ (3‐, 4‐, 4‐, 6‐ and 12‐ connected nodes, respectively).

Crystal engineering is the field of chemistry that studies the design, properties, and applications of crystalline solids and has rapidly evolved from its initial focus on crystal design to its current emphasis on controlling bulk properties [14]. It was not until 1989 that principles for design of coordination networks (CNs) were presented by Hoskins and Robson, in an article that laid the foundation for the design of new CNs by following the principles of self‐assembly and crystal engineering to generate a diamondoid (**dia**) topology network [15]. They followed up in 1990 with an article that introduced the important concept of modular self‐assembly through the “node‐and‐linker” approach [16] to generate an interpenetrated square lattice (**sql**) topology network that laid the groundwork for future development of reticular crystalline materials, including porous CNs (PCNs) [17, 18, 19]. A linker refers to a ligand (organic or inorganic) that connects two adjacent nodes, a node being any chemical entity that has three or more points of connection [20]. As illustrated in Scheme 1, the role of the linker is to propagate the inherent connectivity and geometry of the node. In such a manner, families of CNs based upon topologies such as **dia**, **sql**, primitive cubic (**pcu**), or honeycomb (**hcb**) are accessible through use of metal cations as 4‐connected (4‐c) tetrahedral, 4‐c square planar, 6‐c octahedral or 3‐c trigonal nodes, respectively. Metal clusters and organic moieties with three or more connections were also soon introduced as nodes, followed by high connectivity polyhedral nodes that can generate high symmetry CNs, e.g., face centered cubic (**fcu**) topology networks can be generated by 12‐c nodes. Since selected nodes typically pre‐existed as molecular compounds, the term molecular building block (MBB) was coined [21], or, in the case of polyhedral nodes, supermolecular building block (SBB) [22]. The network structures depicted in Scheme 1 are representative examples of network structures that can be designed by the *node and linker* crystal engineering approach and, as we now know, there are many potential nodes and many linkers, thus far resulting in >131,682 CNs archived in the metal‐organic framework (MOF) subset of the Cambridge Structural Database (CSD), specifically, the CSD release 2025.1 (version 6.00, build 0.617, from May 2025 [23]. Inspired by Robson's *node and linker* crystal engineering approach introduced in 1989 [16], the design of families of CNs advanced rapidly in the 1990s. In 1994, Fujita and colleagues reported a non‐interpenetrated variant of Robson's 1990 CN with **sql** topology [16], and MacGillivray et al. reported interpenetrated **dia** and **hcb** nets [24]. The **dia** and **sql** CNs are based upon 4,4′‐bipyridine (4,4′‐bipy) linkers and 4,4′‐bipy is the most commonly used linker in the MOF subset of the CSD with 8592 entries (CSD release 2025.1, version 6.00, build 0.617, from May 2025). Fujita's **sql** net was studied by single‐crystal X‐ray diffraction (SCXRD), which revealed clathration of *o*‐dibromobenzene [25]. Such **sql** nets remain of topical interest as they are amenable to fine‐tuning through metal, linker or counterion substitution. That multiple moieties can be substituted is profound from a crystal engineering perspective as second generation, Gen‐2, materials can be formed by systematic variation of composition that in turn modifies pore size, shape and chemistry [17]. The following year, Moore and Lee coined the term MBB [21], which can be defined as a known molecule or coordination complex that can be used to construct network solids. MBB‐based nodes have since been widely employed in the construction of CN families, as exemplified by CNs containing metal carboxylate clusters, including 4‐*c* nodes such as the “paddlewheel” cluster, M_2_(RCOO)_4_. **sql** topology CNs based upon paddlewheel clusters of formula [M_2_(1,4‐bdc)_2_]_n_, 1,4‐bdc = 1,4‐benzenedicarboxylate, were reported to be porous by Mori et al. (M = Cu) and Yaghi et al. (M = Zn) in 1997 [26] and 1998 [27], respectively. Also in 1997, Kitagawa's group reported the methane sorption properties of [Co_2_(4,4′‐bpy)_3_(NO_3_)_4_]_n_, the first report of permanent porosity in CNs [28]. Shortly thereafter in 1999, the first PCN with ultra‐high surface area, **HKUST‐1** ([Cu_3_(BTC)_2_]_n_, a.k.a., Cu‐BTC, BTC = 1,3,5‐benzene‐tricarboxylate) [29], was reported. **HKUST‐1** is comprised paddlewheel MBB nodes and demonstrated that PCNs can exhibit properties previously unattainable in earlier classes of physisorbents. The gravimetric surface area record set by **HKUST‐1** was soon eclipsed by **MOF‐5**, also reported in 1999 [30].

Distinct from MOFs in composition are “hybrid” CNs comprised of combinations inorganic and organic linker ligands. The prototypal CN of this type, [Zn(4,4′‐bipy)_2_(SiF_6_)]_n_, **SIFSIX‐1‐Zn**, was reported as a non‐interpenetrated **pcu** topology CN in 1995 by Zaworotko's group [31] and the Cu variant was subsequently studied for its methane storage properties by Kitagawa et al. [32] Such CNs are also highly modular as they can be regarded as pillared **sql** nets, meaning that there are at least three variables (linker, pillar and metal) for fine‐tuning of pore size, shape and chemistry. An ultramicroporous variant, [Zn(pyrazine)_2_(SiF_6_)]_n_, **SIFSIX‐3‐Zn**, was found to offer exceptional performance for trace capture of CO_2_ and is the prototypal hybrid ultramicroporous material, HUM [33], although such structures had been known since the 1990s [34]. HUMs marked an important step forward in terms of HC separation as their narrow pores, when integrated with fluoro‐rich pore walls, afforded tight binding enhanced by strong electrostatics, thereby enabling new benchmarks for trace capture of several light HCs, including C_2_H_2_ [35, 36]. Gen‐2 HUMs have resulted in further improvements in adsorptive separation performance by at least two orders of magnitude [37, 38, 39, 40]. Pillaring of [M_2_(1,4‐bdc)_2_]_n_ **sql** nets was also used as the design principle to prepare a large family of MOFs derived from **DMOF‐1**, **DMOF‐1** being comprised of [M_2_(1,4‐bdc)_2_]_n_ nets pillared by dizazabicylooctane, dabco [41].

Several design approaches to PCNs were therefore established by 2000, although several notable developments followed. In 2003, X. M. Chen and colleagues introduced the first metal azolate framework (MAF) with zeolitic (sodalite, **SOD**) topology (**MAF‐3**), [Zn(bim)_2_] (bim = benzimidazolate) [42]. Chen's work spawned development of the family of adsorbents now commonly known as zeolitic imidazolate frameworks (ZIFs) [43, 44]. Also in 2003, Yaghi and O'Keeffe introduced and defined the concept of reticular synthesis, i.e. the design of reticular materials using pre‐determined building blocks to form families of extended solid‐state network architectures [45]. In 2004, Zaworotko's group introduced the idea supermolecular building blocks (SBBs), CNs built by self‐assembly of metal–organic polyhedra (MOPs) [22]. SBBs can offer high connectivity (≥8) and typically better control over network topology than low connectivity nodes. Prototypal examples of SBBs that form PCNs include 12‐c [M_6_(bdc)_12_]^12−^ (M = Ni, Co) [46] and the 24‐c “nanoball” SBB, Cu_24_(1,3‐bdc)_24_ [47]. In 2005, Yaghi's group introduced covalent organic frameworks (COFs), porous networks comprised solely of organic nodes and linkers, by following the same design principles used to design reticular CNs [48]. Also in 2005, O'Keeffe and Yaghi reported CNs featuring metal‐based rod building blocks (RBBs) [49]. The coordination environment of RBBs in 3D CNs (e.g., the **M‐MOF‐74** family based upon **Zn‐MOF‐74** ([Zn_2_(dobdc)]_n_; H_4_dobdc = 2,5‐dioxido‐1,4‐benzenedicarboxylic acid) [49]) typically precludes interpenetration, thereby enabling extra‐large porosity by extending the linker length.

With design rules in place by the mid‐2000s, attention shifted to structure‐function relationships. While coordination complexes and organic molecules have long been known for their ability to include guest molecules (clathrate formation) [50, 51], often involving transformations between closed and open phases (switching), the first examples of 2D and 3D switching CNs were not reported until the early 21^st^ century [52, 53]. Werner complexes, the prototypical coordination compounds, had their guest‐clathration capabilities systematically explored by Schaeffer in 1957 [51]. In 1969, Barrer's group reported sorption isotherms of Werner complexes upon exposure to different gases and vapors [50]. Specifically, when **[Co(etpy)_4_(NCS)_2_]** (etpy = 4‐ethylpyridine) was exposed to benzene, toluene, and xylenes, switching (stepped) adsorption isotherms were observed for some gases, later attributed to phase transformations between non‐porous and large pore forms of the sorbent [50, 54]. In 1997, the first gas sorption studies of PCNs that verified permanent porosity were reported by the group of Kitagawa [28], the 3D CN, {[M_2_(4,4′‐bpy)_3_(NO_3_)_4_](H_2_O)_x_}_n_ (M = Co., x = 4; M = Ni, x = 4; M = Zn, x = 2). Mori [26] studied the 2D CN, [Cu_2_(1,4‐bdc)_2_]_n_, and shortly thereafter, Yaghi's group reported the sorption properties of the Zn(II) analogue, [Zn_2_(1,4‐bdc)_2_]_n_, **MOF‐2** [27]. In 1998, Kitagawa and colleagues introduced the concept of third‐generation CNs or flexible CNs [55], which later became recognized as “soft” or stimuli‐responsive CNs [56, 57]. The first CNs with ultra‐high surface areas, **HKUST‐1**, [Cu_3_(1,3,5‐benzenetricaboxylate)_2_]*_n_* [29], and **MOF‐5** [30] ([Zn_4_O(bdc)_3_, bdc = benzene‐1,4‐dicarboxylate]), were reported in 1999. **HKUST‐1** and **MOF‐5**, the latter with a specific surface area >3000 m^2^/g, demonstrated that PCNs can exhibit properties that were hitherto unattainable in previous classes of sorbents. The study of porous materials with extra‐large surface areas remains a topical subject. The groups of Hupp and Kaskel extended the upper limit of Brunauer–Emmett–Teller (BET) surface area to approximately 7000 m^2^/g in **NU‐110** [Cu_3_(L^6^–_(110)_)(H_2_O)_3_]_n_ (L^6^–_(110)_ = the hexa‐anion) [58], and 7800 m^2^/g in **DUT‐60** (Zn_4_O(bbc)_4/3_(bcpbd), bbc = 1,3,5‐tris(4′‐carboxy [1,1′‐biphenyl]‐4‐yl)benzene, bcpbd = 1,4‐bis‐*p*‐carboxyphenylbuta‐1,3‐diene), respectively.

HC separation was one of the first potential applications addressed by reticular sorbents, and a large body of subsequent work has provided insight into selective binding sites and the supramolecular chemistry that drives sorbent‐sorbate binding (Scheme 3). For example, Kitagawa's group in 2004 reported on the first “shape responsive” sorbent which exhibited induced fit binding of benzene, {[Cu_2_(pzdc)_2_(bpy)]}_n_ (pzdc = pyrazine‐2,3‐dicarboxylate), **CPL‐2** [59]. In 2005, the same group reported C_2_H_2_/CO_2_ selectivity in **CPL‐1**, i.e., [Cu_2_(pzdc)_2_(pyz)]*_n_*, (pyz = pyrazine) [60]. The crystallographically determined C_2_H_2_ binding sites in **CPL‐1** channels revealed H‐bonding between non‐coordinated oxygen atoms of **CPL‐1** and C_2_H_2_ [60]. In 2001, Kitagawa's prediction of “third‐generation” flexible porous coordination polymers, PCPs [55], was validated by Kaneko et al. when they studied the two‐dimensional (2D) CN **Cu(4,4′‐bpy)_2_(BF_4_)_2_** (**ELM‐11(Cu)**, ELM = Elastic Layered Material, or **sql‐1‐Cu‐BF_4_**) [52]. In 2007, Kajiro and Kanoh's work on **ELM‐12**, [Cu(bpy)_2_(OTf)_2_]_n_ (MOF; OTf^−^ = triflate, also known as **sql‐1‐Cu‐OTf** [61]) confirmed that the observed guest‐induced stepped isotherms (type F‐II isotherm) [54] were a manifestation of a “*breathing effect*” from a less open phase to a more open phase. Such structural flexibility can also play a role in HC separation. That same year, Kitagawa's group reported benzene (Bz)‐selective binding over cyclohexane (Cy) driven by CH···π interactions between the flexible host framework {[Zn(*μ*_4_‐TCNQ−TCNQ)bpy] (TCNQ = tetracyanoquinodimethane) and Bz molecules [62]. Although unsaturated metal centers (UMCs) had been previously reported in **HKUST‐1** [29] and **Zn‐MOF‐74** [49], they were not utilized as sorbate binding sites until Matzger's work on CO_2_ capture at low pressure in 2008 [63], followed shortly thereafter by B. Chen's work on C_2_H_2_ capture by **HKUST‐1** [64]. Subsequently, Yaghi's and B. Chen's groups independently investigated the influence of UMCs on CO_2_‐selective and C_2_H_2_‐selective separation over N_2_ and CO_2_, respectively, in **Mg‐MOF‐74,** [Mg_2_(dobdc)]_n_ and **UTSA‐74**, [Zn_2_(H_2_O)(dobdc)·0.5H_2_O]_n_ [65]. Long's group also exploited UMCs for C_2_H_6_ and propane (C_3_H_8_) purification driven by interactions between UMCs and olefinic HCs in **Fe‐MOF‐74**, e.g., **[Fe_2_(dobdc)]** [66]. However, in sorbents that exploit UMCs for separation, the coordination of atmospheric water by UMCs can hinder real‐world applications [67, 68, 69].

As mentioned earlier, the Cu analog of **SIFSIX‐1‐Zn**, **SIFSIX‐1‐Cu**, exhibits excellent methane (CH_4_) storage performance as detailed by Kitagawa's group in 2000 [32]. The stronger electrostatics associated with inorganic linker ligands was fully exploited when gas separation selectivity an order of magnitude better than the previous best‐performing CO_2_ selective physisorbents was observed in an ultramicroporous variant of **SIFSIX‐1‐Zn** [31]. This material, the prototypical HUM **SIFSIX‐3‐Zn** ([Zn(pyrazine)_2_(SiF_6_)]*_n_*, was reported in 2013 to be the first physisorbent with ultrahigh (>1000 at ≤10,000 ppm) trace CO_2_/N_2_ selectivity and also set a new benchmark for CO_2_/CH_4_ selectivity (feed ratio = 1:1, temperature: 298 K, total pressure: 1 bar) [33]. The related **pcu** topology sorbent, **SIFSIX‐2‐Cu** ([Cu(dpa)_2_(SiF_6_)]*_n_*, dpa = 4,4′‐dipyridylacetylene), and its interpenetrated variant, **SIFSIX‐2‐Cu‐i**, highlighted the profound impact of pore size and pore chemistry on sorption properties, with the latter being the first sorbent of any type with high enough selectivity to enable removal of trace (1%) C_2_H_2_ from C_2_H_4_ [35]. Subsequently, by using three ultramicroporous sorbents in series (each suited for a specific trace gas impurity), Zaworotko and B. Chen's groups demonstrated that synergistic sorbent separation technology produces polymer‐grade C_2_H_4_ (purity >99.9%) in a single‐step process [70].

In 2013, Long's group reported that the triangular‐shaped pores of **Fe_2_(BDP)_3_** (BDP^2−^ = 1,4‐benzenedipyrazolate) enabled separation of hexane isomers [71]. An example of fine‐tuning pore size by crystal engineering was reported by Feng's group in 2020, whereby a pore‐space‐partitioning strategy was introduced to adjust the pore size of **Fe‐MOF‐74** for C_2_H_6_/C_2_H_4_ separation [72]. Isostructural to **MOF‐5**, **CUB‐5**, [Zn_4_O(1,4‐cdc)_3_], features the substituted aliphatic linker cdc, cubane‐1,4‐dicarboxylate, and methine units orient towards the pores, providing adsorption sites for the π and CH moieties of Bz [73]. In 2021, Shimizu and co‐workers reported an easily scalable, low‐cost, moderately hydrophobic MOF, **CALF‐20** ([Zn_2_(1,2,4‐triazolate)_2_(oxalate)]), with properties suitable for CO_2_ capture from flue gas. Under humid conditions, **CALF‐20** preferentially adsorbs CO_2_ and can be readily regenerated [74].

In 2022, Rosseinsky's group introduced the use of machine learning to explore MOFs for evaluating guest accessibility, which is crucial for several HC separations [75]. This approach has the potential to be an effective tool for screening thousands of hypothetical and literature‐reported MOFs from the CSD MOF database [76, 77], including the Computation‐Ready, Experimental (CoRE) MOF database [78], to identify the most likely separations that can be targeted by particular MOFs. More recently, in 2023, a cage‐on MOF strategy was applied to incorporate porous coordination cages (PCCs) onto the external surface of a MOF for selective dye adsorption using **PCN‐222@PCCs** (PCN‐222, Zr_6_(μ_3_‐OH)_8_(OH)_8_‐(TCPP)_2_, TCPP = meso‐tetra(4‐carboxyphenyl)porphyrin) and **MIL‐101@PCCs** (**MIL‐101**, Cr_3_F(H_2_O)_2_O(1,4‐bdc)_3_, 1, 4‐bdc = 1,4‐benzendicarboxylic acid) [79]. In 2024, Xing's group investigated a molecular sieving mechanism in a cage‐type structure **ZU‐609** (Cu(eds)(dps)_2_, eds = 1,2‐ethanedisulfonate, dps = 4,4′‐dipyridylsulfide) with 1D channel‐shaped narrow windows and 3D cage‐shaped large voids. **ZU‐609** broke the trade‐off between separation selectivity and capacity [80], aligned with the Zaworotko group's earlier 2021 report on overcoming this *Catch‐22* [81]. The limited diffusion path functions as a sieve to exclude large molecules, while the coexisting large channel allows for the rapid diffusion of adsorbed molecules.

**SCHEME 2:**
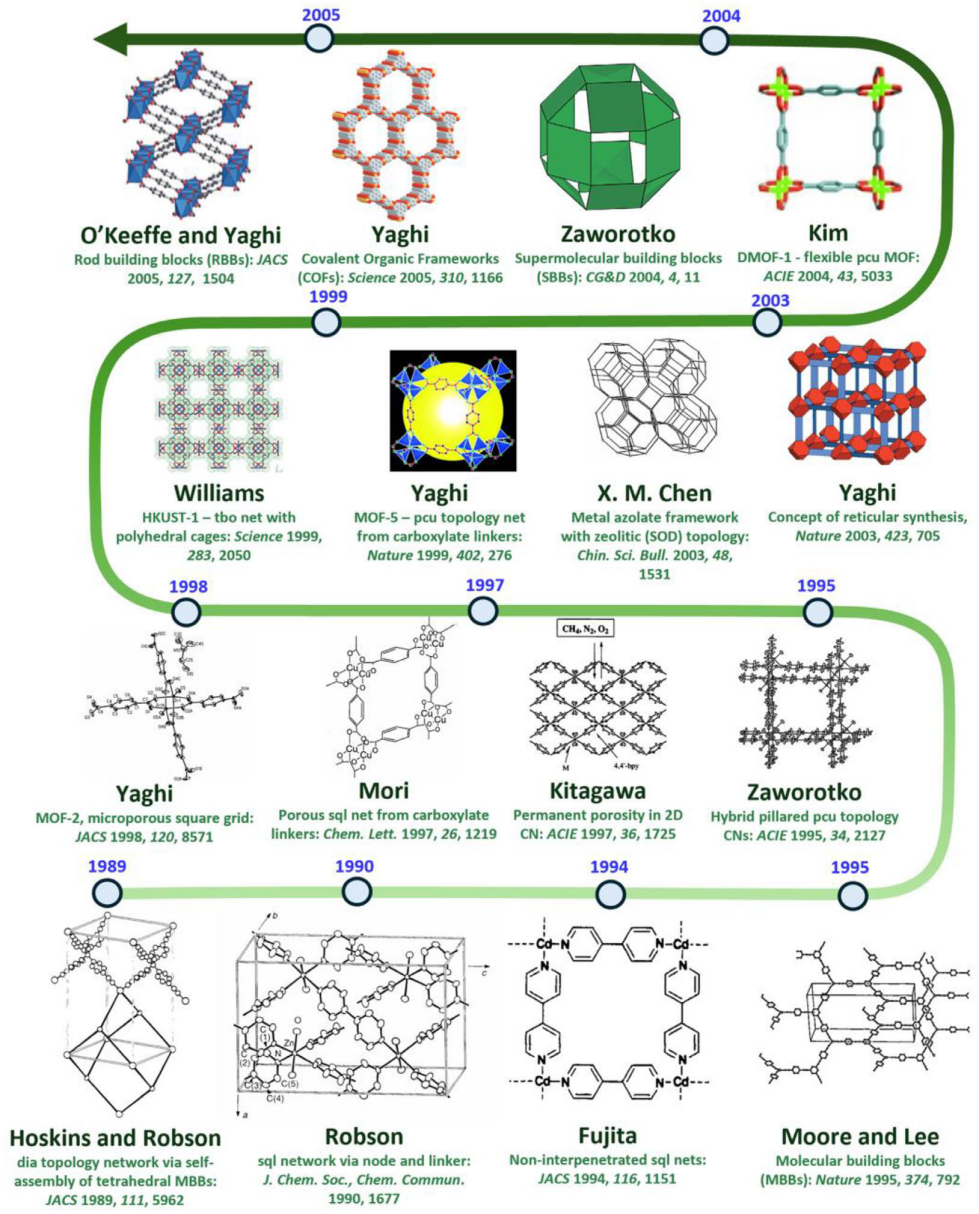
Chronology of key discoveries in the crystal engineering of reticular materials. Reprinted with permissions from ref [15, 16, 21, 22, 25, 26, 27, 28, 29, 30, 31, 41, 42, 45, 48, 49]. Copyright 1989, Royal Society of Chemistry; Copyright 1990, Royal Society of Chemistry; Copyright 1994, The American Association for the Advancement of Science; Copyright 1995, Springer Nature; Copyright 1995, Wiley‐VCH Verlag GmbH & Co. KGaA, Weinheim; copyright 1997, Wiley‐VCH Verlag GmbH & Co. KGaA, Weinheim; Copyright 1997, The Chemical Society of Japan; Copyright 1998, The American Association for the Advancement of Science; Copyright 1999, The American Association for the Advancement of Science; copyright 1999, Springer Nature; Copyright 2003, Springer Nature; Copyright 2003, Springer Nature; Copyright 2004, Wiley‐VCH Verlag GmbH & Co. KGaA, Weinheim; Copyright 2004, The American Association for the Advancement of Science; Copyright 2005, The American Association for the Advancement of Science; Copyright 2005, The American Association for the Advancement of Science.

**SCHEME 3:**
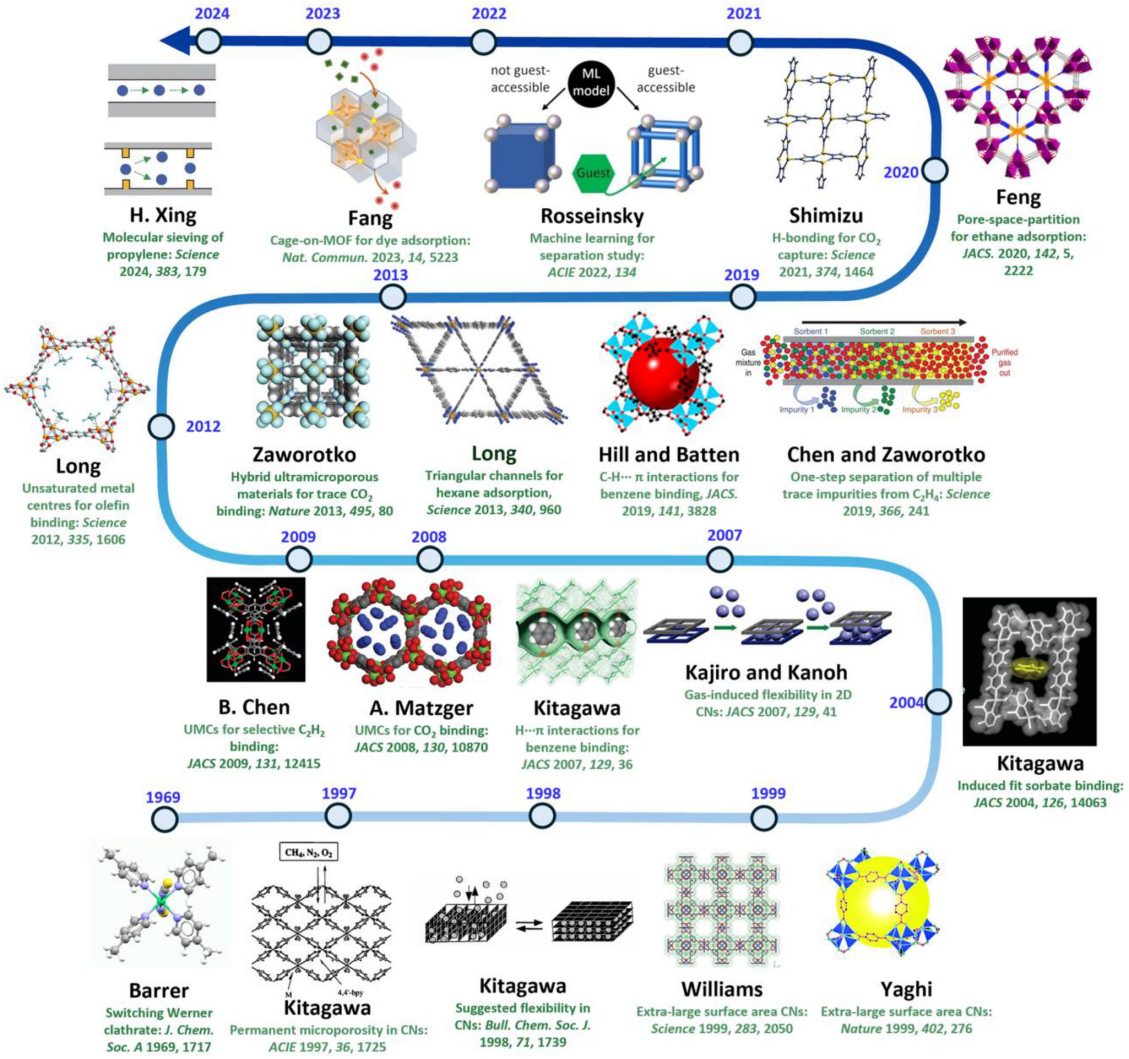
Key discoveries concerning structure‐function relationships in physisorbents with particular emphasis upon binding sites that drive strong performance for hydrocarbon separation and purification. Reprinted with permissions from ref [28, 29, 30, 33, 50, 55, 59, 61, 62, 63, 64, 66, 70, 71, 72, 73, 74, 75, 79, 80]. Copyright 1969, Royal Society of Chemistry; copyright 1997, Wiley‐VCH Verlag GmbH & Co. KGaA, Weinheim; copyright 1998, The Chemical Society of Japan; Copyright 1999, The American Association for the Advancement of Science; Copyright 1999, Springer Nature; Copyright 2004, The American Association for the Advancement of Science; Copyright 2007, The American Association for the Advancement of Science; Copyright 2007, The American Association for the Advancement of Science; Copyright 2008, The American Association for the Advancement of Science; Copyright 2009, The American Association for the Advancement of Science; Copyright 2012, The American Association for the Advancement of Science; copyright 2013, Springer Nature; Copyright 2013, The American Association for the Advancement of Science; Copyright 2019, The American Association for the Advancement of Science; Copyright 2019, The American Association for the Advancement of Science; Copyright 2020, The American Association for the Advancement of Science; Copyright 2021, The American Association for the Advancement of Science; Copyright 2022, Wiley‐VCH Verlag GmbH & Co. KGaA, Weinheim; Copyright 2023, Springer Nature; Copyright 2024, The American Association for the Advancement of Science.

As indicated by Scheme 2, the key design concepts that afforded the most common families of PCNs were developed over two decades ago, and the separation potential of such materials started to be addressed shortly thereafter. This review focuses upon advances made in terms of properties and insight with respect to HC separation by physisorbents (Scheme 3), with particular emphasis upon two distinct classes of crystalline adsorbents, PCNs (including MOFs and HUMs) and COFs. Therefore, in the following sections we highlight examples of MOFs, HUMs and COFs for HC separation in the gas phase (C1‐C4 for the following separations: CH_4_/N_2_, N_2_/CH_4_, CO_2_/CH_4_, C_2_H_2_/C_2_H_4_, CO_2_/C_2_H_2_, C_2_H_4_/C_2_H_6_, C_2_H_6_/C_2_H_4_, C_3_H_4_/C_3_H_6_, C_3_H_6_/C_3_H_8_, multicomponent HC mixtures including those comprising CO_2_ and C4 isomers) and the liquid phase (separation of C6 isomers and C8 isomers). To obtain insight into the observed separation performance, the top‐performing reticular sorbents for each binary/multicomponent mixture are identified and analyzed with regard to their structure‐property relationships. Nine features have been identified by us and others as playing a key role in observed HC separation performance: H‐bonding; electrostatics; kinetics; flexibility‐driven induced fit; Van der Waals interactions; π interactions (C‐H···π interactions and π···π interactions); polarizability; pore size; UMCs. The relevance of each parameter to the top‐performing reticular sorbents for each separation is evaluated through radar plots in each of the following sections.

As a general rule, when comparing separation performances in tabular form, this article ranks the top‐performing reticular sorbents in order of decreasing adsorption selectivity, from top to bottom (Tables 2, 3, 4, 5, 6, 7, 8, 9, 10, 11, 12, 13, 14, 15). For binary separations, feed compositions were typically reported using equimolar ratios (1:1, v/v), though other variations were also examined depending on the context, including 1:2, 1:9, 1:99, 0.5:99, and 2:98. Most hydrocarbon separation studies were conducted at 298 K, although the full temperature range studied extends from 233 K to 523 K.

**TABLE 2:** Key physicochemical properties of adsorptives relevant to HC purification [82, 83, 84, 85, 86].

Molecule	Structure	Kinetic diameter (Å)	Molecular size (Å^3^)	Boiling point (K)	Polarizability (× 10^−25^/cm^3^)	Dipole moment (×10^18^/esu cm^2^)	Quadrupole moment (×10^26^/esu cm^2^)
**CH_4_**	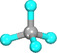	3.8	3.7 × 3.7 × 3.7	111.6	26	0	0
**N_2_**	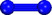	3.64	—	77.3	17.6	0	1.52
**CO_2_**	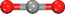	3.3	3.18 × 3.33 × 5.36	194.7	29.11	0	−4.30
**C_2_H_2_**	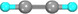	3.3	3.32 × 3.34 × 5.70	189.3	33.3‐39.3	0	7.50
**C_2_H_4_**	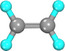	4.163	3.28 × 4.18 × 4.84	169.4	42.52	0	1.50
**C_2_H_6_**	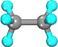	4.443	4.08 × 3.81 × 4.82	184.5	44.3‐44.7	0	0.65
**C_3_H_4_ (MA)**	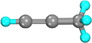	4.2	4.16 × 4.01 × 6.51	250	55.5	0.75	—
**C_3_H_4_ (PD)**	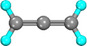	—	6.2 × 3.8 × 3.8	239	56.9	0	—
**C_3_H_6_**	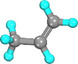	4.678	4.16 × 4.65 × 6.44	225.4	62.6	0.366	—
**C_3_H_8_**	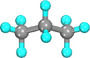	4.3‐5.118	4.02 × 4.52 × 6.61	231.1	62.9‐63.7	0.084	—
**C_4_H_6_**	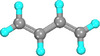	5.2	3.4 × 5.36 × 7.84	268.6	—	0	—
***i*‐C_4_H_8_**	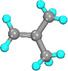	4.23	4.52 × 5.76 × 6.71	276.87	—	0.253	—
***n*‐C_4_H_8_**	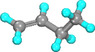	4.83	4.17 × 5.05 × 7.87	266.77	81	0.3	—
**2‐C_4_H_8_**	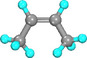	4.49	4.2 × 5.5 × 7.6	276.87	—	0.253	—
***i*‐C_4_H_10_**	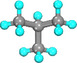	5.278		261.34	81.4‐82.9	0.132	—
***n*‐C_4_H_10_**	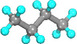	4.687	4.2 × 4.6 × 8.1	272.66	82	0.05	—
**C_6_H_6_**	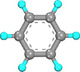	5.349‐5.85	6.6 × 7.3 × 3.3	353.24	100‐107.4	0	—
***c*‐C_6_H_12_**	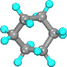	6.0‐6.182	7.2 × 6.6 × 5.0	343.93	107.7‐110	0	—
***n*‐C_6_H_14_ (*n‐*HEX)**	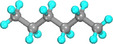	4.3	9.7 × 4.5 × 4.0	341.88	119	0	—
***i*‐C_6_H_14_ (2MP)**	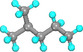	5.5	9.2 × 6.4 × 5.3	333.4	—	0.1	—
***neo*‐C_6_H_14_ (22DMB)**	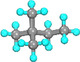	6.2	8.0 × 6.7 × 5.9	322.87	—	—	—
**3‐methylpentane (3MP)**	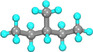	5.5	9.3 × 6.2 × 5.2	336.40	—	—	—
**2,3‐Dimethylbutane(23DMB)**	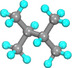	5.6	7.8 × 6.7 × 5.3	331	—	—	—
**Ethylbenzene**	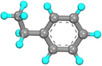	6.7	9.3 × 7.0 × 4.0	409.36	142	0.59	—
***p*‐Xylene**	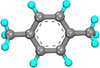	6.7	9.0 × 6.5 × 4.0	411.53	137‐149	0.1	—
***m*‐Xylene**	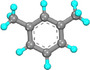	7.1	8.4 × 7.2 × 4.0	412.34	142	0.37	—
***o*‐Xylene**	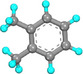	7.4	7.7 × 7.2 × 4.0	417.59	141‐149	0.640	—

**Abbreviations**: MA: methylacetylene; PD: propadiene; *n*‐HEX: *n*‐hexane; 3MP: 3‐methylpentane; 2MP: 2‐methylpentan; 22DMB: 2,2‐dimethylbutane; 23DMB: 2,3‐dimethylbutane.

**TABLE 3:** CH_4_/N_2_ and N_2_/CH_4_ binary separations. The following parameters are listed for comparison: pore size; BET surface area (S_BET_); single‐component gas uptakes; adsorption enthalpies (*Q*_st_); adsorption selectivity; regeneration temperature; and attributed mechanisms. Reticular sorbents are listed in decreasing order of binary selectivity for CH_4_‐selective sorbents (above) and N_2_‐selective sorbents (underneath).

Binary	Adsorbent, network dimensionality (nD)	Pore size^e^ (Å)	CH_4_ uptake at 1 bar (mmol/g)	N_2_ uptake at 1 bar (mmol/g)	*Q*_st_ (kJ/mol)	Binary Selectivity^a^	Regeneration temperature (K)	S_BET_ ^e^ (m^2^/g)	Mechanism	Refs.
CH_4_/N_2_	**Ni(ina)_2_**, 3D	5.0 × 5.8	1.82	0.53	28.0/17.9	15.8	298	470	C‐H···π interactions	[87]
	**Al‐CDC**, 3D	5.4 × 5.4	1.43	0.23	27.5/18.6	13.1	—	380	Van der Waals interactions	[88]
	**Co_3_(C_4_O_4_)_2_(OH)_2_**, 3D	4.1 × 4.3	0.4	0.19	25.1/18.1	12.5	373	—	Van der Waals, electrostatics	[89]
	**Al‐fumarate**, 3D	5.7 × 6	1.14	0.2	15.9/6.3	11.7	393	1130	C‐H···π interactions	[90]
	**SBMOF‐1**, 3D	7.3 × 7.3	0.92	0.18	23.2/16.1	11.5	—	149.7	Van der Waals interactions	[91]
	**STAM‐1**, 3D	5 × 5; 7 × 7	0.63	0.11	20.0/15.0	10.8	298	110	Van der Waals interactions	[92]
	**ATC‐Cu**, 3D	4.43 × 5.39	2.9	0.75	26.8/16.0	9.7	298	600	Electrostatics, van der Waals interactions	[93]
	**ROD‐8a**, 3D	6.5 × 11.8; 8.5 × 9.5	0.77	0.13	16.7	9.1	—	369.3	—	[94]
	**NKMOF‐8‐Me**, 3D	5.7 × 5.7	1.76	0.31	28.0/18.9	9.0	298	626.1	Van der Waals interactions	[95]
	**Ni(3‐ain)_2_**, 3D	5 × 5	2.1	0.65	30.9/17.0	9.0	298	615.5	C‐H···π interactions	[87]
	**MIL‐160**, 3D	5 × 5	0.47	0.14	11/7	8.9	393	1104	C‐H···π interactions	[90]
	**Al‐FUM‐Me**, 3D	5.0^e^	1.21	0.23	24.06/17.41	8.6	298	611.8	C–H···O interactions	[96]
	**Cu(ina)_2_**, 3D	4.7^d^	0.827	0.121	17.5/–	8.34^b^	—	251.8^d^	Polarizability, electrostatics	[97]
	**Ni‐Qc‐5**, 3D	3.6^f^	1.26	0.28	31.5/23.5	7.4	298 vacuum	700^f^	Van der Waals interactions, electrostatics	[98]
	**Ni‐MA‐BPY**, 3D	5.1^e^	1.0	0.21	23.5/19.6	7.4	298	464^e^	CH_4_ polarizability	[99]
	**Co(AIP)(BPY)_0.5_**, 3D	8.8 × 7.9 [100]	1.03	0.26	21.2/–	7.3	—	—	Size sieving	[101]
	**Co‐MA‐BPY**, 3D	5.1^e^	0.92	0.2	22.8/18.4	7.2	298	451^e^	CH_4_ polarizability	[99]
	**CAU‐10‐H**, 3D	5 × 5	0.74	0.2	7.5/5	7.2	393	469	C‐H···π interactions	[90]
	**MIL‐53(Al)**, 3D	7.3 × 7.7	0.57	0.15	15/5	7.1	393	915	C‐H···π interactions	[90]
	**ZIF‐94**, 3D	9.1 × 9.1	1.5	0.36	23.9/20.0	7	298	597	Size sieving	[102]
	**Ni(OAc)_2_L**, 3D	2.5 × 19; 2 × 2	1.76	0.47	27.8/19.8	7.0	—	1168	C‐H⋯arene interactions	[103]
	**Cu(hfipbb)(H_2_hfipbb)_0.5_**, 3D	4.6^e^	0.47	0.13	24.1/19.8	6.9^b^	—	105	Pore size	[104]
	**Ni‐BPZ**, 3D	5.8 × 8.6	1.56	0.4	23/18	6.6	393	790	C‐H···π interactions	[105]
	**TUT‐100**, 3D	8.0^e^	2.02	0.31	23.7/19.6	6.3	—	604	Pore size	[106]
	**[Ni_3_(HCOO)_6_]**, 3D	5 × 6	2.06	1.56	24.82‐19.33/–	6.1^b^	298	233	Surface polarizability	[107]
	**MIL‐120Al**, 3D	5.4 × 4.7	1.5	0.47	20.87/17.83	6.0	298	343	C‐H···π interactions	[108]
	**Ni‐BTC**, 3D	9 × 9	1.67	0.31	—	5.1	—	1088.15	Polarizability	[109]
	**[Co_3_(HCOO)_6_]**, 3D	5 × 6	1.39	0.77	20.03/19.7	5.1^b^	298	294	Surface polarizability	[107]
	**Al‐FUM**, 3D	6.3^e^	0.91	0.23	20.60/16.16	5.1	298	1023.3	—	[96]
	**UiO‐66‐Br_2_**, 3D	7‐8^e^	0.72	0.19	—	5.06	298	622	Polarizable bromine at UMCs	[110]
	**UTSA‐30a**, 3D	3.2 × 3.2	0.63	0.165	—	5	—	592	Pore size	[111]
	**[Mg_3_(OOCH)_6_]_2_**, 3D	4.9^d^	2.64	1.52	24.9‐25.5/–	4.9‐5.3	433	—	UMCs	[112]
	**CFAb‐FumMOF‐2**, 3D	—	0.95	0.22	16.7/–	4.94	—	1116.92	C‐H···O interactions	[113]
	**CFAb‐FumMOF‐1**, 3D	5.9^e^	0.98	0.22	18.64/–	4.87	—	1073.08	C‐H···O interactions	[113]
	**Cu(bpy)_2_(OTf)_2_**, 2D	7.2	0.25	0.07	19.6/16.0	4.8	323	205.6	Flexibility	[61, 114, 115]
	**CFAs‐FumMOF‐1**, 3D	6.8^e^	0.98	0.25	17.84/–	4.73	—	1124.86	C‐H···O interactions	[113]
	**CFAs‐FumMOF‐2**, 3D	—	0.92	0.22	17.34/–	4.73	—	1163.94	C‐H···O interactions	[113]
	**[Cu(Me‐4py‐trz‐ia)]*_n_***, 3D	5.5 × 5.5	1.12	0.30	18.0/12.0	4.2	—	1473	—	[116]
	**Ni(2‐ain)_2_**, 3D	4.8 × 4.8	0.3	0.1	22.9/11.1	4.2	298	—	—	[87]
	**ZIF‐93**, 3D	17.9 × 17.9	0.52	0.16	15.8/12.5	3.6	298	846	—	[102]
	**ZIF‐68**, 3D	10.3^e^	0.40	0.121	15.69/11.88	3.5	—	1283^g^	Electrostatics	[117]
	**Al‐BDC**, 3D	8.2^e^	0.71	0.23	18.81/15.52	3.4	298	1335.6	—	[96]
	**Cu(bpy)_2_(OTf)_2_**, 3D	3.9 × 3.9	0.35	0.11	11.0/10.0	3.2	323	225.4	Flexibility	[61, 114, 115]
	**Co/DOBDC**, 3D	11‐12	1.91	0.12	19.6/–	3.2	298	1089.3	UMCs	[118]
	**Al‐NDC**, 3D	9.2^e^	0.48	0.15	14.89/11.06	3.1	298	1545.2	—	[96]
	**ZIF‐69**, 3D	7.8^e^	0.50	0.138	16.24/12.76	3.0	—	1251^g^	Electrostatics	[117]
	**MIL‐100(Cr)**, 3D	5 × 5; 8 × 8	0.60	0.170	—	3.0	298	1528.7	UMCs	[118]
	**ZIF‐8**, 3D	11.6 × 11.6	0.35	0.14	12.4/9.8	2.8^c^	298	1425	—	[102, 119, 120]
	**MIL‐101(Cr)** (293K), 3D	5‐12 and 18–35^e^	0.65	0.129	15.7/12.0	2.7	—	2560	—	[121]
	**ZIF‐90**, 3D	11.2 × 11.2	0.47	0.20	15.9/12.5	2.3	298	1059	—	[102]
	**Al‐BPDC**, 3D	11.5^e^	0.26	0.12	11.75/9.83	2.2	298	1729.9	—	[96]
	**Ni‐MOF‐74**, 3D	12^d^	2.54	1.92	20.3/–	1.35	298	1405.1^d^	UMCs	[97]
N_2_/CH_4_	**V‐MOF‐74**, 3D (Computational study)	12 × 12	—	—	—	—	—	—	UMCs	[122]
	**Fe‐MOF‐74**, 3D (Computational study)	12 × 12	—	—	5.3/5.5	—	—	—	UMCs	[122]
	**V_2_Cl_2.8_(btdd)**, 3D	—	—	1.9	35/56	72^h^	—	1930	UMCs	[123]
	**MIL‐100(Cr)** (283K), 3D	5 × 5; 9 × 9	0.8	1.64	27/39	8	283	2040	UMCs	[124]
	**TYUT‐96Cr**, 3D	6^e^	1.25	1.03	27/37	4.6	—	—	UMCs	[125]
	**MIL‐101(Cr)−NO_2_**, 3D	7.9, 20.2 and 20.7^e^	0.57	0.72	–/30.01	2.8	298	1907	UMCs	[126]
	**MIL‐101(Cr)** (283K), 3D	16 × 16	0.91	0.96	20/40	1.8	—	4000	UMCs	[127]

^a^ For v/v = 1/1 equimolar mixture, predicted by IAST.

^b^ Calculated from the ratio of Henry constants.

^c^ Determined from theoretical calculations.

^d^ Pore size distribution and BET surface area calculated from the Ar adsorption isotherm at 87K.

^e^ Pore size distribution and BET surface area calculated from the N_2_ adsorption isotherm at 77K.

^f^ Pore size distribution and BET surface area calculated from the CO_2_ adsorption isotherm at 195K.

^g^ Accessible surface area.

^h^ For v/v = 2/98 mixture, predicted by IAST.

**TABLE 4:** CO_2_/CH_4_ binary separations. The following parameters are listed for comparison: pore size; BET surface area (S_BET_); single‐component gas uptakes; adsorption enthalpies (*Q*_st_); adsorption selectivity; regeneration temperature; and attributed mechanisms. Reticular sorbents are listed in decreasing order of binary selectivity for CO_2_‐selective sorbents (above) and CH_4_‐selective sorbents (below).

Adsorbent, network dimensionality (nD)	Pore size (Å)	CH_4_ uptake at 1 bar (mmol/g)	CO_2_ uptake at 1 bar (mmol/g)	*Q*_st_ (CO_2_/CH_4_) (kJ/mol)	Selectivity^a^	Regeneration temperature (K)	S_BET_ (m^2^/g)	Mechanism	Refs.
**Cu‐F‐pymo**, 3D	3.3 × 3.3	—	1.61	29.1/–	> 10^7^	He flow at 298 K	146	Molecular sieving	[128]
**MUF‐16**, 3D	3.6 × 7.6	0.04	2.13	32.3/–	6690	Vacuum or inert gas	214	H‐bonding	[129]
**Qc‐5‐Cu‐sql‐β**, 3D	3.3 × 3.3	0.06	2.16	36/–	3300	—	222	Molecular sieving	[130]
**In(aip)_2_**, 3D	3.4‐3.6	—	1.27	34.3/–	1808	—	223.5	Molecular sieving, ‐NH_2_	[131]
**MUF‐16(Ni)**, 3D	—	0.09	2.13	37.3/–	1220	Vacuum or inert gas	204	H‐bonding	[129]
**PEI‐mediated amine‐MIL‐101(Cr)‐c**, 3D	—	∼0.05	3.6	—	931 (0.5 bar)	—	96.4	UMCs	[132]
**MUF‐16(Mn)**, 3D	—	0.09	2.25	36.6/–	470	Vacuum or inert gas	205	H‐bonding	[129]
**[Zn(odip)_0.5_(bpe)_0.5_]**, 3D	3.6	0.43	5.3	42.3/19.3	376	—	550	H‐bonding, Flexibility	[133]
**NbOFFIVE‐1‐Ni**, 3D	—	0.18	2.31	54/–	366	—	195	F‐CO_2_	[134]
**UiO‐66(N_10%_‐Zr)**, 3D	4^b^	0.25	2.1	35.7/5.9	326	He flow at 393 K	1648	N‐CO_2_, Kinetic	[135]
**SIFSIX‐3‐Zn**, 3D	3.84 × 3.84	—	2.54	45/	231	—	250	Electrostatics	[33]
**TIFSIX‐3‐Ni**, 3D	—	0.22	2.21	50/–	158	333	200	F‐CO_2_	[134]
**ZU‐66**, 3D	3.55 × 3.55	0.21	4.56	35/–	136	He flow at 298 K	177	Molecular sieving	[136]
**SIFSIX‐14‐Cu‐i**, 3D	3.6 × 3.6	0.17	4.71	37.7/–	46.7	He flow at 298 K	612	Molecular sieving	[137]
**NJU‐Bai8**, 3D	4 × 3.3	—	2.57	37.7/–	40.8^c^	—	1103	N‐CO_2_	[138]
**3D‐IL‐COF‐1b**, 3D	—	—	—	—	35.1	—	537	CO_2_, ionic liquid	[139]
**SIFSIX‐2‐Cu‐i**, 3D	5.15 × 5.15	—	5.4	31.9/	33	—	735	Electrostatics	[35]
**IRH‐3**, 3D	4.19 × 4.19	0.07	2.7	—	27	—	525.4	N‐CO_2_	[140]
**3D‐IL‐COF‐1**, 3D	8.3	0.37%	5.34%	—	23.1	—	517	CO_2_, ionic liquid	[139]
**3D‐IL‐COF‐2**, 3D	10.7	0.85%	7.61%	—	22.3	—	653	CO_2_, ionic liquid	[139]
**3D‐IL‐COF‐3**, 3D	12.4	0.33%	4.93%	—	21.5	—	870	CO_2_, ionic liquid	[139]
**Zn‐dmtrz‐mip**, 3D	5.7 × 5.8	0.85	2.92	26.5/–	17.8	Inert gas	463.9	H‐bonding	[141]
**TIFSIX‐2‐Cu‐i**, 3D	—	0.76	4.22	35.8/–	16	—	590	F‐CO_2_	[134]
**[Cu_3_(μ_3_‐OH)(PCA)_3_]**, 3D	3.8 × 9.7	0.67	2.93	31.5/–	15.9	—	583	UMCs	[142]
**Er‐BDC‐NH_2_**, 3D	4.5 × 4.5, 6.0 × 6.0	0.31	1.92	26/17	15	—	539	Dipole‐quadrupole interaction	[143]
**NJU‐Bai7**, 3D	3.4 × 3.4	—	2.91	40.5/–	14.1^c^	—	1155	Pore size	[138]
**MIL‐53‐Al powder**, 3D	7^b^	0.64	2.58	23.27/16.98	14.42	Vacuum	1555	Van der Waals interactions	[144]
**NJU‐Bai35**, 3D	—	—	3.13	33.4/–	11.6	—	863	Molecular sieving	[145]
**MIL‐53‐Al beads**, 3D	8.2 × 8.2	0.51	2.13	21.48/16.20	11.41	Vacuum	981	Van der Waals interactions	[144]
**ZIF‐78**, 3D	7.1 × 7.1	0.67	2.32	—	10.6	—	620	Dipole‐quadrupole interactions	[146]
**Mg‐MOF‐74**, 3D	10.2	1.05	8.61	73/18.5	8	—	1174	UMCs	[147]
**In‐MOF‐3**, 3D	5.0 × 5.0	0.23	0.97	29.4/–	7.8^e^	—	261	H‐bonding, π···π interactions	[148]
**NiNi‐Pyz**, 3D	3.8 × 3.9	4.13	1.44	30/–	7.2	—	453	Electrostatics	[149]
**MIL‐53(Al)**, 3D (303K)	5.1	0.2	1.3	—	7.0	393	—	CO_2_, hydroxyl interaction	[150]
**Sm‐BDC‐NH_2_**, 3D	4.5 × 4.5, 6.0 × 6.0	0.44	1.96	23/18	6.56	—	528	Dipole‐quadrupole interaction	[143]
**Y‐BDC‐NH_2_**, 3D	4.5 × 4.5, 6.0 × 6.0	0.89	3.53	28/19	6.3	—	824	Dipole‐quadrupole interaction	[143]
**CoNi‐Pyz**, 3D	3.9 × 4.1	3.56	1.36	29.2/–	6.0	—	440	Electrostatics	[149]
**MOF‐508b**, 3D (303 K)	4.0 × 4.0	—	1.78	14.9/–	3‐6	—	—	Quadrupole interactions	[151]
**MAF‐66**, 3D	—	1.31	4.41	26/19	5.8	—	1014	N‐CO_2_	[152]
**CNT@Cu_3_(BTC)_2_**, 3D	—	7.49	13.52(18 bar)	—	5.67	—	1458	—	[153]
**In‐MOF‐1**, 3D	8.0 × 8.0	0.24	0.92	24/–	5.5^e^	—	122	H‐bonding, π···π interactions	[148]
**3D‐COF‐1a**, 3D	—	—	—	—	5.3	—	596	CO_2_, ionic liquid	[139]
**In‐MOF‐2**, 3D	6.0 × 6.0	0.50	1.26	26.4/–	5.2^e^	—	255	H‐bonding, π···π interactions	[148]
**Cu‐BTC powder**, 3D (308K)	10	0.77	3.94	15.0/22.8	5.1	423	—	—	[154]
**SYSU**, 3D	6.3 × 6.3	—	3.11	28.2/–	4.7	—	1110	Pore size	[138]
**MIL‐101 powder**, 3D	6‐20^b^	0.42	1.54	26.23/14.82	4.09	Vacuum	2758	Van der Waals interactions	[144]
**MIL‐101 beads**, 3D	6‐20^b^	0.34	1.12	22.62/14.54	3.70	—	1963	Van der Waals interactions	[144]
**Cu3(BTC)_2_**, 3D	6	4.47	6.71(18 bar)	—	2.28	—	1587	—	[153]
**UMCM‐1**, 3D	14	5.01	15.82(18 bar)	—	1.82	—	2932	—	[153]
**ZIF‐8**, 3D	11.6	3.28	7.04(18 bar)	—	1.32	—	1311	—	[153]
**MOF‐177**, 3D	10.6	4.2	17.0 (18 bar)	—	0.89	—	2157	—	[153]
**UTSA‐28**, 3D	3.2 × 4.5, 3.8 × 3.8	0.36	3	42.9/–	—	He flow at 323 K	330	Molecular sieving	[155]

^a^ For CO_2_/CH_4_ v/v = 1/1 equimolar mixture at 1 bar, 298 K, predicted by IAST.

^b^ Pore size distribution, BET surface area calculated from the N_2_ adsorption isotherm at 77K.

^c^ Separation ratios at 273 K.

^d^ Pore size distribution, BET surface area calculated from the CO_2_ adsorption isotherm at 195K.

^e^ For CO_2_/CH_4_ v/v = 15/85 equimolar mixture at 1 bar, 298 K, predicted by IAST.

**TABLE 5:** C_2_H_2_/CO_2_ binary separations. The following parameters are listed for comparison: pore size; BET surface area (S_BET_); single‐component gas uptakes; adsorption enthalpies (*Q*_st_); adsorption selectivity; regeneration temperature; and attributed mechanisms. Reticular sorbents are listed in decreasing order of binary selectivity for C_2_H_2_‐selective sorbents (above) and CO_2_‐selective sorbents (below).

Adsorbent, network dimensionality (nD)	Pore size (Å)	C_2_H_2_ uptake at 1 bar (mmol/g)	CO_2_ uptake at 1 bar (mmol/g)	*Q*_st_ (kJ/mol)	Selectivity^a^	Regeneration temperature	S_BET_ (m^2^/g)	Mechanism	Refs.
**SOFOUR‐TEPE‐Zn**, 3D	3.63 × 3.81, 3.31 × 3.32	3.98	0.63	45.6/26.3	16833	—	410.96	H‐bonding, Van der Waals interactions	[156]
**ZUL‐330**, 2D	3.3 × 3.7	5.91	1.1	—	10086	—	352	H‐bonding, C−H···π interactions	[157]
**ZUL‐430**, 2D	3.0 × 3.3	5.74	1.12	—	3499	—	364	H‐bonding, C−H···π interactions	[157]
**NCU‐100a (SIFSIX‐dps‐Cu)**, 3D	1.4 × 3.0	4.57	0.49	56.3/–	1787	He flow at 298 K	358	H‐bonding	[158]
**UTSA‐300a**, 2D	2.4 × 3.3	3.3	0.2	57.6/–	10^3^	—	311	H‐bonding, van der Waals interactions	[159]
**JNU‐1**, 3D	8	2.9	—	47.6/–	285.6	393	818	Flexibility, dipole interactions	[160]
**Cu^I^@UiO‐66‐(COOH)_2_**, 3D	4.4, 5.0^g^	2.44	0.85	74.5/28.9	185	—	302.3	UMCs	[161]
**CPL‐1‐NH_2_**, 3D	3.8 × 4.4	1.84	0.21	50/32.4	119	—	103	H‐bonding	[162]
**Ni(4‐DPDS)_2_CrO_4_**, 3D	5.2 × 5.2	2.99	1.79	75.4/37	67.7	He flow at 323 K	317	H‐bonding, C‐H···π interactions	[163]
**ZNU‐1**, 3D	4.4	3.4	1.7	54/44	56.6	—	532	H‐bonding	[164]
**ATC‐Cu**, 3D	4.43 × 5.39	5.0	4.02	79.1/–	53.6	—	600	UMCs, van der Waals interactions	[165]
**ATC‐Cu (MOF‐11)**, 3D	4.4 × 5.4	5.01	4.02	79.1/–	53.6	—	600	UMCs, van der Waals interactions	[165]
**ZJU‐74a**, 3D	3.6 × 3.8	3.83	3.08	44.5/30	36.5	He flow at 373 K	694	H‐bonding, UMCs	[166]
**SNNU‐277**, 3D	5.5 × 5.5	3.64	1.2	42.6/30.5	32.8	—	459	C‐H···π, electrostatics	[167]
**NKMOF‐1‐Ni**, 3D	5.8 × 5.8	2.7	2.3	60.3/40.9	30	—	382	H‐bonding, π···π interactions	[168]
**[C*o*_3_(OH)_2_(HCOO)_2_(CPT)_2_]**, 3D	6.64 × 7.44	5.07	1.49	24.8/20.1	28	—	778	H‐bonding, electrostatics	[169]
**SIFSIX‐21‐Ni**, 3D	3.2 × 3.6	4.05	1.27	40.6/16.1	27.7	N_2_ flow at 333 K	871	Electrostatics, H‐bonding	[81]
**CPL‐1**, 2D	4.0 × 6.0	1.9	0.07	42.5/31.9	26^c^	—	571	H‐bonding	[60]
**ZJU‐196**, 3D	5.1 × 5.1	3.7	0.4	39.2/–	25^d^	—	—	Flexibility, pore size	[170]
**MOF‐OH**, 3D	4	3.04	1.20	17.5/–	25	—	150	Van der Waals interactions	[171]
**FeNi‐M′MOF**, 3D	4.15 × 4.27; 3.94 × 4.58	4.29	2.72	27/24.5	24	—	383	UMCs, π···π stacking interactions	[172]
**SNNU‐98‐Mn**, 3D	5.2^b^	19.88	12.09	60.2/29.2	22.7	—	720.9	Electrostatics	[173]
**[Ni_3_(HCOO)_6_]_n_**, 3D	4.3 × 4.3	2.4	1.6	40.9/24.5	22	Vacuum at 403 K	289	Pore size, H‐bonding	[174]
**Ni(4‐DPDS)_2_WO_4_**, 3D	5.0 × 5.0	2.05	1.43	77.2/40.5	20.9	He flow at 323 K	188	H‐bonding, C‐H···π interactions	[163]
**DICRO‐4‐Ni‐i**, 3D	6.2 × 6.6	1.9	1.0	37.7/33.9	18.2^d^	—	398	H‐bonding	[175]
**SNNU‐98‐Zn**, 3D	4.2^b^	14.64	7.90	41.3/35.9	17.4	—	398.2	Electrostatics	[173]
**TCuCl**, 3D	3.69 × 3.69	3.0	2.0	41/30.1	16.9	N_2_ flow at 333 K	167	Halogen···HC interactions	[176]
**Ni(4‐DPDS)_2_MoO_4_**, 3D	5.2 × 5.2	2.77	1.99	47.8/39.8	14.5	He flow at 323 K	225	H‐bonding, C‐H···π interactions	[163]
**SNNU‐98‐Co**, 3D	4.8^b^	13.79	8.05	53.5/34.7	14.4	—	328.3	Electrostatics	[173]
**JLU‐MOF103**, 3D	3.4 × 2.6, 4.2 × 2.6	4.3	1.9	31.7/25.8	14.0	—	697	H‐bonding	[177]
**Pacs‐CoMOF‐2a**	5.8^b^ × 6.6^b^	5.40	2.81	34.2/–	13	—	196	UMCs	[178]
**BSF‐3‐Co**, 3D	6.79, 6.22, 6.33	3.85	2.41	42.7/22.4	12.7	He flow at 298 K	437	Electrostatics, H‐bonding	[179]
**MIL‐100(Fe)**, 3D	5.5 × 8.6	5.3	2.5	65/–	12.5^d^	—	2300	UMCs	[180]
**ZJU‐40a**, 3D	10.2, 9.6 × 22.3	9.64	3.34	34.5/–	11.5	—	2858	H‐bonding, pore size	[181]
**Co‐MOF**, 3D	—	6.47	2.68	33/27	11	—	973	H‐bonding	[182]
**CAU‐10‐NH_2_**, 3D	3.8, 5.3	3.57	2.08	31.3/24.5	10.8	Vacuum at 333 K	403	Van der Waals interactions	[183]
**GEFSIX‐21‐Cu**, 3D	3.14 × 3.14	5.0	1.8	32.3/22.2	10.8	333 K	1014	H‐bonding	[184]
**ZNU‐9**, 3D	10.3^b^	7.94	4.32	33.1/26.6	10.3	Ar flow at 323 K	1635	H‐bonding, van der Waals interactions	[185]
**TIFSIX‐2‐Cu‐i**, 3D	5.1 × 5.1	4.1	4.3	46/35.8	10^d^	—	685	H‐bonding, electrostatics	[36]
**MIL‐160**, 3D	4.6 × 9.8	8.5	4.02	31.8/–	10	—	1138	H‐bonding	[186]
**JCM‐1**, 3D	12.5 × 3.9	3.3	1.7	36.9/33.4	10	373 K	550	Pore size, H‐bonding, dipolar interactions	[187]
**ZJUT‐2a**, 3D	3.2 × 3.2	3.4	2.2	41.5/35.5	10	—	350	H‐bonding, van der Waals interactions	[188]
**TCuBr**, 3D	3.59 × 3.59	2.8	2.0	36.6/30.7	9.5	N_2_ flow at 333 K	173	Halogen···HC interactions	[176]
**TCuI**, 3D	3.66 × 3.66	2.2	1.6	38.4/26.8	5.3	N_2_ flow at 333 K	250	Halogen···HC interactions	[176]
**UTSA‐74a**, 3D	8.0 × 8.0	4.8	3.2	31/25	9	—	830	UMCs	[65]
**CTGU‐39‐a**, 3D	5.0 × 5.2	2.92	1.99	26.8/19.2	8.4	He flow at 298 K	586.1	H‐bonding, π···π interactions	[189]
**JUN‐4**, 3D	4.8, 9.3^b^	9.82	7.1	26.8/19.7	8.2	He flow at 298 K	1533	UMCs	[190]
**SNNU‐150‐Al**, 3D	8.5^b^	4.33	1.98	29/24.8	7.27	—	—	UMCs	[191]
**FJU‐22a**, 3D	7.1 × 7.1	5.1	5.0	23/18.8	7.1^e^	—	828	H‐bonding, van der Waals interactions	[192]
**ZJU‐60a**, 3D	4.4 × 5.4	6.7	3.3	17.6/15.2	6.7^e^	—	1627	UMCs, pore size	[193]
**NTU‐55**, 3D	10.4^b^	6.05	3.13	25.3/22	6.6^e^	He flow at 333 K	2300	UMCs	[194]
**UTSA‐83a**, 2D	3.5 × 6.6	0.53	0.17	24.4/16.6	6.2	—	70^f^	H‐bonding	[195]
**MUF‐17**, 3D	4.7 × 4.8	2.7	2.2	49.5/33.8	6	Vacuum at 333 K	247	H‐bonding, repulsive electrostatics	[196]
**CPM‐107op**, 3D	—	4.35	1.55	37/24	5.7	Vacuum	319	Flexibility	[197]
**ZJUN‐13**, 3D	6.8^b^, 11.8^b^	5.28	3.92	33.5/22.5	5.64	He flow at 333 K	1352	H‐bonding	[198]
**PCP‐33**, 3D	11 × 20	5.4	2.6	27.5/2.62	5.6^d^	—	1248	UMCs	[199]
**NUM‐20**, 3D	8	1.74	0.79	38.83/37.14	5.4	—	319.17	Electrostatics, C‐H···π interactions	[200]
**FJU‐6‐TATB**, 3D	9.2‐17.1	4.9	4.5	29/26	5.3‐3.1	Vacuum at 353 K	1306	H‐bonding	[201]
**HKUST‐1‐UF**, 3D	—	7.82	4.84	27.7/23.5	5.1	He flow at 423 K	1877	H‐bonding, UMCs	[202]
**UPC‐110**, 3D	6^b^	3.27	1.08	24.6/14	5.1	—	250	UMCs	[203]
**JXNU‐5**, 3D	4.6^b^, 6.7^b^	2.5	1.55	32.9/25.2	5	—	406	H‐bonding, electrostatics	[204]
**HKUST‐1‐UF‐GLY_0.3_**, 3D	—	8.86	5.45	30.4/19.1	4.8	He flow at 423 K	2033	H‐bonding, UMCs	[202]
**Ag NP@Fe_2_O_3_@** **Zn‐MOF‐74**, 3D	7‐10^b^	6.7	5.13	—	4.73	—	936	π‐complexation, plasmon‐driven photothermal effect	[205]
**CAU‐10‐CH_3_**, 3D	5.8 × 5.8	3.5	1.41	25.18/23.4	4.6	298 K	312	H‐bonding, C‐H···π interactions	[206]
**SNNU‐45**, 3D	4.5	5.98	4.33	40/27	4.5	He flow at 298 K	1006	UMCs	[207]
**JLU‐MOF102**, 3D	5.5 × 4.4, 6.8 × 3.3	5.6	3.3	23.3/20.6	4.5	—	1236	H‐bonding	[177]
**UTSA‐220**, 3D	4.5‐5.5, 3.1‐4.8	3.40	3.38	29/27	4.4	He flow at 298 K	577	H‐bonding	[208]
**FJU‐89a**, 3D	12 × 8	4.53	2.73	31/27.8	4.3	—	774	—	[209]
**FJU‐90a**, 3D	5.4 × 5.1	8.0	4.6	25.1/20.7	4.3	Vacuum	1572	H‐bonding, pore size	[210]
**Cu_2_(ade)_2_(PA)_2_**, 3D	2 × 6	2.19	1.5	26.8/23.6	4.2	—	401	UMCs	[211]
**FJU‐114**, 3D	9.2	3.44	1.74	33/29.1	4.2	—	891	π···π interactions	[212]
**TFT‐COF**, 2D	7.5 × 7.5	1.76	1.39	76.8/25.5	4.18	—	689	H‐bonding	[213]
**UPC‐120(Th)**, 3D	8.6 × 8.6, 8 × 8	1.89	1.54	24.13/23.29	4.11	He flow at 298 K	450	H‐bonding	[214]
**ZJU‐199a**, 3D	5‐7.5^b^	5.71	2.78	38.5/29.0	4	—	987	Pore size, UMCs	[215]
**Hex‐Zn‐MOF 1a**, 3D	8.6^b^, 9.8^b^	3.18	2.21	39/27	4	—	770.3	H‐bonding, electrostatics	[216]
**CAU‐10‐H**, 3D	4.7 × 4.7	4.01	2.68	32.8/21.4	4	He flow at 298 K	627	C−H···π, dipole–dipole interactions	[217]
**CAU‐10‐H**, 3D	6.8 × 7.5	4.01	2.62	28/25	4.0	298 K	627	H‐bonding, C‐H···π interactions	[206]
**mot‐Cu(Br‐BDC)**, 3D	4.2 × 4.7, 12 × 24.1	1.53	1.08	26.1/25.6	3.9	He flow at 298 K	303	UMCs, pore size	[218]
**NBU‐3‐Mn/Fe**, 3D	—	3.03	1.61	29/41	3.9	—	551	UMCs	[219]
**UPC‐120(Zr)**, 3D	8.6 × 8.6, 8 × 8	2.14	1.53	25.8/14.03	3.71	He flow at 298 K	520.7	H‐bonding	[214]
**Cu‐CPAH**, 3D	6‐9^b^	5.88	3.93	35.4/31.5	3.6	—	880	H‐bonding, UMCs	[220]
**FJI‐H36**, 3D	12.9 × 12.9, 8.4 × 10.2	6.46	4.2	36.1/29.2	3.5	—	899	H‐bonding, UMCs	[221]
**UTSA‐68a**, 3D	6.5 × 6.5; 7.5 × 9.5	3.13	1.77	25.8/–	3.4	—	1954	UMCs	[222]
**CTGU‐40‐a**, 3D	5 × 5	3.31	2.21	27.8/23.1	3.3	He flow at 298 K	758.4	H‐bonding, π···π interactions	[189]
**TpPa‐F**, 2D	11.9	5.22	2.52	38.1/28.2	3.3	—	1048	H‐bonding	[223]
**SNNU‐98‐Ni**, 3D	4.8^b^	13.17	9.08	39.3/38.7	3.3	—	473.5	Electrostatics	[173]
**UPC‐200(Al)‐F‐BIM**, 3D	7 × 11	6.2	2.5	20.5/13.5	3.15	—	2212.8	Pore size	[224]
**JNU‐1**, 3D	16.3 × 6.6	2.7	2.2	13/–	3	—	818	UMCs, dipole interactions	[160]
**QDU‐MOF‐1**, 3D	7.76	6.3	—	72.52/39.89	2.95	—	1465	H‐bonding	[225]
**LIFM‐210**, 3D	5‐7.25^b^	2.96	2.04	28.3/24.6	2.81	—	607	Cation···π interactions	[226]
**JXNU‐18**, 3D	5.8‐8.5^b^	2.46	1.41	37.4/22.4	2.81	—	622	H‐bonding, C‐H···π interactions	[227]
**Cu‐tztp MOF 1a**, 3D	5.4‐8.6^b^	5.02	3.35	38.3/26.2	2.7	—	798.9	H‐bonding, C‐H···π interactions	[228]
**TpPa‐H**, 2D	12.4	4.87	3.3	39. 9/–	2.7	—	1270	H‐bonding	[223]
**NKM‐123**, 3D	3.8 × 3.8	1.24	0.69	29.3/25.2	2.6	—	217.39	C‐H···π interactions, H‐bonding	[229]
**FJU‐129**, 3D	6.6, 10^b^	3.14	1.33	24.78/24.76	2.5	—	1726	Electrostatics, C‐H···π interactions	[230]
**Zn‐MOF‐74**, 3D	11 × 11	5.5	5.4	22.1/25	2	—	1360	UMCs	[65]
**ZJU‐30a**, 3D	4.0 × 4.0; 5.6 × 5.6	2.31	1.87	31.3/–	1.7	—	228	UMC, pore size	[222]
**Cu_2_(pzdc)_2_(pyz)**, 3D	4 × 6	2.0	—	42.5/31.9	—	—	—	H‐bonding	[60]

^a^ IAST selectivity at 1 bar for 1:1 (v/v) C_2_H_2_/CO_2_.

^b^ Determined from Horvath–Kawazoe method applied on N_2_ isotherm at 77 K.

^c^ Uptake ratio at 0.01 bar for 270 K measurements.

^d^ C_2_H_2_/CO_2_ uptake ratio at 0.5 bar.

^e^ IAST selectivity at 0.15 bar for 1:1 (v/v) C_2_H_2_/CO_2_.

^f^ Determined from CO_2_ isotherm at 195 K. S_BET_ = Brunauer–Emmett–Teller (BET) theory‐based surface areas from N_2_ isotherm recorded at 77 K, unless otherwise mentioned.

^g^ Pore size calculated by Non‐Linear Density Functional Theory (NLDFT) method based on 87 K Ar isotherms.

**TABLE 6:** C_2_H_2_/C_2_H_4_ binary separations. The following parameters are listed for comparison: pore size; BET surface area (S_BET_); single‐component gas uptakes; adsorption enthalpies (*Q*_st_); adsorption selectivity; regeneration temperature; and attributed mechanisms. Reticular sorbents are listed in decreasing order of binary selectivity for C_2_H_2_ selective sorbents (above) and C_2_H_4_ selective sorbents (below).

Adsorbent, network dimensionality (nD)	Pore size (Å)	C_2_H_2_ uptake at 1 bar (mmol/g)	C_2_H_4_ uptake at 1 bar (mmol/g)	*Q*_st_ (kJ/mol)	Selectivity^a^	Regeneration temperature	S_BET_ (m^2^/g)	Mechanism	Refs.
**UTSA‐300a** ^e^, 2D	2.4 × 3.3	3.1	0.04	57.6/–	∼10^4^ ^f^	298 K	311	π···π interactions, H‐bonding	[159]
**NCU‐100a** ^e^ (**UTSA‐300‐Cu**), 2D	4.3 × 3.6	4.57	0.32	60.5/–	7291.3^f^	He flow at 298 K	358	Flexibility, H‐bonding, Pore size	[231]
**bnn‐1‐Ca‐H_2_O** ^e^, 3D	3.4 × 3.4	2.2	0.16	–/–	6966.4^f^	Vacuum at 298 K	210	Molecular sieving	[232]
**SIFSIX‐14‐Cu‐i** ^e^, 3D	3.4 × 3.4	1.8	0.6	40/37	6320^f^	—	612	Molecular sieving, H‐bonding	[39]
**UTSA‐200a**, 3D	3.4 × 3.4	3.65	0.63	—	6320	—	612	Molecular sieving, H‐bonding	[39]
**ZJU‐300a**, 3D	4.1^d^	5.37	2.39	61.1/39.7	1672	—	796	H‐bonding, supramolecular interactions	[233]
**NKMOF‐1‐Ni**, 3D	5.8 × 5.8	2.72	2.14	60.3/44.9	1272.6^c^, 30^b^	—	382	H‐bonding, π···π interactions	[168]
**GeFSIX‐14‐Cu‐i** ^e^ (**ZU‐33**), 3D	3.0 × 3.0	4.1	0.76	43.6/–	1100^f^	He flow at 298 K	424	Size sieving, van der Waals interactions, H‐bonding	[234]
**FJI‐W88**, 3D	4.3 × 2.9	4.36	2.31	31.5/29.3	698	Ar flow at 353 K	487	Van der Waals interactions	[235]
**TIFSIX‐14‐Cu‐i**, 3D	3.6 × 3.6	3.78	1.41	54/40	229	—	425	Pore size, C‐H···π interactions	[236]
**TIFSIX‐2‐Cu‐i**, 3D	5.1 × 5.1	3.9	2.1	46/–	55^b^, 212.2^b^	—	685	H‐bonding	[36]
**GeFSIX‐2‐Cu‐i**, 3D	4.5 × 4.5	3.9	2.2	42.6/32	67	He flow at 298 K	424	Pore size, H‐bonding, Van der Waals interactions	[234]
**sql‐SIFSIX‐bpe‐Zn**, 2D	—	1.79	1.25	67.5/38.4	53.1	Vacuum at 313 K	—	H‐bonding, σ···π and π···π interactions	[237]
**SIFSIX‐2‐Cu‐i**, 3D	5.2 × 5.2	4.02	2.19	41.9/30.7	44.54, 41.01^b^	—	735	Pore size, H‐bonding	[35]
**ZNU‐6**, 3D (278 K)	8.5 × 8.5, 4 × 4	8.06	6.5	37.2/29.0	43.8^c^	Ar flow at 393 K	1330	Electrostatics	[238]
**Ni‐gallate**, 3D	3.5 × 4.9	3.59	2.1	46/–	43.7	He flow at 393 K	424	H‐bonding, pore size	[239]
**NbOFFIVE‐2‐Ni‐i** (**ZU‐62‐Ni**), 3D	3.0 × 3.9	3.0	0.8	43/–	37.2	298 K	404	Pore size	[240]
**M′MOF‐3a**, 3D	3.4 × 4.8	1.9	0.4	27.1/27.3	24.03, 34.17^b^	—	110	Pore size, π···π interactions	[241]
**CPL‐1**, 3D	4.0 × 6.0	2.07	0.31	40.2/36.3	26.75	He flow at 393 K	414	Pore size, H‐bonding	[242]
**Mg‐gallate**, 3D	3.6 × 4.8	4.39	3.03	33/–	20.9	He flow at 393 K	559	Pore size, H‐bonding, Van der Waals interactions	[239]
**UTSA‐100a**, 3D	4.3 × 4.3	4.27	1.66	22/–	10.72, 19.55^b^	353 K	970	Pore size, Van der Waals interactions	[243]
**GeFSIX‐dps‐Cu** ^e^, 2D	1.8 × 2.6; 2.5 × 4.4	4.28	0.16	–/–	19^g^	—	382	Size sieving, H‐bonding	[244]
**UTSA‐60a**, 3D	4.8 × 4.0	3.12	2.05	36/–	16	—	484	UMC, pore size	[245]
**Co‐gallate**	3.7 × 5.0	3.88	3.37	47/–	15	He flow at 393 K	475	Pore size, H‐bonding, Van der Waals interactions	[239]
**ELM‐12**, 2D	4.3 × 3.9	2.56	1.0	25.4/–	14.8	He flow at 373 K	706	Flexibility, H‐bonding	[246]
**APPT‐Cd‐ClO_4_** ^−^, 3D	11 × 11	1.75	0.44	28.5/3.7	14.71	298 K	205	H‐bonding	[247]
**SIFSIX‐1‐Cu**, 3D	8.0 × 8.0	8.5	4.11	30,37/23.5	8.82, 13.72	—	250	Pore size, H‐bonding, Van der Waals interactions	[35]
**SIFSIX‐3‐Zn**, 3D	4.2 × 4.2	3.64	2.24	21, 31/28.8	8.82, 13.72^b^	He flow at 298 K	250	Pore size, H‐bonding	[35]
**MECS‐5**, 3D	4.5 × 5.3, 3.4 × 4.5, 1.8 × 3.9	3.85	1.14	26.1/26	12.6	—	964	Molecular sieving	[248]
**CPL‐2**, 3D	9.0 × 6.0	3.13	1.86	30.8/20.3	12	He flow at 393 K	495	Pore size, H‐bonding	[242]
**Pacs‐CoMOF‐2a**, 3D	5.8 × 6.6	5.4	2.81	34.2/–	11.5	—	196	UMCs	[178]
**NTU‐92**, 3D	3.0 × 3.2	1.47	0.58	35.5/24.5	10	Vacuum at 353 K	347	H‐bonding, C‐H···π interactions	[249]
**UTSA‐220**, 3D	4.5 × 4.1; 2.1 × 5.0	3.4	2.53	29/24	10, 8.8^b^	He flow at 298 K	577	Pore size, H‐bonding	[208]
**Na@COF‐ECUT‐1**, 2D	8	4.00	2.20	19.21	9.41	—	149	UMCs	[250]
**MUF‐17**, 3D	3.1 × 3.5; 4.7 × 4.8	3.02	2.16	49.5/31.1	8.73	He flow at 403 K	247	Pore size, electrostatics	[196]
**JCM‐1**, 3D	3.9 × 12.5	3.34	1.56	36.9/34.2	8.1	He flow at 373 K	550	H‐bonding, pore size	[187]
**NTU‐91**, 3D	3.7 × 10.9	1.32	0.63	32.2/22.2	8	Vacuum at 353 K	287	—	[249]
**Sr‐TCPE**, 3D	5.2 × 4.3; 5.9 × 5.2	1.52	0.9	29/–	8	—	—	Pore size	[251]
**ZJU‐198a**, 3D	3.6 × 4.1; 2.1 × 5.0	3.25	2.95	26.1/37.4	7.2	He flow at 298 K	343.1	Pore size	[252]
**NTU‐90**, 3D	4 × 11.4	1.25	0.71	32/22	6.6	Vacuum at 353 K	321	—	[249]
**COF‐ECUT‐1**, 2D	12	2.47	1.28	7.68	6.33 (1:99)	—	306	H‐bonding	[250]
**SIFSIX‐2‐Cu**, 3D	10.5 × 10.5	5.38	2.02	26.3/20.8	6, 4.95	—	3140	H‐bonding	[35]
**UTSA‐67a**, 3D	3.3 × 3.3	5.13	2.81	32/	6	—	1136.7	Pore size	[253]
**CPL‐5**, 3D	11.0 × 6.0	3.01	1.84	31.3/19.1	6	He flow at 393 K	523	Pore size, H‐bonding	[242]
**SIFSIX‐3‐Ni**, 3D	4.2 × 4.2	3.3	1.75	30.5/30.3	5.03, 5.98	—	223	Pore size	[35]
**NBU‐1**, 3D	3.8^d^	3.64	2.07	38.3/37.9	5.9^b^	Vacuum, 323 K	368	UMCs	[254]
**Ni‐DCPTP**, 3D	6.7^d^, 10^d^	6.54	4.48	38.9/–	5.5	He flow at 298 K	857	H‐bonding	[255]
**Al‐PyDC**, 3D	5.8^d^	8.24	3.44	35.3/27.8	4.3	N_2_ flow at 373 K	1134	H‐bonding, C‐H···π interactions	[256]
**PAF‐120**, 2D	24	2.27	1.22	37.5/343.3	4.1	—	801	Electrostatics	[257]
**PAF‐110**, 2D	17, 25	2.23	1.29	38.4/22.6	3.9	—	910	H‐bonding	[258]
**HUST‐6**, 3D	—	3.49	2.38	31.1/30.2	3.8	—	645.3	UMCs	[259]
**CTF‐PO71**, 2D	7, 14	3.3	2.3	28‐26/22‐19	2.8‐1.8	—	1401	Electrostatics	[260]
**Fe‐BDC‐TPT‐BF_4_**, 3D		9.4	5.92	—	2.7	—	1307.8	Electrostatics	[261]
**Mg‐MOF‐74**, 3D	11 × 11	8.37	7.45	41/–	2.18	—	927	UMCs	[262]
**NOTT‐300**, 3D	6.5 × 6.5	6.34	4.28	32/16	2.17, 2.3^b^	—	1370	H‐bonding, π···π, dipole interactions	[263]
**Fe‐MOF‐74**, 3D	11 × 11	6.8	6.1	46/–	2.08, 2.1^b^	—	1350	UMCs	[66]
**Co‐MOF‐74**, 3D	11 × 11	8.17	7.02	45/	1.7	—	1018	UMCs	[262]
**RPM3‐Zn**, 3D	—	2.14	0.89	—	—	—	328	Flexibility	[264]
**BUT‐11**, 3D	11^d^, 12.2^d^	7.14	3.44	20/–	—	298	1233	Pore size, H‐bonding	[265]

^a^ IAST selectivity at 1 bar for 1:99 (v/v) C_2_H_2_/C_2_H_4_.

^b^ IAST selectivity at 1 bar for 1:1 (v/v) C_2_H_2_/C_2_H_4_.

^c^ IAST selectivity at lowest C_2_H_2_ loading for 1:99 (v/v) C_2_H_2_/C_2_H_4_.

^d^ Determined from Horvath–Kawazoe method or non‐local density functional theory applied on N_2_ isotherm at 77 K.

^e^ IAST selectivities are qualitative, because of molecular sieving.

^f^ Not applicable because of virial fits not conforming to stepped isotherms obtained at 298 K and 273 K.

^g^ Uptake ratio at C_2_H_2_/C_2_H_4_ (0.1/0.9).

**TABLE 7:** CO_2_/C_2_H_2_ binary separations. The following parameters are listed for comparison: pore size; BET surface area (S_BET_); single‐component gas uptakes; adsorption enthalpies (*Q*_st_); adsorption selectivity; regeneration temperature; and attributed mechanisms. Reticular sorbents are listed in decreasing order of binary selectivity for CO_2_ selective sorbents (above) and C_2_H_2_ selective sorbents (below).

Adsorbent, network dimensionality (nD)	Pore size (Å)	CO_2_ uptake at 1 bar (mmol/g)	C_2_H_2_ uptake at 1 bar (mmol/g)	*Q*_st_ (kJ/mol)	S^a^	Regeneration temperature	S_BET_ (m^2^/g)	Mechanism	Refs.
**ALF**, 3D	4.1 × 5.3	3.85	0.15	51/39	6.5 × 10^5^	—	—	H‐bonding	[266]
**Cu‐F‐pymo**, 3D	3.4 × 3.4, 3.2 × 3.2	1.19	0.10	28.8/–	>10^5^	He flow at 298 K	93	Molecular sieving	[267]
**MUF‐4**, 3D	2.2 × 2.2	3.17	—	24.4/–	3360^e^	He flow at 298 K	1094	Electrostatics, pore size	[268]
**Zn‐ox‐mtz**, 3D	5.3 × 3.5, 5.2 × 5.7	3.07	0.25	43.02/–	1064.9	—	466	H‐bonding	[269]
**PMOF‐1**, 3D	5.5 × 6.8	2.38	0.33	—	694	—	—	H‐bonding	[270]
**MUF‐16**, 3D	3.6 × 7.6	2.13	0.18	32.3/25.8	510	—	214	H‐bonding	[129]
**ZnAtzCO_3_**, 3D	2.9 × 5.1, 3.5 × 5.1	0.66	2.80	32.6/22.4	151	—	455.6	Electrostatics	[271]
**Cd‐NP**, 3D	3.2 × 3.2, 6.1 × 4.5 × 4.5	2.59	0.43	27.7/–	85	—	305^c^	Electrostatics	[272]
**MUF‐16 (Ni)**, 3D	3.6 × 7.6	2.13	0.34	37.3/–	46	Vacuum	204	H‐bonding	[129]
**Ce(IV)‐MIL‐140‐4F**, 3D	3.4‐4.3	4.92	1.85	39.5/27.4	>40	He flow at 298 K	360	F···C = O	[273]
**MUF‐16 (Mn)**, 3D	3.6 × 7.6	2.25	0.43	36.6/–	31	Vacuum	205	H‐bonding	[129]
**JNU‐5‐Me**, 3D	2.5 × 2.5	4.7	0.25	37.4/1.7	17.7	Vacuum at 298 K	646	H‐bonding, van der Waals interactions	[274]
**Tm(OH‐bdc)**, 3D	6.3 × 9.3; 6.3 × 10.6	5.8	2.0	45.2/17.8	17.5^b^	—	923	H‐bonding	[275]
**CD‐MOF‐2**, 3D	4.2 × 4.2; 7.8 × 7.8; 17 × 17	2.7	2.0	67.2/25.8	16.6^b^	He flow at 298 K	922	H‐bonding	[276]
**Mg‐CUK‐1** (233 K), 3D	8.1 × 10.6	6.43	3.97	—	12.1	He flow at 298 K	500‐600	H‐bonding, C‐H···π interactions	[277]
**Co‐CUK‐1** (233 K), 3D	8.1 × 10.6	7.59	5.31	43.5/33.3	9.5	He flow at 298 K	500‐600	H‐bonding, C‐H···π interactions	[277]
**Mn(bdc)(dpe)**, 3D	3.3 × 3.5	2.1	0.3	29/27.6	8.8	—	535^c^	Flexibility, C‐H···π interactions	[278]
**SIFSIX‐3‐Ni**, 3D	4.2 × 4.2	2.7	3.3	50.9/36.7	7.7	—	368	H‐bonding, electrostatics	[36]
**CD‐MOF‐1**, 3D	4.2 × 4.2; 7.8 × 7.8; 17 × 17	2.9	2.2	41.0/17.6	6.6^b^	He flow at 298 K	1094	H‐bonding	[276]
**PCP‐NH_2_‐ipa**, 3D	4.4 × 4.8	3.21	1.94	36.6/26.8	6.4	Vacuum	—	—	[279]
**Bi‐BTC**, 3D	4^b^	1.76	2.4	32.19/35.37	5.14	—	92.51	—	[280]
**PCP‐NH_2_‐bdc**, 3D	4.4 × 5.3	3.03	1.91	34.57/25.6	4.4	Vacuum	—	Electrostatics	[279]
**Y‐ bptc**, 3D	9.46 × 9.46, 4.2 × 4.2	2.48	1.17	31.5/–	4.1	—	—	Pore size, H‐bonding, kinetic	[281]
**FJUT‐1**, 3D	5.8, 6.9^b^	5.95	4.84	31.88/25.97	4.06	—	1240	H‐bonding, C‐H···π interactions	[282]
**Zn(atz)(BDC‐Cl_4_)_0.5_**, 3D	4.7 × 6.8	1.54	0.8	32.7/25.4	2.4	—	—	Electrostatics	[283]
**Co(HL)**, 3D	6.2^b^	10.69	6.25	—	1.7	—	773	Flexibility	[284]
**Eu‐MOF**, 3D	6.2 × 7.4	1.04	0.93	31.4/27.3	1.1	—	143	—	[285]
**NbOFFIVE‐1‐Ni**, 3D	3.0 × 3.9	2.2	2.4	54.6/34	0.9	—	280	—	[286]
**AlFFIVE‐1‐Ni**, 3D	3.2 × 3.2	2.75	4.6	47/38	0.6	—	258	—	[286]

^a^ IAST selectivity at 1 bar for CO_2_/C_2_H_2_ (1:1) mixture.

^b^ IAST selectivity at 1 bar for CO_2_/C_2_H_2_ (1:2) mixture.

^c^ Surface area calculated from CO_2_ at 195 K or 273 K isotherm.

^d^ Kinetic selectivity at ambient temperature.

^e^ Kinetic selectivity is calculated from the ratio of the diffusion time constants for two gases.

**TABLE 8:** C_2_H_4_/C_2_H_6_ binary separations. The following parameters are listed for comparison: pore size; BET surface area (S_BET_); single‐component gas uptakes; adsorption enthalpies (*Q*_st_); adsorption selectivity; regeneration temperature; and attributed mechanisms. Reticular sorbents are listed in decreasing order of binary selectivity for C_2_H_4_ selective sorbents (above) and C_2_H_6_ selective sorbents (below).

Adsorbent, network dimensionality (nD)	Pore size (Å)	C_2_H_4_ uptake at 1 bar (mmol/g)	C_2_H_6_ uptake at 1 bar (mmol/g)	*Q*_st_ (kJ/mol)	Selectivity^a^	Regeneration temperature	S_BET_ (m^2^/g)	Mechanism	Refs.
**UTSA‐280**, 3D	3.2 × 4.5; 3.8 × 3.8	2.5	0.098	34.1/–	10^4^ ^b^	He flow at 353 K	331	Molecular sieving, H‐bonding, π···π stacking, van der Waals interactions	[287]
**NUS‐6(Hf)‐Ag**, 3D	10, 17	2.02	1.35	56.5/29.8	106.3, 6^c^	He flow at 298 K	1027	Cation···π	[288]
**ITQ‐55**, 3D	2.33 × 5.71, 3.08 × 5.71	1.28	0.76	—	100	—	—	Flexibility, pore shape	[289]
**Cu^I^@UiO‐66‐(COOH)_2_**, 3D	4.1^d^	1.86	0.85	48.5/26.7	80.8	He flow at 413 K	320	Molecular sieving, π‐complexation	[290]
**Co‐gallate**, 3D	3.69 × 4.95	3.37	0.31	44/–	52	—	475	Molecular sieving	[291]
**Mg‐gallate**, 3D	3.56 × 4.84	3.03	0.26	39/–	37.3	—	559	Molecular sieving	[291]
**Ni‐gallate**, 3D	3.47 × 4.85	1.97	0.28	32/–	16.8	—	424	Molecular sieving	[291]
**NOTT‐300**, 3D	6.5 × 6.5	4.28	0.85	16/11	47.8	He flow at 373 K	1370	H‐bonding, π···π stacking, intermolecular dipole interactions	[263]
**ZnAtzPO_4_@CMC**, 3D (273 K)	5 × 5	0.64	1.01	—	34.7	—	—	Electrostatics	[292]
**PAF‐1‐SO_3_Ag**, 3D	8.0	4.06	2.23	106/–	27	—	783	π‐complexations	[293]
**10 wt% Ag/CPL‐2**, 3D	7‐11^d^	0.9	0.15	—	26.1	He flow at 298 K	12	Electrostatics	[294]
**Fe_2_(*m*‐dobdc)**, 3D	12	7.0	6.0	55/–	25	He flow at 298 K	1295	UMCs	[295]
**Fe_2_(dobdc)**, 3D (318K)	11	6.02	—	45/25	13‐18	—	1350	UMCs	[66]
**(Cr)‐MIL‐101‐SO_3_Ag**, 3D^e^	–, 15–18^d^	3.26, 4.32	1.47, 1.22	63/16, 120/–	9.7, 16	—	1374, 1253	UMCs, π‐complexation	[296, 297]
**NaETS‐10**, 3D	8.0	1.7	1.3	—	14	—	289	UMCs	[298]
**Fe‐MOF‐74**, 3D	11	6.28	5.10	47.5/25	13.6	—	1350	UMCs	[66]
**Zeolite 13X**, 3D	2.3	2.8	2.2	—	13.4	873 K	406	π‐cation interactions	[299]
**ZnAtzPO_4_**, 3D	3.82 × 4.94	1.92	1.04	29.98/	12.4	He flow at 298 K	470	Kinetic	[300]
**1.6AgM‐DS**, 3D	—	3.37	0.94	59.2/–	9.5	378 K	846	π‐complexation	[301]
**MIL‐101‐SO3Ag@CMC**, 3D	—	2.2	1.0	49.5/18.0	7.0	N_2_ flow at 373 K	1320	UMCs	[302]
**Cu(OPTz)**, 3D	3 × 3	2.3	0.4	27.7/–	6	—	—	Flexibility	[303]
**GT‐18**, 3D	3 × 7	0.6	0.2	—	6.8	—	—	Pore shape, diffusivities	[304]
**Co‐MOF‐74**, 3D	11	6.21	5.25	43.6/–	5.82	—	1341	UMCs	[305]
**Mg‐MOF‐74**, 3D	11	7.4	6.4	42/	5.6	—	927	UMCs	[262]
**Ca(squarate)**, 3D	6.9 × 6.9	2.3	1.3	33.9/28.4	4.9	He flow at 353 K	224	π···π interactions, H‐bonding	[306]
**Zeolite 5A**, 3D	5.0	2.45	1.72	37/33	4.5	He flow at 553 K	457‐600	Cation···π interactions	[307]
**NUS‐36**, 3D	—	1.5	1.0	44/44	4.1	He flow at 298 K	79.1	Pore size, van der Waals interactions	[308]
**HKUST‐1**, 3D	10, 14	7.20	6.03	39/31	3.6	—	1500‐2100	UMCs	[262]
**Co(VTTF)**, 2D (283K)	14.6 × 9.8	4.9	0.98	—	2.03	—	—	Flexibility	[309]
**Cu^I^‐MFU‐4 L**, 3D	—	3.79	2.78	79.9/–	—	He flow at 413 K	2961	UMCs	[310]
**ZNU‐10**, 3D	8.59	2.13	1.57	26.4/23.2	1.68	Vacuum	1171	H‐bonding, van der Waals interactions	[311]
**UiO‐66‐ADC**, 3D	4.4	1.7	1.6	36/36	0.55	—	556	—	[308]
**ZU‐901**, 3D	3.4 × 4.2	1.55	0.26	39.28/24.2	—	Vacuum	229.86^f^	H‐bonding	[312]

^a^ IAST selectivity at 1 bar for 1:1 (v/v) C_2_H_4_/C_2_H_6_.

^b^ IAST selectivities are qualitative, because of molecular sieving.

^c^ IAST selectivity at 0.01 bar for 1:1 (v/v) C_2_H_4_/C_2_H_6_.

^d^ Determined from Horvath–Kawazoe method applied on the N_2_ isotherm at 77 K.

^e^ Two consecutive reports on this sorbent document distinct values that are included using a comma between them. *S*_BET_ = Brunauer–Emmett–Teller (BET) theory‐based surface areas from N_2_ isotherm recorded at 77 K, unless otherwise mentioned.

^f^ Langmuir surface areas.

**TABLE 9:** C_2_H_6_/C_2_H_4_ binary separations. The following parameters are listed for comparison: pore size; BET surface area (S_BET_); single‐component gas uptakes; adsorption enthalpies (*Q*_st_); adsorption selectivity; regeneration temperature; and attributed mechanisms. Reticular sorbents are listed in decreasing order of binary selectivity for C_2_H_6_ selective sorbents (above) and C_2_H_4_ selective sorbents (below).

Adsorbent, network dimensionality (nD)	Pore size (Å)	C_2_H_6_ uptake at 1 bar (mmol/g)	C_2_H_4_ uptake at 1 bar (mmol/g)	*Q*_st_ (kJ/mol)	S (binary)^b^	Regeneration temperature	S_BET_ (m^2^/g)	Mechanism	Refs.
**NIIC‐20‐Bu**, 3D	3.5, 11.6	2.5	1.4	24.1/22.9	15.4	—	1440	H‐bonding, C‐H···π interactions	[313]
**MAF‐49**, 3D	3.3 × 3.0	1.7	1.7	60/48	9	—	—	H‐bonding, electrostatics	[314]
**NIIC‐20‐GI**, 3D	3.5, 12	2.1	1.7	31.5/30.3	8.7	—	1600	—	[313]
**NIIC‐20‐Pe**, 3D	3.6, 11.5	2.2	1.6	25.4/24.3	8.4	—	1286	—	[313]
**Fe_2_(O_2_)dobdc**, 3D	14 × 14	3.32	2.54	66.8/36.5	4.4	298 K	1073	H‐bonding, Van der Waals interactions	[315]
**NIIC‐20‐Pr**, 3D	3.6, 11.8	2.4	1.9	34.2/32.4	4.0	—	2260	—	[313]
**X‐dia‐1‐Ni_0.89_Co_0.11_**, 3D (273 K)	—	4.96	0.54	—	5.47	—	—	Flexibility	[316]
**UTSA‐30**, 3D	3.2 × 3.2	2.1	2.1	30/30	3.8	—	592	Pore size, shape	[111]
**X‐dia‐1‐Ni**, 3D (273 K)	—	5.54	1.58	23.4/20.5	3.51	—	—	Flexibility	[316]
**NIIC‐20‐Et**, 3D	6 × 3.5, 3.5 × 3.5	2.4	1.8	29.8/29.7	3.5	—	2700	—	[313]
**Cu(Qc)_2_**, 2D	3.3 × 3.3	1.85	0.78	30/25.4	3.4	—	240	Flexibility, C‐H···π interactions	[317]
**CuIn(3‐ain)_4_**, 3D	6.7	2.71	2.68	32.11/32.06	3.31	—	472	H‐bonding, C‐H···π interactions	[318]
**ZIF‐62**, 3D	5.1	1.65	—	—	3.3	—	994	—	[319]
**F‐MOF‐2**, 3D	6.5	1.2^c^	1.0^c^	37/–	3.3^c^	—	390	H‐bonding	[320]
**NPU‐3**, 3D	12.2^a^	3.36	2.22	18.71/17.79	3.21	He flow at 313 K	1834	H‐bonding, C‐H···π interactions	[321]
**SBMOF‐2**, 3D	3.6 × 3.6	2.8	2.7	32.3/29.2	3	—	195	C‐H···π interactions, H‐bonding	[322]
**Co(AIN)_2_**, 3D	4.72 × 4.72	3.17	3.17	34.7/34.1	2.96	He flow at 298 K	450	C‐H···π interactions	[323]
**Zn‐FBA**, 3D	6.0 × 6.0	1.25	1.14	42.8/39.8	2.9	—	297	H‐bonding, van der Waals interactions	[324]
**PCP‐IPA**, 3D	4.7 × 5.6	2.5	2.13	37.73/20	2.80	N_2_ flow at 333 K, 337 K	486.7	H‐bonding, van der Waals interactions	[325]
**ZJU‐121**, 3D	3.7 × 3.7	3.1	3.19	47.1/43	2.74	He flow at 298 K	923	—	[326]
**NKMOF‐8‐Br**, 3D	7.11 × 6.15	4.22	3.67	40.8/33.6	2.65	373 K	352	C‐H···π interactions	[327]
**Al‐MOFM_15_**, 3D	3 × 3, 7.7 × 7.7	2.23	1.29	45/32.8	2.51	298 K	793	H‐bonding, π···π, C‐H···π interactions	[328]
**UiO‐66‐2CF_3_**, 3D	7.34 × 7.34	0.88	0.5	14.5/–	2.5	—	467	Pore size	[329]
**Ni(IN)_2_**, 3D	5.06 × 4.37	3.05	0.89	34.5/33.3	2.44	433 K	520	C‐H···π interactions	[330]
**Zn(ad)(int)**, 3D	8^a^	2.32	2.22	33.2/28.9	2.4	—	617	H‐bonding, C‐H···π interactions	[331]
**Ni(sdba)(dabco)_0.5_**, 3D	6.8 × 7.1, 4.9 × 3.0, 3.6 × 5.0	3.15	2.99	29.77/28.2	2.28	—	668	H‐bonding, C‐H···π interactions	[332]
**Cu‐APC**, 2D	5.1 × 7.2	1.56	1.55	29.2/28.8	2.27	298 K	655.5	Flexibility, H‐bonding, C‐H···π interactions	[333]
**ZIF‐3**, 3D	5, 8	6.0	5.5	28.5/24.2	2.22	—	2997	—	[334]
**ZUL‐C4**, 3D	6.00	1.7	2.9	31.6/24.3	2.21	N_2_ flow at 298 K	1190	Van der Waals, C–H···O dipolar interactions	[335]
**ZIF‐1**, 3D	6.5	1.48	—	—	2.2	—	1502	—	[319]
**ZIF‐20**, 3D	6.5, 15	2.16	—	—	2.2	—	1767	—	[319]
**Zn(BDC)(H_2_BPZ)**, 3D	8.0‐10.2^a^	3.63	3.29	31.8/23.2	2.2	—	906.5	H‐bonding, C‐H···π interactions	[336]
**PCP‐IPA‐NH_2_**, 3D	3.3 × 5.2	1.7	1.9	–/32.4	2.19	—	387	H‐bonding, C‐H···π interactions	[337]
**ZIF‐22**, 3D	7, 14.8	2.3	—	—	2.15	—	1818	—	[319]
**CuTiF_6_‐TPPY**, 3D	5.0 × 8.0	5.32	4.32	34.2/29.6	2.12	He flow at 298 K	685	H‐bonding, Van der Waals interactions	[338]
**Fe_2_(BDP)_3_**, 3D	4.9 × 4.9	2.25	2.11	23.9/19.8	2.1	—	1097	UMCs	[339]
**ZIF‐96**, 3D	17, 8.5	0.82	—	—	2.1	—	1890	—	[319]
**CTF‐DCTC‐500**, 3D	5.7, 7.9, 11.8	3.10	2.34	25.4/23.7	2.08	Vacuum at 298 K	916	Pore size	[340]
**Tb‐MOF‐76(NH_2_)**, 3D	7.2 × 7.2	3.27	2.97	32.8/30.9	2.05	—	—	H‐bonding, C‐H···π interactions	[341]
**dia‐4‐Co**, 3D	7.8 × 9.9	4.6	4.07	24.56/23.37	2.04	Vacuum at 343 K	—	—	[342]
**ZUL‐C3**, 3D	6.16	1.5	2.3	29.5/19.9	2.04	N_2_ flow at 298 K	1200	Van der Waals interactions, C–H···O dipolar interactions	[335]
**ZJU‐30**, 3D	4.0 × 4.0; 5.6 × 5.6	2.1	2.0	29.7/28.1	2	—	228	Pore size, UMCs	[343]
**ZJU‐120a**, 3D	4.4 × 4.4	4.91	3.93	27.6/17	2	He flow at 298 K	1597	C‐H···π interactions	[326]
**Ni(TMBDC)(DABCO)_0.5_**, 3D	5.9	5.45	5.02	39/31	2.0	Vacuum	894	—	[344]
**ZIF‐2**, 3D	5.5, 6.5	2.61	—	—	2.0	—	2746	—	[319]
**ZIF‐68**, 3D	10.5, 8.5	1.6	—	—	2.0	—	1831	—	[319]
**HIAM‐210**, 3D	4.02^a^	2.34	2.12	31.24/22.72	2.0	He flow at 373 K	566	H‐bonding, Van der Waals interactions	[345]
**In‐BH(im)_3_‐BDC**, 3D	6.81 × 6.15	2.67	2.38	27.8/23.7	2.0	—	914	H‐bonding	[346]
**MUF‐15**, 3D	8.5 × 3.5, 7 × 3.8, 3.2 × 1.2	4.69	4.15	28.2/29.2	1.96	He flow at 343 K	1130	C‐H···π, Van der Waals interactions	[347]
**Y‐BTC**, 3D	7.0 × 7.0	3.5	3.1	22/19	1.92	—	933	UMCs	[348]
**COF‐1**, 2D	9	2.4	1.9	22.5/22.2	1.92	He flow at 298 K	819	π···π, C‐H···π interactions	[349]
**PCN‐245**, 3D	10^a^	3.27	2.39	20.5/23.0	1.9	423 K	1743	Van der Waals interactions	[350]
**PCN‐250**, 3D	5.5 × 5.5; 9.6 × 9.6	5.2	4.2	23/21	1.9	—	1470	Pore size, van der Waals interactions	[351]
**C‐PDA‐3**, 3D	—	6.57	5.10	22/21.6	1.9	—	3160	H‐bonding, electrostatics	[352]
**(Hf)DUT‐52**, 3D	7.9^a^, 10^a^	4.02	3.22	25.6/24	1.9	—	1505	C‐H···π interactions	[353]
**MIL‐53(Al)‐FA**, 3D	7^a^	3.7	3.5	34/30.5	1.9	—	1132	Van der Waals interactions	[354]
**Ni‐MOF‐2**, 3D	8.0	4.7	5.9	23.6/21.4	1.9	He flow at 298 K	1501	C‐H···π interactions, van der Waals interactions	[355]
**HIAM‐102**, 3D	4.6 × 4.6	1.97	2.14	32.1/27.9	1.9	He flow at 373 K	191	Van der Waals interactions	[356]
**ZIF‐71**, 3D	17	0.49	—	—	1.9	—	1224	—	[319]
**Al‐PyDC**, 3D	5.8^a^	4.20	3.44	30.1/27.8	1.9	He flow at 373 K	1134	H‐bonding, C‐H···π interactions	[256]
**MOF‐808‐Bzz**, 3D	12, 14.8	2.20	1.43	29.87/26.43	1.9	—	1003	H‐bonding, C‐H···π interactions	[357]
**Zn_2_(TM‐bdc)(dabco)**, 3D	—	5.23	4.86	31.5/30.2	1.89	—	962.5	Van der Waals interactions	[358]
**NKMOF‐14‐PZ**, 3D	8.5 × 8.5	3.4	5.6	21.3/19.2	1.89	—	1561	H‐bonding, C‐H···π interactions	[359]
**NKMOF‐8‐Me**, 3D	6.96 × 6.21	4.82	4.67	38.4/37.6	1.88	373 K	655	C‐H···π interactions	[327]
**Zn(BTFM)(DABCO)_0.5_**, 3D	4.1‐6.6	2.74	2.01	27.2/24.7	1.88	N_2_ flow at 393 K	720	H‐bonding, C‐H···π interactions	[360]
**Eu‐BTC**, 3D	6.0 × 6.0	3.1	2.9	26/21	1.87	—	720	UMCs	[348]
**Y‐TATB**, 3D	7.5, 12	4.33	2.98	22.9/20.6	1.87	Vacuum at 393 K	1562	Van der Waals, C‐H···π interactions	[361]
**FHI‐H11‐Me**, 3D	—	2.59	2.08	38.9/25.9	1.85	—	—	H‐bonding, C‐H···π interactions	[362]
**Zn‐ATA**, 3D	3.5^f^	1.08	1.20	32.5/27.4	1.84^e^	298 K	344^g^	H‐bonding, C‐H···π interactions	[363]
**Cu(BTFM)(DABCO)_0.5_**, 3D	4.2‐5.8	3.02	2.09	25.3/22.7	1.83	N_2_ flow at 393 K	646	—	[360]
**IRMOF‐8**, 3D	11.0 × 11.0	4.1	2.9	54/50	1.8	—	1360	Electrostatics	[364]
**Sm‐BTC**, 3D	—	1.56	1.64	26/23	1.8	—	700	Van der Waals interactions	[348]
**CPM‐80‐Fe**, 3D	—	4.56	4.05	24.1/22.4	1.8	—	863	Van der Waals interactions, C‐H···π interactions	[365]
**CPM‐80‐Co**, 3D	—	4.33	3.86	22.5/22.6	1.8	—	895	Van der Waals interactions, C‐H···π interactions	[365]
**CPM‐80‐Zn**, 3D	—	4.77	4.24	24.6/23.1	1.8	—	995	Van der Waals interactions, C‐H···π interactions	[365]
**CPM‐81‐Co**, 3D	—	5.51	5.07	24.2/24.1	1.8	—	1015	Van der Waals interactions, C‐H···π interactions	[365]
**CPM‐81‐Zn**, 3D	—	4.46	4.13	25.5/22.6	1.8	—	907	Van der Waals interactions, C‐H···π interactions	[365]
**ZIF‐97**, 3D	16	0.83	—	—	1.8	—	1541	—	[319]
**ZIF‐8**, 3D	3.4^a^	2.54	1.5	17.2/16.1	1.8	He flow at 373 K	1844	Kinetic	[366]
**NUM‐7a**, 3D	4.7 × 7.8	2.85	2.62	35.8/30.0	1.76	—	345	Van der Waals, C‐H···π interactions, H‐bonding	[367]
**dia‐4‐Ni**, 3D	7.8 × 9.9	4.46	4.01	24.85/23.59	1.76	Vacuum at 343 K	260	H‐bonding, C‐H···π interactions	[342]
**CPM‐733**, 3D	7.3 × 7.3	7.1	6.4	23.4/22.5	1.75	—	1328.5	Pore size	[72]
**Ca(H_2_tcpb)**, 3D	5.5 × 5.5	2.78	2.67	35.1/26.8	1.75	—	200	—	[368]
**Co_2_V‐bdc‐tpt**, 3D	7.3 × 7.3	6.43	7.19	23.4/22.5	1.75	—	1162	UMCs	[72]
**ZIF‐10**, 3D	8.5	1.15	—	—	1.75	—	3385	—	[319]
**MCOF‐1**, 3D	6.4	3.3	2.8	30.0/27.5	1.73	He flow at 298 K	1045	—	[349]
**Zn(sdba)(dabco)_0.5_**, 3D	6.8 × 7.1, 3.2 × 5.0, 3.8 × 5.1	2.83	2.68	28.96/27.94	1.72	—	224	H‐bonding, C‐H···π interactions	[332]
**SBMOF‐1**, 3D	4.2 × 4.2	1.3	1.3	36.3/35.0	1.7	—	145	C‐H···π interactions, H‐bonding	[322]
**MFM‐300(In)**, 3D	6.8^a^	5.1	4.9	30/28	1.7	—	1030	Van der Waals interactions	[369]
**ZIF‐4**, 3D	—	2.3	2.2	–/–	1.7	He flow at 373 K	300	Kinetic	[370]
**JXNU‐9**, 3D	6.4^a^, 9.2^a^, 11.7^a^	3.61	2.45	23.6/21.4	1.7	—	1660	C‐H···π interactions	[371]
**ScBPDC**, 3D	8.43^a^	3.42	2.41	16.4/15.4	1.7	N_2_ flow at 393 K	1777.7	C‐H···π interactions, H‐bonding	[372]
**BUT‐10**, 3D	12^a^	4.76	3.56	24/22.5	1.7	—	1726	C‐H···π interactions	[373]
**Ni‐4PyC**, 3D	5‐9^a^	3.84	3.5	29.07/37.32	1.7	He flow at 424 K	943	H‐bonding, C‐H···π interactions	[374]
**Tb‐MOF‐76**, 3D	7.9 × 7.9	2.8	3.0	25.2/22.4	1.7	—	—	H‐bonding, C‐H···π interaction	[341]
**ZIF‐80**, 3D	5.5, 9.5, 12	0.5	—	—	1.7	—	1571	—	[319]
**ZIF‐93**, 3D	17	0.67	—	—	1.7	—	1592	—	[319]
**Zn_2_(DM‐bdc)(dabco)**, 3D	—	3.91	2.96	25.8/24.2	1.7	—	1133.8	Van der Waals interactions	[358]
**Zn‐atz‐ipa**, 3D	2.8 × 2.8, 5.5 × 5.5	1.81	1.80	45.8/40.0	1.7	He flow at 333 K	650	H‐bonding	[70]
**PCN‐250(Fe_2_Zn)**, 3D	7	5.95	5.38	22.5/–	1.70	Vacuum at 423 K	1544	H‐bonding, C‐H···π interactions	[375]
**UiO‐67‐(NH_2_)_2_**, 3D	5.2 × 5.2, 7.1 × 7.1, 11.5 × 11.5	5.32	4.32	26.5/24.5	1.7	—	2022	Van der Waals interactions	[376]
**MIL‐53‐BDC**, 3D	6.3	2.93	2.78	27.2/22.3	1.7	He flow at 298 K	630	O‐H···π, C‐H···π interactions	[377]
**IRMOF‐6**, 3D	9.4, 14.7	2.67	1.60	—	1.68	—	3066	Pore size	[339]
**Ni(bdc)(ted)_0.5_**, 3D	4.0 × 4.0, 7.0 × 7.0	4.8	3.29	29/25	1.66	—	1905	Van der Waals interactions	[378]
**ZIF‐69**, 3D	40^a^	2.2	1.74	26/23	1.65	—	882	—	[379]
**DUT‐8 (Ni)**, 3D	18.37 × 18.37	5.3	2.97	25.8/24.1	1.65	He flow at 298 K	2440	C‐H···π interactions	[380]
**PCP‐FDCA**, 3D	3.8 × 5.2	3.0	3.2	35.9/33.3	1.65	—	542	H‐bonding, C‐H···π interactions	[337]
**IRMOF‐7**, 3D	10.5	1.27	0.93	—	1.65	—	3331	Pore size	[339]
**UPC‐66**, 3D	8.08 × 11.87, 10.37	2.72	2.36	15.61/17.83	1.65	—	483	C‐H···π interactions	[381]
**NKU‐0821a**, 3D	13.5 × 10.3	4.29	3.67	27.3/25.0	1.65	He flow at 373 K	962	H‐bonding, C‐H···π interactions	[382]
**CPM‐233**, 3D	6.8 × 6.8	7.4	6.5	25.4/25.0	1.64	—	1598	Pore size	[72]
**Co(bdc)(ted)_0.5_**, 3D	5.1 × 3.7, 7.6 × 7.6	4.13	2.77	29/24	1.64	—	1708	Van der Waals interactions	[378]
**Mg_2_V‐bdc‐tpt**, 3D	6.8 × 6.8	6.58	7.45	27.3/26.7	1.64	—	1597	UMCs	[72]
**LIFM‐XYY‐6**, 3D	—	2.99	2.24	29.5/28.6	1.63	He flow at 358 K	1240	H‐bonding, C‐H···π, van der Waals interactions	[383]
**ZSTU‐2**, 3D	—	2.73	2.35	33/32	1.62	298 K	862	—	[384]
**NUM‐9**, 3D	4.5 × 4.5	2.48	2.32	35.75/32.32	1.61	—	330	C‐H···π interactions, H‐bonding	[385]
**JUN‐2**, 3D	3.7 × 3.7	4.1	3.6	29.4/26.7	1.6	Vacuum at 298 K	1219	Pore size, H‐bonding	[386]
**PCN‐250(Fe)**, 3D	6.5	6.00	5.48	22.5/–	1.6	Vacuum at 423 K	1541	H‐bonding, C‐H···π interactions	[375]
**ZIF‐7**, 3D	3.0 × 3.0; 5.0 × 5.0	1.9	1.8	—	1.6	—	230	Flexibility	[387]
**USTA‐38**, 3D	4.6 × 6.6	4.6	3.3	24.4/29.4	1.6	—	1090	Pore size	[388]
**[Ni(bdc)(ted)_0.5_]**, 3D	7.6 × 7.6; 5.1 × 3.7	5.0	3.4	21.5/18.4	1.6	—	1701	Van der Waals interactions	[389]
**LIFM‐63**, 3D	5.6, 8.6	3.0	2.1	25.8/–	1.6	298 K	1486	H‐bonding, C‐H···π interactions	[390]
**ZIF‐6**, 3D	6, 9	0.77	—	—	1.6	—	3401	—	[319]
**NbU‐9**, 3D	3.8 × 3.8, 6.4 × 6.4	3.39	3.04	35.8/–	1.6	—	671.3	UMCs	[391]
**CPM‐82‐Zn**, 3D	—	4.02	3.55	25.8/25.7	1.6	—	533	Van der Waals interactions, C‐H···π interactions	[365]
**MAF‐X10(Cl)**, 3D	4.9 × 6.2	5.87	4.05	25.0/23.5	1.6	—	1751	C‐H···Cl, C‐H···π interactions	[392]
**NKCOF‐21**, 3D	14^a^	4.4	3.3	26.2/23.6	1.6	373 K	1397	H‐bonding	[393]
**NKU‐0210**, 3D	8^a^	4.73	—	21.8/–	1.6	He flow at 373 K	1290	He purging at 373 K	[394]
**PCN‐250(Fe_2_Ni)**, 3D	6.5	6.19	5.80	22.2/–	1.57	Vacuum at 423 K	1666	H‐bonding, C‐H···π interactions	[375]
**SNNU‐40**, 3D	6.5^a^	7.54	4.9	18/18.1	1.57	298 K	2233.8	H‐bonding, C‐H···π interactions	[395]
**COF‐300**, 3D	7.8	4.1	3.1	26.9/25.0	1.57	He flow at 298 K	1455	—	[349]
**ZJNU‐115**, 3D	8.58	4.21	3.75	28.2/27.7	1.56	—	1291	Van der Waals interactions, C‐H···π interactions	[396]
**ZJNU‐7**, 3D	5.0, 5.9, 7.3	4.13	3.80	29.7/29.3	1.56	—	1180	H‐bonding, C‐H···π interactions	[397]
**Cu(bdc)(ted)_0.5_**, 3D	4.8 × 3.2, 7.5 × 7.5	3.68	2.53	29/24	1.55	—	1631	Van der Waals interactions	[378]
**Zn(bdc)(ted)_0.5_**, 3D	4.8 × 3.2, 7.5 × 7.5	4.45	3.16	31/25	1.54	—	1781	Van der Waals interactions	[378]
**NbU‐12**, 3D	5 × 5	3.67	2.5	21.8/20.4	1.53	—	1395.6	C‐H···π interactions	[398]
**MUV‐11**, 3D	—	1.83	1.72	25/23	1.53	298 K	180	—	[384]
**MIL‐53‐NDCA**, 3D	8.4	4.24	3.12	24.2/17.0	1.53	He flow at 298 K	1590	O‐H···π, C‐H···π interactions	[377]
**NPU‐2**, 3D	10.5^a^	4.44	3.45	19.64/18.18	1.52	He flow at 313 K	2133	H‐bonding, C‐H···π interactions	[321]
**PCN‐250(Fe_2_Co)**, 3D	6.5	6.21	5.82	22.2/–	1.52	Vacuum at 423 K	1675	H‐bonding, C‐H···π interactions	[375]
**CPM‐35**, 3D	6.3^a^	3.57	2.59	23.3/21.4	1.51	—	783	C‐H···π interactions	[399]
**CPM‐223‐tpbz**, 3D	6.8	6.88	6.25	21.9/23.3	1.51	—	1662	Pore size	[72]
**UiO‐67**, 3D	11.7^a^, 16.1^a^	4.43	2.95	—	1.5	—	1775	—	[373]
**Cr‐BTC(O_2_)**, 3D	25, 29	3.3	2.89	37.2/24	1.5	Vacuum at 298 K	1135	H‐bonding, UMCs	[400]
**UPC‐613**, 3D	7^a^, 12^a^	2.55	2.31	31.83/28.51	1.5	—	853	UMCs	[401]
**Ni_2_(HBTC)_2_(bpy)_0.6_(dabco)_1.4_**, 3D	5.3^a^	4.8	4.6	33.5/31.7	1.5	—	1070	H‐bonding, Van der Waals interactions	[402]
**CPM‐723**, 3D	6.8	6.91	6.67	21.7/20.0	1.5	—	1369.8	Pore size	[72]
**1a‐tz**, 3D	7.3 × 11.8	3.4	3.3	35/33	1.5	—	845	H‐bonding	[403]
**MIL‐142a**, 3D	7.0 × 7.0	3.8	2.9	27.3/25.1	1.5	—	1580	Van der Waals interactions	[404]
**MAF‐X10**, 3D	4.9 × 6.8	5.07	3.57	23.4/22.4	1.5	—	1644	H‐bonding, C‐H···π interactions	[392]
**NKCOF‐22**, 3D	16^a^	2.9	1.8	25.9/24.1	1.5	373 K	1580	—	[393]
**COF‐320**, 3D	13.5 × 6.2	2.4	1.8	26.9/25.1	1.49	He flow at 298 K	923	—	[349]
**CPM‐736**, 3D	5.9	4.03	3.88	30.4/30.1	1.48	—	472.5	Pore size	[72]
**COF‐102**, 3D	11.5	1.9	1.6	28.7/25.1	1.48	He flow at 298 K	33122	—	[349]
**A‐66**, 3D	5.6 × 6.8	6.56	4.45	23.7/22.6	1.48^h^	—	—	H‐bonding, C‐H···π interactions	[405]
**MIL‐53‐BPDC**, 3D	10.9	2.97	2.07	22.1/22.1	1.47	He flow at 298 K	1780	O‐H···π, C‐H···π interactions	[377]
**Azole‐Th‐1**, 3D	10	4.5	3.6	28.6/26.1	1.46	He flow at 298 K	983	Van der Waals interactions	[406]
**PCN‐250(Fe_2_Mn)**, 3D	6.5	5.53	5.24	21.4/–	1.45	Vacuum at 423 K	1391	H‐bonding, C‐H···π interactions	[375]
**MIL‐125**, 3D	—	4.83	3.98	23.6/17	1.43	298 K	1435	H‐bonding, C‐H···π interactions	[384]
**DUT‐8 (Cu)**, 3D	18.47 × 18.47	4.74	2.44	25.5/18.3	1.43	He flow at 298 K	2370	C‐H···π interactions	[380]
**CPM‐238**, 3D	5.9	5.56	5.25	24.7/24.4	1.43	—	1444	Pore size	[72]
**CPM‐738**, 3D	5.9	1.67	4.56	27.9/26.5	1.42	—	1161.5	Pore size	[72]
**Zn‐PNMI**, 3D	6.4 × 6.4	1.6	1.7	24.5/23.8	1.42	Vacuum at 393 K	305	H‐bonding	[407]
**CPM‐63 m**, 3D	9.58	2.84	2.53	31.8/29.8	1.41	—	1023	Open Lewis basic N sites	[408]
**In‐soc‐MOF‐1**, 3D	7.65 × 5.65; 10 × 10	4.0	3.7	28.4/25.2	1.4	—	1223	Pore size, van der Waals interactions	[409]
**UTSA‐33**, 3D	5.4 × 6.5; 4.8 × 5.8	2.8	2.7	32/32	1.4	—	660	Pore size	[410]
**UTSA‐35**, 3D	7.7 × 5.8	2.4	2.1	30/28	1.4	—	742	Pore size, van der Waals interactions	[411]
**UPC‐612**, 3D	14^a^, 20^a^	3.58	2.8	22.39/16.94	1.4	—	2016	UMCs	[401]
**Zr‐bptc**, 3D	7^a^, 13^a^	3.26	3.08	27/24	1.4	Vacuum at 393 K	1085.3	Electrostatics	[412]
**Ni(HBTC)(bpy)**, 3D	5.5^a^	6.6	6.0	33.6/32.2	1.4	—	1474	H‐bonding, Van der Waals interactions	[402]
**LIFM‐31**, 3D	6.8, 11.8	2.4	1.8	26.9/–	1.4	298 K	1711	—	[390]
**LIFM‐62**, 3D	5.9, 11.7	2.6	2.0	24.7/–	1.4	298 K	1977	—	[390]
**Mn‐PNMI**, 3D	8.0 × 8.0	2.8	2.0	23.5/16.9	1.38	Vacuum at 393 K	818	H‐bonding	[407]
**Dy‐BTC**, 3D	—	1.90	1.90	32/27	1.37	—	947	Van der Waals interactions	[348]
**NKCOF‐62**, 3D	7^a^	2.21	2.0	29.6/26.3	1.37	He flow at 373 K	714	Van der Waals interactions	[413]
**MOF‐841**, (273 K) 3D	5.1 × 5.1	3.4	4.7	24.8/20.8	1.35	He flow at 298 K	1426	C‐H···π interactions, van der Waals interactions	[414]
**NPU‐1**, 3D	7.4^a^	4.5	3.2	29.1/23.95	1.32	He flow at 313 K	1580	H‐bonding, C‐H···π interactions	[321]
**MOF‐545**, 3D	13^a^, 33^a^	3.12	2.57	22.7/21.5	1.31	Vacuum at 393 K	2265.4	C‐H···π interactions, H‐bonding	[415]
**Cu(ina)_2_**, 2D	4.1 × 4.1	2.0	1.9	—	1.3	—	228	—	[317]
**MIL‐53 (Al)**, 3D (323K)	8.5 × 8.5, 2.6 × 13.6	2.05	1.69	22.5/20.5	1.3	—	—	—	[416]
**MAF‐X10(Me)**, 3D	4.9 × 5.8	5.01	4.01	26.7/21.2	1.3	—	1782	H‐bonding, C‐H···π interactions	[392]
**LIFM‐61**, 3D	7.3, 11.8	1.7	1.4	24.1/–	1.3	298 K	1194	—	[390]
**CPOC‐301**, 3D	12 × 6	3.37	3.92	32.4/24.2	1.3	He flow at 373 K	1962	C‐H···π interactions	[417]
**Co_3_(*μ*_3_‐OH)(tipa)(bpy)_1.5_**, 3D	4.8 × 4.8	1.34	1.36	37.2/33.6	1.3	—	408.4	C‐H···π, π···π interaction	[418]
**NKCOF‐23**, 3D	17^a^	2.7	2.2	24.3/23.0	1.3	373 K	1900	—	[393]
**Cd‐PNMI**, 3D	7.6 × 7.6	1.9	1.4	19.4/13.8	1.27	Vacuum at 393 K	264	H‐bonding	[407]
**Zn‐atz‐oba**, 3D	3.2 × 4.4	1.7	1.7	30/27	1.27	He flow at 333 K	710	H‐bonding	[419]
**BUT‐151**, 3D	11.2 × 11.2	3.99	3.72	31.06/23.95	1.26		1330	Pore size, C‐H···π interactions	[420]
**DBA‐3D‐COF‐1**, 3D	28	1.7	1.7	16.8/15.9	1.24	—	5083	—	[421]
**MOF‐808‐Ind**, 3D	12, 14.8	1.68	1.39	28.43/26	1.23	—	948	H‐bonding, C‐H···π interactions	[357]
**MOF‐808‐Izo**, 3D	12, 14.8	1.74	1.56	28.61/26.73	1.22	—	1130	H‐bonding, C‐H···π interactions	[357]
**COF‐8**, 2D	18.7	1.6	1.5	27.5/25.1	1.21	He flow at 298 K	1180	—	[349]
**TJT‐100**, 3D	8.7 × 11.6	3.7	3.4	29/25	1.2	—	890	H‐bonding, van der Waals interactions	[422]
**LIFM‐28**, 3D	6.8, 11.8	1.0	0.9	26.3/–	1.2	298 K	927	—	[390]
**COF‐6**, 2D	6.4	2.1	2.1	29.2/27.5	1.20	He flow at 298 K	706	—	[349]
**NH_2_‐MIL‐125**, 3D	—	4.69	4.41	25/23	1.18	298 K	1180	—	[384]
**BUT‐150**, 3D	13.0 × 13.0	4.30	4.26	23.87/23.68	1.15	—	1641	Pore size, C‐H···π interactions	[420]
**Ni‐DBA‐3D‐COF**, 3D	26	1.83	1.83	11.6/9.7	1.15	—	4763	UMCs	[421]
**COF‐10**, 2D	31.7	1.0	0.9	26.6/25.1	1.13	He flow at 298 K	2056	—	[349]
**MOF‐808**, 3D	7.3, 18	1.14	1.05	15.37/14.59	1.05	—	1574	—	[357]
**CTF‐DCTC‐400**, 3D	7.3, 11.8	1.82	1.68	22.7/22.0	1.04	Vacuum at 298 K	771	Pore size	[340]
**Au‐PCM‐102**, 3D	6.7 × 8.2	2.7	2.5	—	—	—	1449	Van der Waals interactions	[423]
**Ag‐PCM‐102**, 3D	6.7 × 8.2	3.5	2.9	—	—	—	1558	Van der Waals interactions	[423]
**RPM3‐Zn**, 3D	—	1.56	0.89	—	—	—	328	Flexibility, H‐bonding	[264]
									
**IRMOF‐4**, 3D	9.2	3.09	1.45	—	—	—	1438	Pore size	[339]
**IRMOF‐5**, 3D	7	1.73	0.91	—	—	—	861	Pore size	[339]
**Cu(1,3‐bdc)(ted)_0.5_**, 3D	22	2.43	2.15	23.8/26.5	—	—	1673	H‐bonding, C‐H···π interactions	[424]
**Co(1,3‐bdc)(ted)_0.5_**, 3D	—	2.58	2.13	34/29.3	—	—	1802	H‐bonding, C‐H···π interactions	[424]
**UTSA‐34a**, 3D	12.8	2.79	2.63	—	—	—	991	UMCs	[425]
**FJI‐C4**, 3D	5.5	2.96	2.74	32.7/33.1	—	—	690	Pore size	[426]
**NTU‐24**, 3D	0.86 × 0.86, 4.6 × 4.6	5.27	4.55	29/24	—	He flow at 298 K	1620	Pore size, shape	[427]
**NTU‐25**, 3D	1.22 × 1.22, 4.88 × 4.88	4.38	4.24	31/27	—	He flow at 298 K	1540	Pore size, shape	[427]
**CPM‐63a**, 3D	9.58	2.86	2.51	33.9/30	—	—	1127	Open Lewis basic N sites	[408]

^a^ Determined from Horvath–Kawazoe/DFT method applied on N_2_ isotherm at 77 K.

^b^ IAST selectivity at 1 bar for 1:1 (v/v) C_2_H_6_/C_2_H_4_.

^c^ Adsorption pressure at 40kPa.

^d^ Adsorption pressure at 88.5 kPa.

^e^ IAST selectivity at 1 bar for 1:9 (v/v) C_2_H_6_/C_2_H_4_.

^f^ Pore size determined from CO_2_ adsorption isotherms recorded at 195 K.

^g^ Langmuir surface areas determined from the corresponding CO_2_ adsorption isotherms recorded at 195 K.

^h^ Uptake ratio.

**TABLE 10:** C_3_H_4_/C_3_H_6_ binary separations. The following parameters are listed for comparison: pore size; BET surface area (S_BET_); single‐component gas uptakes; adsorption enthalpies (*Q*_st_); adsorption selectivity; regeneration temperature; and attributed mechanisms. Reticular sorbents are listed in decreasing order of binary selectivity for C_3_H_4_ selective sorbents (above) and C_3_H_6_ selective sorbents (below).

Adsorbent, network dimensionality (nD)	Pore size (Å)	C_3_H_4_ uptake at 1 bar (mmol/g)	C_3_H_6_ uptake at 1 bar (mmol/g)	*Q*_st_ (kJ/mol)	Selectivity^a^	Regeneration temperature	S_BET_ (m^2^/g)	Mechanism	Refs.
**NKMOF‐1‐Ni**, 3D	5.7 × 5.7	3.5	2.1	54.0/38.0	1217.8^e^	—	374	H‐bonding, π···π interactions	[428]
**NKMOF‐11**, 3D	5.7 × 5.7	3.1	1.5	84.8/30.5	1074^d^	He flow at 363 K	376	H‐bonding, π···π interactions	[429]
**BUT‐306**, 3D	3	1.32	0.11	—	636	He flow at 393 K	305	Dipole‐dipole, C‐H···π interactions	[430]
**FJI‐W1**, 3D	8 × 8	7.09	6.27	61.7/36.1	593^d^	—	1376	H‐bonding	[431]
**TIFSIX‐14‐Cu‐i**, 3D	3.4^c^	3.88	1.40	57/49	355	He flow at 338 K	481^c^	Flexibility, H‐bonding, Van der Waals interactions	[432]
**ZU‐16‐Co**, 3D	3.62 × 3.62	2.45	2.1	87.5/–	248^d^	Ar flow at 373 K	—	H‐bonding	[433]
**sql‐NbOFFIVE‐bpe‐Cu‐AB**, 2D	3.96 × 5.56	3.04	2.10	69/53	220	Vacuum at 333 K	295^d^	H‐bonding	[434]
**GeFSIX‐14‐Cu‐i** (**ZU‐33**), 3D	3.4 × 3.4	3.34	1.5	54/–	217^d^	He flow at 338 K	463^c^	H‐bonding	[432]
**Co‐gallate**, 3D	4.0^c^	3.21	1.49	82/47	152	He flow at 393 K	475	H‐bonding, Van der Waals interactions	[435]
**UTSA‐200**, 3D	3.4 × 4.2	3.58	1.20	62.3/45.4	149.5	—	617	Flexibility, pore size, H‐bonding	[436]
**Ni‐gallate**, 3D	3.9^c^	2.65	0.90	84/53	113	He flow at 393 K	424	H‐bonding, Van der Waals interactions	[435]
**ELM‐12**, 2D	6.1 × 4.3 × 4.3, 6.8 × 4.0 × 4.2	2.74	1.38	60.6/15.8	84	He flow at 323 K	740 [437]	H‐bonding	[438]
**ZJUT‐1a**, 3D	7.5 × 3.7 × 3.7	3.97	1.25	38/–	70	—	222	H‐bonding, Van der Waals interactions	[439]
**SIFSIX‐3‐Ni**, 3D	3.8 × 5.0	2.98	2.67	68/47	76	He flow at 393 K	351	Pore size, shape, H‐bonding	[440]
**Mg‐gallate**, 3D	4.1^c^	3.75	1.50	66/44	65	He flow at 393 K	559	H‐bonding, Van der Waals interactions	[435]
**NTU‐88**, 2D	4.4 × 7.9	3.84	0.07	29.4/27.5	52.7^f^	—	420	Molecular sieving	[441]
**ZU‐62** (**NbOFFIVE‐2‐Cu‐i**), 3D	6.75, 6.94, 7.20	3.60	2.60	71/56	48	—	476	H‐bonding, Van der Waals interactions	[37]
**Cu(dps)_2_(GeF_6_)**, (**GeFSIX‐dps‐Cu**), 3D	1.8 × 2.6	3.7	0.08	50.2/–	46.5^f^	—	382	Molecular sieving	[244]
**CPOS‐1**, 3D	5.3 × 5.3	0.93	0.36	44.2/31.6	43.9^d^	—	336.2	Van der Waals interactions	[442]
**Zeolite 5A (yNa^+^)**, 3D	4.4‐5.2^c^	3.62	2.58	52/30	43	He flow at 298 K	503	Pore size	[443]
**SIFSIX‐2‐Cu‐i**, 3D	5.2 × 5.2	3.80	2.60	45/37	25	He flow at 298–308 K	586	Pore size, shape, H‐bonding	[440]
**NTU‐100‐NH_2_**, 3D	5.9^b^	6.7	5.58	43.5/40	23.2	—	947	H‐bonding	[444]
**SIFSIX‐Cu‐TPA** (**ZNU‐2‐Si**), 3D	6.27‐9.84^b^	8.46	—	39.35/34.26	19.3	Ar flow at 298 K or vacuum	1339	H‐bonding, van der Waals interactions	[445]
**TIFSIX‐Cu‐TPA** (**ZNU‐2‐Ti**), 3D	6.56‐9.4^b^	7.66	—	43.5/35	16.2	Ar flow at 298 K or vacuum	1380	H‐bonding, van der Waals interactions	[445]
**NbOFFIVE‐Cu‐TPA** (**ZNU‐2‐Nb**), 3D	7.85‐9.4^b^	7.28	—	42/32.5	13.8	Ar flow at 298 K or vacuum	1281	H‐bonding, van der Waals interactions	[445]
**ZNU‐2**, 3D	8.5 × 8.5, 8.8 × 4	7.70	5.30	43/34.5	12.5	—	1380	Pore size, shape, H‐bonding, Van der Waals interactions	[446]
**SIFSIX‐1‐Cu**, 3D	8.0 × 8.0	8.76	5.90	49/35	9	He flow at 298–308 K	1128	Pore size, shape, H‐bonding	[440]
**Cu‐FINA‐2**, 3D	5.48 × 4.87	1.36	0.67	45.3/40.3	6.1	Vacuum	175.9	H‐bonding	[447]
**BUT‐309**, 3D	4.1 × 4.1	1.07	0.8	84.3/50.8	5.2	He flow at 298 K	—	H‐bonding	[448]
**Cu‐FINA‐1**, 3D	5.68 × 4.76	1.8	1.41	45.5/44.5	4.5	Vacuum	389.4	H‐bonding	[447]
**MFM‐300(In)**, 3D	6.8^b^	6.25	5.20	35/31	3.7	—	1030	Van der Waals interactions	[369]
**SIFSIX‐3‐Ni**, 3D	5.03 × 3.75	3.28	2.9	45/30	3.6	He flow at 298 K	368	Molecular sieving (Kinetic)	[449]
**Cu‐INA**, 3D	7.86 × 6.95	2.8	2.1	43/39	3.1	Vacuum	428.7	H‐bonding	[447]
**JXNU‐6**, 3D	10.4 × 10.4	5.07	3.57	39.9/27.1	3.1^d^	—	856.3	H‐bonding	[450]
**BUT‐310**, 3D	9 × 9	10.8	7.8	32.3/30.6	2.73	298 K	1811	UMCs	[451]
**Cu‐APC**, 3D	5.1 × 7.2	3.75	1.73	29.9/25.7	2.6	298 K	655.5	H‐bonding, C‐H···π interactions	[333]
**NbOFFIVE‐1‐Ni**, 3D	4.66 × 3.21	2.99	2.39	38/30	2.4	He flow at 298 K	248	Molecular sieving (Kinetic)	[449]
**NKCOF‐36**, 3D	6, 9^b^	8.49	6.49	39.7/37.4	2.22 (1/99)	373 K	1093	Van de Waals interactions, pore size	[115]
**NKCOF‐37**, 3D	6, 10^b^	7.80	6.04	37.7/36.6	2.06 (1/99)	373 K	1838	Van de Waals interactions, pore size	[115]
**COF‐1**, 2D	15 × 15	4.61	3.61	–/–	1.59	—	640	—	[115]
**JXNU‐15(NH_2_)**, 3D	10.9	12.1	9.8	24.3/20.9	1.48^d^	—	2458	H‐bonding, C‐H···π interactions	[452]
**NTU‐100‐NO_2_**, 3D	6.2^b^	4.94	3.48	32.5/31	1.43	—	688	H‐bonding	[444]
**JXNU‐15**, 3D	11.8	13.9	11.2	17.8/15.7	1.28^d^	—	2806	H‐bonding, C‐H···π interactions	[452]
**BUT‐308**, 3D	2.1 × 2.1	4.36	2.19	—	—	He flow at 298 K	—	H‐bonding	[448]
**BUT‐305**, 3D	—	1.98	1.96	—	—	He flow at 393 K	351	—	[430]
**SIFSIX‐3‐Ni**, 3D	3.6	3.32	2.73	45/30	—	He flow at 340 K	368	—	[453]
**FCOF‐5**, 3D	—	3.51	1.97	30.91/27.92	—	He flow 363 K	—	Flexibility	[454]

^a^ For v/v = 1/1 equimolar mixture, predicted by IAST.

^b^ Pore size, BET surface area calculated from the N_2_ sorption isotherm at 77K.

^c^ Pore size, BET surface area calculated from the CO_2_ sorption isotherm at 195K, 273K.

^d^ For v/v = 1/99 mixture, predicted by IAST.

^e^ For v/v = 0.5/99 mixture, predicted by IAST.

^f^ Uptake ratio.

^g^ Adsorption selectivity.

**TABLE 11:** C_3_H_6_/C_3_H_8_ binary separations. The following parameters are listed for comparison: pore size; BET surface area (S_BET_); single‐component gas uptakes; adsorption enthalpies (*Q*_st_); adsorption selectivity; regeneration temperature; and attributed mechanisms. Reticular sorbents are listed in decreasing order of binary selectivity for C_3_H_6_ selective sorbents (above) and C_3_H_8_ selective sorbents (below).

Adsorbent, network dimensionality (nD)	Pore size (Å)	C_3_H_6_ uptake at 1 bar (mmol/g)	C_3_H_8_ uptake at 1 bar (mmol/g)	*Q*_st_ (kJ/mol)	Selectivity^a^	Regeneration temperature	S_BET_ (m^2^/g)	Mechanism	Refs.
**HAF‐1**, 3D	4.14	1.22	0.04	65.88/–	1.67 ×10^7^	He flow at 423 K	410.6	Molecular sieving, H‐bonding, C‐H⋅⋅⋅π interactions	[455]
**UTSA‐400**, 3D	3.2 × 3.2	1.84	0.05	60.5/–	10^7^	He flow at 298 K	226^b^	H‐bonding, van der Waals interactions	[456]
**NTU‐85‐WNT**, 3D	4.57 × 4.57	0.45	0.003	49.9/–	1570	Vacuum at 323 K	220	H‐bonding	[457]
**Zn(ox)_0.5_(trz)**, 3D (323K)	2.9	2.29	—	—	1565	—	546	Kinetics (Pore size)	[458]
**Cd‐HFDPA**, 3D	7.3 × 7.3	1.16	0.96	—	827	—	171.98	π complexations, electrostatics	[459]
**MIP‐203**, 3D (303 K)	6.3	0.33	0.45	—	551.4	—	5.8	Flexibility, H‐bonding, electrostatics	[460]
**JNU‐3a**, 3D	5.6^e^	2.62	2.14	29.3/16.1	513	He flow at 298 K	588	Flexibility, molecular sieving	[461]
**Co‐gallate**, 3D	4.2 × 5.1	1.79	0.14	41/–	330	—	486.8	H‐bonding, O‐H⋅⋅⋅π, van der Waals interactions	[462]
**FDC‐4**, 3D	3.76 × 3.76	5.43	0.88	35/–	318	—	—	Molecular sieving	[463]
**CAU‐10‐OMe**, 3D	—	1.4	0.3	—	276	—	179	Flexibility	[464]
**Zn(ox)_0.5_(atrz)**, 3D (323K)	3.3	1.64	—	—	220	—	521	Kinetics (Pore size)	[458]
**ELM‐12**, 2D	4 × 4	1.48	1.36	30/28	204	—	—	Kinetics	[465]
**HIAM‐301**, 3D	4.6^d^	3.16	0.27	27/–	150	—	579	H‐bonding, π⋅⋅⋅π interactions	[466]
**1to**, 3D	4.7–5.0	3.07	1.70	38.1/9	80	He flow at 423 K	1360	π‐complexation, van der Waals interactions	[467]
**[Zn(2‐cim)_2_]**, 3D	12.5 × 12.5	2.26	2.39	34/30	60	—	—	Kinetics (Pore size)	[468]
**Milli‐Zn‐ATA**, 3D	3.7	1.0	—	44.9/34.8	60^e^	N_2_ flow at 373 K	349	π⋅⋅⋅π interactions	[469]
**ATC‐Cu**, 3D	6 × 6	3.62	3.16	69.86/39.92	58.7	Vacuum	—	UMCs	[470]
**Fe_2_(m‐dobdc)**, 3D	—	7.5	6.0	73/42	55	—	—	UMCs	[295]
**ZJU‐75a**, 3D	4.1 × 4.4	3.31	2.33	65.9/33.1	54.2	He flow at 323 K	391	UMCs	[471]
**Co‐MOF‐74**, 3D	11‐12	7.29	—	49/–	45	C_3_H_8_ Flow	—	UMCs	[472]
**1tm**, 3D	—	2.30	1.92	18.2/28.5	40.8	He flow at 423 K	1171	H‐bonding, C‐H⋅⋅⋅π interactions	[467]
**Mn_2_(m‐dobdc)**, 3D	—	7.5	6.0	70/38	40	—	—	UMCs	[295]
**Co_2_(m‐dobdc)**, 3D	—	7.5	6.0	53/52.5	39	298 K	—	UMCs	[295]
**NTU‐65‐CoTi**, 3D	3.5 × 4.2, 4.7 × 6.0	2.56^g^	0.07^g^	—	37.05^h^	—	1078.5	Flexibility	[473]
**Ni_2_(m‐dobdc)**, 3D	—	7.5	6.0	55/45	35	298 K	—	UMCs	[295]
**ZnAtzPO_4_**, 3D	3.8	1.1	0.3	27.5/–	31	N_2_ flow at 298 K	420	H‐bonding	[474]
**Ni(AIP)(BPY)_0.5_**, 3D	6.0	1.94	0.45	48.7/–	31	—	355	π‐complexations	[475]
**Co(AIP)(BPY)_0.5_**, 3D	6.6	1.99	0.49	41.79/–	21	—	260	π‐complexations	[475]
**Co_2_(5‐aip)_2_(bpy)**, 3D	—	2.0	0.5	42.37/33.68	21	He flow at 423 K	—	C‐H⋅⋅⋅π interactions	[476]
**Zn(AIP)(BPY)_0.5_**, 3D	7.1	1.87	0.59	40.67/–	20	—	344	π‐complexations	[475]
**Zn_2_(5‐aip)_2_(bpy)**, 3D	—	1.91	0.76	46.2/25.5	19.8	423 K	—	UMCs	[477]
**MFM‐520**, 3D	—	2.33	2.03	53.4/43.2	17	He flow at 318 K	313	H‐bonding, π⋅⋅⋅π interactions	[478]
**MOF‐74‐Mn**, 3D	11	7.2	6.0	54/35	16.6	—	1797	UMCs	[305]
**1tp**, 3D	—	2.00	1.50	15.2/7	15.3	He flow at 423 K	893	H‐bonding, C‐H⋅⋅⋅π interactions	[467]
**GeFSIX‐3‐Ni**, 3D	4.42	2.0	0.55	42/22.8	15	He flow at 373 K	246	H‐bonding, π⋅⋅⋅π interactions	[479]
**MOF‐74‐Fe**, 3D	11	6.9	6.2	44/33	14.7	—	1536	UMCs	[66]
**1**, 3D	5.3, 6.4	1.92	2.13	11.8/20	14.3^h^	He flow at 423 K	859	π‐complexations, C‐H⋅⋅⋅π, van der Waals interactions	[467]
**Fe‐MOF‐74**, 3D (318K)	12 × 12	6.24	5.19	44/33	13.6	—	—	UMCs	[295]
**Cu(0.6)@MIL‐100(Fe)**, 3D	5.5	—	—	—	13.2	298 K	1490	UMCs	[480]
**BTO‐MOF**, 3D	3.05 × 3.05	0.59	0.45	—	12	—	283	Kinetics (Pore size)	[481]
**DBTO‐MOF**, 3D	3.34 × 3.34	1.0	0.84	—	11	—	457	Kinetics (Pore size)	[481]
**Ni‐NP**, 3D	6.3	3.57	2.13	57/32.3	10.5	He flow at 373 K	543	UMCs	[482]
**MOF‐74‐Ni**, 3D	11	7.0	5.7	52/36	10.4	—	1532	UMCs	[305]
**GeFSIX‐3‐Co**, 3D	4.72	1.9	1.09	—	10	He flow at 373 K	—	H‐bonding, π⋅⋅⋅π interactions	[479]
**NTU‐65‐CoZr**, 3D	3.2 × 4.1, 4.8 × 5.5	0.38^g^	0.04^g^	—	8.91^h^	—	537.3	Flexibility	[473]
**MIP‐202**, 3D	6.3	0.30	0.13	—	8.8	—	13.1	H‐bonding, electrostatics	[460]
**MAF‐23‐O**, 3D	—	1.34	1	54/34	8.8	—	—	H‐bonding	[483]
**MOF‐74‐Co**, 3D	11	6.8	5.9	51/35	8.6	—	1438	UMCs	[305]
**NiNi‐Pyz**, 3D	3.9 × 3.8	3.26	2.77	52/–	7.8	—	448.7	UMCs, C‐H⋅⋅⋅π interactions	[484]
**NTU‐85‐F**, 3D	7.031	0.886	1.02	61.2/55.3	7.0	—	350	H‐bonding	[457]
**AGTU‐3a**, 3D	—	0.5	1.2	68/34	7	—	227	UMCs	[485]
**MIL‐101(Cr)‐SO_3_H‐Ag**, 3D	15	4.3	3.0	—	6.0	—	1253	UMCs	[296]
**MOF‐74‐Mg**, 3D	11	7.5	6.0	47.5/35	5.5	—	1835	UMCs	[305]
**Py^1/3^@Cu‐BTC**, 3D	8.5	7.0	6.7	—	5.5	—	1510	Electrostatics	[486]
**[Zn_2_(BDC‐ Cl)_2_(Py_2_TTz)]*_n_***, 3D	5.4, 4.9	3.32	1.62	—	5.2	He flow at 298 K	441	Flexibility	[487]
**SIFSIX‐2‐Cu‐i**, 3D	4.7	2.65	—	35.82/20	5.0	338 K	—	H‐bonding, π⋅⋅⋅π interactions	[488]
**FePt‐M′MOF**, 3D	4.2	2.1	2.05	46.3/–	4.7	—	432	UMCs	[489]
**NJU‐Bai8**, 3D	4.0 × 3.3	2.89	2.89	—	4.6	Vacuum at 338 K	1048	Flexibility	[490]
**FeNi‐Pyz**, 3D	4.0 × 4.0	3.08	3	51.4/–	4.3	—	—	UMCs, C‐H⋅⋅⋅π interactions	[484]
**CoNi‐Pyz**, 3D	3.9 × 4.1	3.38	3.19	51/–	4.2	—	—	UMCs, C‐H⋅⋅⋅π interactions	[484]
**GeFSIX‐2‐Cu‐i**, 3D	4.5	2.69	1.8	36.25/20.37	4.0	338 K	—	H‐bonding, π⋅⋅⋅π interactions	[488]
**MOF‐74‐Zn**, 3D	11	6.3	5.5	46/34	3.9	—	1277	UMCs	[305]
**MUF‐17**, 3D	5.7	2.44	2.02	49/17.5	3.8	—	310	Electrostatics	[491]
**NKU‐FlexMOF‐1**, 3D	—	3.20	2.33	61.1/52.5	2.31	—	952	Flexibility, electrostatics	[492]
**MIL‐100(Cr)‐DAA**, 3D	—	6.5	7.5	49.5/36	2	298 K	3501.6	Electrostatics	[493]
**[Co(mpba)_2_]**, 3D	5.4 × 3.9	1.13	1.10	—	1.81	—	220^f^	Flexibility	[494]
**NTU‐65‐FeTi**, 3D	3.8 × 4.1, 5.0 × 6.0	0.06^g^	0.03^g^	—	1.74^h^	—	931.8	Flexibility	[473]
**Cu_x_O_y_ @HP‐Cu‐BTC(CTAB)**, 3D	7, 12.9	3.42	3.06	18.38/19.47	1.66	—	817.5	H‐bonding, van der Waals, π⋅⋅⋅π interactions	[495]
**[Mn(mpba)_2_]**, 3D	3.1 × 3.0	1.42	1.33	—	1.64	—	231^f^	Flexibility	[494]
**Cu_x_O_y_@HP‐Cu‐BTC(SDBS)**, 3D (313K)	8, 9	6.09	5.03	1.95/25.44	1.53	—	620	H‐bonding, van der Waals, π⋅⋅⋅π interactions	[495]
**NTU‐65‐FeZr**, 3D	3.5 × 4.3, 5.0 × 5.6	0.03^g^	0.02^g^	—	1.45^h^	—	1066.1	Flexibility	[473]
**ZIF‐7**, 3D	—	2.4	2.3	—	1.25	—	—	Flexibility	[387]
**MAF‐23**, 3D	3.6	1.23	0.85	60.2/57.9	1.2	—	—	H‐bonding	[483]
**MIL‐101(Cr)**, 3D	—	7.9	6.2	36.9/36.3	1.2	298 K	3179.7	Electrostatics	[493]
**MIL‐101‐Cr‐SO_3_H**, 3D	15	4.5	3.8	—	1.1	—	1856	UMCs	[296]
**Cu_x_O_y_ @HP‐Cu‐BTC(N,N)**, 3D	—	4.74	4.59	3.61/18.33	0.94	—	1194.1	H‐bonding, van der Waals, π⋅⋅⋅π interactions	[495]
**CPL‐1**, 3D	10–13	1.82	0.29	—	—	298 K	330	Flexibility	[496]
**Zn_3_(OH)_2_(pzdc)(atz)**, 3D	4.0 × 2.1, 3.7 × 1.3	2.1	—	—	—	—	—	Flexibility, molecular sieving	[497]
**Y‐dbai**, 3D	4.4	2.57	0.1	55/–	—	Ar flow at 423 K	405.4	Molecular sieving	[498]
**Y‐abtc**, 3D	4.7^c^	2	0.07	50/–	—	—	427	Pore size	[499]
**Cu‐BTC**, 3D	—	8.0	6.8	41.9/28.7	—	—	1500–2100	Cation⋅⋅⋅π	[500]
**MIL‐100(Fe)**, 3D	—	1.3	0.6	—	—	—	2266	UMCs	[501]
**Ftw‐MOF‐ABTC**, 3D	4.44 × 2.35	2.30	2.46	48.8/38	—	—	—	Kinetics	[502]
**KAUST‐7 (NbOFFIVE‐1‐Ni)**, 3D	4.75 × 3.05	0.6	0.1	57.4/–	—	He flow at 298 K	280	H‐bonding	[38]
**Zn‐ATZ‐IP(OH)‐a**, 3D	2.7 × 2.7	0.65	0.05	55.2/–	—	—	270	Pore size, N‐H⋅⋅⋅π	[503]

^a^ IAST selectivity at 1 bar for 1:1 (v/v) C_3_H_6_/ C_3_H_8_.

^b^ The Brunauer–Emmett–Teller surface area calculated from CO_2_ isotherm at 298K.

^c^ Pore size calculated from CO_2_ isotherm at 298K.

^d^ Pore size calculated from N_2_ isotherm at 77K.

^e^ Pore size calculated from Ar isotherm at 87K.

^f^ Langmuir surface area.

^g^ Uptake at 0.5 bar.

^h^ Uptake ratio.

^i^ Separation factor.

**TABLE 12:** C_3_H_8_/C_3_H_6_ binary separations. The following parameters are listed for comparison: pore size; BET surface area (S_BET_); single‐component gas uptakes; adsorption enthalpies (*Q*_st_); adsorption selectivity; regeneration temperature; and attributed mechanisms. Reticular sorbents are listed in decreasing order of binary selectivity for C_3_H_8_ selective sorbents (above) and C_3_H_6_ selective sorbents (below).

Adsorbent, network dimensionality (nD)	Pore size (Å)	C_3_H_8_ uptake at 1 bar (mmol/g)	C_3_H_6_ uptake at 1 bar (mmol/g)	*Q*_st_ (kJ/mol)	Selectivity (binary)^a^	Regeneration temperature	S_BET_ (m^2^/g)	Mechanism	Refs.
**Ni(ADC)(TED)_0.5_**, 3D	4.8^b^	2.32	2.11	65.3/56.5	6.4	He flow at 298 K	679	C‐H⋅⋅⋅π interactions	[504]
**Ni(bpe)_2_(WO_4_)**, 3D	5.6 × 5.6	0.67	0.57	42/36	2.75	Ar flow at 328 K	—	Pore shape, electrostatics	[505]
**JUN‐90**, 3D	5.1 × 5.15, 5.6 × 5.6	2.41	2.19	38.2/33.7	2.7	—	294	H‐bonding, C‐H⋅⋅⋅π interactions	[506]
**PCP‐IPA**, 3D	4.7 × 5.6	2.23	2.25	50.94/43.36	2.48	N_2_ flow at 373 K	486.7	H‐bonding, van der Waals interactions	[325]
**FDMOF‐2**, 3D	6.0^b^	5.04	4.15	34.6/30.9	2.18	298 K	1011	C‐H⋅⋅⋅π interactions, H‐bonding	[507]
**NUM‐7**, 3D	4.7 × 7.8	2.98	3.1	40.03/38.19	1.7	—	345 [367]	C‐H⋅⋅⋅π interactions	[508]
**WOFOUR‐1‐Ni**, 3D	5.6 × 5.6	1.16	0.89	42/36	1.6	Ar flow at 328 K	315 [509]	Van der Waals interactions	[505]
**Zr‐BPYDC**, 3D	11.8^b^	6.8	7.2	32.5/29.5	1.6	—	2080	Van der Waals interactions	[510]
**MoFOUR‐1‐Ni**, 3D	—	1.07	1.12	—	1.58	Ar flow at 328 K	456 [511]	—	[505]
**JNU‐9‐CH_3_**, 3D	9.5	2.96	2.92	31.1/–	1.5	Vacuum at 298 K	716	Van der Waals interactions	[512]
**CPM‐734c**, 3D	9.3^b^	8.73	9.05	31.5/30.8	1.44	—	1944	Pore size, van der Waals interactions	[513]
**HIAM‐402**, 3D	8–12	∼5.8–6.25	∼5.8–6.25	34.5/31.2	1.43	—	1442	H‐bonding	[514]
**ZIF‐8**, 3D	3.4 × 3.4, 12 × 12	4.4	4.5	—	1.4	—	1844	—	[366]
**BUT‐10**, 3D	10^b^	6.25	6.43	32.7/30	1.4	—	1726	H‐bonding, C‐H⋅⋅⋅π interactions	[515]
**g‐C_3_N_4_@Zr‐BPDC**, 3D	10.9^b^	8.9	8.9	29/27.5	1.4	—	2409	Van der Waals interactions	[510]
**Zr‐bpy**, 3D	11.2^b^	8.21	8.46	32/–	1.25	—	1605	—	[515]
**CPM‐736t**, 3D	10.9^b^	10.9	11.5	25.2/24.7	1.25	—	2087	Pore size, van der Waals interactions	[513]
**Zr‐BPDC**, 3D	10.9^b^	8.8	8.4	47/46	1.2	—	2094	Van der Waals interactions	[510]
**FDMOF‐1**, 3D	6.9^b^	5.45	5.31	28.5/27.1	1.19	298 K	1176	—	[507]
**UiO‐67**, 3D	11.2^b^	9.38	9.8	36.3/–	1.09	—	1775	—	[515]
**Zn‐DMOF**, 3D	7.5^b^	6.79	7.23	24.7/23.1	1.06	298 K	1880	—	[507]
**CPM‐734t**, 3D	8.6^b^	8.4	9.33	30.6/28.7	0.99	—	1727	Pore size, van der Waals interactions	[513]
**ZIF‐67**, 3D	3.3 × 3.3	3.5	3.5	—	—	He flow at 298 K	1500	Flexibility	[505, 516]
**ZU‐609**, 3D	4.2 × 5.1	2.34	0.13	43/–	—	N_2_ flow at 298 K	380	Molecular sieving	[80]

^a^ For v/v = 1/99 equimolar mixture, predicted by IAST.

^b^ Pore size, BET surface area calculated from the N_2_ sorption isotherm at 77K.

**TABLE 13:** C1‐C3 multicomponent gas mixtures separations in reticular sorbents. The following parameters are listed for comparison: pore size; BET surface area (S_BET_); single‐component gas uptakes; adsorption enthalpies (*Q*_st_); adsorption selectivity; regeneration temperature; and attributed mechanisms.

Adsorbent, network dimensionality (nD)	Pore size (Å)	Gas uptake at 1 bar (mmolg^−1^)	*Q*_st_ (kJmol^−1^)	Components	Selectivity^a^	Regeneration temperature (K)	S_BET_ (m^2^g^−1^)	Mechanism	Refs.
**TIFSIX‐2‐Cu‐i**, 3D	5.1 × 5.1	CO_2_: 4.27 C_2_H_2_: 4.38 C_2_H_4_: 2.75 C_2_H_6_: 2.2	CO_2_: 35.8 C_2_H_2_: 46.3 C_2_H_4_: 35.9 C_2_H_6_: 34.5	C_2_H_2_/C_2_H_4_ (1/1)	48.8	He flow at 333 K	685	H‐bonding	[70]
				C_2_H_2_/C_2_H_6_ (1/1)	97.8				
				C_2_H_2_/CO_2_ (1/1)	6.1				
**SIFSIX‐3‐Ni**, 3D	4.2 × 4.2	CO_2_: 2.7 C_2_H_2_: 3.59 C_2_H_4_: 1.98 C_2_H_6_: 1.53	CO_2_: 50.9 C_2_H_2_: 36.7 C_2_H_4_: 31.7 C_2_H_6_: 23.7	CO_2_/C_2_H_2_ (1/1)	6.9	He flow at 333 K	230	Electrostatics	[70]
				CO_2_/C_2_H_4_ (1/1)	103				
				CO_2_/C_2_H_6_ (1/1)	308				
**Zn‐atz‐ipa**, 3D	5.6 × 5.6	CO_2_: 1.90 C_2_H_2_: 1.99 C_2_H_4_: 1.80 C_2_H_6_: 1.81	CO_2_: 31.5 C_2_H_2_: 37.5 C_2_H_4_: 40.0 C_2_H_6_: 45.8	C_2_H_6_/C_2_H_2_ (1/1)	2	He flow at 333 K	650	H‐bonding	[70]
				C_2_H_6_/C_2_H_4_ (1/1)	1.7				
				C_2_H_6_/CO_2_ (1/1)	5				
**MCOF‐1**, 3D	6.4	CH_4_: 0.40 C_2_H_4_: 1.61 C_2_H_6_: 1.96 C_3_H_8_: 2.46	15 19 41 –	C_2_H_4_/CH_4_ (1/1)	26	—	874	Pore size	[517]
				C_2_H_6_/CH_4_ (1/1)	88				
				C_3_H_8_/CH_4_ (1/1)	1800				
**UTSA‐34b**, 3D	12.8	C_2_H_2_: 5.36 CH_4_: 0.89 C_2_H_4_: 4.24 C_2_H_6_: 4.24	C_2_H_2_: 49 CH_4_: 20 C_2_H_4_: 31 C_2_H_6_: 31	C_2_H_2_/CH_4_ (1/1)	24	—	991.4	UMCs	[425]
				C_2_H_4_/CH_4_ (1/1)	17				
				C_2_H_6_/CH_4_ (1/1)	17				
**UTSA‐35a**, 3D	7.7 × 5.8	CH_4_: 0.31 C_2_H_2_: 3.85 C_2_H_4_: 2.71 C_2_H_6_: 3.26 C_3_H_6_: 6.18 C_3_H_8_: 5.84	17.2 29.4 28.0 30.0 33 41.8	C_3_H_6_/CH_4_ (1/1)	80	—	742.7	Van der Waals interactions and pore size	[411]
				C_3_H_8_/CH_4_ (1/1)	75				
				C_2_H_2_/CH_4_ (1/1)	21				
				C_2_H_4_/CH_4_ (1/1)	8				
				C_2_H_6_/CH_4_ (1/1)	15				
**[La(BTB)H_2_O]**, 3D	10	(At 10kPa) CH_4_: 0.31 CO_2_: 3.85 C_2_H_4_: 2.71 C_2_H_6_: 3.26	(At 10kPa) 0.38 8.9 6.6 6.2	CO_2_/CH_4_ (1/1)	—	—	1024	UMCs	[518]
				C_2_H_4_/CH_4_ (1/1)	12 (273K)				
				C_2_H_6_/CH_4_ (1/1)	22 (273K)				
**TJT‐100**, 3D	8.7 × 11.6	C_2_H_4_: 3.4 C_2_H_6_: 3.66	C_2_H_2_: 31 C_2_H_4_: 29 C_2_H_6_: 25	C_2_H_6_/C_2_H_4_ (1/99)	1.2	He flow at 423 K	890	H‐bonding and van der Waals interactions	[422]
				C_2_H_2_/C_2_H_4_ (1/99)	1.8				
**FNU‐2**, 3D	6, 8, 12^b^	CH_4_: 0.59 C_2_H_6_: 1.58 C_3_H_8_: 1.51	CH_4_: 6 C_2_H_6_: 10.5 C_3_H_8_: 12.4	C_3_H_8_/CH_4_ (5/85)	638.9	—	193.3	Van der Waals interactions	[519]
				C_2_H_6_/CH_4_ (10/85)	43.9				
**ZNU‐6**, 3D	8.22, 10.76	CO_2_: 4.76 C_2_H_2_: 8.06 C_2_H_4_: 4.76	CO_2_: 37.2 C_2_H_2_: 37.1 C_2_H_4_: 29	C_2_H_2_/C_2_H_4_	8.19	Ar flow at 393 K	1330.3	π···π interactions	[520]
				CO_2_/C_2_H_4_	7.84				
**NKCOF‐62**, 2D	8 × 8	C_2_H_2_: 2.54 C_2_H_4_: 1.88 C_2_H_6_: 2.1	C_2_H_2_: 30.5 C_2_H_4_: 26.3 C_2_H_6_: 29.6	C_2_H_2_/C_2_H_4_ (1/1)	1.3	—	714	Pore size, noncovalent interactions	[413]
				C_2_H_6_/C_2_H_4_ (1/1)	1.37				
**MOF‐303**, 3D	5.8^c^	C_2_H_2_: 7.91 C_2_H_6_: 5.01	C_2_H_2_: 31.7 C_2_H_4_: 24.3 C_2_H_6_: 25.1	C_2_H_2_/C_2_H_4_ (1/99)	2.4	—	1244	H‐bonding, C‐H···π interactions	[521]
				C_2_H_6_/C_2_H_4_ (1/1)	1.7				
**FJI‐W‐66a**, 3D	5.2 × 4.4	C_2_H_2_: 1.88 C_2_H_4_: 1.29 C_2_H_6_: 1.32	C_2_H_2_: 42.36 C_2_H_4_: 38.49 C_2_H_6_: 40.48	C_2_H_2_/C_2_H_4_ (1/99)	2.31	—	329	Flexibility	[522]
				C_2_H_6_/C_2_H_4_ (1/99)	1.4				
**Zn‐fa‐atz**, 3D	5.5 × 4.9	CO_2_: 2.8 C_2_H_2_: 2.65 C_2_H_4_: 2.1 C_2_H_6_: 2.05	CO_2_: 30.2 C_2_H_2_: 30.6 C_2_H_4_: 29.3 C_2_H_6_: 35.9	CO_2_/C_2_H_4_ (1/1)	1.4	—	—	H‐bonding	[523]
				C_2_H_2_/C_2_H_4_ (1/1)	1.5				
				C_2_H_6_/C_2_H_4_ (1/1)	1.4				
**NTUniv‐58**, 3D (308 K)	4‐6^b^	C_2_H_2_: 1.85 C_2_H_4_: 1.52 C_2_H_6_: 1.43	C_2_H_2_: 35.1 C_2_H_4_: 33.9	C_2_H_2_/C_2_H_4_ (1/99)	6.2	—	442	H‐bonding, π···π interactions	[524]
**NTUniv‐59**, 3D (308 K)	4‐6^b^	C_2_H_2_: 1.85 C_2_H_4_: 1.43 C_2_H_6_: 1.46	C_2_H_2_: 39 C_2_H_4_: 32	C_2_H_2_/C_2_H_4_ (1/99)	17.2	—	258	H‐bonding, π···π interactions	[524]
**LIFM‐XYY‐1**, 3D	—	C_2_H_2_: 1.85 C_2_H_4_: 1.43 C_2_H_6_: 1.46	C_2_H_2_: 29.6 C_2_H_4_: 28.8 C_2_H_6_: 29.5	C_2_H_6_/C_2_H_4_ (1/1)	1.51	He flow at 358 K	1343	H‐bonding, C‐H···π interactions	[383]
				C_2_H_2_/C_2_H_4_ (1/99)	1.07				
**LIFM‐XYY‐2**, 3D	—	C_2_H_2_: 3.26 C_2_H_4_: 2.92 C_2_H_6_: 3.65	C_2_H_2_: 33.1 C_2_H_4_: 28.8 C_2_H_6_: 29.5	C_2_H_6_/C_2_H_4_ (1/1)	1.48	He flow at 358 K	1724	H‐bonding, C‐H···π interactions	[383]
				C_2_H_2_/C_2_H_4_ (1/99)	1.51				
**LIFM‐XYY‐3**, 3D	—	C_2_H_2_: 2.73 C_2_H_4_: 2.10 C_2_H_6_: 2.53	C_2_H_2_: 31.0 C_2_H_4_: 28.5 C_2_H_6_: 29.6	C_2_H_6_/C_2_H_4_ (1/1)	1.33	He flow at 358 K	1274	H‐bonding, C‐H···π interactions	[383]
				C_2_H_2_/C_2_H_4_ (1/99)	1.42				
**LIFM‐XYY‐4**, 3D	—	C_2_H_2_: 3.54 C_2_H_4_: 2.74 C_2_H_6_: 3.89	C_2_H_2_: 27.2 C_2_H_4_: 25.0 C_2_H_6_: 29.3	C_2_H_6_/C_2_H_4_ (1/1)	1.68	He flow at 358 K	1743	H‐bonding, C‐H···π interactions	[383]
				C_2_H_2_/C_2_H_4_ (1/99)	1.41				
**LIFM‐XYY‐5**, 3D	—	C_2_H_2_: 3.07 C_2_H_4_: 2.56 C_2_H_6_: 3.39	C_2_H_2_: 25.9 C_2_H_4_: 18.3 C_2_H_6_: 26.7	C_2_H_6_/C_2_H_4_ (1/1)	1.53	He flow at 358 K	1694	H‐bonding, C‐H···π interactions	[383]
				C_2_H_2_/C_2_H_4_ (1/99)	1.31				
**LIFM‐XYY‐6**, 3D	—	C_2_H_2_: 2.97 C_2_H_4_: 2.24 C_2_H_6_: 2.99	C_2_H_2_: 37.1 C_2_H_4_: 31.5 C_2_H_6_: 32.2	C_2_H_6_/C_2_H_4_ (1/1)	1.63	He flow at 358 K	1240	H‐bonding, C‐H···π interactions	[383]
				C_2_H_2_/C_2_H_4_ (1/99)	1.53				
**LIFM‐XYY‐7**, 3D	—	C_2_H_2_: 4.86 C_2_H_4_: 4.07 C_2_H_6_: 5.23	C_2_H_2_: 22.3 C_2_H_4_: 23.0 C_2_H_6_: 24.9	C_2_H_6_/C_2_H_4_ (1/1)	1.50	He flow at 358 K	2079	H‐bonding, C‐H···π interactions	[383]
				C_2_H_2_/C_2_H_4_ (1/99)	1.24				
**LIFM‐XYY‐8**, 3D	—	C_2_H_2_: 3.81 C_2_H_4_: 3.30 C_2_H_6_: 4.21	C_2_H_2_: 25.9 C_2_H_4_: 26.7 C_2_H_6_: 27.4	C_2_H_6_/C_2_H_4_ (1/1)	1.47	He flow at 358 K	2104	H‐bonding, C‐H···π interactions	[383]
				C_2_H_2_/C_2_H_4_ (1/99)	1.14				
**LIFM‐28**, 3D	11.1 × 11.1	C_2_H_2_: 0.87 C_2_H_4_: 0.78 C_2_H_6_: 0.97	C_2_H_2_: 26.6 C_2_H_4_: 26.0 C_2_H_6_: 28.8	C_2_H_6_/C_2_H_4_ (1/1)	1.24	He flow at 358 K	862	—	[383]
				C_2_H_2_/C_2_H_4_ (1/99)	1.04				
**PCN‐700**, 3D	5.6 × 5.6	C_2_H_2_: 0.94 C_2_H_4_: 0.87 C_2_H_6_: 1.28	C_2_H_2_: 25.6 C_2_H_4_: 23.6 C_2_H_6_: 26.3	C_2_H_6_/C_2_H_4_ (1/1)	1.53	He flow at 358 K	849	—	[383]
				C_2_H_2_/C_2_H_4_ (1/99)	1.07				
**NPU‐1**, 3D	7.4^c^	C_2_H_2_: 5.09 C_2_H_4_: 4.2 C_2_H_6_: 4.5	C_2_H_2_: 27.8 C_2_H_4_: 23.9 C_2_H_6_: 29.1	C_2_H_6_/C_2_H_4_ (1/1)	1.32	He flow at 313 K	1580	H‐bonding	[321]
				C_2_H_2_/C_2_H_4_ (1/99)	1.40				
**Azole‐Th‐1**, 3D	9.2^c^	C_2_H_2_: 3.63 C_2_H_4_: 3.62 C_2_H_6_: 4.47	C_2_H_4_: 26.1 C_2_H_6_: 28.6	C_2_H_6_/C_2_H_4_ (1/1)	1.46	He flow at 298 K	983	C‐H···π interactions	[406]
				C_2_H_2_/C_2_H_4_ (1/99)	1.00				
**UiO‐67(NH_2_)_2_**, 3D	5.2 × 5.2, 7.1 × 7.1, 11.5 × 11.5	C_2_H_2_: 5.9 C_2_H_4_: 4.32 C_2_H_6_: 5.32	C_2_H_2_: 27.4 C_2_H_4_: 24.5 C_2_H_6_: 26.5	C_2_H_6_/C_2_H_4_ (1/1)	1.70	—	2815	H‐bonding, C‐H···π interactions	[376]
				C_2_H_2_/C_2_H_4_ (1/99)	2.10				
**UPC‐612**, 3D	14, 20^c^	C_2_H_2_: 3.0 C_2_H_4_: 2.92 C_2_H_6_: 3.58	C_2_H_2_: 23.94 C_2_H_4_: 16.94 C_2_H_6_: 22.39	C_2_H_6_/C_2_H_4_ (1/1)	1.40	—	2016	—	[401]
				C_2_H_2_/C_2_H_4_ (1/99)	1.08				
**UPC‐613**, 3D	7, 12^c^	C_2_H_2_: 2.83 C_2_H_4_: 2.31 C_2_H_6_: 2.55	C_2_H_2_: 30.4 C_2_H_4_: 28.5 C_2_H_6_: 31.8	C_2_H_6_/C_2_H_4_ (1/1)	1.47	—	853	—	[401]
				C_2_H_2_/C_2_H_4_ (1/99)	1.38				
**NUM‐9a**, 3D	4.5 × 4.5	C_2_H_2_: 2.33 C_2_H_4_: 2.23 C_2_H_6_: 2.48	C_2_H_2_: 35.8 C_2_H_4_: 32.3 C_2_H_6_: 35.8	C_2_H_6_/C_2_H_4_ (10/90)	1.62	—	330	H‐bonding, C‐H···π interactions	[385]
				C_2_H_2_/C_2_H_4_ (1/99)	1.48				
**Zn(ad)(int)**, 3D	8^c^	C_2_H_2_: 3.04 C_2_H_4_: 2.21 C_2_H_6_: 2.32	C_2_H_2_: 34.7 C_2_H_4_: 29 C_2_H_6_: 33.3	C_2_H_6_/C_2_H_4_ (1/1)	2.4	—	617	H‐bonding, π···π, C‐H···π interactions	[331]
				C_2_H_2_/C_2_H_4_ (1/99)	1.61				
**CuTiF6‐TPPY**, 3D	5 × 5, 8 × 8	C_2_H_2_: 3.62 C_2_H_4_: 2.42 C_2_H_6_: 2.82	C_2_H_2_: 36.5 C_2_H_4_: 29.6 C_2_H_6_: 34.2	C_2_H_6_/C_2_H_4_ (1/1)	2.12	He flow a 298 K	685	H‐bonding, C‐H···π, van der Waals interactions	[338]
				C_2_H_2_/C_2_H_4_ (1/99)	5.03				
**UPC‐66‐a**, 3D (293 K)	6.01 × 13.84, 7.04	C_2_H_2_: 3.18 C_2_H_4_: 2.41 C_2_H_6_: 2.76	C_2_H_2_: 17.6 C_2_H_4_: 17.8 C_2_H_6_: 15.6	C_2_H_6_/C_2_H_4_ (1/1)	1.65	—	483	C‐H···π interactions	[381]
				C_2_H_2_/C_2_H_4_ (1/1)	1.05				
**[Zn(BDC)(H_2_BPZ)]*_n_***, 3D	8‐10.2	C_2_H_2_: 4.46 C_2_H_4_: 2.59 C_2_H_6_: 3.63	C_2_H_2_: 28.7 C_2_H_4_: 23.2 C_2_H_6_: 31.8	C_2_H_6_/C_2_H_4_ (1/1)	2.20	—	906.5	H‐bonding, C‐H···π interactions	[336]
				C_2_H_2_/C_2_H_4_ (1/99)	1.60				
**Zn‐atz‐oba**, 3D	3.2, 4.4	C_2_H_2_: 2.77 C_2_H_4_: 2.02 C_2_H_6_: 2.04	C_2_H_2_: 27.5 C_2_H_4_: 27 C_2_H_6_: 30	C_2_H_6_/C_2_H_4_ (1/1)	1.27	He flow at 333 K	710.7	H‐bonding	[419]
				C_2_H_2_/C_2_H_4_ (1/99)	1.43				
**Zn‐ATA**, 3D	4.1 × 3.8	C_2_H_2_: 2 C_2_H_4_: 1.20 C_2_H_6_: 1.08	C_2_H_2_: 24.5 C_2_H_4_: 27.4 C_2_H_6_: 32.5	C_2_H_6_/C_2_H_4_ (1/1)	1.84	N_2_ flow at 373 K	344	H‐bonding, C‐H···π interactions	[363]
				C_2_H_2_/C_2_H_4_ (1/99)	1.81				
**ZJNU‐7**, 3D	5.0, 5.9, 7.3	C_2_H_2_: 5.04 C_2_H_4_: 3.8 C_2_H_6_: 4.13	C_2_H_2_: 34.1 C_2_H_4_: 29.3 C_2_H_6_: 29.7	C_2_H_6_/C_2_H_4_ (1/1)	1.56	—	1180	H‐bonding, C‐H···π interactions, π‐complexion	[397]
				C_2_H_2_/C_2_H_4_ (1/99)	2.31				
**ZJNU‐115**, 3D	8.58	C_2_H_2_: 4.73 C_2_H_4_: 3.75 C_2_H_6_: 4.21	C_2_H_2_: 29.2 C_2_H_4_: 27.7 C_2_H_6_: 28.2	C_2_H_6_/C_2_H_4_ (1/1)	1.56	—	1291	C‐H···π interactions, π‐complexion	[396]
				C_2_H_2_/C_2_H_4_ (1/99)	2.05				
**BFFOUR‐Cu‐dpds**, 2D	3.9 × 4.0, 3.3 × 3.8	C_2_H_4_: 1.31 C_2_H_6_: 0.1 C_3_H_6_: 1.23 C_3_H_8_: 0.09 *n*‐C_4_H_8_: 0.96 *n*‐C_4_H_10_: 0.09	C_2_H_4_: 34.44 C_3_H_6_: 31.13 *n*‐C_4_H_8_: 33.37	C_2_H_4_/C_2_H_6_ (1/1)	68.8	He flow at 333 K	140	H‐bonding, C‐H···π interactions	[525]
				C_3_H_6_/C_3_H_8_ (1/1)	108.4				
				*n*‐C_4_H_8_/*n*‐C_4_H_10_ (1/1)	22.9				
**NTUniv‐63**, 3D	3.4 × 3.4, 4.4 × 4.4	C_2_H_2_: 2.73 C_2_H_4_: 2.07 C_2_H_6_: 2.05	C_2_H_2_: 34.2 C_2_H_4_: 32.7 C_2_H_6_: 33.9	C_2_H_2_/C_2_H_4_ (1/99)	7.3	—	518	H‐bonding, N‐H···π interactions	[526]
				C_2_H_6_/C_2_H_4_ (1/99)	1.7				
**FJI‐Y9**, 3D	9‐12^c^	C_2_H_2_: 4.51 C_2_H_4_: 3.93 C_2_H_6_: 4.69 C_3_H_6_: 5.85	C_2_H_2_: 27.2 C_2_H_4_: 23.7 C_2_H_6_: 24.5 C_3_H_6_: 28.7	C_2_H_2_/C_2_H_4_ (1/99)	3.8	—	1420	H‐bonding, C‐H···π interactions	[527]
				C_2_H_6_/C_2_H_4_ (10/90)	1.79				
**Zr‐TCA**, 3D	6 × 7	C_2_H_2_: 2.78 C_2_H_4_: 2.02 C_2_H_6_: 2.28	C_2_H_2_: 43.8 C_2_H_4_: 23.9 C_2_H_6_: 35.3	C_2_H_2_/C_2_H_4_ (1/99)	5.64	—	350.9	H‐bonding, C‐H···π, polarizability	[528]
				C_2_H_6_/C_2_H_4_ (10/90)	2.72				
**CAU‐23**, 3D	6.3 × 7.6	C_2_H_2_: 4.7 C_2_H_4_: 3.8 C_2_H_6_: 4.0	C_2_H_2_: 32.8 C_2_H_4_: 25.4 C_2_H_6_: 28.4	C_2_H_2_/C_2_H_4_ (1/15)	1.50	He flow at 298 K	1240	Van der Waals interactions	[529]
				C_2_H_6_/C_2_H_4_ (1/1)	1.54				
**3D‐IL‐COF‐1**, 3D	8.3	—	—	CO_2_/N_2_ (1/1)	24.6	—	517	—	[139]
				CO_2_/CH_4_ (1/1)	23.1				
**3D‐IL‐COF‐2**, 3D	10.7	—	—	CO_2_/N_2_ (1/1)	24.0	—	653	—	[139]
				CO_2_/CH_4_ (1/1)	22.3				
**3D‐IL‐COF‐3**, 3D	12.4	—	—	CO_2_/N_2_ (1/1)	24.4	—	870	—	[139]
				CO_2_/CH_4_ (1/1)	21.5				
**3D‐COF‐1a**, 3D	—	—	—	CO_2_/N_2_ (1/1)	7.1	—	596	—	[139]
				CO_2_/CH_4_ (1/1)	5.3				
**3D‐IL‐COF‐1b**, 3D	—	—	—	CO_2_/N_2_ (1/1)	43.6	—	537	—	[139]
				CO_2_/CH_4_ (1/1)	35.1				
**TpTa‐NO_2_**, 2D	15 × 15	CO_2_: 2.02 C_2_H_2_: 2.85 C_2_H_4_: 1.4	CO_2_: 36.26 C_2_H_2_: 42.57 C_2_H_4_: 33.07	CO_2_/N_2_ (15/85)	125.23	—	398	H‐bonding, van der Waals interactions	[530]
				C_2_H_2_/C_2_H_4_ (1/99)	37.96				
				C_2_H_2_/CO_2_ (10/90)	10.58				
				CO_2_/C_2_H_4_ (10/90)	2.45				
**CMOM‐7**, 3D	5.3^b^, 8.9^c^	CH_4_: 0.68 C_2_H_6_: 3.10 C_3_H_8_: 3.40	CH_4_: 20.9 C_2_H_6_: 27.9 C_3_H_8_: 31.2	C_3_H_8_/C_2_H_6_ (1/1)	13.3	He flow at 298 K	861.38	H‐bond, C‐H···π interactions	[531]
				C_2_H_6_/CH_4_ (1/1)	12.5				
				C_3_H_8_/CH_4_ (1/1)	40.1				
**GNU‐1**, 3D	2.3 × 11.2	CH_4_: 1.12 C_2_H_6_: 4.6 C_3_H_8_: 6.64	CH_4_: 14.7 C_2_H_6_: 29.2 C_3_H_8_: 35.5	C_3_H_8_/CH_4_ (5/85)	330.1	—	1297	H‐bonding, electrostatics, C‐H···π interactions	[532]
				C_2_H_6_/CH_4_ (10/85)	17.5				
**MOF‐303**, 3D	5‐7	CH_4_: 0.86 C_2_H_6_: 4.96 C_3_H_8_: 4.74	CH_4_: 19 C_2_H_6_: 24 C_3_H_8_: 34	C_3_H_8_/CH_4_ (5/85)	5114	N_2_ flow at 333 K	1220	H‐bonding, pore size	[533]
				C_2_H_6_/CH_4_ (10/85)	26				
**MIL‐160**, 3D	5‐7	CH_4_: 0.94 C_2_H_6_: 4.65 C_3_H_8_: 5.08	CH_4_: 19 C_2_H_6_: 28 C_3_H_8_: 35	C_3_H_8_/CH_4_ (5/85)	174	N_2_ flow at 333 K	1188	H‐bonding, pore size	[533]
				C_2_H_6_/CH_4_ (10/85)	20				
**UiO‐66**, 3D	8.6, 11.1	CH_4_: 0.60 C_2_H_6_: 1.67 C_3_H_8_: 1.79	C_2_H_6_: 26.76 C_3_H_8_: 32.69	C_3_H_8_/CH_4_ (1/1)	65	He flow at 373 K	1305	C‐H···π interactions	[534]
				C_2_H_6_/CH_4_ (1/1)	8				
**UiO‐66‐Naph**, 3D	5.5, 10.5	CH_4_: 0.38 C_2_H_6_: 1.24 C_3_H_8_: 1.39	C_2_H_6_: 28.29 C_3_H_8_: 37.92	C_3_H_8_/CH_4_ (1/1)	741	He flow at 373 K	881	C‐H···π interactions	[534]
				C_2_H_6_/CH_4_ (1/1)	32				
**DUT‐52**, 3D	10.3, 17.3	CH_4_: 0.54 C_2_H_6_: 1.89 C_3_H_8_: 2.21	C_2_H_6_: 24.57 C_3_H_8_: 28.89	C_3_H_8_/CH_4_ (1/1)	48	He flow at 373 K	1641	C‐H···π interactions	[534]
				C_2_H_6_/CH_4_ (1/1)	5				
**UiO‐66‐Anth**, 3D	5.7, 10.1	CH_4_: 0.21 C_2_H_6_: 0.70 C_3_H_8_: 0.90	C_2_H_6_: 28.95 C_3_H_8_: 35.16	C_3_H_8_/CH_4_ (1/1)	535	He flow at 373 K	676	C‐H···π interactions	[534]
				C_2_H_6_/CH_4_ (1/1)	32				
**CFA‐1**, 3D	10‐12^c^	CH_4_: 0.21 C_2_H_6_: 0.70 C_3_H_8_: 0.90	CH_4_: 18.3 C_2_H_6_: 29.3 C_3_H_8_: 33.1	C_3_H_8_/CH_4_ (5/85)	11.7	—	1903.3	Polarizability, H‐bonding	[535]
				C_2_H_6_/CH_4_ (10/85)	225.1				
**CFA‐1‐NiCl_2_‐2.3**, 3D	10‐12^c^	CH_4_: 0.21 C_2_H_6_: 0.70 C_3_H_8_: 0.90	CH_4_: 16.7 C_2_H_6_: 28.0 C_3_H_8_: 32.3	C_3_H_8_/CH_4_ (5/85)	15.2	—	2090.7	Polarizability, H‐bonding	[535]
				C_2_H_6_/CH_4_ (10/85)	382.7				
**Ni‐FDMOF**, 3D	7.2^c^	CH_4_: 0.67 C_3_H_6_: 8.08 C_3_H_8_: 8.08	CH_4_: 16.6 C_3_H_6_: 32.8 C_3_H_8_: 35.2	C_3_H_8_/CH_4_	123.8	423 K	1110	H‐bonding	[536]
				C_3_H_6_/CH_4_	79.5				
**Zn‐BPZ‐SA**, 3D	6.4‐8.4	C_2_H_4_: 2.85 C_3_H_6_: 3.05 C_2_H_6_: 2.97 C_3_H_8_: 2.73	C_2_H_4_: 23.13 C_3_H_6_: 33.65 C_2_H_6_: 26.4 C_3_H_8_: 32.7	C_3_H_6_/C_2_H_4_ (1/1)	4.8	—	925	H‐bonding, C‐H···π, π···π interactions	[537]
				C_3_H_8_/C_2_H_6_ (1/1)	4.3				
**Fe‐pyz**, 3D	3.8 × 3.2	CH_4_: 0.75 C_2_H_6_: 1.67 C_3_H_8_: 2.75	CH_4_: 16 C_2_H_6_: 52 C_3_H_8_: 32	C_3_H_8_/CH_4_ (5/85)	89	—	361	H‐bonding, van der Waals interactions	[538]
				C_2_H_6_/CH_4_ (10/85)	23				
**Co‐pyz**, 3D	3.9 × 3.3	CH_4_: 1.00 C_2_H_6_: 1.67 C_3_H_8_: 1.63	CH_4_: 20 C_2_H_6_: 41 C_3_H_8_: 50	C_3_H_8_/CH_4_ (5/85)	78	—	458	H‐bonding, van der Waals interactions	[538]
				C_2_H_6_/CH_4_ (10/85)	22				
**Ni‐pyz**, 3D	3.8 × 3.9	CH_4_: 2.94 C_2_H_6_: 3.13 C_3_H_8_: 3.31	CH_4_: 23 C_2_H_6_: 38 C_3_H_8_: 70	C_3_H_8_/CH_4_ (5/85)	53	—	572	H‐bonding, van der Waals interactions	[538]
				C_2_H_6_/CH_4_ (10/85)	18				
**Zn(bdc)(ted)_0.5_**, 3D	7.5 × 7.5, 4.8 × 3.2	CH_4_: 0.70 C_2_H_6_: 4.9 C_3_H_8_: 6.6 C_4_H_10_: 6.9	CH_4_: 19.8 C_2_H_6_: 21.3 C_3_H_8_: 24.2 C_4_H_10_: 29.6	C_4_H_10_/CH_4_ (1/1)	180	—	1904	Van der Waals interactions	[539]
				C_3_H_8_/CH_4_ (1/1)	67				
				C_2_H_6_/CH_4_ (1/1)	13				
**TIFSIX‐Cu‐TPA**, 3D	2.4 × 2.4	CH_4_: 0.68 C_2_H_6_: 4.4 C_3_H_8_: 4.91	CH_4_: 18.5 C_2_H_6_: 27.3 C_3_H_8_: 29.4	C_3_H_8_/CH_4_ (1/1)	68.6	Ar flow at 373 K	—	Van der Waals interactions	[540]
				C_2_H_6_/CH_4_ (1/1)	16.2				
**Co‐MOF**, 3D	4.3 × 4.3	CH_4_: 0.74 C_2_H_6_: 2.62 C_3_H_8_: 2.65	CH_4_: 29.67 C_2_H_6_: 37.17 C_3_H_8_: 38.21	C_3_H_8_/CH_4_ (1/1)	290	He flow at 373 K	345.8	H‐bonding, C‐H···π, van der Waals interactions	[541]
				C_2_H_6_/CH_4_ (1/1)	26				
**SU‐100**, 3D	7.1 × 7.6	CH_4_: 0.66 CO_2_: 2.24 C_2_H_2_: 2.63 C_2_H_6_: 1.69	CH_4_: 23.3 CO_2_: 32.3 C_2_H_2_: 41.7 C_2_H_6_: 33.2	C_2_H_2_/CH_4_ (1/1)	48.2	He flow at 343 K	388	H‐bonding, C‐H···π, π···π interactions	[542]
				C_2_H_6_/CH_4_ (1/1)	39.3				
				CO_2_/CH_4_ (1/1)	38.7				
**Cu‐IPA**, 3D	9.3^c^	CH_4_: 0.81 C_2_H_6_: 2.57 C_3_H_8_: 3.10	CH_4_: 11.4 C_2_H_6_: 39.3 C_3_H_8_: 43.9	C_3_H_8_/CH_4_ (5/85)	296	373 K	640.2	Van der Waals interactions	[543]
				C_2_H_6_/CH_4_ (10/85)	35				
**Cd(II)‐MOF**, 3D	4.2^c^	CH_4_: 0.73 C_2_H_6_: 3.19 C_3_H_8_: 3.02	CH_4_: 27.7 C_2_H_6_: 33.3 C_3_H_8_: 50.5	C_3_H_8_/CH_4_ (5/85)	233.8	—	327.9	H‐bonding, van der Waals interactions	[544]
				C_2_H_6_/CH_4_ (10/85)	34.3				
**(Me_2_NH_2_) [Ni_3_(*μ*_3_‐OH)** **(CF_3_‐BPDC‐CF_3_)_3_(tpt)]*_n_***, 3D	11.5 × 11.5, 9.3 × 9.3	CH_4_: 0.46 C_2_H_6_: 2.78 C_3_H_8_: 5.09	CH_4_: 7.74 C_2_H_6_: 20.2 C_3_H_8_: 29.2	CH_4_/C_3_H_8_ (85/5)	60.1	—	1495	H‐bonding, C‐H···π, interactions	[545]
				CH_4_/C_2_H_6_ (85/10)	7.1				
**C‐PVDC‐800**, 3D	5.2‐5.3	CH_4_: 1.54 C_2_H_6_: 5.29 C_3_H_8_: 5.17	CH_4_: 19.5 C_2_H_6_: 35.6 C_3_H_8_: 78.1	C_3_H_8_/CH_4_ (1/1)	3387	He flow at 423 K	1087	Kinetics	[546]
				C_2_H_6_/CH_4_ (1/1)	75				
**ANPC‐1‐800**, 3D	—	CH_4_: 1.45 C_2_H_6_: 6.84 C_3_H_8_: 9.74	CH_4_: 16.7 C_2_H_6_: 23.7 C_3_H_8_: 23.3	C_3_H_8_/CH_4_ (1/1)	110.4	Vacuum	2836	Van der Waals interactions	[547]
				C_2_H_6_/CH_4_ (1/1)	14.5				
**ANPC‐2‐700**, 3D	—	CH_4_: 1.12 C_2_H_6_: 4.88 C_3_H_8_: 8.80	CH_4_: 15.4 C_2_H_6_: 23.6 C_3_H_8_: 28.9	C_3_H_8_/CH_4_ (1/1)	162.5	Vacuum	2729	Van der Waals interactions	[547]
				C_2_H_6_/CH_4_ (1/1)	13.5				
**ANPC‐2‐800**, 3D	—	CH_4_: 1.15 C_2_H_6_: 4.94 C_3_H_8_: 11.5	CH_4_: 15.6 C_2_H_6_: 22.6 C_3_H_8_: 26.8	C_3_H_8_/CH_4_ (1/1)	120.2	Vacuum	3177	Van der Waals interactions	[547]
				C_2_H_6_/CH_4_ (1/1)	11.9				
**BSF‐1**, 3D	—	CH_4_: 0.66 C_2_H_6_: 1.17 C_3_H_8_: 0.99	CH_4_: 23.7 C_2_H_6_: 28.6 C_3_H_8_: 33.7	C_3_H_8_/CH_4_ (1/1)	353	—	535	Electrostatics, H‐bonding	[548]
				C_2_H_6_/CH_4_ (1/1)	23				
**FJI‐C4**, 3D	5.9 × 5.9	CH_4_: 1.15 C_2_H_6_: 2.21 C_3_H_8_: 1.63	CH_4_: 23.1 C_2_H_6_: 40.9 C_3_H_8_: 42.9	C_3_H_8_/CH_4_ (1/1)	293.4	—	690	Pore size, electrostatics, π···π interactions	[426]
				C_2_H_6_/CH_4_ (1/1)	39.7				
**FJI‐H22**, 3D	12.8 × 15.08, 6.05 × 11.83	CH_4_: 0.88 C_2_H_6_: 1.49 C_3_H_8_: 1.10	—	C_3_H_8_/CH_4_ (1/1)	145.23	—	483	—	[549]
				C_2_H_6_/CH_4_ (1/1)	11.95				
**JLU‐Liu5**, 3D	—	CH_4_: 1.00 C_2_H_6_: 2.37 C_3_H_8_: 1.59	CH_4_: 19 C_2_H_6_: 29.8 C_3_H_8_: 21.6	C_3_H_8_/CH_4_ (1/1)	107.8	—	707	UMCs	[550]
				C_2_H_6_/CH_4_ (1/1)	17.6				
**JLU‐Liu6**, 3D	—	CH_4_: 0.81 C_2_H_6_: 1.63 C_3_H_8_: 1.30	CH_4_: 24.9 C_2_H_6_: 46.5 C_3_H_8_: 12.3	C_3_H_8_/CH_4_ (1/1)	274.6	—	544	UMCs	[550]
				C_2_H_6_/CH_4_ (1/1)	20.4				
**JLU‐Liu7**, 3D	5.2 × 10.8	CH_4_: 1.06 C_2_H_6_: 3.57 C_3_H_8_: 2.57	CH_4_: 20.9 C_2_H_6_: 34.8 C_3_H_8_: 28.5	C_3_H_8_/CH_4_ (1/1)	128.5	—	879	—	[551]
				C_2_H_6_/CH_4_ (1/1)	50.4				
**JLU‐Liu38**, 3D	8.6‐11	CH_4_: 0.48 C_2_H_6_: 4.96 C_3_H_8_: 8.39	CH_4_: 17.5 C_3_H_8_: 29.2	C_3_H_8_/CH_4_ (1/1)	98	—	1784	Van der Waals interactions	[552]
				C_2_H_6_/CH_4_ (1/1)	12.5				
**JUC‐100**, 3D	14	CH_4_: 0.64 C_2_H_6_: 3.07 C_3_H_8_: 3.09	CH_4_: 27.1 C_2_H_6_: 26.1	C_3_H_8_/CH_4_ (1/1)	80	—	2040	—	[553]
				C_2_H_6_/CH_4_ (1/1)	11				
**JUC‐103**, 3D	10	CH_4_: 0.73 C_2_H_6_: 2.85 C_3_H_8_: 2.77	CH_4_: 23.5 C_2_H_6_: 22.6	C_3_H_8_/CH_4_ (1/1)	55	—	1484	—	[553]
				C_2_H_6_/CH_4_ (1/1)	8				
**JUC‐106**, 3D	8	CH_4_: 0.51 C_2_H_6_: 2.61 C_3_H_8_: 2.59	CH_4_: 26.1 C_2_H_6_: 24.2	C_3_H_8_/CH_4_ (1/1)	75	—	1122	—	[553]
				C_2_H_6_/CH_4_ (1/1)	13				
**UTSA‐35a**, 3D	3.4 × 3.8, 2.0 × 3.8, 3.1 × 4.4	CH_4_: 0.43 C_2_H_6_: 2.43 C_3_H_8_: 2.97	CH_4_: 24.4 C_2_H_6_: 36.1	C_3_H_8_/CH_4_ (1/1)	80	—	806	Molecular sieving	[554]
				C_2_H_6_/CH_4_ (1/1)	20				
**MFM‐202a**, 3D	9 × 9	CH_4_: 0.45 C_2_H_6_: 4.21 C_3_H_8_: 6.76	CH_4_: 19 C_2_H_6_: 21 C_3_H_8_: 39	C_3_H_8_/CH_4_ (1/1)	87	—	2220	—	[555]
				C_2_H_6_/CH_4_ (1/1)	10				
**InOF‐1**, 3D	7.4 × 7.4	CH_4_: 0.64 C_2_H_6_: 4.14 C_3_H_8_: 4.25	CH_4_: 18.8 C_2_H_6_: 25.1 C_3_H_8_: 31.4	C_3_H_8_/CH_4_ (1/1)	90	—	1109	Electrostatics	[556]
				C_2_H_6_/CH_4_ (1/1)	17				
**RT‐MIL‐100(Fe)**, 3D	—	CH_4_: 0.36 C_2_H_6_: 2.22 C_3_H_8_: 6.78	CH_4_: 19 C_2_H_6_: 23 C_3_H_8_: 26	C_3_H_8_/CH_4_ (5/85)	33.3	—	2482	H‐bonding	[557]
				C_2_H_6_/CH_4_ (10/85)	6				
**MIL‐101‐Cr**, 3D	10.5, 16.9, 32.2	CH_4_: 0.49 C_2_H_6_: 1.59 C_3_H_8_: 3.35	C_2_H_6_: 22.2 C_3_H_8_: 29.2	C_3_H_8_/CH_4_ (1/1)	84.3	He flow at 373 K	2961	H‐bonding, C‐H···π interactions	[558]
				C_2_H_6_/CH_4_ (1/1)	22.5				
**MIL‐101‐Fe**, 3D	11, 15.5, 32.2	CH_4_: 0.45 C_2_H_6_: 1.25 C_3_H_8_: 3.29	C_2_H_6_: 25.6 C_3_H_8_: 34.5	C_3_H_8_/CH_4_ (1/1)	24.9	He flow at 373 K	2617	H‐bonding, C‐H···π interactions	[558]
				C_2_H_6_/CH_4_ (1/1)	15.4				
**MIL‐101‐Fe‐NH_2_**, 3D	11, 16.8, 24.4, 31	CH_4_: 0.46 C_2_H_6_: 1.35 C_3_H_8_: 3.32	C_2_H_6_: 24.6 C_3_H_8_: 28.3	C_3_H_8_/CH_4_ (5/85)	42.5	He flow at 373 K	2648	H‐bonding, C‐H···π interactions	[558]
				C_2_H_6_/CH_4_ (10/85)	11.6				
**A‐AC‐3**, 3D	20‐40^c^	CH_4_: 1.38 C_2_H_6_: 7.09 C_3_H_8_: 11.34	—	C_3_H_8_/CH_4_ (1/4)	76.6	—	2928	—	[559]
				C_2_H_6_/CH_4_ (1/4)	16.9				
**A‐AC‐4**, 3D	20‐40^c^	CH_4_: 1.18 C_2_H_6_: 6.59 C_3_H_8_: 11.76	—	C_3_H_8_/CH_4_ (1/4)	88.8	—	3131	—	[559]
				C_2_H_6_/CH_4_ (1/4)	15.1				
**sPI‐A‐H**, 3D	3.5, 4.8^b^	CH_4_: 0.39 C_2_H_6_: 1.59 C_3_H_8_: 2.00	—	C_3_H_8_/CH_4_ (1/1)	66.7	—	665	Polarizability, π···π interactions	[560]
				C_2_H_6_/CH_4_ (1/1)	13.3				
**UiO‐67**, 3D	10.9, 13.58	CH_4_: 0.56 C_2_H_6_: 4.26 C_3_H_8_: 9.50	CH_4_: 22 C_2_H_6_: 32.3 C_3_H_8_: 47.5	C_3_H_8_/CH_4_ (5/85)	73.7	—	2591	—	[561]
				C_2_H_6_/CH_4_ (10/85)	8.1				
**GTGU‐15**, 3D	13.8, 20^c^	CH_4_: 0.40 C_2_H_6_: 2.13 C_3_H_8_: 12.13	CH_4_: 19 C_2_H_6_: 21.3 C_3_H_8_: 21.7	C_3_H_8_/CH_4_ (5/85)	32.61	Vacuum at 393 K	3164	H‐bonding	[562]
				C_2_H_6_/CH_4_ (10/85)	5.12				
**JLU‐Liu22**, 3D	6.8 × 6.8	CH_4_: 0.71 C_2_H_6_: 3.3 C_3_H_8_: 4.15	CH_4_: 11 C_2_H_6_: 30.5 C_3_H_8_: 30.3	C_3_H_8_/CH_4_ (5/85)	144.89	—	1487	UMCs, Polarizability	[563]
				C_2_H_6_/CH_4_ (10/85)	5.5				
**MIL‐142A**, 3D	7, 10	CH_4_: 0.54 C_2_H_6_: 3.82 C_3_H_8_: 5.32	CH_4_: 13.7 C_2_H_6_: 25.5 C_3_H_8_: 26.6	C_3_H_8_/CH_4_ (5/85)	85.51	Vacuum at 423 K	1425	Van der Waals interactions	[564]
				C_2_H_6_/CH_4_ (10/85)	8.6				
**Ni(TMBDC)(DABCO)_0.5_**, 3D	5^c^	C_2_H_6_: 5.81 C_3_H_8_: 5.54	CH_4_: 14 C_2_H_6_: 36 C_3_H_8_: 59	C_3_H_8_/CH_4_ (5/85)	274	N_2_ flow at 373 K	940	Van der Waals interactions	[565]
				C_2_H_6_/CH_4_ (10/85)	29				
**NKM‐101**, 3D	5.1 × 5.1	—	CH_4_: 17.2 C_2_H_6_: 25 C_3_H_8_: 23.8	C_3_H_8_/CH_4_ (5/85)	189.66	—	552	—	[566]
				C_2_H_6_/CH_4_ (10/85)	18.19				
**ZUL‐C2**, 3D	4.76 × 4.76	C_2_H_6_: 2.82 C_3_H_8_: 2.52	CH_4_: 71 C_2_H_6_: 45 C_3_H_8_: 5.32	C_3_H_8_/CH_4_ (5/85)	741	—	462	H‐bonding, van der Waals interactions	[567]
				C_2_H_6_/CH_4_ (10/85)	82				
**ZUL‐C1**, 3D	4.99 × 4.99	C_2_H_6_: 2.95 C_3_H_8_: 2.72	CH_4_: 54 C_2_H_6_: 33 C_3_H_8_: 23	C_3_H_8_/CH_4_ (5/85)	158	—	504	H‐bonding, van der Waals interactions	[567]
				C_2_H_6_/CH_4_ (10/85)	28				
**BSF‐2**, 3D	—	CH_4_: 0.48 C_2_H_6_: 1.22 C_3_H_8_: 1.77	CH_4_: 23.5 C_2_H_6_: 32.8 C_3_H_8_: 39.7	C_3_H_8_/CH_4_ (5/85)	681	He flow at 313 K	403	H‐bonding	[568]
				C_2_H_6_/CH_4_ (10/85)	25				

^a^ IAST selectivity at 1 bar 298 K.

^b^ Pore size distribution determined from Horvath–Kawazoe method applied on CO_2_ isotherm at 195 K.

^c^ Pore size distribution calculated based on NLDFT model from N_2_ sorption at 77K.

^d^ Henry's selectivity

**TABLE 14:** C4 isomers (C_4_H_6_/*i*‐C_4_H_8_/*n*‐C_4_H_8_/*cis*‐C_4_H_8_/*i*‐C_4_H_10_/*n*‐C_4_H_10_) binary or multicomponent separations. The following parameters are listed for comparison: pore size; BET surface area (S_BET_); single‐component gas uptakes; adsorption enthalpies (*Q*_st_); uptake ratio; analysis method, regeneration temperature; and attributed mechanisms. Reticular sorbents are listed in decreasing order of binary or multicomponent uptake ratio for C4 isomers.

Adsorbent, network dimensionality (nD)	Pore size (Å)	Components	Uptake ratio (v/v = 1/1)	Capacity (mmol/g)	*Q*_st_ (kJ/mol)	Analyzation method	Regeneration temperature	S_BET_ (m^2^/g)	Mechanism	Refs.
**Y‐fcu‐MOF**, 3D	4.7 × 4.7	*n*‐C_4_H_10/_ *i*‐C_4_H_10_	+ꝏ	*n*‐C_4_H_10_: 1.97 *i*‐C_4_H_10_: 0.05	—	Breakthrough	—	691^a^	Molecular sieving	[569]
**CALF‐20**, 3D	4.68	*n*‐C_4_H_8_/*i*‐C_4_H_8_	1.67 × 10^7^	*n*‐C_4_H_8_: 2.87 *i*‐C_4_H_8_: 0.11	*n*‐C_4_H_8_: 86.49 *i*‐C_4_H_8_: 0.21	Breakthrough	—	—	Molecular sieving, H‐bonding	[570]
**IPM‐101**, 3D	3.9 × 3.9	*n*‐C_4_H_10_/*i*‐C_4_H_10_	2241^e^	*n*‐C_4_H_8_: 2.3	*n*‐C_4_H_8_: 39.8	Breakthrough, sorption	373 K	342.8	H‐bonding	[571]
**ZU‐36‐Co** (**GeFSIX‐3‐Co**), 3D	3.82 × 3.82, 5.25 × 5.25	*n*‐C_4_H_8_/*i*‐C_4_H_8_	2050^f^	*n*‐C_4_H_8_: 2.35 *i*‐C_4_H_8_: 0.17 *n*‐C_4_H_10_: 2.20 *i*‐C_4_H_10_: 0.12	—	Breakthrough, sorption	He flow at 373 K	257.2	H‐bonding; Van der Waals interactions	[572]
		*n*‐C_4_H_10_/*i*‐C_4_H_10_	140.4^f^		—					
**ZIF‐8/DMPU‐water slurry**, 3D	11.6, 3.4 [573]	*i*‐C_4_H_10_/*n*‐C_4_H_10_	890^g^	*n*‐C_4_H_10_: 4.0	—	Breakthrough, sorption	323.15 K	—	Kinetics	[574]
**NUIG2**, 2D	5.5, 3.7	C_4_H_10_/C_3_H_8_	2.21	—	C_4_H_10_: 38.91	Sorption	—	—	Hydrophobic interactions	[575]
		C_4_H_10_/C_2_H_2_	20.9							
		C_4_H_10_/CH_4_	648							
**TMOF‐1**, 3D	4.5 × 4.5	C_4_H_6_/*i*‐C_4_H_8_	519.2^e^	*n*‐C_4_H_8_: 1.05 C_4_H_6_: 1.65 *i*‐C_4_H_8_: 0.2	—	Breakthrough, sorption	He flow at 423 K	318	H‐bonding	[576]
		*n*‐C_4_H_8_/*i*‐C_4_H_8_	5.42 (93.2^e^)							
		C_4_H_6_/*n*‐C_4_H_8_	1.5 (5.95^e^)							
**MnINA**, 3D	4.62	*n*‐C_4_H_8_/*i*‐C_4_H_8_	327.7	*n*‐C_4_H_8_: 1.79 *i*‐C_4_H_8_: 1.76	—	Breakthrough, sorption	He flow at 373 K	—	C−H···π interactions	[577]
**ZU‐601**, 3D	6.7 × 2.2	C_4_H_6_/*n*‐C_4_H_8_	207	C_4_H_6_: 2.9 *n*‐C_4_H_8_: 0.014 *trans*‐C_4_H_8_: – *iso*‐C_4_H_8_: 0.022	—	Breakthrough, sorption	Vacuum at 393 K	223	H‐bonding	[578]
		C_4_H_6_/*trans*‐C_4_H_8_	—							
**FJI‐H38**, 3D	8.74 × 10.54	*n*‐C_4_H_10_/*i*‐C_4_H_10_	246.75	*n*‐C_4_H_10_:2.39 *i*‐C_4_H_10_: ∼0	*n*‐C_4_H_10_: 20.61	Breakthrough	—	296.2	H‐bonding	[579]
**Znpyc‐CF_3_**, 3D	7.8^d^	*n*‐C_4_H_10_/*i*‐C_4_H_10_	187	*n*‐C_4_H_10_: 2.48 *i*‐C_4_H_10_: 1.17	*n*‐C_4_H_10_: 34.8 *i*‐C_4_H_10_: 25.2	Breakthrough	—	948	H‐bonding	[580]
**ZU‐619**, 3D	9.4 × 9.4	C_4_H_6_/*i*‐C_4_H_8_	72.58^e^	*n*‐C_4_H_8_: 0.68 C_4_H_6_: 1.58 *i*‐C_4_H_8_: 0.32	—	Breakthrough, sorption	He flow at 423 K	154	H‐bonding	[576]
		*n*‐C_4_H_8_/*i*‐C_4_H_8_	1.89 (6.36^e^)							
		C_4_H_6_/*n*‐C_4_H_8_	2.55 (11.42^e^)							
**Y‐abtc‐160**, 3D	—	C_4_H_6_/*n*‐C_4_H_8_	40.93	C_4_H_6_: 2.21 *n*‐C_4_H_8_: 0.054 *i*‐C_4_H_8_: 0.035	—	Breakthrough, sorption	—	332.2	Pore size, H‐bonding, π⋅⋅⋅π interactions	[581]
		C_4_H_6_/*i*‐C_4_H_8_	63.14							
**Mn‐bpdc MOF**, 3D	4.2 × 6.1	C_4_H_6_/*n*‐C_4_H_8_	40.0	C_4_H_6_: 0.04 *n*‐C_4_H_8_:0.98 *i*‐C_4_H_8_:0.03 *n*‐C_4_H_10_:0.01 *i*‐C_4_H_10_:∼0	—	Breakthrough, sorption	298 K	159	H‐bonding	[582]
		C_4_H_6_/*i*‐C_4_H_8_	45.0							
**COF‐300 (dia‐c5)**, 3D	5.9 × 5.9	*i*‐C_4_H_8_/*trans*‐C_4_H_8_	38.4	*n*‐C_4_H_8_: 7.8 *trans*‐C_4_H_8_: 1.1 *i*‐C_4_H_8_: 10.7 *n*‐C_4_H_10_: 6.0 *i*‐C_4_H_10_: 8.0 C_4_H_6_: 9.3	*n*‐C_4_H_8_: 13.1 *trans*‐C_4_H_8_: 2.6 *cis*‐C_4_H_8_: 2.6 *i*‐C_4_H_8_: 20.0 *n*‐C_4_H_10_: 11.8 *i*‐C_4_H_10_: 16.5 C_4_H_6_: 15.5	Sorption	—	5546	Pore shape, π···π interactions	[583]
		*i*‐C_4_H_8_/*cis*‐C_4_H_8_	35.8							
		*i*‐C_4_H_8_/*n*‐C_4_H_10_	13.4							
		*i*‐C_4_H_8_/*n*‐C_4_H_8_	6.3							
		*i*‐C_4_H_8_/*i*‐C_4_H_10_	2.6							
		*i*‐C_4_H_8_/C_4_H_6_	2.5							
**[Ag_2_(*o*‐Hmpba)_2_(*o*‐H_2_mpba)_2_]**, 2D	1.8 × 4.4	*n*‐C_4_H_10_/*i*‐C_4_H_10_	23	*n*‐C_4_H_10_: 0.9 *i*‐C_4_H_10_: 0.012	—	Breakthrough, sorption	—	0.99	Molecular‐Sieving, flexibility	[584]
**BFFOUR‐Cu‐dpds**, 2D	3.9 × 4.0	*n*‐C_4_H_8_/*n*‐C_4_H_10_	22.9^f^	*n*‐C_4_H_8_: 0.70	*n*‐C_4_H_8_: 33.37	Breakthrough, sorption	333 K	140	C‐H∙∙∙F, C‐H∙∙∙π interactions	[525]
**TaOFFIVE‐3‐Ni** (**ZU‐96**), 3D	3.2‐4.7	C_4_H_6_/*i*‐C_4_H_8_	20.83	C_4_H_6_: 1.90 *n*‐C_4_H_8_:0.98 *i*‐C_4_H_8_:0.09	—	Breakthrough, sorption	—	—	H‐bonding	[585]
**Y‐dbai**, 3D	4.4 × 4.4	C_4_H_6_/*i*‐C_4_H_8_	20.6	C_4_H_6_: 2.88 *n*‐C_4_H_8_: 1.07 i‐C_4_H_8_: 0.14	C_4_H_6_: 58 *n*‐C_4_H_8_: 46	Breakthrough, sorption	Ar flow at 423 K	—	C─H···π, C─H···O, C─H···N interactions	[586]
		1‐C_4_H_8_/*i*‐C_4_H_8_	7.6							
**ZU‐602**, 3D	6.8 × 2.5	C_4_H_6_/*n*‐C_4_H_8_	66	C_4_H_6_: 1.45 *n*‐C_4_H_8_: 0.022 *trans*‐C_4_H_8_: 0.144 *i*‐C_4_H_8_: 0.022	—	Breakthrough, sorption	Vacuum at 393 K	123	H‐bonding	[578]
		C_4_H_6_/*trans*‐C_4_H_8_	10.1							
**CuINA**, 3D	4.84	*n*‐C_4_H_8_/*i*‐C_4_H_8_	7.1	*n*‐C_4_H_8_: 1.56 *i*‐C_4_H_8_: 1.50	—	Breakthrough, sorption	He flow at 373 K	—	C−H···π interactions	[577]
**SOFOUR‐DPDS‐Ni**, 2D	5.3 × 4.5 × 5.8	C_4_H_6_/*i*‐C_4_H_8_	9.9 (2321.8^e^)	C_4_H_6_: 1.36 *n*‐C_4_H_8_: 1.25 *i*‐C_4_H_8_: 0.17	C_4_H_6_: 77 *n*‐C_4_H_8_: 38	Breakthrough, sorption	He flow at 343 K	270	Van der Waals interactions, H‐bonding	[587]
		*n*‐C_4_H_8_/*i*‐C_4_H_8_	8.7 (233.5^e^)							
**KAUST‐7** (**NbOFFIVE‐1‐Ni**), 3D	3.14 × 3.14	C_4_H_6_/*n*‐C_4_H_8_	3.18	C_4_H_6_: 1.55 *n*‐C_4_H_8_: 0.49 *i*‐C_4_H_8_: 0	—	Breakthrough, sorption	Ar flow at 378 K	205.96^b^	Pore size	[588]
		C_4_H_6_/*i*‐C_4_H_8_	10.4							
**ZU‐36‐Ni** (**GeFSIX‐3‐Ni**), 3D	4.75 × 4.75	*trans*‐C_4_H_8_/*cis*‐C_4_H_8_	7	*trans*‐C_4_H_8_: 2.45	*trans*‐C_4_H_8_: 42.0	Breakthrough, sorption	298 K	313^c^	π–π interactions	[589]
**GeFSIX‐14‐Cu‐i** (**ZU‐33**), 3D	3.0 × 4.2	C_4_H_6_/*n*‐C_4_H_8_	4.68	C_4_H_6_:2.67 *n*‐C_4_H_8_: 0.57 *i*‐C_4_H_8_:0.42	—	Breakthrough	—	424.1^c^	Molecular recognition, molecular sieving	[590]
		C_4_H_6_/*i*‐C_4_H_8_	6.40							
		*n*‐C_4_H_8_/*i*‐C_4_H_8_	1.37							
**KAUST‐8** (**AlFFIVE‐1‐Ni**), 3D	5.01 × 5.01	C_4_H_6_/*i*‐C_4_H_8_	5.19	C_4_H_6_: 2.49 *n*‐C_4_H_8_: 1.94 *i*‐C_4_H_8_: 0.48	—	Breakthrough, sorption	Ar flow at 378 K	240	Pore size, H‐bonding	[588]
		*n*‐C_4_H_8_/*i*‐C_4_H_8_	3.81							
**Ni_2_(m‐dobdc)**, 3D	9.8 [591]	*n*‐C_4_H_8_/*cis*‐C_4_H_8_ ^f^ (2.3±0.1^g^)	2.4	—	—	Breakthrough, sorption	—	1321 [592]	UMCs	[593]
		*n*‐C_4_H_8_/*trans*‐C_4_H_8_ ^f^(2.1±0.1^g^)	6.0							
		*n*‐C_4_H_8_/(*cis*‐C_4_H_8_, *trans*‐C_4_H_8_)	2.8							
**NbFSIX‐2‐Cu‐i** (**ZU‐52**), 3D	4.1 × 4.3	C_4_H_6_/*n*‐C_4_H_8_	1.15	C_4_H_6_:2.64 *n*‐C_4_H_8_: 2.26 *i*‐C_4_H_8_:0.48	—	Breakthrough	—	440.0^c^	Molecular recognition, molecular sieving	[590]
		C_4_H_6_/*i*‐C_4_H_8_	5.74							
		*n*‐C_4_H_8_/*i*‐C_4_H_8_	5.00							
**Co_2_(m‐dobdc)**, 3D	9.8 [591]	*n*‐C_4_H_8_/*cis*‐C_4_H_8_ ^f^	2.9	—	—	Breakthrough, sorption	—	1264 [592]	UMCs	[593]
		*n*‐C_4_H_8_/*trans*‐C_4_H_8_ ^f^	5.7							
**SIFSIX‐3‐Ni**, 3D	3.8 × 3.8 [594]	C_4_H_6_/*n*‐C_4_H_8_	1.00	C_4_H_6_:2.46 *n*‐C_4_H_8_: 2.45 *i*‐C_4_H_8_:0.52	—	Breakthrough	—	368 [137]	—	[590]
		C_4_H_6_/*i*‐C_4_H_8_	4.73							
		*n*‐C_4_H_8_/*i*‐C_4_H_8_	4.73							
**ZU‐36‐Fe** (**GeFSIX‐3‐Fe**), 3D	4.85 × 4.85	*trans*‐C_4_H_8_/*cis*‐C_4_H_8_	3.6	*trans*‐C_4_H_8_: 1.81	*trans*‐C_4_H_8_: 61.8	Breakthrough, sorption	298 K	295^c^	Pore size	[589]
**TMA‐VT‐1**, 3D	4.3 × 4.3	C_4_H_6_/*n*‐C_4_H_8_	1.14	C_4_H_6_: 0.80 *n*‐C_4_H_8_: 0.70 *n*‐C_4_H_10_: 0.25	—	Breakthrough, sorption	He flow	38	Van der Waals interactions	[595]
		C_4_H_6_/*n*‐C_4_H_10_	3.20							
**Mg‐gallate**, 3D	3.6 × 4.6	*trans*‐C_4_H_8_/*cis*‐C_4_H_8_	3.2	*trans*‐C_4_H_8_: 1.85 *cis*‐C_4_H_8_: 0.58	—	Breakthrough	He flow at 393 K	559 [291]	Shape selective, C···H–O interactions	[596]
**Co‐gallate**, 3D	3.7 × 5.0 [291]	*trans*‐C_4_H_8_/*cis*‐C_4_H_8_	2.9	*trans*‐C_4_H_8_: 1.67 *cis*‐C_4_H_8_: 0.58	—	Breakthrough	He flow at 393 K	475 [291]	Shape selective, C···H–O interactions	[596]
**GeFSIX‐2‐Cu‐i** (**ZU‐32**), 3D	4.5 × 4.5	C_4_H_6_/*n*‐C_4_H_8_	1.12	C_4_H_6_:3.67 *n*‐C_4_H_8_: 3.3 *i*‐C_4_H_8_:1.25	—	Breakthrough	—	467.6^c^	Molecular recognition, molecular sieving	[590]
		C_4_H_6_/*i*‐C_4_H_8_	2.94							
		*n*‐C_4_H_8_/*i*‐C_4_H_8_	2.26							
**TMA‐VT‐5**, 3D	7.4 × 7.4, 3.9 × 3.9	C_4_H_6_/*n*‐C_4_H_8_	1.29	C_4_H_6_: 0.97 *n*‐C_4_H_8_: 0.75 *n*‐C_4_H_10_: 0.37	—	Breakthrough, sorption	He flow	5	Van der Waals interactions	[595]
		C_4_H_6_/*n*‐C_4_H_10_	2.62							
**Ni‐gallate**, 3D	3.5 × 4.9 [291]	*trans*‐C_4_H_8_/*cis*‐C_4_H_8_	2.5	*trans*‐C_4_H_8_: 1.09 *cis*‐C_4_H_8_: 0.44	—	Breakthrough	He flow at 393 K	424 [291]	Pore shape, C···H–O interactions	[596]
**K‐VT‐1**, 3D	4.3 × 4.3	C_4_H_6_/*n*‐C_4_H_8_	1.16	C_4_H_6_: 1.0 *n*‐C_4_H_8_: 0.86 *n*‐C_4_H_10_: 0.54	—	Breakthrough, sorption	He flow	235	Van der Waals interactions	[595]
		C_4_H_6_/*n*‐C_4_H_10_	1.85							
**TIFSIX‐Cu‐TPB**, 3D	7.02 × 7.10, 8.28 × 8.31	C_4_H_6_/*i*‐C_4_H_8_	1.31 (2.6^f^)	C_4_H_6_: 3.35 *n*‐C_4_H_8_: 2.96 *i*‐C_4_H_8_: 2.56	C_4_H_6_: 61.4 *n*‐C_4_H_8_: 55.3 *i*‐C_4_H_8_: 59.4	Breakthrough, sorption	He flow at 333 K	426.8	C−H···π, π···π interactions, H bonding	[597]
		C_4_H_6_/*n*‐C_4_H_8_	1.13 (2.2^f^)							
										
**SIFSIX‐Cu‐TPB**, 3D	6.96 × 7.12, 8.37 × 8.43	C_4_H_6_/*i*‐C_4_H_8_	1.32 (1.7^f^)	C_4_H_6_: 3.23 *n*‐C_4_H_8_: 2.71 *i*‐C_4_H_8_: 2.44	C_4_H_6_: 62.4 *n*‐C_4_H_8_: 55.8 *i*‐C_4_H_8_: 59.2	Breakthrough, sorption	He flow at 333 K	257.3	C−H···π, π···π interactions, H bonding	[597]
		C_4_H_6_/*n*‐C_4_H_8_	1.19 (1.5^f^)							
**TIFSIX‐2‐Cu‐i**, 3D	4.8 × 4.8	C_4_H_6_/*n*‐C_4_H_8_	1.10	C_4_H_6_:4.05 *n*‐C_4_H_8_: 3.67 *i*‐C_4_H_8_:2.90	—	Breakthrough	—	480.5 [598]	H‐bonding	[590]
		C_4_H_6_/*i*‐C_4_H_8_	1.40							
		*n*‐C_4_H_8_/*i*‐C_4_H_8_	1.27							
**SIFSIX‐2‐Cu‐i**, 3D	4.7 × 4.7	C_4_H_6_/*n*‐C_4_H_8_	1.3	C_4_H_6_:3.99 *n*‐C_4_H_8_: 3.06 *i*‐C_4_H_8_:1.74	—	Breakthrough	—	503 [137]	H‐bonding	[590]
		C_4_H_6_/*i*‐C_4_H_8_	2.29							
		*n*‐C_4_H_8_/*i*‐C_4_H_8_	1.76							
**Mn_2_(*m*‐dobdc)**, 3D	9.8 [591]	*n*‐C_4_H_8_/*cis*‐C_4_H_8_ ^f^	2.0	—	—	Breakthrough, sorption	—	1349 [592]	UMCs	[593]
**Fe_2_(*m*‐dobdc)**, 3D	9.8 [591]	*n*‐C_4_H_8_/*cis*‐C_4_H_8_ ^f^	2.0	—	—	Breakthrough, sorption	—	1295 [592]	UMCs	[593]
**Zn‐bzc‐2CH_3_**, 3D	4.13 × 4.13	*n*‐C_4_H_10_/*i*‐C_4_H_10_	*n*‐C_4_H_10_ selective	*n*‐C_4_H_10_:2.38 *i*‐C_4_H_10_: 0.03	—	Breakthrough, sorption	—	387.2	Van der Waals interactions	[599]
**Zr‐fum‐fcu‐100**, 3D	4.7 × 4.7, 5.2 × 5.2, 7.6 × 7.6	*n*‐C_4_H_10_/*i*‐C_4_H_10_	*n*‐C_4_H_10_ selective	*n*‐C_4_H_10_:1.23	—	Breakthrough, sorption	—	718	Pore size	[600]
**SIFSIX‐1‐Cu**, 3D	9.5 × 9.5 [601]	C_4_H_6_/*n*‐C_4_H_8_	1.10	C_4_H_6_:6.75 *n*‐C_4_H_8_: 6.43 *i*‐C_4_H_8_:6.28	—	Breakthrough	—	1178 [602]	—	[590]
		C_4_H_6_/*i*‐C_4_H_8_	1.13							
		*n*‐C_4_H_8_/*i*‐C_4_H_8_	1.00							
**Cu_3_(BTC)_2_** (**HKUST‐1**), 3D	9 × 9	*i*‐C_4_H_8/_ *i*‐C_4_H_10_	*i*‐C_4_H_8_ selective	*i*‐C_4_H_8_: 6.5 *i*‐C_4_H_10_:5.1	*i*‐C_4_H_10_: ‐42	Breakthrough	—	1055 [603]	Van der Waals interactions	[604]
**ZJNU‐30a**, 3D	13.6, 21.6^d^	*trans*‐C_4_H_8_/*cis*‐C_4_H_8_/*n*‐C_4_H_8_/*i*‐C_4_H_8_	*cis*‐C_4_H_8_ selective	—	—	Breakthrough	—	1570	—	[605]
**ZJNU‐80a**, 3D	9.2^d^	C_4_H_6_/*n*‐C_4_H_8_	1.00	C_4_H_6_:8.35 *n*‐C_4_H_8_: 8.35 *i*‐C_4_H_8_:6.80	—	Adsorption	—	2279	Pore size	[606]
		C_4_H_6_/*i*‐C_4_H_8_	1.23							
		*n*‐C_4_H_8_/*i*‐C_4_H_8_	1.23							
**NOTT‐101a**, 3D	10.2^d^	C_4_H_6_/*n*‐C_4_H_8_	1.00	C_4_H_6_:9.28 *n*‐C_4_H_8_: 9.28 *i*‐C_4_H_8_:9.15	—	Adsorption	—	2755	Pore size	[606]
		C_4_H_6_/*i*‐C_4_H_8_	1.01							
		*n*‐C_4_H_8_/*i*‐C_4_H_8_	1.01							
**Zn(Hmpba)_2_**, 3D	5.4 × 6.4	C_4_H_6_/*n*‐C_4_H_8_	1.11	C_4_H_6_:3.10 *n*‐C_4_H_8_: 2.80 *i*‐C_4_H_8_:2.80	—	Breakthrough	—	—	Flexibility	[607]
		C_4_H_6_/*i*‐C_4_H_8_	1.11							
		*n*‐C_4_H_8_/*i*‐C_4_H_8_	1							
**Cu_4_(m_4_‐O)(m_2_‐ OH)_2_(Me_2_trz‐pba)_4_**, 3D	4.5 × 5.5	*n*‐C_4_H_8_/*i*‐C_4_H_8_	0.94	*n*‐C_4_H_8_: 4.10 *i*‐C_4_H_8_:4.35	*n*‐C_4_H_8_: 49.5 *i*‐C_4_H_10_:46.4 *n*‐C_4_H_8_: 45.6 *i*‐C_4_H_8_: 40.4	Adsorption	—	—	Flexibility	[608]
**[Zn(NO_2_ip)(dpe)]*_n_*** (**SD‐65**), 3D	Discrete voids	C_4_H_6_/*i*‐C_4_H_8_/*n*‐C_4_H_8_/2‐C_4_H_8_/*i*‐C_4_H_10_/*n*‐C_4_H_10_	C_4_H_6_ selective	C_4_H_6_: 1.16	—	Breakthrough	298 K	—	C−H···π interactions	[609]
**Y‐fum‐fcu‐MOF**, 3D	4.7 × 4.7	C_4_H_6_/*i*‐C_4_H_8_/*n*‐C_4_H_8_/*cis*‐C_4_H_8_/*trans*‐C_4_H_8_	trans‐C_4_H_8_ selective	—	—	GC, breakthrough	N_2_ flow at 423 K	835	Pore shape, size	[610]
**[Zn_2_(btm)_2_]**, 3D	3.6 × 3.6	C_4_H_6_/*i*‐C_4_H_8_/*n*‐C_4_H_8_/*n*‐C_4_H_10_	*i*‐C_4_H_8_ selective	C_4_H_6_:2.0 *i*‐C_4_H_8_: 2.0 *n*‐C_4_H_8_:2.0 *n*‐C_4_H_10_: 2.0	—	Breakthrough	—	622^b^ [611]	UMCs, pore size, shape	[82]
**CMS‐PMOF‐1**, 3D	5 × 5	*n*‐C_4_H_10_/*i*‐C_4_H_10_	*n*‐C_4_H_10_ selective	*n*‐C_4_H_10_: 1.9 *i*‐C_4_H_10_: 1.43	—	Breakthrough, sorption	—	799	Molecular sieving	[612]
**UiO‐66 (Zr)**, 3D	8 × 8, 11 × 11	*n*‐C_4_H_10_/*i*‐C_4_H_10_	*n*‐C_4_H_10_ selective	—	—	NMR	—	1145	Kinetics	[613]
**RUB‐41**, 3D	2.7 × 5, 4 × 6.5	*trans*‐C_4_H_8_/*cis*‐C_4_H_8_/*n*‐C_4_H_8_	—	—	—	Breakthrough, sorption	—	—	Pore size, shape	[614]

^a^ BET surface area was calculated by Ar sorption isotherm.

^b^ Langmuir surface area calculated from CO_2_ sorption at 195 K or 298 K.

^c^ BET surface area calculated from CO_2_ sorption isotherms at 196 K or 273 K.

^d^ Pore size calculated from N_2_ isotherm at 77 K.

^e^ Henry selectivity.

^f^ Ideal Adsorbed Solution Theory (IAST) selectivity.

^g^ Selectivity calculated based on mass ratio.

**TABLE 15:** C6 alkane (*n*‐HEX, 2MP, 3MP, 22DMB, 23DMB) and cyclic (Bz, Cy) isomers binary or multicomponent separations. The following parameters are listed for comparison: pore size; BET surface area (S_BET_); single‐component gas uptakes; adsorption enthalpies (*Q*_st_); selectivity; analysis method, regeneration temperature; and attributed mechanisms. Reticular sorbents are listed in decreasing order of binary or multicomponent uptake ratio for C6 isomers.

	Adsorbent, network dimensionality (nD)	Pore size (Å)	Components	Binary selectivity^a^	Capacity (mmol/g)	*Q*_st_ (kJ/mol)	Analyzation method	Regeneration temperature (K)	S_BET_ (m^2^/g)	Mechanism	Refs.
C6 alkane isomers	**UU‐200**, 3D (303K)	5 × 5	*n*‐HEX/22DMB	1×10^5^	*n*‐HEX: 1.69 3MP: 1.14 22DMB: 0.09	*n*‐HEX: 53.3 3MP: 44.9	Breakthrough	—	482^h^	Molecular sieving, C‐H···O H‐bonding, C‐H···π interactions	[615]
			3MP/22DMB	3×10^4^							
			*n*‐HEX/3MP	160							
	**ZUL‐C5**, 3D	4.61	*n*‐HEX/22DMB	863	*n*‐HEX: 1.70 (303 K) 3MP: 1.71 (303 K) 22DMB: 0.37 (303 K)	—	Breakthrough	N_2_, 423 K	611	Van der Waals interactions	[616]
			3MP/22DMB	124							
	**CopzNi**, 3D	7.11 × 7.22	2MP/22DMB	420 (303K)	*n*‐HEX (363K): 1.88	*n‐*HEX: 78.8 2MP: 45.2	GC	N_2_, 423 K	607	Molecular sieving	[617]
			*n*‐HEX/2MP	171.7 (363K)							
	**ZUL−C6**, 3D	4.94 × 5.76	*n*‐HEX/22DMB	172	*n*‐HEX: 1.08 3MP: 0.86	—	Breakthrough	—	607	Flexibility	[618]
	**NipzNi**, 3D	7.10 × 7.27	2MP/22DMB	27.3(303K)	*n*‐HEX (363K): 1.49	*n‐*HEX: 65.2 2MP: 32.1	GC	N_2_, 393K	664	Molecular sieving	[617]
			*n*‐HEX/2MP	73.7 (363K)							
	**JNU‐80‐LP**, 3D	6.5	2PMP/22DMB	45.3^g^	*n‐*HEX: 2.1 2MP: 1.7	—	GC	—	670	Molecular sieving	[619]
	**Zn‐tcpt**, 3D	4.9^f^	3MP/22DMB	28.5^g^	*n*‐HEX: 3.25 3MP: 2.14	—	Breakthrough	—	1135	Pore size	[620]
			*n*‐HEX/22DMB	43.3^g^							
	**Mn‐dhbq**, 3D (303K, 393K)	5.58^f^	3MP/23DMB	34.57^g^ (303K) 22.18^g^ (333K)	*n*‐HEX: 1.53	—	Breakthrough	N_2_ flow at 423K	429	Polarizability, H‐bonding	[621]
	**Zeolite 5A**, 3D	4 × 4 [622]	*n‐*HEX/3MP	29^b^	—	—	GC	—	650	Molecular sieving	[623]
			*n‐*HEX/22DMB	18^b^							
			3MP/22DMB	1.0^b^							
	**{[Fe_3_(μ_3_‐O)](6FDCA)_3_·2DMF}**, 3D (303K)	8.45 × 11.02	*n*‐HEX/22DMB	27.83^f^	*n*‐HEX: 0.79^g^ 3MP: 0.58^g^ 22DMB: 0.43^g^	—	Breakthrough	298	269.5	Molecular sieving	[624]
			*n*‐HEX/3MP	8.66^f^							
			3MP/22DMB	3.82^f^							
	**BNF‐100a**, 3D	14.3	*n*‐HEX/22DMB	13.6^g^	*n*‐HEX: 1.33 (303K) 3MP: 1.23 (303K) 22DMB: 0.1 (303K)	—	Breakthrough	—	483.1	C‐H⋯π interactions	[625]
			3MP/22DMB	12.5^g^							
	**Zr_6_(μ_3_‐O)_4_(μ_3_‐OH)_4_(bptc)_3_**_,_ 3D	4.5	*n‐*HEX/3MP	13^g^	3MP: 1.23 (423K) 3MP: 0 22DMB: 0	*n*‐HEX: 48	Breakthrough	—	1030	Van de Waals interactions	[626]
	**ZIF‐8**, 3D (398 K)	11.6, 3.4	*n‐*HEX/3MP	3.7^b^	—	—	GC	—	1813	Kinetics	[623]
			*n‐*HEX/22DMB	11.9^b^							
			3MP/22DMB	9.2^b^							
	**Zn(BPZ(NH_2_)_2_)**, 3D	5.4 × 5.4	*n*‐HEX/2MP	3.42^j^	—	*n*‐HEX: 61.7 2MP: 59.8 3MP: 67.9 23DMB: 54.2 22DMB: 56.9	GC	—	155	Molecular sieving	[627]
			*n*‐HEX/3MP	4.77^j^							
			*n*‐HEX/23DMB	8.12^j^							
			*n*‐HEX/22DMB	10.76^j^							
	**Zn‐Me_2_BPZ**, 3D	4.9 × 4.9	*n*‐HEX/2MP	2.42^j^	—	*n*‐HEX: 85.0 2MP: 82.9 3MP: 80.7 23DMB: 79.3 22DMB: 71.9	GC	—	123	Molecular sieving	[627]
			*n*‐HEX/3MP	3.28^j^							
			*n*‐HEX/23DMB	5.27^j^							
			*n*‐HEX/22DMB	9.48^j^							
	**Cu‐MoO_4_‐TPA**, 3D	4.96^f^	*n*‐HEX/23DMB	10.5^g^	*n*‐HEX: 1.2 2MP: 0.42 3MP: 0.42 23DMB: 0.12 22DMB: 0.03	—	Breakthrough	—	217	C‐H···O interactions	[628]
			*n*‐HEX/2MP	2.61							
			*n*‐HEX/3MP	2.67							
	**IM‐22**, 3D	—	*n‐*HEX/3MP	1.7^b^	—	—	GC	—	575	Kinetics	[623]
			*n‐*HEX/22DMB	8.4^b^							
			3MP/22DMB	5.3^b^							
	**ZIF‐8**, 3D (423K)	11.6, 3.4	*n‐*HEX/2MP	5.8^b^	*n‐*HEX: 5.9, 2MP: 1.05	—	GC	—	1285.2	Kinetics	[573]
	**MIL‐140C**, 3D	5.7 × 5.7	*n*‐HEX/3MP	7^g^	—	*n*‐HEX: 71.5	Breakthrough	—	—	Kinetics	[629]
	**UiO‐66(Zr)**, 3D	6 × 6	22DMB/*n‐*HEX	7.6^d^	—	—	GC	—	885 [630]	Pore size, shape	[631]
			23DMB/*n‐*HEX	5.2^d^							
			*n‐*HEX/2MP	0.9^d^							
	**HKUST‐1**, 3D	5.6 × 5.6	22DMB/*n‐*HEX	0.5^d^	—	—	GC	—	1055 [603]	UMCs	[631]
			23DMB/*n‐*HEX	0.61^d^							
			*n‐*HEX/2MP	6.8^d^							
	**MIL‐125(Ti)**, 3D	6.13 × 6.13, 12.55 × 12.55 [632]	22DMB/*n‐*HEX	0.82^d^	—	—	GC	—	1446 [632]	Pore shape	[631]
			23DMB/*n‐*HEX	0.79^d^							
			*n‐*HEX/2MP	3.9^d^							
	**MIL‐125(Ti)‐NH_2_**, 3D (373K)	5‐7	23DMB/22DMB	1.4^g^	—	—	Breakthrough	—	1550	Molecular sieving	[633]
			3MP/22DMB	1.8^g^							
			*n‐*HEX/22DMB	2.6^g^							
	**MIL‐140B**, 3D	4.0. × 4.0	*n*‐HEX/3MP	5^g^	—	*n*‐HEX: 84.5	Breakthrough	—	—	Kinetics	[629]
	**UiO‐66‐Br**, 3D (423K)	—	22DMB/*n‐*HEX	2.9^b^	—	—	Breakthrough	—	600	—	[634]
			23DMB/*n‐*HEX	2.9^b^							
			23MP/*n‐*HEX	2.9^b^							
	**UiO‐66‐NO_2_**, 3D (423K)	—	22DMB/*n‐*HEX	1.3^b^	—	—	Breakthrough	—	650	—	[634]
			23DMB/*n‐*HEX	1.3^b^							
			23MP/*n‐*HEX	1.1^b^							
	**UiO‐66‐NH_2_**, 3D (423K)	—	22DMB/*n‐*HEX	1.2^b^	—	—	Breakthrough	—	670	—	[634]
			23DMB/*n‐*HEX	1.2^b^							
			23MP/*n‐*HEX	1.1^b^							
	**HIAM‐410LI**, 3D	4 × 4	3MP/22DMB	1.31^b^	3MP: 1.50 3MP: 1.24 22DMB: 1.03	3MP: 90.1 3MP: 55.3 22DMB: 27.9	Breakthrough	—	832	Pore size, shape	[635]
	**MIL‐100(Cr)‐MEDA**, 3D (343 K)	6.6 × 2.5 × 1.8	23DMB/22DMB	1.4^g^	*n*‐HEX: 0.57 3MP: 0.35 23DMB: 0.30 22DMB: 0.21	—	Breakthrough	—	2400	Molecular sieving	[633]
			3MP/22DMB	1.8							
			*n*‐HEX/23DMB	2.7							
	**ZIF‐76**, 3D	5.4, 12.2 [636]	3MP/*n‐*HEX	0.8^b^	—	—	GC	—	1560	Pore shape	[623]
			22DMB/*n‐*HEX	0.7^b^							
			22DMB/3MP	0.8^b^							
	**[Zn_2_(HBDC)_2_(dmtrz)_2_]**, 3D	6.7	*n*‐HEX/3MP/22DMB	*n*‐HEX selective	*n*‐HEX: 13.3wt%^c^ 3MP: 12.1wt%^c^ 22DMB: 3.5wt%^c^	—	GC	—	552	Molecular sieving	[637]
	**MIL‐53(Fe), 3D (CF_3_)_2_**, 3D	5.6 × 5.6 (LP), 4.2 × 4.2 (NP)	*n‐*HEX/3MP/22DMB	*n*‐HEX selective	—	—	GC	—	12	Flexibility, molecular sieving	[638]
	**UiO‐66**, 3D	7 × 7	*n‐*HEX/3MP/22DMB/23DMB	*n*‐HEX selective	—	—	GC	—	885 [630]	Van der Waals interactions	[639]
	**MOF‐CJ_3_**, 3D	11.6 × 11.6	*n‐*HEX/3MP/22DMB	*n*‐HEX selective	—	—	GC	—	525	Van der Waals interactions	[640]
	**MIL‐101(Cr)**, 3D	16 × 16, 12 × 12	*n‐*HEX/2MP/22DMB/23DMB	*n*‐HEX selective	*n‐*HEX: 10.2 2MP: 9.2 22DMB: 8.6 23DMB: 8.4	—	GC	—	3990	Van der Waals interactions, pore size	[641]
	**Fe_2_(BDP)_3_**, 3D (433K)	13.2 × 13.2	*n*‐HEX/2MP/3MP/22DMB/23DMB	*n*‐HEX selective	*n‐*HEX: 1.32, 2MP: 1.18, 3MP: 1.27, 23DMB: 1.37, 22DMB: 1.22	—	GC	—	1230	Van der Waals interactions	[71]
	**Ca(H_2_tcpb)**, 3D (333K)	5.5 × 5.5	*n*‐HEX/2MP/3MP/22DMB	*n*‐HEX selective	*n‐*HEX: 1.14, 3MP: 0.12, 22DMB: 0	—	GC	N_2_, 423K	220	Molecular sieving	[642]
	**Zr_6_(μ_3_‐O)_4_(μ_3_‐OH)_4_(bptc)_3_**, 3D	14.6 × 17.2	*n*‐HEX/3MP/23DMB	*n*‐HEX selective (423K)	*n*‐HEX: 1.51, 3MP: 0.29, 23DMB: 0	*n‐*HEX: 48	GC	—	1030	Molecular sieving	[626]
	**Al‐fumarate**, 3D (453K)	5.7 × 6.0	*n‐*HEX/3MP/23DMB	*n*‐HEX selective	—	—	GC	—	879.5^e^	Van der Waals interactions	[643]
	**MIL‐127(Fe)**, 3D	6 × 6, 10 × 10	*n‐*HEX/3MP/23DMB/22DMB	*n*‐HEX selective	—	—	Breakthrough	—	1400	Molecular sieving	[633]
	**HIAM‐203**, 3D (423K)	4.82 × 4.82	*n*‐HEX/3MP/22DMB	—	*n*‐HEX selective	—	GC	—	499	H‐bonding, pore size	[644]
	**ZIF‐69**, 3D (423K)	7.8 × 7.8	—	—	*n‐*HEX: 3.95, 3MP: 1.16	—	GC	—	845.1	Kinetics	[573]
	**Zr‐abtc**, 3D	7 × 7	*n*‐HEX/3MP/22DMB	*n*‐HEX selective	*n*‐HEX: 3.31 3MP: 2.14 22DMB: 1.51	—	Breakthrough	—	1300	Pore size	[645]
	**Al‐bttotb**, 3D	5.6 × 5.6	*n*‐HEX/3MP/22DMB	*n*‐HEX selective	*n*‐HEX: 1.75 3MP: 1.09 22DMB: 0.14	—	Breakthrough	—	600	Pore size	[645]
	**Co‐FA**, 3D	5.5 × 5.5	*n*‐HEX/3MP/22DMB	*n*‐HEX selective	*n*‐HEX: 1.22 3MP: 1.37 22DMB: 0.31	—	Breakthrough	—	350	Pore size	[645]
	**ZSM‐5**, 3D	5.1 × 5.5, 5.4 × 5.6	*n*‐HEX/3MP/22DMB	*n*‐HEX selective	*n*‐HEX: 1.31 3MP: 0.32 22DMB: 0.17	—	Breakthrough	—	360	Pore size	[645]
	**Zeolite 5A**, 3D	5, 11.4	*n*‐HEX/3MP/22DMB	*n*‐HEX selective	*n*‐HEX: 1.69 3MP: 0.32 22DMB: 0.32	—	Breakthrough	—	650	Pore size	[645]
	**NU‐2002**, 3D	4.7 × 4.7	*n*‐HEX/2MP/3MP/23DMB/22DMB	*n*‐HEX+2MP+3MP selective	*n*‐HEX: 1.41 2MP: 1.54 3MP: 1.56 23DMB: 1.67 22DMB: 1.31	—	Vapor sorption isotherms	—	405	Pore size	[646]
	**NU‐2200**, 3D	5 × 5	*n*‐HEX/2MP/3MP/23DMB/22DMB	*n*‐HEX selective	*n*‐HEX: 1.32 2MP: 1.03 3MP: 0.89 23DMB: 0.21 22DMB: 0.17	—	GC	—	355	Pore size, shape	[647]
	**ZIF‐8‐90‐S1**, 3D	3.1	—	*n*‐HEX selective	2.82	—	Vapor sorption isotherms	—	1374	Van der Waals interactions	[648]
	**ZIF‐8‐90‐S5**, 3D	8.8	—	*n*‐HEX selective	3.10	—	Vapor sorption isotherms	—	1208	Van der Waals interactions	[648]
	**ZIF‐8**, 3D (373K)	3.4, 11.4	*n*‐HEX/2MP/23DMB/22DMB	*n*‐HEX selective	*n*‐HEX: 2.6 2MP: 2.3 23DMB: 1.8 22DMB: 0.2	—	Vapor sorption isotherms	—	1800	Molecular sieving	[649]
	**Zn_2_(bdc‐R)_2_(bpy)**, 3D	—	*n*‐HEX/3MP/22DMB	*n*‐HEX selective	*n*‐HEX: 1.68 3MP: 0.48 22DMB: ∼0	—	Vapor sorption isotherms	—	—	Flexibility, π···π interactions	[650]
	**MIP‐214**, 3D	6.1 × 6.1, 2.6 × 2.6	*n*‐HEX/2MP/3MP/23DMB/22DMB	—	*n*‐HEX: 1.41 2MP: 0.32 3MP: 0.21 23DMB: 0.15 22DMB: 0.1	—	Breakthrough	—	1140	Pore shape, size	[651]
	**NU‐2004**, 3D	5 × 5	*n*‐HEX/2MP/3MP/23DMB/22DMB	—	*n*‐HEX: 1.47 2MP: 0.74 3MP: 0.8 23DMB: 0.27 22DMB: 0.27	—	Vapor sorption isotherms	—	435	Molecular sieving	[652]
	**NU‐2005**, 3D	5 × 5	*n*‐HEX/2MP/3MP/23DMB/22DMB	—	—	—	Vapor sorption isotherms	—	380	Molecular sieving	[652]
	**HIAM‐318**, 3D	5.4 × 5.4	*n*‐HEX/3MP/22DMB	*n*‐HEX, 3MP selective	*n*‐HEX: 1.49 3MP: 1.34 22DMB: 0.22	—	Breakthrough	—	599	Pore size	[653]
	**CAU‐21‐ODB**, 3D	4.6 × 4.6	*n*‐HEX/3MP/22DMB	*n*‐HEX selective	*n*‐HEX: 1.64	—	Breakthrough	—	275	Pore size, shape	[654]
	**BTAPa‐CF_3_**, 3D	6.3 × 6.3, 14.5 × 14.5	*n*‐HEX/3MP/22DMB/23DMB	*n*‐HEX selective	—	—	GC	—	953	Pore size	[655]
	**COK‐18**, 3D (373K)	11 × 11	*n‐*HEX/2MP/3MP/23DMB/22DMB	*n*‐HEX selective	—	—	GC	—	800	Polarizability	[656]
	**Zn(bdc)(4‐4′‐bpy)_0.5_** (**MOF‐508a**), 3D	4 × 4	*n‐*HEX/2MP/22DMB	*n*‐HEX selective	—	—	GC	—	553.7 [657]	Pore size, shape	[658]
	**Zn(bdc)(dabco)_0.5_**, 3D	7.5 × 7.5, 3.8 × 4.7	*n‐*HEX/3MP/22DMB	*n*‐HEX selective	—	—	Breakthrough	—	1450 [41]	Pore size, Van der Waals interactions	[659]
	**Al‐bttotb**, 3D (303K)	5.6^f^	*n‐*HEX/2MP/3MP/22DMB/23DMB	*n*‐HEX selective	*n‐*HEX: 1.75 3MP: 1.09 23DMB: 0.49	—	Breakthrough	—	572	Pore size	[660]
	**Zr_6_O_4_(OH)_4_(bptc)_3_**, 3D (423K)	4.5 × 4.5, 12 × 12	*n‐*HEX/3MP/23DMB	*ns*‐HEX selective	*n‐*HEX: 1.51 3MP: 0.17 23DMB: 0.03	48 (*n‐*HEX)	Breakthrough	—	1030	Van der Waals interactions	[626]
	**Zr_6_O_4_(OH)_8_(H_2_O)_4_(abtc)_2_**, 3D (423K)	7 × 7	*n‐*HEX/3MP/23DMB	*n*‐HEX selective	*n‐*HEX: 1.21 3MP: 1.04 23DMB: 0.52	—	Breakthrough	—	1318	Van der Waals interactions	[626]
	**HIAM‐302**, 3D	7.6 × 7.6	*n‐*HEX/3MP/22DMB	*n*‐HEX selective	*n‐*HEX: 1.91 3MP: 1.09 22DMB: 0	—	Breakthrough	—	388	Van der Waals interactions	[661]
	**ZIF‐8**, 3D	3.4, 11.4	*n‐*HEX/2MP/22DMB/23DMB	*n*‐HEX selective	—	—	GC	—	—	Van der Waals interactions	[662]
	**MIL‐140A**, 3D	3.2 × 3.2	*n‐*HEX/2MP/3MP/22DMB/23DMB	*n*‐HEX selective	—		Breakthrough	—	423	Van der Waals interactions	[663]
	**MIL‐140B**, 3D	4.0 × 4.0	*n‐*HEX/2MP/3MP/22DMB/23DMB	*n*‐HEX selective	—	*n‐*HEX: 54.6 2MP: 51.2 3MP: 47.5 23DMB: 44.4 23DMB: 41.3	Breakthrough	—	472	Van der Waals interactions	[663]
	**MIL‐140C**, 3D	5.7 × 5.7	*n‐*HEX/2MP/3MP/22DMB/23DMB	*n*‐HEX selective	—		Breakthrough	—	782	Van der Waals interactions	[663]
	**[Zn_2_(PdTCPP)(Bpa)]_n_**, 3D (333K)	5.7 × 9.8	*n‐*HEX/3MP	*n*‐HEX selective	*n‐*HEX: 1.39 3MP: 0	—	Breakthrough	—	—	Flexibility	[664]
	**Ca(H_2_tcpb)**, 3D (393K)	5.0‐6.2	*n‐*HEX/3MP/22DMB	*n*‐HEX selective	*n*‐HEX: 1.02 3MP: 0	—	Breakthrough	—	220	Molecular size	[642]
	**ZIF‐8**, 3D	11.6, 3.4 [573]	*n*‐HEX/2MP/3MP/22DMB/23DMB	*n*‐HEX selective	—	—	Breakthrough	—	—	Kinetic	[665]
	**ZIF‐8**, 3D	11.6, 3.4	*n*‐HEX/3MP/22DMB	*n*‐HEX selective	—	—	Breakthrough	—	1950	Kinetic, diffusion	[666]
	**Zr‐MIL‐140B**, 3D (343K)	3.8 × 3.8	*n*‐HEX/2MP/3MP/22DMB/23DMB	*n*‐HEX selective	*n*‐HEX: 0.25 2MP: 0.11 3MP: 0.08 22DMB: 0.02 23DMB: 0.03	*n*‐HEX: 55.1 2MP: 52.8 3MP: 44.3 22DMB: 40.1 23DMB: 43.5	Vapor sorption isotherms	—	—	Pore shape	[667]
	**UiO‐66**, 3D (343K‐473K)	7, 11, 8	22DMB/23DMB/3MP/*n*‐HEX	22DMB selective	*n*‐HEX: 0.87 3MP: 2.64 22DMB: 2.81 23DMB: 3.04^e^	—	Breakthrough	—	1050	Pore size	[668]
	**MIL‐160(Al)**, 3D	5.8 × 5.8	*n*‐HEX/2MP/3MP/22DMB/23DMB	*n*‐HEX selective	—	—	Breakthrough	—	—	Van der Waals interactions	[669]
	**CAU‐10‐H**, 3D (303K)	6.2, 11.6	*n*‐HEX/3MP/22DMB	*n*‐HEX selective	*n*‐HEX: 1.56 3MP: 1.10 22DMB: 0.63	—	Breakthrough	N_2_ flow at 423K	483	Van der Waals interactions	[670]
	**CAU‐10‐Br**, 3D (303K)	4.1, 11.6	*n*‐HEX/3MP/22DMB	*n*‐HEX selective	*n*‐HEX: 0.55 3MP: 0.13 22DMB: 0.05	—	Breakthrough	N_2_ flow at 423K	463	Van der Waals interactions	[670]
	**CAU‐10‐H/Br**, 3D (303K)	5.4, 11.6	*n*‐HEX/3MP/22DMB	*n*‐HEX selective	*n*‐HEX: 1.46 3MP: 0.70 22DMB: 0.09	—	Breakthrough	N_2_ flow at 423 K	362	Van der Waals interactions	[670]
	**[Zn_9_(tba)_9_(dabco)_3_]**, 3D	11.92 × 11.92	*n*‐HEX/3MP/22DMB/23DMB	*n*‐HEX selective	—	—	Breakthrough	—	1175	Shape sieving, shape, diffusion	[671]
	**JNU‐2**, 3D	7.4 ×7.4	*n*‐HEX/2MP/3MP/23DMB/22DMB	*n*‐HEX selective	—	—	Breakthrough	Vacuum at 473 K	—	Kinetics	[672]
	**[Zn_2_(*L*‐AlaPyr)_2_]**, 3D	—	*n*‐HEX/2MP/3MP/23DMB/22DMB	*n*‐HEX selective	—	—	NMR, GC	—	—	Pore shape	[673]
C6 cyclic isomers	**CoV‐FA‐TPA**, 3D	—	Bz/Cy	235.4	Bz: 2.47 Cy: 0.14	—	NMR	He flow at 333 K	887	π⋯π interactions	[674]
	**MFM‐68‐Cl_2_**, 3D	9.3	Bz/Cy	277	Bz: 4.62	Bz: 60	Breakthrough	N_2_ flow at 353 K	—	C‐H⋯π interactions	[675]
	**Ni_3_(OH)(Ina)_3_(BDC)_1.5_**, 3D	7^e^	Bz/Cy	145^g^	Bz: 2.9 Cy: 0.02	—	Vapor sorption isotherms	—	1255	π⋯π interactions	[676]
	**MAF‐7Br**, 3D	3.4 × 3.4	Bz/Cy	113	Bz: 2.55 Cy: 0.17	—	GC	—	—	Van der Waals interactions	[677]
	**MAF‐40**, 3D	8.2 × 10.2	Bz/Cy	40	Bz: 0.72 Cy: 0.46	—	GC	—	338	Kinetic	[678]
	**Mn‐MOF‐74**, 3D	11 × 11	Bz/Cy	37.5^g^	Bz: 9.38 Cy: 0.25	—	Vapor sorption isotherms	—	1500‐1600	UMCs	[679]
	**CUB‐30**, 3D	8, 18, 21	Cy/Bz	28.5	—	—	Breakthrough	—	2930	Pore size	[680]
	**Zn‐BDC**, 2D	5.3 × 5.3	Bz/Cy	22.6	Bz: 2.15 Cy: 0.1	—	Breakthrough	—	308	Pore size	[681]
	**[Cu(etz)]** (**MAF‐2**), 3D	9 × 9	Bz/Cy	21^g^	Bz: 10.5 Cy: 0.5	—	Vapor sorption isotherms	—	—	Flexibility, C‐H⋯π interactions	[682]
	**Cu‐1‐NO_3_**, 1D to 2D	5.7^j^	Bz/Cy	18.9^g^	Bz: 1.7 Cy: 0.09	—	Vapor sorption isotherms	—	—	C‐H⋯π, π⋯π interactions	[683]
	**[Zn(ip)(bpa)]_n_** (**CID‐23**), 3D	8.6 × 8.6	Bz/Cy	13.3^g^	Bz: 1.2 Cy: 0.09	—	Vapor sorption isotherms	—	553^h^	Flexibility, pore size	[684]
	**LiZn‐bdc‐bpy**, 3D	7 × 7	Bz/Cy	12.6^g^	Bz: 2.89 Cy: 0.23	—	Vapor sorption isotherms	—	742	C‐H⋯π, π⋯π, C‐H⋯O interactions	[685]
	**MAF‐24 β**, 3D	3.3 × 3.3	Bz/Cy	10.8^g^	Bz: 10.24 Cy: 0.95	—	Vapor sorption isotherms	—	444^h^	Pore size, C‐H⋯π interactions	[686]
	**DAT‐MOF‐1**, 3D	6.71 × 7.08	Bz/Cy	8.4^g^	Bz: 1.51 Cy: 0.18	—	Vapor sorption isotherms	—	—	π‐electron deficient	[687]
	**Cd‐ATAIA**, 3D	11.3 × 5.92, 11.99 × 20.08, 5.21 × 4.91	Bz/Cy	6.7^g^	Bz: 2.36 Cy: 0.35	—	Vapor sorption isotherms	—	62	π‐electron deficient	[688]
	**Cd‐dtztp**, 3D	10.0‐15.9^f^	Bz/Cy	6.0^g^	Bz: 6.52 Cy: 1.09	—	Vapor sorption isotherms	—	575.8	Pore size, C‐H⋯π, π⋯π interactions	[689]
	**ZnL**,^j^ 3D	1.75 × 3.65, 0.32 × 2.22	Bz/Cy	5.1^g^	Bz: 5.6 Cy: 1.1	—	Vapor sorption isotherms	—	—	C‐H⋯π interactions	[690]
	**[Zn(μ_4_‐TCNQ−TCNQ)bpy]**, 3D	10.1 × 10.1	Bz/Cy	4^g^	Bz: 3.6 Cy: 0.9	—	Vapour sorption isotherms	—	—	Size, C‐H⋯π interactions	[62]
	**ZnL′**,^k^ 3D	9.11	Bz/Cy	3.3^g^	Bz: 1.4 Cy: 0.45	—	Vapor sorption isotherms	—	—	π⋯π interactions	[691]
	**CUB‐5**, 3D	12^b^	Bz/Cy	2.7	—	—	Vapor sorption isotherms	—	3107	C‐H···π interactions	[692]
	**MUF‐77**, 3D	8.2, 17.5, 20.5	Cy/Bz	2.45	—	—	Breakthrough	—	3604	Pore size	[680]
	**3DL‐MOF‐1** (**[Zn_4_O(pdc)_3_]**), 3D	11^b^	Bz/Cy	2.3	—	—	Vapor sorption isotherms	—	2378	C‐H···π interactions	[692]
	**Mn(TCNQ−TCNQ)bpy**, 3D	10.1 × 10.1	Bz/Cy	2.2^g^	Bz: 3.7 Cy: 1.7	—	Vapor sorption isotherms	—	—	C‐H⋯π interactions	[693]
	**Cu_2_I_2_(BTTP4)]**, 3D	10 × 10	Bz/Cy	2.2^g^	Bz: 2.6 Cy: 1.2	—	Vapor sorption isotherms	—	496^i^	π⋯π interaction	[694]
	**CUB‐5**, 3D	7, 12^f^	Bz/*n*‐HEX/2MP/23DMB	Bz selective	Benzene: 7.3 *n*‐HEX: 4.9 2MP: 5.1 23DMB: 4.7	—	Breakthrough	—	2614	C‐H···π interactions	[73]
	**BUT‐53**, 3D	7.8 × 7.8	Bz/air	Trace Bz capture	—	—	Breakthrough	N_2_ flow at 393 K	811	C‐H···π, C‐H···N interactions	[695]
	**BUT‐54**, 3D	10.0 × 10.0	Bz/air	Trace Bz capture	—	—	Breakthrough	N_2_ flow at 393 K	1128	C‐H···π, C‐H···N interactions	[695]
	**BUT‐55**, 3D	8.0 × 8.0	Bz/air	Trace Bz capture	—	—	Breakthrough	N_2_ flow at 393 K	873	C‐H···π, C‐H···N interactions	[695]
	**BUT‐56**, 3D	8.0 × 8.0	Bz/air	Trace Bz capture	—	—	Breakthrough	N_2_ flow at 393 K	897	C‐H···π, C‐H···N interactions	[695]
	**BUT‐57**, 3D	11.5 × 11.5	Bz/air	Trace Bz capture	—	—	Breakthrough	N_2_ flow at 393 K	970	C‐H···π, C‐H···N interactions	[695]
	**BUT‐58**, 3D	8.0 × 8.0	Bz/air	Trace Bz capture	—	—	Breakthrough	N_2_ flow at 393 K	849	C‐H···π, C‐H···N interactions	[695]
	**MIL‐125‐Zn**, 3D		Bz/air	Trace Bz capture	—	—	Breakthrough	N_2_ flow at 353 K	—	UMCs, electrostatics	[696]
	**2‐bpe‐Zn**, 3D	12.76 × 8.39	Bz/Cy	—	Bz: 5.5 Cy: ∼0	—	Vapor sorption isotherms	—	—	π⋯π interactions	[697]
	**1‐bpy‐Zn**, 3D	10.49 × 8.34	Bz/Cy	—	Bz: 2.32 Cy: ∼0	—	Vapor sorption isotherms	—	—	π⋯π interactions	[697]
	**MFOF‐1**, 3D	7.2, 10.3^b^	Bz/Cy	1.5^g^	Bz: 20.38 Cy: 13.18	—	Vapor sorption isotherms	—	2287	π‐electron deficient	[698]
	**IPM‐MOF‐110**, 3D	6.17 × 7.68	Bz/Cy	—	Bz: 4.57 Cy: ∼0	—	Vapor sorption isotherms	—	937	π‐electron deficient	[699]
	**Cu‐terpy‐I**, 3D	10.56 × 7.32	Bz/Cy	—	Bz: 2.77 Cy: ∼0	—	Vapor sorption isotherms	—	—	C‐H⋯π, π⋯π interactions	[700]
	**Cu‐bpp‐BF_4_**, 3D	—	Bz/Cy	—	Bz: 11 Cy: ∼0	Bz: 50	Vapor sorption isotherms	—	14	Flexibility, C‐H⋯π interactions	[701]

^a^ IAST Selectivity calculated from mixtures of binary (v/v = 1/1) C6 compounds.

^b^ Selectivity calculated based on binary breakthrough curves.

^c^ Capacity of weight precent based on breakthrough data (wt%).

^d^ Calculated selectivity by binary of 50:50 *n‐*HEX and its branched isomer.

^e^ BET surface area was calculated by Ar sorption isotherm.

^f^ Pore size and BET surface area were calculated by N_2_ isotherm at 77K.

^g^ Uptake ratio.

^h^ Langmuir surface area.

^i^ Pore size and BET surface area calculated from CO_2_ sorption at 195K.

^j^ Separation coefficient. ^j^ L = (*R*,*R*)‐(−)‐*N*,*N*′‐bis(3‐*tert*‐butyl‐5‐(4‐ethynylpyridyl)salicylidene)‐1,2‐diaminocyclohexane.

^k^ L′ = 1,2,4,5‐Benzene tetracarboxylic anhydride.

## 3 HC Separation in the Gas Phase

Under ambient conditions (298 K, 1 bar), most of the light HCs (C1‐C4 HCs, i.e., CH_4_, C_2_H_2_, C_2_H_4_, C_2_H_6_, C_3_H_4_, C_3_H_6_, C_3_H_8_, C_4_H_6_, C_4_H_8_, and C_4_H_10_, and their isomers) exist in the gas phase, the others being volatile liquids. Their utility in the commodity chemicals sector requires a high level of purity, which in turn requires energy‐ and capital‐intensive separation technologies. Herein, we highlight the potential of physisorbents to enable adsorptive separation of C1–C4 HCs by focusing upon the nature of the binding sites in the best‐performing physisorbents in the context of purifying a specific HC from a binary mixture or a multicomponent feed gas stream.

### 3.1 Methane Separation

CH_4_ offers advantages over other fossil fuels, thanks to its ample natural reserves, low cost, and high fuel value of 57.1 kJ/mol [105]. However, CH_4_ is primarily obtained from natural gas (NG), which is typically contaminated with impurities such as carbon dioxide (CO_2_) and nitrogen (N_2_), among others. These impurities can lower its heating value and energy content. Selective separation of CH_4_ from mixtures containing CO_2_ or N_2_ can therefore enhance the calorific value of low‐quality NG. NG sweetening (purification) is relevant herein as sorbents tend to strongly adsorb (and thereby remove) acidic gases like H_2_S and CO_2_ from NG [702]. Moreover, biogas prepared by anaerobic decomposition of organic waste is primarily composed of CH_4_ and CO_2_ [703].

For adsorptive purification of CH_4_, adsorbents can be categorized into three groups: (1) CH_4_–selective adsorbents, which exhibit stronger interactions for CH_4_ over N_2_, separation being typically driven by thermodynamic equilibrium. As detailed above, in 1997, Kondo et al. reported the first example of a 3D MOF with permanent porosity; CH_4_/N_2_ separation selectivity was observed [28]. (2) N_2_‐selective adsorbents, which selectively adsorb N_2_ over CH_4_, performance typically enabled by UMCs or sieving. In 2007, a prototypical MOF for N_2_/CH_4_ separation was reported by S. Ma et al. by utilizing a molecular sieving mechanism [704]. (3) CO_2_‐selective adsorbents offer a higher affinity for CO_2_ than CH_4_, typically driven by sorbate‐sorbent interactions enhanced by control of pore chemistry and pore size. Zaworotko and co‐workers reported that HUMs such as **SIFSIX‐3‐Zn** offer optimal pore size and favorable electrostatic interactions from inorganic anions, which are highly effective for trace CO_2_/CH_4_ separation by offering an order of magnitude improvement in selectivity versus previous benchmark reticular sorbents for this separation [33].

#### 3.1.1 CH_4_‐Selective Separation (CH_4_/N_2_)

Separating CH_4_ from N_2_ is a challenge due to their similar kinetic diameters (CH_4_: 3.80 Å and N_2_: 3.64 Å) and comparable polarizabilities (CH_4_: 26.0 × 10^−25^ cm^3^ and N_2_: 17.6 × 10^−25^ cm^3^) (Table 2). In adsorptive separation, as both N_2_ and CH_4_ are nonpolar molecules, most CNs tend to selectively adsorb CH_4_ over N_2_ because of the slightly higher polarizability of CH_4_, but typically with relatively low selectivity (Table 3). Table 3 presents the leading reticular sorbents reported to date for CH_4_/N_2_ and N_2_/CH_4_ separation, arranged in decreasing order of adsorption selectivity. Table 3 reveals little correlation between selectivity and pore size, surface area, or single‐component gas uptake. The mechanisms for the six top‐performing CH_4_‐selective reticular sorbents are primarily attributed to Van der Waals interactions, while all N_2_‐selective sorbents are driven by UMC binding sites. The current top‐performing sorbents are discussed below.

**FIGURE 1:**
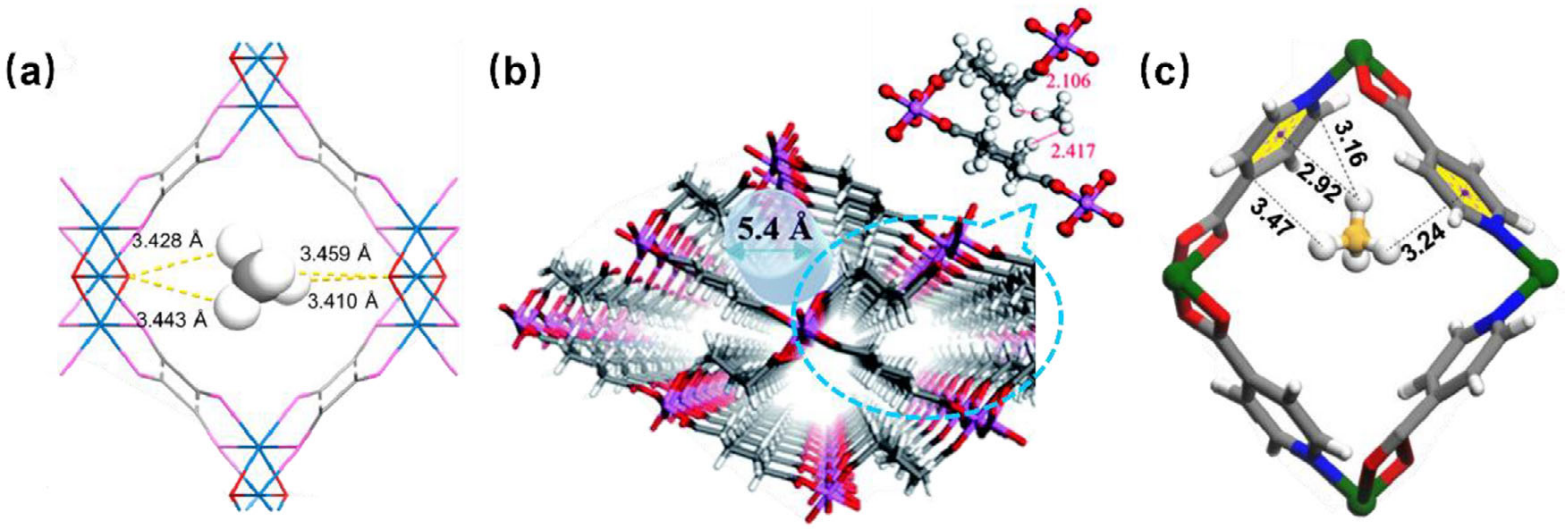
Examples of top‐performing reticular sorbents for CH_4_/N_2_ separation based upon selectivity: (a) **[Co_3_(C_4_O_4_)_2_(OH)_2_]** [89] (b) **Al‐CDC** [88] (c) **Ni(ina)_2_** [87] with feed ratio, temperature, and total pressure set at 1:1, 298 K, and 1 bar, respectively. (Reprinted with permission from ref. [89]: copyright 2018, American Institute of Chemical Engineers; ref. [88]: Copyright 2020, The Royal Society of Chemistry; ref. [87]: copyright 2022, Wiley‐VCH GmbH.).

In 2018, Bao's group synthesized an ultramicroporous CN featuring a pore size of 4.1 × 4.3 Å^2^, **[Co_3_(C_4_O_4_)_2_(OH)_2_]** (C_4_O_4_^2−^  =  squarate) (Figure 1a) [89]. Thanks to the binding between CH_4_ molecules and hydroxo groups that line the pores of **[Co_3_(C_4_O_4_)_2_(OH)_2_]**, an IAST CH_4_/N_2_ selectivity of 12.5 and CH_4_ uptake of 0.4 mmol/g at 298 K were achieved.

In 2020, a family of CNs known as **M‐CDC** (CDC = *trans*‐1,4‐cyclohexanedicarboxylic acid) featuring various metal centers demonstrated impressive separation performance for CH_4_/N_2_ mixtures (Figure 1b) [88]. **Al‐CDC**, in particular, exhibited IAST selectivity of 13.1 at 298 K and CH_4_ uptake of 1.43 mmol/g, a result supported by dynamic column breakthrough experiments. Computational studies indicated that strong van der Waals interactions between pore walls and CH_4_ molecules were enabled by confinement.

In 2022, Q. Yang and colleagues reported a new benchmark for CH_4_/N_2_ IAST selectivity (15.8) in **Ni(ina)_2_** (Figure 1c, feed ratio = 1:1, temperature: 298 K, total pressure: 1 bar) [87]. **Ni(ina)_2_**, featuring a pore size of 5.0 × 5.8 Å^2^, also exhibited exceptional CH_4_ uptake of 1.82 mmol/g at 298 K. Theoretical calculations and analysis of the CH_4_‐loaded single‐crystal structures revealed a CH_4_‐selective binding site rich with C─H···π interactions. With favorable thermal and moisture stability, low cost, and scalability, **Ni(ina)_2_** is therefore a promising candidate for further development.

#### 3.1.2 N_2_‐Selective Separation (N_2_/CH_4_)

Whereas CH_4_ selectivity over N_2_ is the most common situation, utilizing CH_4_ would necessitate an additional desorption process. There are, however, CNs that preferentially adsorb N_2_ over CH_4_ by relying upon reticular sorbent‐sorbate interactions (Table 3).

**FIGURE 2:**
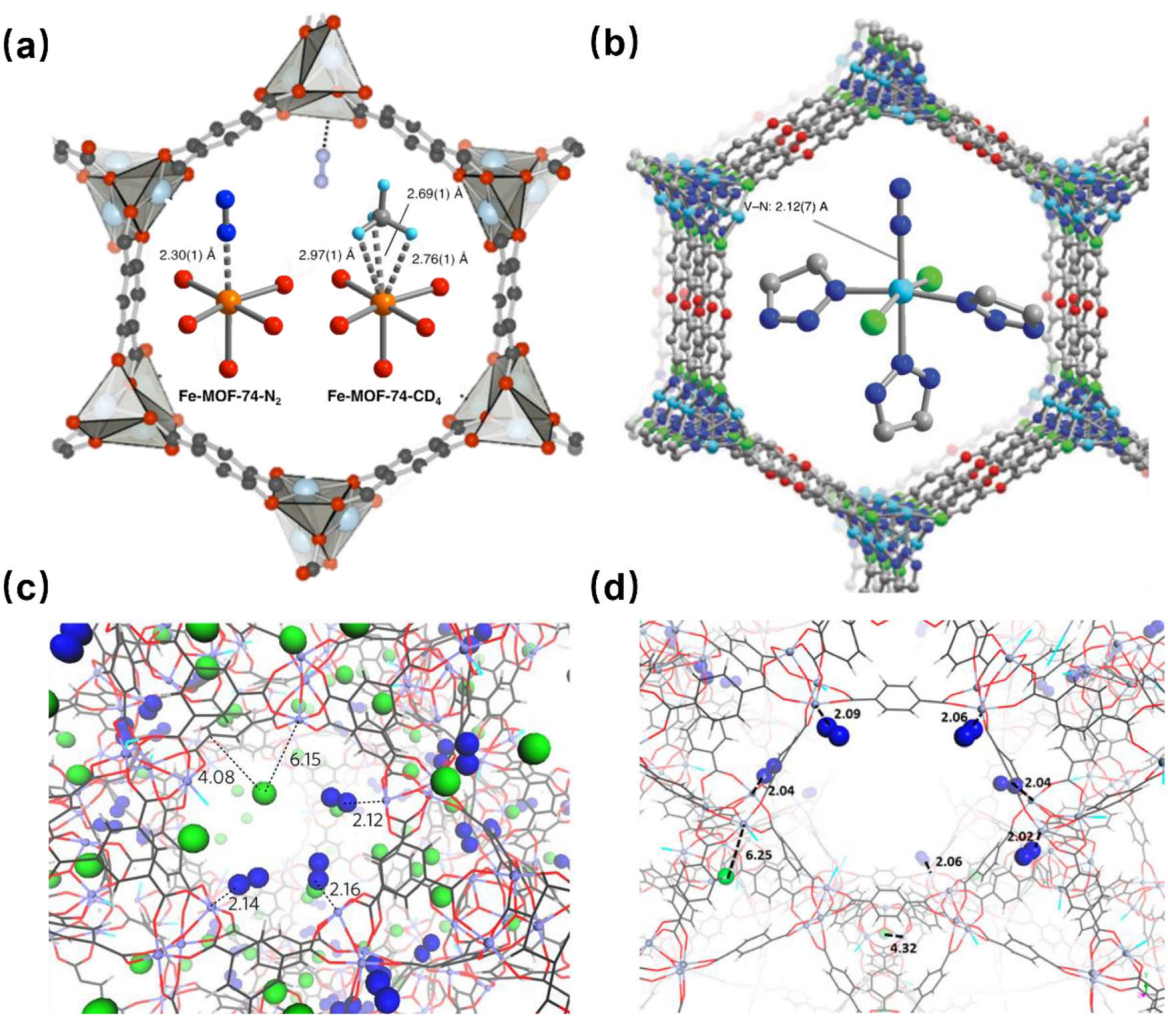
Examples of top‐performing N_2_/CH_4_ separating reticular sorbents that exhibit high selectivity for N_2_ over CH_4_: (a) **V‐MOF‐74** [122] (b) **V(II)‐MOF** [123] (c) **MIL‐100(Cr)** [124] (d) **MIL‐101(Cr)** [127] with feed ratio, temperature, and total pressure set at 1:1, 298 K, and 1 bar, respectively. (Reprinted with permission from ref. [122]: Copyright 2013, American Chemical Society; ref. [123]: Copyright 2020, Springer Nature; ref. [124]: Copyright 2017 Springer Nature; ref. [127]: Copyright 2017, American Chemical Society.).

In 2014, Long and colleagues predicted the potential of **V‐MOF‐74** for separating N_2_ from CH_4_ through quantum‐mechanical computations. This prediction was based on the presence of selective back‐bonding interactions between the unsaturated vanadium ions in **V‐MOF‐74** and the empty π* orbitals of N_2_ (Figure 2a) [122]. In 2020, they successfully synthesized another MOF with exposed V(II) sites, which facilitated the formation of back‐bonding interactions with weakly π‐acidic N_2_ [123]. The separation performance for N_2_/CH_4_ mixtures by **V(II)‐MOF** revealed an exceptionally high N_2_/CH_4_ IAST selectivity at lower N_2_ concentrations (72 at an N_2_:CH_4_ volumetric ratio of 2:98). Introducing π‐basic metal centers into porous adsorbents is likely to be an effective general design strategy for selective capture of π‐acidic molecules. (Figure 2b).

In 2017, drawing inspiration from biomimetic and metal‐dinitrogen chemistry, Chang and co‐workers reported a mesoporous N_2_‐selective CN, **MIL‐100(Cr)**, featuring accessible Cr(III) sites. **MIL‐101(Cr)** demonstrated a capacity to selectively capture N_2_ over CH_4_ and O_2_ based on thermodynamic equilibrium (Figure 2c) [124]. The calculated IAST selectivity was 8, and N_2_ uptake was determined to be 1.64 mmolg^−1^ at 298 K. The N_2_‐selective binding mechanism was elucidated through a combination of experimental and computational studies, which suggested that strong binding affinity between N_2_ and the unsaturated Cr(III) sites drives separation performance. Similarly, in 2017 and 2021, two isostructural CNs, **MIL‐101(Cr)** [127] and **MIL‐101(Cr)‐NO_2_** [126], respectively, were reported to offer excellent N_2_/CH_4_ separation efficiencies (Figure 2d). In both cases, the separation performance was attributed to N_2_ selective interactions enabled by the unsaturated Cr(III) sites.

#### 3.1.3 CO_2_‐Selective Separation (CO_2_/CH_4_)

Thanks to the difference in kinetic diameters between CO_2_ (3.3 Å) and CH_4_ (3.8 Å), molecular sieving could be feasible for the separation of CO_2_ from CH_4_. Further, the difference in polarizability between CO_2_ (29.11 × 10^−25^ cm^3^) and CH_4_ (26 × 10^−25^ cm^3^), although small, favors CO_2_ (Table 4). Table 4 presents the leading reticular sorbents reported to date for CO_2_/CH_4_ separation, arranged in decreasing order of adsorption selectivity. The sorbents with relatively high selectivity in Table 4 were found to have pore sizes ranging from 3 to 4 Å. Moreover, molecular sieving, as noted in Table 4, facilitated the highest selectivity values for CO_2_/CH_4_ separation. The *Q*_st_ values for CO_2_ of the top‐performing sorbents with a molecular sieving mechanism are generally below 40 kJmol^−1^, resulting in mild regeneration conditions (inert gas flow at 298 K or under vacuum). However, the CO_2_ uptake in the ten top‐performing reticular sorbents (with pore sizes of around 3.6 Å) is 5.3 mmol/g, whereas for sorbents with larger pores of around 10.2 Å, the CO_2_ uptake increases to 8.61 mmol/g. Nonetheless, due to the trade‐off [81], the separation performance of the large poor is poor, with a selectivity of only 8. Four representative examples with high selectivity and optimal pore sizes (3–4 Å) are discussed below to provide insight into design principles for CO_2_‐selective sorbents.

**FIGURE 3:**
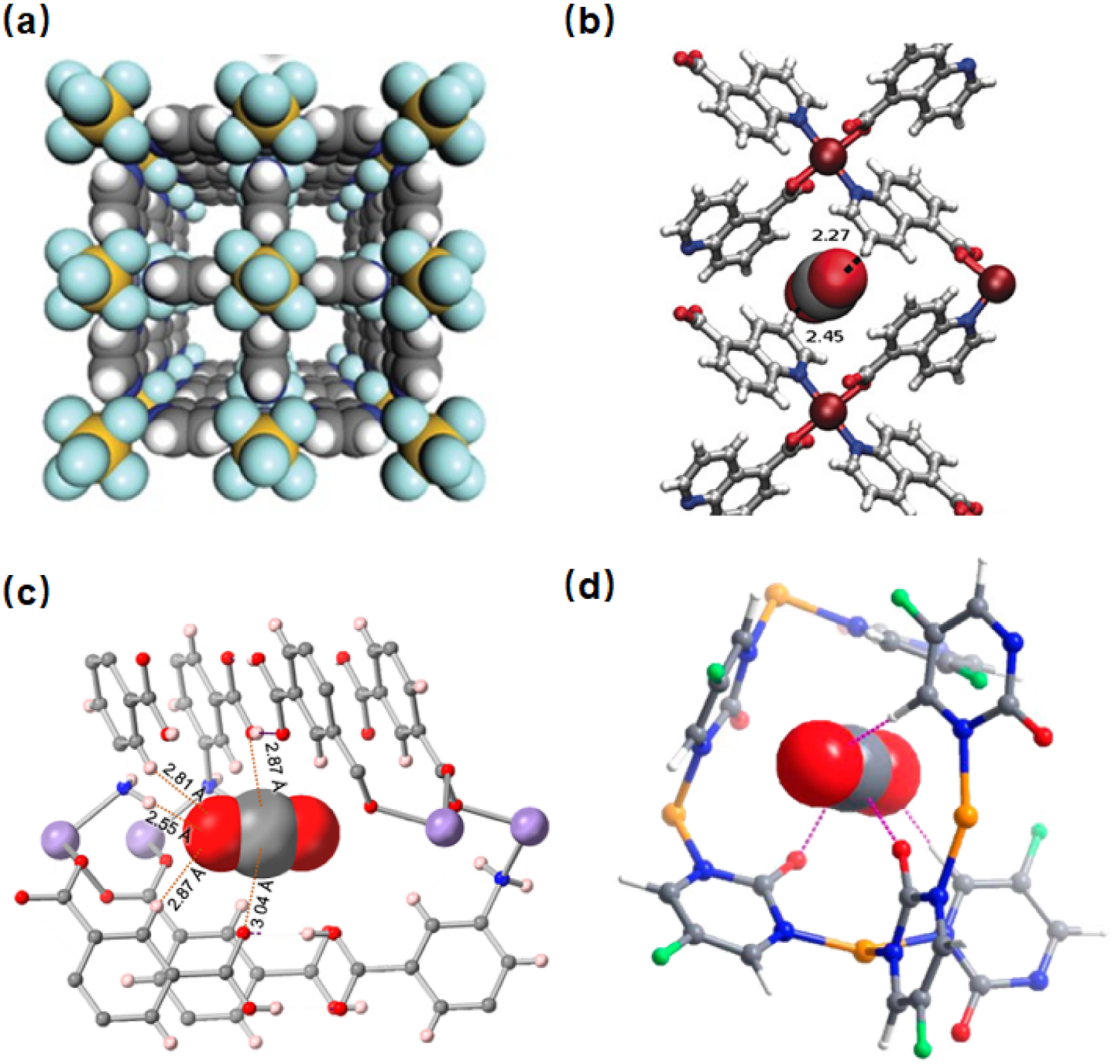
Examples of adsorbents that offer high CO_2_/CH_4_ selectivity: (a) **SIFSIX‐3‐Zn** [33] (b) **Qc‐5‐Cu‐sql‐α** [130] (c) **MUF‐16** [129] (d) **Cu‐F‐pymo** [128] with feed ratio, temperature, and total pressure set at 1:1, 298 K, and 1 bar, respectively. (Reprinted with permission from ref. [130]: copyright 2016, WILEY‐VCH Verlag GmbH & Co. KGaA, Weinheim; ref. [129]: copyright 2021, Springer Nature; ref. [128]: copyright 2022, Elsevier B.V.).

In 2013, Zaworotko's group reported three hybrid CNs, **SIFSIX‐2‐Cu** (pore size 13.05 Å), **SIFSIX‐2‐Cu‐i** (5.15 Å), **SIFSIX‐3‐Zn** (pore size 3.84 Å), with **SIFSIX‐3‐Zn** being the parent of the first family of physisorbents with ultrahigh (>1000 at ≤10 000 ppm) trace CO_2_ selectivity (Figure 3a) [33]. The use of crystal engineering to fine‐tune pore chemistry and size in this family of HUMs with coordinatively saturated metal centers and periodically arrayed hexafluorosilicate (SIFSIX) anions enables a “*sweet spot*” of kinetics and thermodynamics that offers high volumetric uptake even at low CO_2_ partial pressure (0.0004‐0.15 bar).

In 2016, Zaworotko and colleagues reported on the sorption properties of 2‐fold interpenetrated **dia**, **Qc‐5‐M‐dia** (M = Co, Ni, Zn, and Cu; CN), and **sql** topology, **Qc‐5‐Cu‐sql‐α**, CNs (Figure 3b) [130]. **Qc‐5‐Cu‐sql‐α**, with 3.3 × 3.3 Å^2^ pores, enabled molecular sieving and high CO_2_/CH_4_ IAST selectivity of 3300, coupled with CO_2_ uptake of 2.16 mmol/g at 298 K. **Qc‐5‐Cu‐sql‐α** also exhibited stability in the presence of moisture and maintained its separation efficiency even in the presence of water vapor.

In 2021, Telfer's group studied a series of robust and scalable CNs, [M(Haip)_2_] (M = Co, Ni, Mn; H_2_aip = 5‐aminoisophthalic acid), **MUF‐16(Co)** (**MUF‐16**), **MUF‐16(Mn)**, and **MUF‐16(Ni)**, featuring 3.6 × 7.6 Å^2^ pores (Figure 3c) [129]. **MUF‐16** and **MUF‐16(Ni)** exhibited high IAST selectivity values of 6690 and 1220, respectively, towards equimolar CO_2_/CH_4_ mixtures, and CO_2_ uptake as high as 2.13 mmol/g at 298 K. Computational studies revealed strong interactions between CO_2_ and pore walls facilitated by hydrogen bonds formed between the oxygen atoms of CO_2_ molecules and amino/phenyl groups.

In 2022, a CO_2_/CH_4_ IAST selectivity of >10^7^ was reported for **Cu‐F‐pymo** (Figure 3d) [128]. The observed molecular sieving effect was attributed to ultramicropores of 3.3 Å diameter. Computational analyses revealed that the oxygen moieties distributed along the pore walls enhanced binding affinity toward CO_2_ through electrostatic and hydrogen‐bonding interactions. Cycling dynamic column breakthrough experiments validated the CO_2_ separation performance and revealed a dynamic CO_2_ capture capacity of 1078 mmol/kg for an equimolar CO_2_/CH_4_ (v/v = 1/1) mixture.

To enable C1 separations, i.e., CH_4_/N_2_, N_2_/CH_4_, CO_2_/CH_4_, nine mechanisms have been identified, either as a single mechanism or a combination of two or more mechanisms: C─H···π interactions; van der Waals interactions; electrostatics; H‐bonding; polarizability; UMCs; flexibility; kinetics; pore size (molecular sieving). We note that supramolecular chemistry or “chemistry beyond the molecule” relies on non‐covalent interactions to enable the formation of larger, organized structures and that it encompasses a wide range of non‐covalent forces [705]. Thinking “supramolecularly” can provide insight and design rules to advance the functionality of MOFs for gas adsorption and separation. In particular, by exploiting host–guest interactions, the pore structure of MOFs can be engineered to yield materials with superior adsorption capacity and selectivity compared to conventional adsorbents.

**FIGURE 4:**
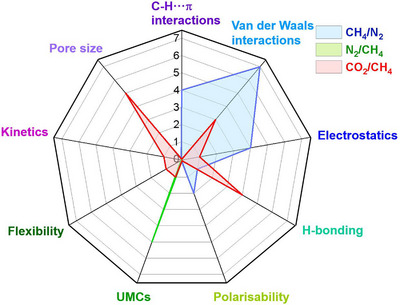
Radar plot illustrating the relative contributions of nine separation mechanisms among the 15 top‐performing CH_4_/N_2_ and CO_2_/CH_4_ selective adsorbents as well as five adsorbents that exhibit N_2_/CH_4_ adsorption selectivity. Every concentric decagon denotes an individual sorbent, with the central decagon symbolizing one sorbent.

The contributions of these mechanisms in the top‐performing adsorbents for CO_2_/N_2_, N_2_/CO_2_, and CO_2_/CH_4_ binary separation are collated as a radar plot in Figure 4.

For CH_4_/N_2_ separation, the higher polarizability of CH_4_ over N_2_ and, especially, van der Waals interactions (tight binding sites in confined space), were observed with the highest frequency. Meanwhile, the presence of negatively charged atoms when coupled with the right pore size can further enhance CH_4_ selectivity. Conversely, for N_2_/CH_4_ separation, the difference in polarizability seems to be of little relevance. Rather, UMCs were observed to play a prominent role in N_2_‐selective separation. Specifically, the unoccupied π* orbitals of N_2_ can interact with unsaturated metals that line pore walls. For CO_2_/CH_4_ separation, the relatively large difference in molecular sizes of CO_2_ (3.18 × 3.33 × 5.36 Å^3^) and CH_4_ (3.7 × 3.7 × 3.7 Å^3^) can enable molecular sieving mechanisms to afford ultra‐high CO_2_ selectivity, as exemplified by the pair of ideal molecular splitters, **Qc‐5‐Cu‐sql‐β** [130] and **SIFSIX‐14‐Cu‐i** [137]. Also, H‐bonding interactions between the pore walls and the oxygen atoms of CO_2_ were found to enhance CO_2_/CH_4_ separation in **MUF‐16** [129].

### 3.2 C2 Separations

C2 HCs are commodities used in the production of value‐added chemicals in the petrochemical industry [706]. Their production results in impurities, which must be removed. Adsorptive separation has emerged as an alternative to current purification technologies, thanks to its potential for reduced energy footprints. In a seminal discovery by the Kitagawa group in 2005, the 2D CN **CPL‐1** was reported to exhibit C_2_H_2_/CO_2_ selectivity of 26 and, therefore, offer potential utility in adsorptive separations [60]. In essence, this work introduced a new paradigm to separate binary gases with similar physicochemical properties, i.e., precise control of pore size and chemistry in PCNs. Several early studies involving structural flexibility and optimal pore size/chemistry were demonstrated for C_2_H_2_/C_2_H_4_ separation. In 2011, B. Chen's group reported adsorption selectivity of up to 5.2 for the narrow pore variants of a flexible PCN, [**Zn_3_(CDC)_3_{Cu(SalPycy)**}]*_n_* (**M′MOF‐3**, CDC = 1,4‐cyclohexanedicarboxylate) [241]. In 2016, the groups of H. Xing, B. Chen and Zaworotko reported that the rigid physisorbent **SIFSIX‐2‐Cu‐i** exhibits C_2_H_2_ selectivity of 44.5 over C_2_H_4_ [35], an order of magnitude beyond the previous benchmark (feed ratio = 1:1, temperature: 298 K, total pressure: 1 bar). In 2018, the first example of a C_2_H_4_ sieve over C_2_H_6_, **UTSA‐280**, was reported by B. Chen's group. It exhibited ultrahigh adsorption selectivity of ≈ 4100 [287]. With respect to C_2_H_6_/C_2_H_4_ “inverse” separation, an azolate ultramicroporous material (AUM), **MAF‐49** (MAF: metal azolate framework) was reported to exhibit reverse selectivity for C_2_H_6_ by J. P. Zhang and X. M. Chen's group in 2015 [314]. Given the differences in separation mechanisms (molecular sieving, flexible sorbents that open only for specific sorbates, and rigid sorbents), comparing calculated selectivity should be regarded only as an indicator of separation performance under dynamic breakthrough conditions.

#### 3.2.1 C_2_H_2_‐Selective Separation

##### 3.2.1.1 C_2_H_2_/CO_2_ and C_2_H_2_/C_2_H_4_ Separation

C_2_H_2_ is an important feedstock in the chemical industry, serving as a precursor to a wide spectrum of commodity chemicals, including polyurethane and polyesters. However, industrial C_2_H_2_ production tends to entail elevated temperatures when it is produced by the breakdown of petroleum gas or the combustion of natural gas. This results in contaminants such as CO_2_ or C_2_H_4_ [707, 708, 709]. Since these gases exhibit similar physicochemical properties and comparable molecular sizes (Table 2), the production of high‐purity C_2_H_2_ from C_2_H_2_/CO_2_ and/or C_2_H_2_/C_2_H_4_ mixtures remains a challenge and has been subjected to in‐depth study (Tables 5 and 6) [710]. Tables 5 and 6 present the leading reticular sorbents thus far reported for C_2_H_2_/CO_2_ and C_2_H_2_/C_2_H_4_ separation, respectively, arranged in decreasing order of adsorption selectivity. The sorbents with the highest C_2_H_2_/CO_2_ and C_2_H_2_/C_2_H_4_ selectivity in Tables 5 and 6 were found to have pore sizes ranging from 3 to 5 Å, although small pore sizes do not always offer a guarantee of high selectivity. A typical trade‐off is evident in Tables 5 and 6: sorbents with larger pore sizes (11‐18 Å) tend to exhibit relatively high uptakes of C_2_H_2_, CO_2_, or C_2_H_4_ (up to 6.46 mmol/g for C_2_H_2_, 4.2 mmol/g for CO_2_, and 7.45 mmol/g for C_2_H_4_), but their selectivities are low (< 3.5). For C_2_H_2_‐selective sorbents in both tables, H‐bonding as a separation mechanism is frequently observed and can result in relatively high *Q*_st_(C_2_H_2_) and selectivity. Due to their structural characteristics, HUMs with optimal pore sizes and abundant H‐acceptor sites from SiF_6_^2−^ anions on the pore walls offer advantages over other reticular sorbents for C_2_H_2_‐selective sorption. Four representative examples (particularly HUMs) with high selectivity from both C_2_H_2_/CO_2_ and C_2_H_2_/C_2_H_4_ binary systems are discussed to illustrate the C_2_H_2_‐binding separation mechanisms (Tables 5, 6).

Reticular sorbents that can adapt their pore structures to better accommodate a specific guest molecule through an induced fit mechanism also offer potential for separation and purification applications. In 2019, D. Li and colleagues reported that the flexible MOF **JNU‐1** exhibited induced fit for C_2_H_2_ with a high adsorption enthalpy of 47.6 kJ mol^−1^ [160]. The diamond‐shaped channel presents UMCs that cooperatively bind to C_2_H_2_ molecules. Consequently, a high C_2_H_2_/CO_2_ selectivity of 285.6, along with a high C_2_H_2_ uptake of 2.9 mmol g^−1^ at 298 K, was determined.

**FIGURE 5:**
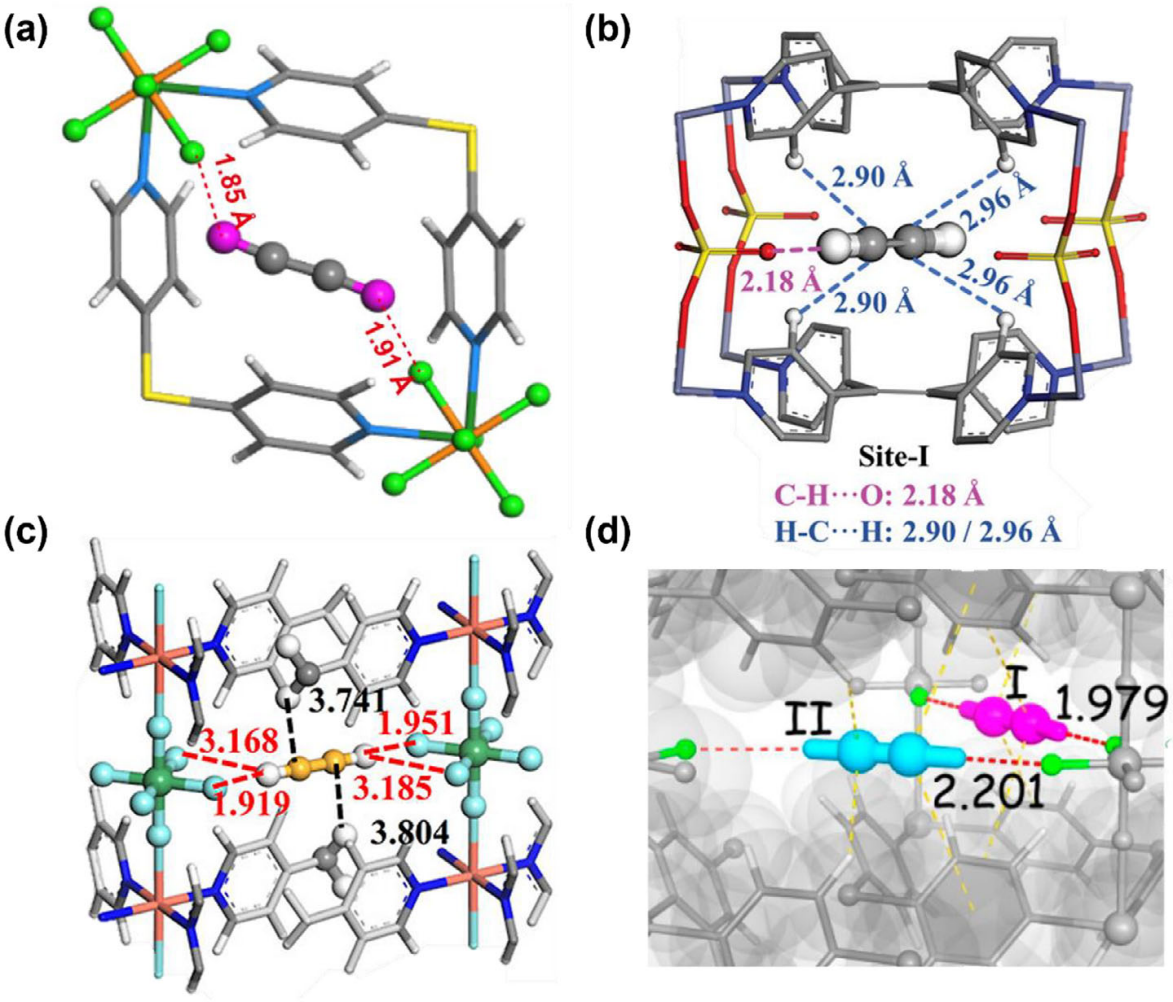
Examples of top‐performing reticular sorbents for C_2_H_2_/CO_2_ separation: (a) **SIFSIX‐dps‐Cu** [158], (b) **SOFOUR‐TEPE‐Zn** [156], (c) **ZUL‐330** [157], and (d) **UTSA‐300a** [159] with feed ratio, temperature, and total pressure set at 1:1, 298 K, and 1 bar, respectively. (Reprinted with permission from ref. [158]: copyright 2022, Springer Nature; ref. [156]: copyright 2023, Wiley‐VCH GmbH; ref. [157]: copyright 2023, American Chemical Society; ref. [159]: copyright 2023, American Chemical Society.).

In 2022, B. Chen and colleagues reported that the HUM [Cu(dps)_2_(SiF_6_)]*_n_* (**SIFSIX‐dps‐Cu**, SIFSIX = SiF_6_^2−^, dps = 4.4′‐dipyridylsulfide) exhibits a pore confinement effect (pore size = 1.4 × 3.0 Å^2^) that distinguishes between C_2_H_2_ and CO_2_ (Figure 5a) [158]. At 298 K, **SIFSIX‐dps‐Cu** adsorbed a high amount of C_2_H_2_ (4.57 mmol/g), and equimolar C_2_H_2_/CO_2_ IAST selectivity was determined to be 1787. Computational studies indicated multiple H‐bonding interactions between C_2_H_2_ and SiF_6_^2−^ within both inter‐ and intralayer spaces.

In 2023, taking inspiration from the prototypal sulfate‐pillared HUM, **SOFOUR‐1‐Zn**, **SOFOUR‐TEPE‐Zn** exploited 1,1,2,2‐tetra(pyridin‐4‐yl) ethene (TEPE) to create a negatively charged pore surface (Figure 5b) [156]. **SOFOUR‐TEPE‐Zn** exhibited high IAST selectivity of 16,833 and C_2_H_2_ uptake of 3.98 mmol/g. Computational studies indicated multiple H‐bonding interactions between C_2_H_2_ molecules and pore walls. Conversely, only weak van der Waals interactions were observed for CO_2_.

In 2023, two‐layered HUMs, **ZUL‐330** (SiFSIX‐dpm‐Cu, [Cu(4,4′‐dipyridylmethane)_2_(SiF_6_)]*_n_*) and **ZUL‐430** (GeFSIX‐dpm‐Cu, [Cu(4,4′‐dipyridylmethane)_2_(GeF_6_)]*_n_*) were reported by Q. Yang and co‐workers (Figure 5c). Compared to **ZUL‐430**, **ZUL‐330** exhibited high C_2_H_2_ uptake (5.91 mmol/g for **ZUL‐330**; 5.74 mmol/g for **ZUL‐430**) and outstanding C_2_H_2_/CO_2_ IAST selectivity (10086 for **ZUL‐330**; 3499 for **ZUL‐430**) [157]. Subtle structural differences in these sorbents resulted in significant differences in separation performance. Dispersion‐corrected density functional theory (DFT‐D) calculations were consistent with the superior separation performance of **ZUL‐330**. Specifically, H‐bonding and van der Waals interactions in **ZUL‐330** and **ZUL‐430** resulted in higher C_2_H_2_ binding energy for **ZUL‐330** (86.1 kJ/mol) than **ZUL‐430** (82.4 kJ/mol), and lower CO_2_ binding energy for **ZUL‐330** (44.5 kJ/mol) than **ZUL‐430** (45.8 kJ/mol).

**FIGURE 6:**
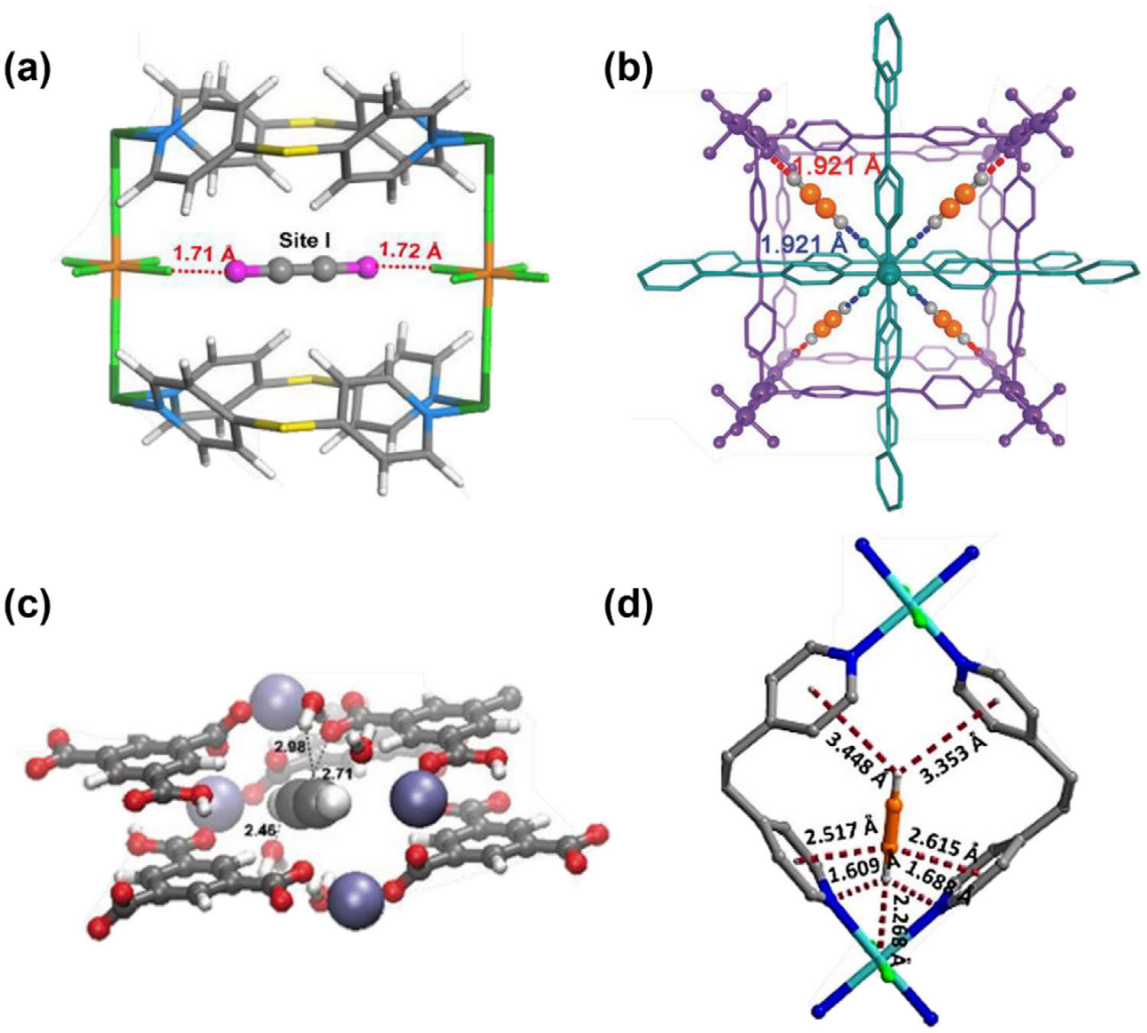
Examples of top‐performing reticular sorbents for C_2_H_2_/C_2_H_4_ separation with high selectivity: (a) **UTSA‐300‐Cu** [231], (b) **SIFSIX‐14‐Cu‐i** [39], (c) **bnn‐1‐Ca‐H_2_O** [232], (d) **sql‐SIFSIX‐bpe‐Zn** [237] with feed ratio, temperature, and total pressure set at 1:1, 298 K, and 1 bar, respectively. (Reprinted with permission from ref. [231]: copyright 2020, American Chemical Society; ref. [39]: copyright 2017, Wiley‐VCH GmbH; ref. [232]: copyright 2020, Wiley‐VCH GmbH; ref. [237]: copyright 2021, Wiley‐VCH GmbH.).

In 2017, B. Chen et al. reported a flexible layered HUM with 2.4 × 3.3 Å^2^ pores (open phase), [Zn(dps)_2_(SiF_6_)]*_n_* (**UTSA‐300**, dps = 4,4′‐dipyridylsulfide). **UTSA‐300** exhibited high IAST selectivity of 1000 for C_2_H_2_/CO_2_ and 10000 for C_2_H_2_/C_2_H_4_ (Figure 5d) [159]. Closed‐pore and open‐pore phases were structurally characterized for **UTSA‐300**. Strong C─H···F and π···π stacking interactions were observed in closed‐pore **UTSA‐300a**, resulting in the shrinkage of the structure. As pressure increased, C_2_H_2_ molecules were observed to bind to two hexafluorosilicate F atoms, thereby breaking intra‐network hydrogen bonds and enabling transformation to the open‐pore structure. As a result, **UTSA‐300a** was found to exhibit high C_2_H_2_ uptake (4.57 mmol/g) accompanied by exclusion of C_2_H_4_ and CO_2_ under ambient conditions. In 2020, B. Chen et al. reported an isostructural HUM, **UTSA‐300‐Cu**, [Cu(dps)_2_(SiF_6_)]*_n_* (also known as **NCU‐100**) [231]. Substitution from zinc(II) to copper(II) resulted in longer Cu‐F distances that expanded the closed pore cavities from 3.9 × 3.5 Å^2^ to 4.3 × 3.6 Å^2^ (Figure 6a). The Cu analog was found to offer more optimal binding with IAST C_2_H_2_/ C_2_H_4_ selectivity of 7291.3 (molecular sieving) and C_2_H_2_ uptake of 4.57 mmol g^−1^.

In 2017, the second generation (Gen‐2) HUM, **SIFSIX‐14‐Cu‐i** ([Cu(azpy)_2_(SiF_6_)]*_n_*, azpy = 4,4′‐azopyridine), was reported by Zaworotko, B. Chen, W. Zhou and Q. Ren et al. [39] The pore size of 3.4 Å was found to effectively block C_2_H_4_ molecules while adsorbing C_2_H_2_ (1.8 mmol g^−1^ at 298 K, Figure 6b)). An IAST C_2_H_2_/C_2_H_4_ selectivity of 6320 was observed, and high‐purity (99.9999%) C_2_H_4_ was produced during dynamic column breakthrough experiments.

In 2020, Zaworotko et al. reported Ca(HBTC)·H_2_O (H_3_BTC = trimesic acid), **bnn‐1‐Ca‐H_2_O**, an ultramicroporous MOF (3.4 Å) that exhibits a sieving effect for C_2_H_2_ and IAST selectivity of 6966.4 (Figure 6c) [232]. Computational studies indicated that the coordinated water molecules in **bnn‐1‐Ca‐H_2_O** enabled the binding of C_2_H_2_.

In 2021, Zaworotko et al. reported a 2D layered HUM with benchmark C_2_H_2_ affinity (isosteric heat of adsorption (*Q*_st_) of 67.5 kJ/mol). In this work, the title sorbent, **sql‐SIFSIX‐bpe‐Zn**, was found to exist in four phases: as‐synthesized (α); activated (β); two C_2_H_2_‐induced phases (β′ and γ) [237]. The high selectivity over C_2_H_2_ resulted from the induced fit of C_2_H_2_, resulting in a C_2_H_2_/C_2_H_4_ selectivity of 53.1 (feed ratio v/v = 1: 99, IAST, 298 K, total pressure: 1 bar). Structural insight revealed H‐bonding interactions between C_2_H_2_ and SiF_6_^2−^ on site I alongside C‐H···F and C‐C···π & C─H···π interactions at site II (Figure 6d).

#### 3.2.2 CO_2_‐Selective Separation (Inverse Separation)

##### 3.2.2.1 CO_2_/C_2_H_2_ Separation

To obtain pure C_2_H_2_, the design of C_2_H_2_‐selective sorbents requires a desorption step, which in turn increases energy consumption and operational expenses. Adsorbents that selectively capture CO_2_ instead of C_2_H_2_ would therefore be desirable as they could enable the direct production of high‐purity C_2_H_2_ during the adsorption process. In recent years, a number of CO_2_‐selective CNs with high CO_2_/C_2_H_2_ selectivity have been reported (Table 7). Table 7 presents the leading reticular sorbents reported to date for CO_2_/C_2_H_2_ separation, listed in decreasing order of adsorption selectivity. The sorbents with relatively high selectivity were found to feature pore sizes ranging from 3 to 8 Å. Upon analyzing the performance metrics in Table 7, it is evident that the sorbents with the highest CO_2_ selectivity over C_2_H_2_ primarily rely on H‐bonding between the sorbate and pore walls. The *Q*_st_ values for the top ten sorbents range from 25 to 51 kJ/mol, requiring relatively low regeneration energy. Five studies reporting reticular sorbents with high CO_2_/C_2_H_2_ selectivity are discussed below.

**FIGURE 7:**
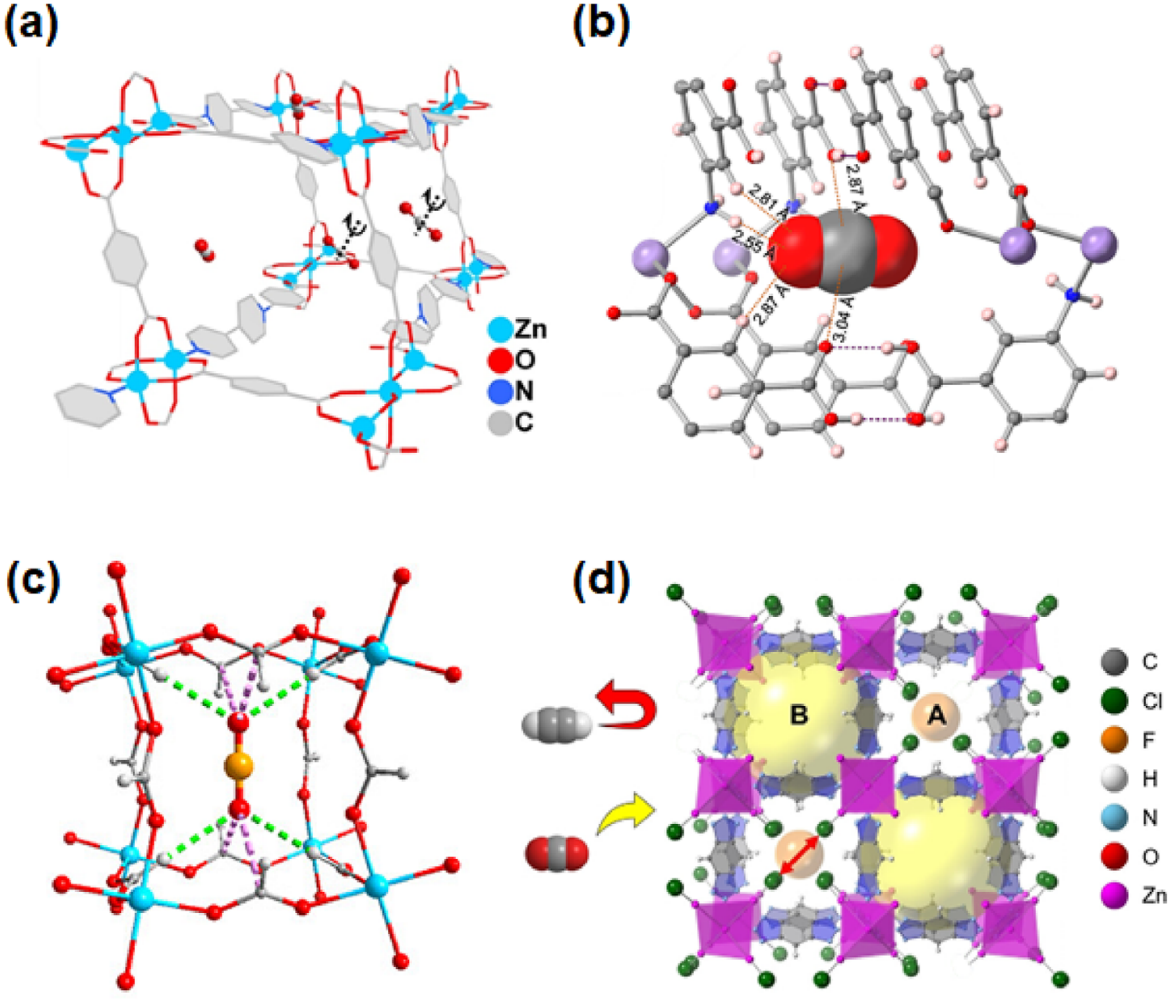
Examples of top‐performing reticular sorbents for CO_2_/C_2_H_2_ separation with high selectivity: (a) **PMOF‐1** [270], (b) **MUF‐16** [129], (c) **ALF** [266], and (d) **MFU‐4** [268] with feed ratio, temperature, and total pressure set at 1:1, 298 K, and 1 bar, respectively. (Reprinted with permission from ref. [270]: Copyright 2021, Wiley‐VCH GmbH; ref. [129]: Copyright 2021, Springer Nature; ref. [266]: Copyright 2023, American Chemical Society; ref. [268]: Copyright 2023, Wiley‐VCH GmbH.).

In 2021, Guo et al. reported a photoinduced electron transfer (PIET) strategy with **PMOF‐1**, which features a photochromically active bipyridinium zwitterion site. This site exhibits high CO_2_ selectivity, making it well‐suited for the separation of CO_2_/C_2_H_2_ mixtures [270]. Induced by light, a photocontrollable gate effect was observed in **PMOF‐1**. Zwitterions have intrinsic electric field gradients due to the presence of electropositive and electronegative groups, and thus are good candidates to construct CO_2_‐selective CNs [711]. Structural data revealed that CO_2_ adsorption is enabled by the PIET process that increases CO_2_‐framework interactions while maintaining the intrinsic electric field gradient (Figure 7a). Weaker C_2_H_2_ adsorption results from hydrogen bonds involving C_2_H_2_.

Considering the lower polarizability of CO_2_ than HCs, in 2021, B. Chen et al. reported the ultramicroporous PCN **Cu‐F‐pymo** ([Cu(5‐F‐pymo)_2_]·1.25 H_2_O, F‐pymo = 5‐fluoropyrimidin‐2‐olate) with pore size 3.4 × 3.4 Å^2^ for inverse CO_2_/C_2_H_2_ separation [267]. IAST selectivity around10^5^ suggests molecular sieving. Structural analysis of partially dehydrated **Cu‐F‐pymo** revealed residual water molecules occupying the C_2_H_2_‐preferential site, further diminishing C_2_H_2_ sorption. Supported by sorption experiments and modelling studies, the sieving effect precludes coadsorption of C_2_H_2_ and ensures high purity and productivity in cycling column breakthrough tests.

In 2021, Telfer et al. reported **MUF‐16** (MUF = Massey University Framework) MOFs with selectivity for CO_2_ over C_2_H_2_ [129]. These MOFs have optimal pore size and electrostatic moieties for CO_2_ binding as well as other favorable noncovalent interactions (Figure 7b). Specifically, the amino and phenyl groups on the pore wall of **MUF‐16** interact with the electropositive oxygen atoms of CO_2_ molecules via H‐bonding. The equimolar CO_2_/C_2_H_2_ IAST selectivity of **MUF‐16** was found to be 510 with a CO_2_ uptake of 2.13 mmolg^−1^.

In 2023, Zhao et al. also used a molecular recognition strategy for CO_2_‐selective sorption with the ultramicroporous adsorbent Al(HCOO)_3_ (**ALF**), achieving strong CO_2_ capture and C_2_H_2_ rejection [266]. The CO_2_/C_2_H_2_ selectivity is facilitated by optimal pore size (4.1 × 5.3 Å^2^) and H‐bonding, with all HCs rejected. In situ FTIR spectroscopy of CO_2_‐loaded **ALF** revealed that the CO_2_ molecules within **ALF** interact with the electropositive CH moieties of formate groups (Figure 7c). **ALF** exhibited high selectivity of 6.5 × 10^5^ with CO_2_ uptake of 3.85 mmol/g at 298 K.

In 2023, Wade et al. reported on **MFU‐4** (Zn_5_Cl_4_(bbta)_3_, bbta = benzo‐1,2,4,5‐bistriazolate), which exhibited a size‐sieving effect that effectively excluded C_2_H_2_ through narrow pore windows (2.2 × 2.2 Å^2^) formed by Zn–Cl groups (Figure 7d) [268]. Column breakthrough experiments demonstrated a high kinetic selectivity of 3360, determined from the ratio of the diffusion time constants for CO_2_ and C_2_H_2_. Additionally, the **MFU‐4**‐packed column was easily regenerated by purging with helium at room temperature.

**FIGURE 8:**
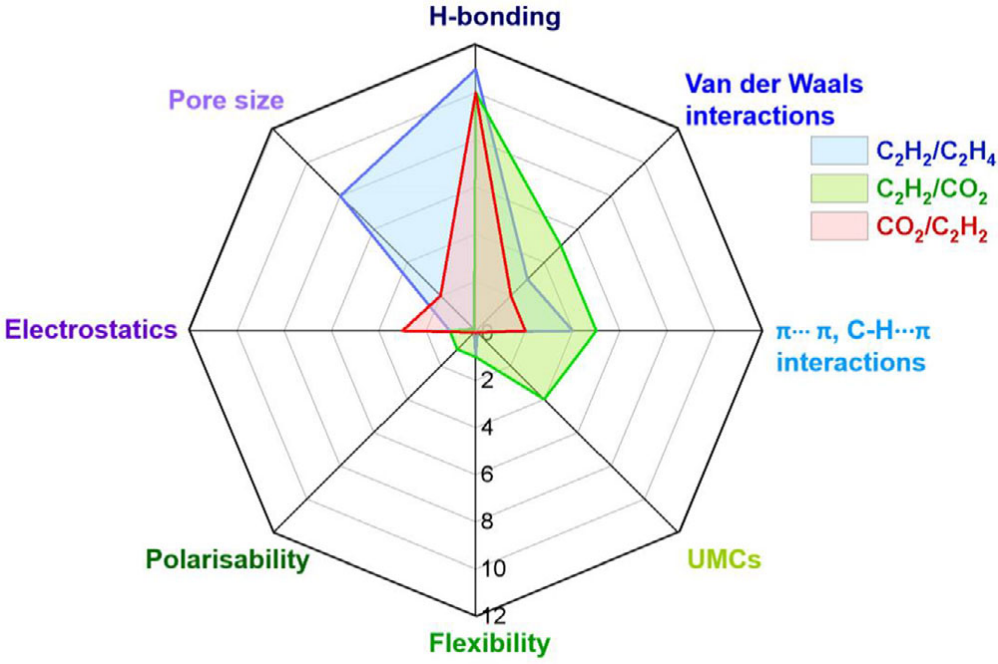
Radar plot showing the contributions of eight separation mechanisms (and their combinations) among the 15 top‐performing reticular sorbents that exhibit binary C_2_H_2_/C_2_H_4_, C_2_H_2_/CO_2_, and CO_2_/C_2_H_2_ adsorption selectivities. Each concentric octagon represents two sorbents, while the central octagon represents one.

As presented in Figure 8, eight mechanisms have been asserted in the 15 top‐performing reticular sorbents that demonstrate C_2_H_2_/C_2_H_4_, C_2_H_2_/CO_2_, and CO_2_/C_2_H_2_ adsorption selectivity: pore size (e.g., ultramicropores inducing partial and/or complete size sieving of C_2_H_2_ over C_2_H_4_ or CO_2_ over C_2_H_2_); π–π interactions (including π···π and C–H···π interactions); framework flexibility (gate opening or switching selective to C_2_H_2_); H‐bonding; electrostatics; dipole‐dipole interactions; van der Waals interactions; UMCs. A radar plot for these three binary mixtures (Figure 8) indicates that hydrogen bonding between sorbates and sorbents tends to play a crucial role in both C_2_H_2_‐ and CO_2_‐selective separations. In essence, the acidic alkyne hydrogen atoms in C_2_H_2_ and the electropositive oxygen atoms in CO_2_ make it feasible for adsorbents embedded with hydrogen donors and acceptors to be highly selective. Additionally, optimal pore size is important for C_2_H_2_/C_2_H_4_ and CO_2_/C_2_H_2_ mixtures, with molecular sieving achieving ultra‐high selectivity in a number of examples as discussed above. When combined with moderate regeneration conditions, the advances summarized in this section indicate that adsorbents that exhibit a molecular sieving effect offer strong potential for further development in commodity purification applications.

#### 3.2.3 C_2_H_4_‐Selective Separation

##### 3.2.3.1 C_2_H_4_/C_2_H_6_ Separation

C_2_H_4_ is one of the most widely produced chemicals in the petrochemical industry, serving as the precursor to a wide range of solvents, polymers, and chemical commodities, including plastics and coatings [712, 713]. Industrially, C_2_H_4_ is manufactured mainly by steam cracking of naphtha or dehydrogenation of C_2_H_6_, invariably resulting in the formation of C_2_H_6_ as a byproduct [714, 715]. The production of C_2_H_4_ requires cryogenic separation from C_2_H_6_, an energy‐consuming step. This drives the development of adsorptive separation of C_2_H_4_ from C_2_H_6_ using solid sorbents. In recent years, several reticular sorbents have been reported for C_2_H_4_/C_2_H_6_ separation to produce polymer‐grade (>99.9% purity) C_2_H_4_ (Table 8). Table 8 presents the leading sorbents reported to date for C_2_H_4_/C_2_H_6_ separation, listed in decreasing order of adsorption selectivity. The sorbents with the highest selectivity in Table 8 were found to have a wide range of pore sizes ranging from 3.8 to 11 Å. Among the top ten reticular sorbents, some of those with smaller pores (3–5 Å) exhibited high selectivity due to molecular sieving. Other sorbents also demonstrated high selectivity, with high *Q*_st_ values for C_2_H_4_. Five studies reporting reticular sorbents with high selectivity are discussed below.

**FIGURE 9:**
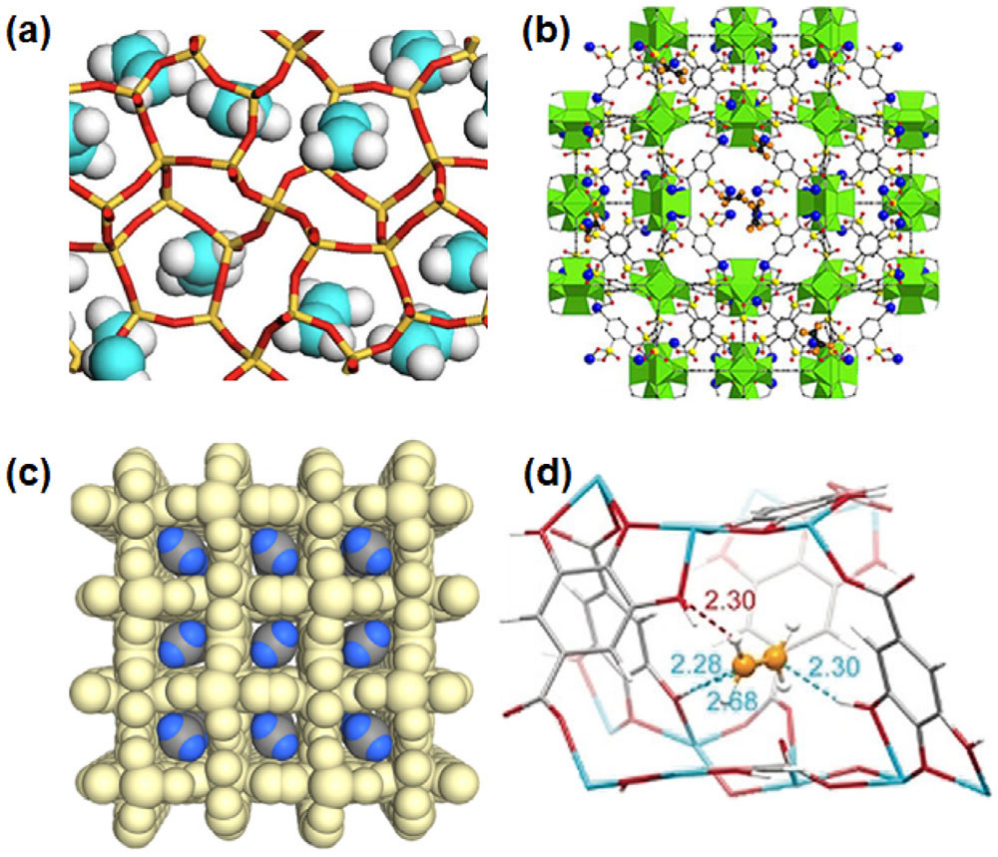
Examples of top‐performing reticular sorbents for C_2_H_4_/C_2_H_6_ separation with high selectivity: (a) Zeolite **ITQ‐55** [289], (b) **NUS‐6(Hf)‐Ag** [288], (c) **UTSA‐280** [287], and (d) **M‐gallate** (M = Ni, Mg, Co) [291] with feed ratio, temperature, and total pressure set at 1:1, 298 K, and 1 bar, respectively. (Reprinted with permission from ref. [289]: Copyright 2018, American Association for the Advancement of Science; ref. [288]: Copyright 2017, American Chemical Society; ref. [287]: Copyright 2018, Springer Nature; ref. [291]: Copyright 2020, American Chemical Society.).

In 2017, Bereciartua et al. reported kinetic separation of C_2_H_4_/C_2_H_6_ in a flexible zeolite (**ITQ‐55**) [289]. **ITQ‐55** possesses two parallel zig‐zag pore channels with a window aperture of 2.33 × 5.71 Å^2^, constraining C_2_H_4_ in this channel induced expansion to 3.08 × 5.71 Å^2^ as indicated by ab initio molecular dynamics simulations (Figure 9a). Kinetic studies revealed that **ITQ‐55** exhibits faster adsorption for C_2_H_4_ than C_2_H_6_ and selectivity of ∼100 with C_2_H_4_ uptake of 1.28 mmol g^−1^ at 298 K. This kinetic separation of a C_2_H_4_/C_2_H_6_ mixture was also demonstrated by dynamic column breakthrough (DCB) experiments.

In the same year, a silver‐modified Hf‐MOF, **NUS‐6(Hf)‐Ag**, demonstrated olefin selectivity and effectiveness for C_2_H_4_/C_2_H_6_ separation (Figure 9b) [288]. High IAST selectivity of 106 for equimolar C_2_H_4_/C_2_H_6_ was obtained with C_2_H_4_ uptake of 2.02 mmol/g at 298 K. Despite the relatively large pores dimensions (10×17 Å), C_2_H_4_ and C_2_H_6_ molecules were distinguished by interactions between C_2_H_4_ and Ag^+^ cations driven by electron donation from C_2_H_4_ to Ag^+^ and concomitant back bonding from the d orbital of Ag^+^ to the vacant π* orbital of C_2_H_4_. **NUS‐6(Hf)‐Ag** demonstrated good recyclability without any appreciable loss of C_2_H_4_ uptake capacity and C_2_H_4_/C_2_H_6_ adsorption selectivity over three consecutive DCB cycles. These results highlight the utility of post‐synthetic incorporation of metal salts into MOFs for olefin‐selective separation [288].

In 2018, an example of molecular sieving was detailed by Lin et al. in a rigid ultra‐microporous MOF (**UTSA‐280**, Figure 9c) [287]. **UTSA‐280** features 1D pore channels with two aperture sizes of 3.2 × 4.5 Å^2^ and 3.8 × 3.8 Å^2^, whose cross‐sectional area (14.4 Å^2^) is midway between those of C_2_H_4_ (13.7 Å^2^) and C_2_H_6_ (15.5 Å^2^) molecules. As a result, **UTSA‐280** exhibited a high C_2_H_4_ uptake of 2.5 mmol/g at 298 K and 1 bar, with almost complete exclusion of C_2_H_6_ (0.09 mmol/g). The calculated IAST selectivity of **UTSA‐280** for an equimolar C_2_H_4_/C_2_H_6_ mixture is over 10,000 at 298 K, setting a new benchmark. SCXRD analysis and DFT calculations revealed that C_2_H_4_ interacts with **UTSA‐280** through multiple weak C─H···O hydrogen bonds and van der Waals interactions. In contrast, C_2_H_6_ is prevented from passing into the channels due to its larger molecular size. Breakthrough experiments confirmed that **UTSA‐280** separated C_2_H_4_ from a C_2_H_4_/C_2_H_6_ mixture with a high productivity of 1.86 mol/kg and effluent purity >99.2%.

In the same year, another family of reticular sorbents for C_2_H_4_/C_2_H_6_ separation, **M‐gallate** (M = Ni, Mg, Co), was reported by Bao et al. (Figure 9d) [291]. The 3D interconnected zig‐zag channels and the aperture sizes of 3.47 × 4.85, 3.56 × 4.84, and 3.69 × 4.95 Å^2^ for Ni, Mg, and **Co‐gallate**, respectively, enabled molecular sieving of C_2_H_4_ (3.28 × 4.18 Å^2^) over C_2_H_6_ (3.81 × 4.08 Å^2^). **Co‐gallate** exhibited a much higher uptake of C_2_H_4_ than C_2_H_6_ (3.37 mmol/g vs. 0.31 mmol/g at 298 K and 1 bar) with a high IAST selectivity of 52 at 298 K. High‐resolution neutron powder diffraction experiments revealed strong binding affinity between C_2_D_4_ molecules and the ligands with multiple C─D···O hydrogen bonding interactions.

In 2019, UMCs were created through immobilization of metal ions within MOFs for highly efficient C_2_H_4_/C_2_H_6_ separation. Qian et al. reported two tailor‐made copper(I)‐chelated adsorbents (**CuI@UiO‐66‐COOH** and **CuI@UiO‐66‐(COOH)_2_**). The introduction of Cu(I) ions cannot only present multiple strong binding sites but also fine‐tune the pore size to better fit C_2_H_4_. **CuI@UiO‐66‐(COOH)_2_** was found to exhibit optimal pore size (4.1 Å) and open Cu(I) sites to selectively bind C_2_H_4_ through π‐complexation. The IAST selectivity of **CuI@UiO‐66‐(COOH)_2_** for an equimolar C_2_H_4_/C_2_H_6_ mixture was 80.8 at 298 K and 1.0 bar. The separation performance of **CuI@UiO‐66‐(COOH)_2_** was confirmed by the breakthrough experiments [290].

Overall, the top‐performing reticular sorbents for C_2_H_4_/C_2_H_6_ separation are enabled by (A) difference in molecular sizes (Table 2) and/or (B) strong noncovalent interactions (e.g., UMCs and a combination of multiple hydrogen bonds). Both factors can play roles in C_2_H_4_‐selective separation.

#### 3.2.4 C_2_H_6_‐Selective Separation

##### 3.2.4.1 C_2_H_6_/C_2_H_4_ Separation

As mentioned above, removal of impurities is required for the production of polymer‐grade C_2_H_4_. However, for solid sorbents, C_2_H_4_‐selective separation requires a second step of sorbent regeneration to obtain purified C_2_H_4_. One‐step purification with selective removal of C_2_H_6_ from C_2_H_4_ is therefore a more desirable approach. However, because C_2_H_4_ features a larger quadrupole moment (Table 2; 1.50 × 10^26^ esu cm^2^) and lower polarizability (Table 2; 42.52 × 10^25^ esu cm^3^) compared to C_2_H_6_ (Table 2; 0.65 × 10^26^ esu cm^2^ and 44.7 × 10^25^ esu cm^3^, respectively), C_2_H_4_/C_2_H_6_ selectivity is typically observed. C_2_H_6_‐selective “reverse” sorption is challenging, but recent reports have achieved such selectivity by utilizing multiple C─H···O/N/F hydrogen bonds, C─H···π bonds, van der Waals interactions, or combinations thereof. Each of these interactions could be potentially augmented by structural flexibility that results in induced fit binding. C_2_H_6_‐selective sorbents are a topical subject [85], and Table 9 presents the leading reticular sorbents reported to date for C_2_H_6_/C_2_H_4_ separation, arranged in decreasing order of adsorption selectivity. As revealed by Table 9, compared to C_2_H_4_‐selective sorption, the selectivity values for C_2_H_6_/C_2_H_4_ tend to be lower, with the highest reported selectivity being 15.4. For this type of “reverse” selective sorption, pore size cannot lead to sieving as C_2_H_6_ is larger than C_2_H_4_. Rather, non‐covalent interactions, which can differ between C_2_H_6_ and C_2_H_4_, are key to their separation. As detailed in Table 9, H‐bonding has been consistently asserted as being involved in the separation mechanism. Flexibility can also play a role in C_2_H_6_/C_2_H_4_ separation, as it can enhance the generally low *Q*_st_ values observed for C_2_H_6_. Five studies reporting reticular sorbents with high selectivity are discussed below.

**FIGURE 10:**
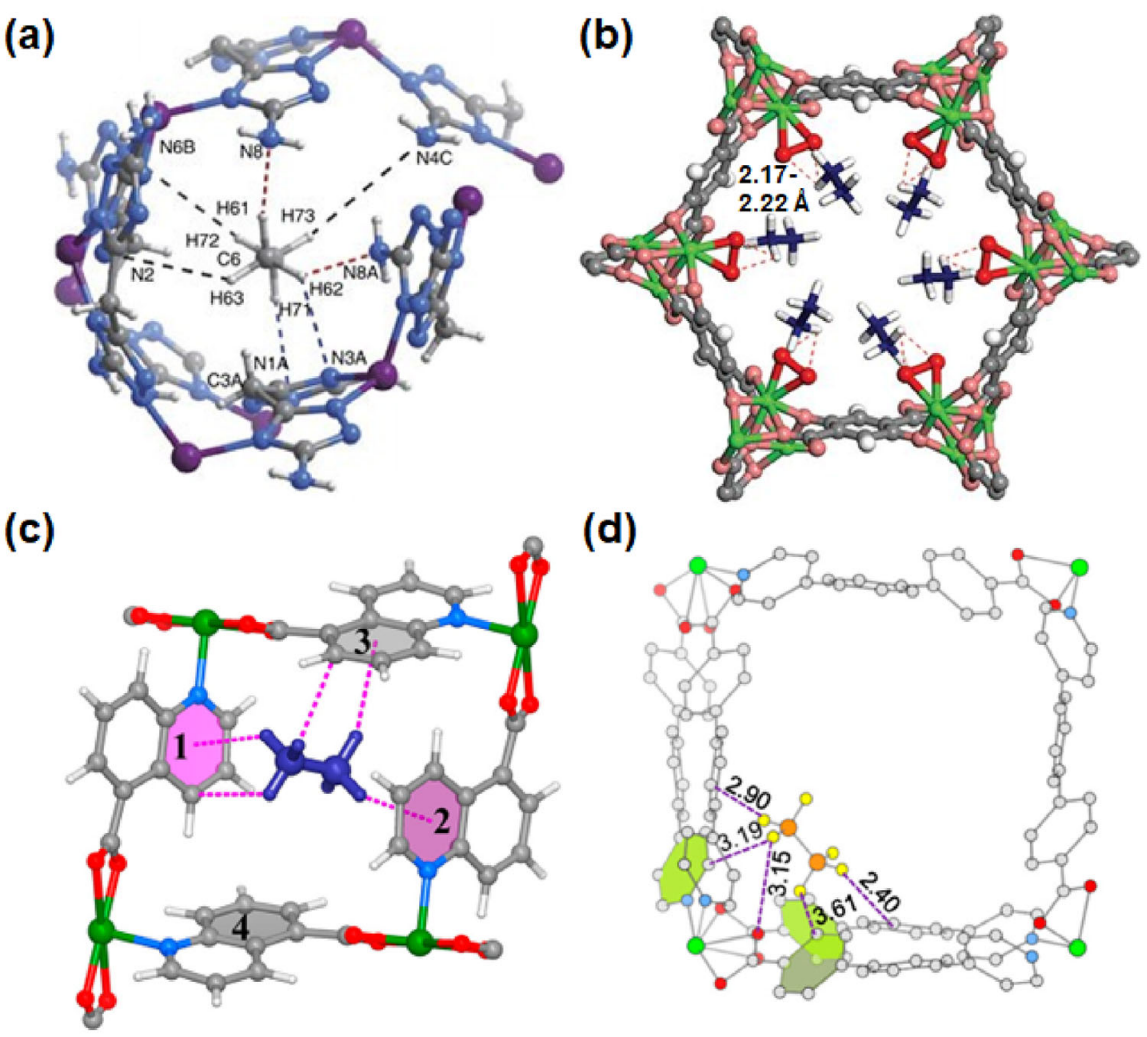
Examples of top‐performing reticular sorbents for C_2_H_6_/C_2_H_4_ separation. (a) **MAF‐49** [314], (b) **Fe_2_(O_2_)(dobdc)** [315], (c) **Cu(Qc)_2_** [317], and (d) **X‐dia‐1‐Ni_0.89_Co_0.11_** [316], with feed ratio, temperature, and total pressure set at 1:1, 298 K, and 1 bar, respectively. (Reprinted with permission from ref. [314]: Copyright 2015, Springer Nature; ref. [315]: Copyright 2017, American Association for the Advancement of Science; ref. [317]: Copyright 2018, American Chemical Society; ref. [316]: Copyright 2024, American Chemical Society.).

X. M. Chen and co‐workers reported a metal‐azolate framework (MAF) in 2015, **MAF‐49** [Zn(batz)]*_n_* (H_2_batz = bis(5‐amino‐1*H*‐1,2,4‐triazol‐3‐yl)methane, Figure 10a), with equimolar C_2_H_6_/C_2_H_4_ IAST selectivity of 9 and C_2_H_6_ uptake of 2.5 mmol g^−1^ at 316 K [314]. The combination of (a) multiple C−H···N hydrogen bonds between C_2_H_6_ molecules and the **MAF‐49** pore surface and (b) dipolar repulsion groups located at specific positions across the narrow pore channels (3.3 × 3.0 Å^2^) enabled preferential adsorption of C_2_H_6_ over C_2_H_4_.

In 2018, B. Chen et al. reported the microporous MOF **Fe_2_(O_2_)(dobdc)** (dobdc^4−^: 2,5‐dioxido‐1,4‐benzenedicarboxylate), which displayed highly selective separation of C_2_H_6_/C_2_H_4_ (Figure 10b) [315]. At 298 K and 1 bar, the C_2_H_6_ uptake and the selectivity for an equimolar C_2_H_6_/C_2_H_4_ mixture were 3.32 mmol/g and 4.4, respectively. Despite the relatively large pore dimensions (14 × 14 Å^2^), the Fe‐peroxo sites on the pore surface of **Fe_2_(O_2_)(dobdc)** enabled recognition of C_2_H_6_ and adsorption of a larger amount of C_2_H_6_ than C_2_H_4_.

In the same year, another report from B. Chen's group on controlling pore apertures and chemistry enabled the material **Cu(Qc)_2_** (Qc^−^ = quinolone‐5‐carboxylate) to feature a weak polar pore surface (Figure 10c). Ultramicroporous **Cu(Qc)_2_**, with a pore size of 3.3 × 3.3 Å^2^ exhibited self‐adaptive sorption behavior for C_2_H_6_ and thus higher binding affinity (C─H···π interactions) towards C_2_H_6_ over C_2_H_4_ [317]. At 298 K and 1 bar, it presented an IAST selectivity of 3.4 for equimolar C_2_H_6_/C_2_H_4_ mixtures and a C_2_H_6_ uptake of 1.85 mmol/g.

As reported in 2020, introducing HC pendant groups in a family of **NIIC‐20** MOFs reduced pore dimensions and increased hydrophobicity, whereas glycerol introduced hydrophilicity with OH‐groups that facilitated hydrogen bond formation [313]. Specifically, **NIIC‐20‐Bu** (1,2‐butane‐diol) exhibited a record‐high equimolar C_2_H_6_/C_2_H_4_ IAST selectivity of 15.4 with C_2_H_6_ uptake of 2.5 mmol/g at 298 K. A tight pore aperture (3.5 Å) and three adsorption sites were identified within the nanocages of **NIIC‐20‐Bu**. Multiple C─H···π and C─H···O interactions between C_2_H_6_ and two phenyl rings were observed; in parallel, carboxyl moieties enabled high inverse adsorption affinity C_2_H_6_ over C_2_H_4_.

**FIGURE 11:**
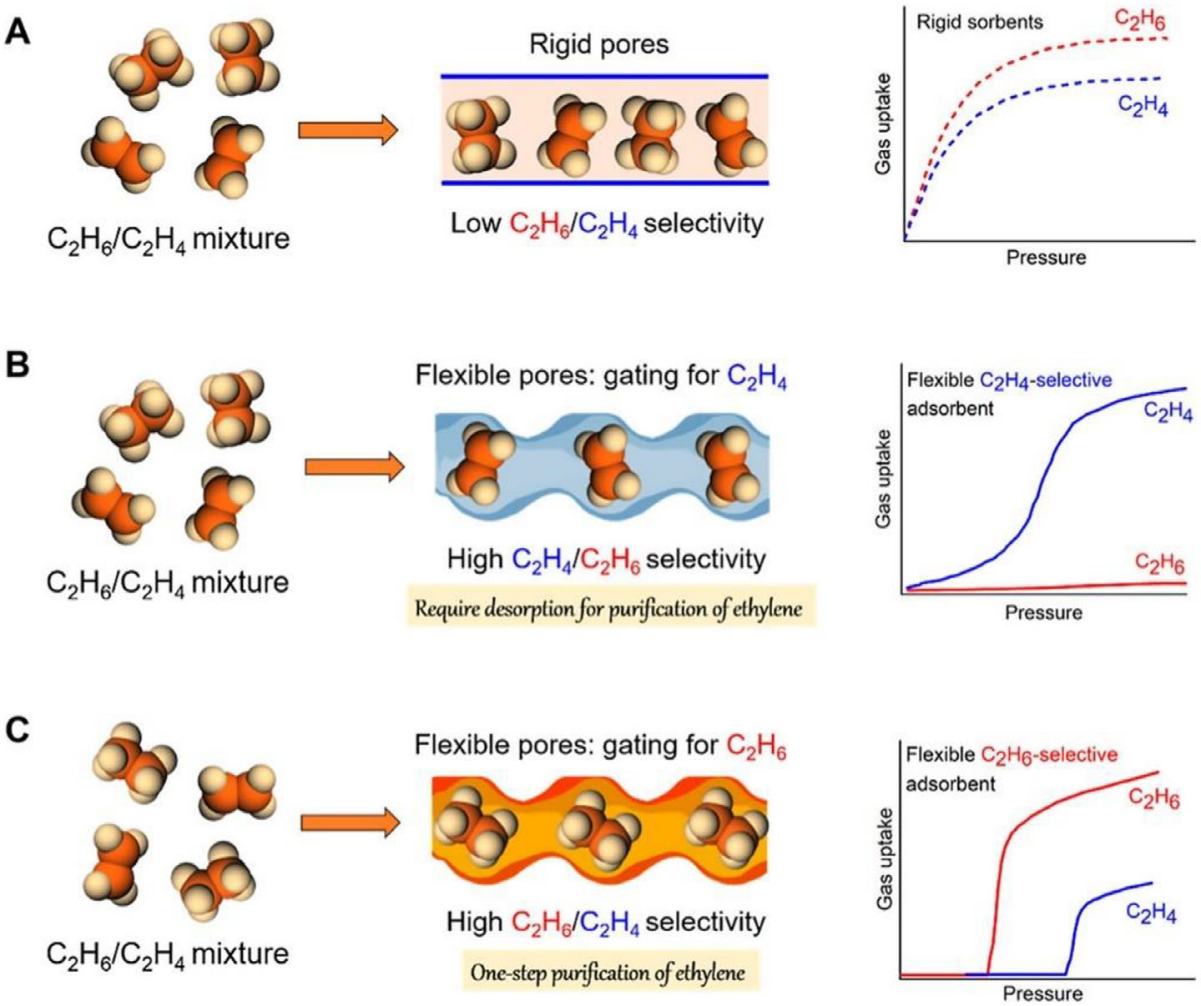
Illustration of rigid and flexible adsorbents for C_2_H_6_/C_2_H_4_‐selective adsorption. (A) Rigid MOFs tend to show little adsorption difference between C_2_H_4_ and C_2_H_6_. (B) Flexible C_2_H_4_‐selective adsorbents require an additional desorption step to release the adsorbed C_2_H_4_ molecules. (C) Flexible C_2_H_6_‐selective porous materials can thereby be optimal for C_2_H_6_/C_2_H_4_ separation. (Reprinted with permission from ref. [316]: Copyright 2024, American Chemical Society.).

Recently, S. M. Wang et al. reported a family of flexible MOFs, **X‐dia‐1‐Ni_0.89_Co_0.11_** and **X‐dia‐1‐Ni**, that exhibit high equimolar C_2_H_6_/C_2_H_4_ IAST selectivities of 5.47 and 3.51 with respective C_2_H_6_ uptakes of 4.96 and 5.54 mmol g^−1^ at 273 K (Figures 10d and 11) [316]. Insights from structural analysis revealed that C_2_H_6_ induced phase transformation abruptly with pressure increase, switching the sorbent from its narrow pore (NP) phase to a large pore (LP) phase. Multiple C‐H···O interactions between C_2_H_6_ and **X‐dia‐1‐Ni**‐LP were found to contribute toward the higher binding energy, −23.4 and −20.5 kJ/mol for C_2_H_6_ and C_2_H_4_, respectively, of **X‐dia‐1‐Ni‐LP**.

**FIGURE 12:**
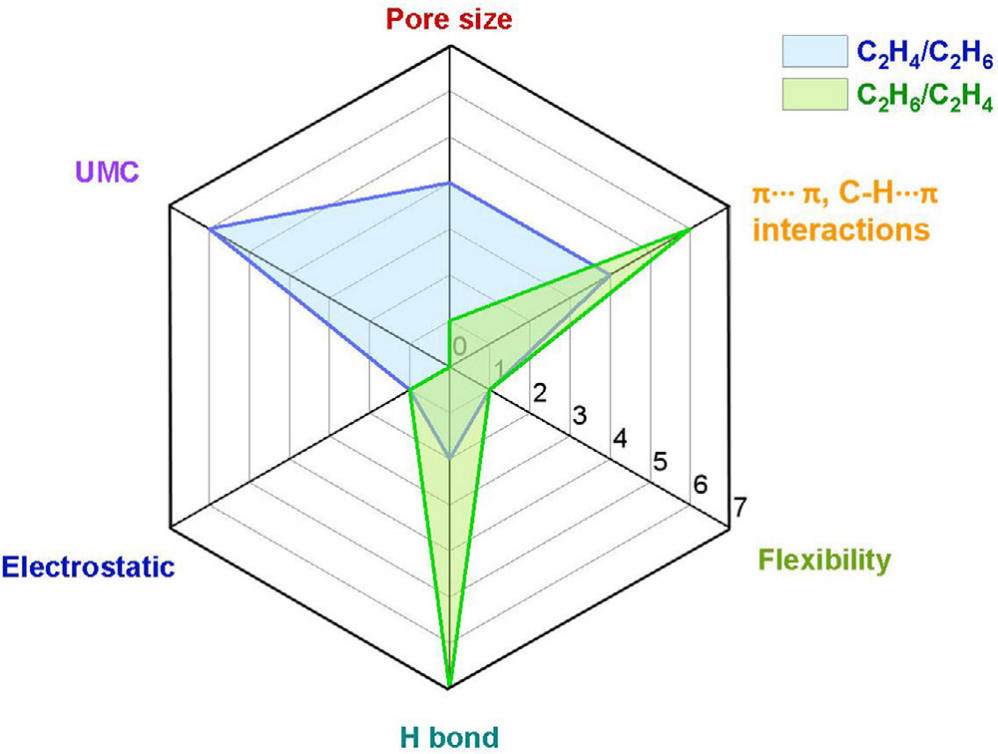
Radar plot presenting the contributions of six separation mechanisms (and their combinations) among the 15 top‐performing reticular sorbents that exhibit binary C_2_H_4_/C_2_H_6_ and C_2_H_6_/C_2_H_4_ adsorption selectivities. Every concentric hexagon denotes an individual sorbent, with the central hexagon symbolizing one sorbent.

As summarized in Figure 12, six mechanisms have been asserted in the 15 top‐performing reticular sorbents that demonstrate C_2_H_4_/C_2_H_6_ and C_2_H_6_/C_2_H_4_ adsorption selectivities: pore size (e.g., ultramicropores inducing partial and/or complete size sieving of C_2_H_4_ over C_2_H_6_ or the opposite); π‐π interactions (including π···π and C─H···π interactions); framework flexibility (gate opening or switching selective to either C_2_H_4_ or C_2_H_6_); hydrogen bonding (H‐bonding); electrostatics; UMCs. Occurrences of these six mechanisms revealed the dominating factors behind both types of separation (C_2_H_4_/C_2_H_6_ is the “normal” selectivity, whereas C_2_H_6_/C_2_H_4_ represents “inverse” selectivity).

With regard to C_2_H_4_/C_2_H_6_ separation, UMCs are the most typical feature. UMCs can result from unsaturated metal centers on the pore walls or post‐synthetic modification by the introduction of salts. Specific binding interactions can be generated between vacant orbitals of the UMCs and π electrons of the olefinic C_2_H_4_ bond. Interactions between π electrons of C_2_H_4_ and aromatic rings from the linker ligands can also enable C_2_H_4_‐selective binding. Coadsorption is generally unavoidable and hinders the harvesting of high‐purity C_2_H_4_ streams. Thanks to the different molecular sizes of C_2_H_4_ and C_2_H_6_ (Table 2), pore size control can result in sieving and ultrahigh C_2_H_4_ selectivity. C_2_H_6_/C_2_H_4_ separations predicated upon superior C_2_H_6_ binding are primarily enabled by noncovalent interactions between C_2_H_6_ and pore walls. For instance, H‐bonding interactions between the alkane hydrogens of C_2_H_6_ and H‐bond acceptor sites, along with C─H···π interactions between C_2_H_6_ and aromatic rings, can play a significant role in C_2_H_6_/C_2_H_4_ separation.

To produce high‐purity C_2_H_4_, a one‐step C_2_H_4_ production process that removes C_2_H_6_ from a C_2_H_6_/C_2_H_4_ mixture is ideal, especially when considering the low energy footprint of sorbent recycling. Unfortunately, unlike C_2_H_4_/C_2_H_6_ separation, molecular sieving is inapplicable for selective capture of C_2_H_6_ over C_2_H_4_. Also, limited by the relatively low difference in binding between C_2_H_6_ and C_2_H_4_, the selectivity for C_2_H_6_/C_2_H_4_ (highest value = 15.4) is much lower than that for C_2_H_4_/C_2_H_6_ (highest value = 106). Physisorbents that feature C_2_H_6_‐induced structural flexibility and selective binding of C_2_H_6_ over C_2_H_4_ possess high upside potential to further boost C_2_H_6_/C_2_H_4_ selectivity.

Overall, for the binary separation of C_2_H_6_/C_2_H_4_, the IAST selectivity trends among the top‐performing reticular sorbents indicate that three approaches tend to enhance performance: (A) strengthening binding affinity towards C_2_H_6_; (B) enhancing host (sorbent)–guest (C_2_H_6_) binding strength; (C) decreasing sorbent‐C_2_H_4_ binding strength.

### 3.3 C3 Separations

C3 HCs, particularly propylene (C_3_H_6_) and propane (C_3_H_8_), are high‐volume commodity chemicals used in the production of fuels, polymers, and other chemical products. The demand for C3 HCs is on the rise. However, achieving the required purity (>99.95%) for polymer production and other chemical processes currently relies on costly and energy‐intensive methods such as cryogenic distillation, which remains the industry standard. Adsorptive separations using porous materials could provide a more energy‐efficient alternative. Notably, after C_2_H_4_, C_3_H_6_ is the second most valuable olefin feedstock in the petrochemical industry, and serves as a feedstock for synthesising various products, including polypropylene. Industrially, C_3_H_6_ is primarily produced through the steam cracking of naphtha, a process that inevitably introduces impurities such as C_3_H_8_, methylacetylene (MA, C_3_H_4_), and trace amounts of propadiene (PD, C_3_H_4_).

#### 3.3.1 C_3_H_4_‐Selective Separation

##### 3.3.1.1 C_3_H_4_/C_3_H_6_ Separation

C_3_H_6_ feeds are inevitably mixed with trace amounts of C_3_H_4_, which is undesirable because of catalyst poisoning. Polymer‐grade C_3_H_6_ requires that the concentration of C_3_H_4_ must be <5 ppm. Compared to the traditional method, cryogenic distillation, adsorptive separation of C_3_H_4_ from C_3_H_6_ using reticular porous materials promise to be more cost‐effective and energy‐efficient (Table 10). Table 10 presents the leading reticular sorbents reported to date for C_3_H_4_/C_3_H_6_ separation, arranged in decreasing order of adsorption selectivity. The sorbents with the highest selectivity were found to have pore sizes ranging from 3.4 to 8 Å. However, pore size was not observed to play a significant role in C_3_H_4_‐selective sorption. Compared to C1‐C2 separations, the *Q*_st_ values between C_3_H_4_ and the frameworks are generally higher, leading to increased energy requirements for sorbent regeneration. H‐bonding between C_3_H_4_ and the frameworks has been frequently observed as a key separation mechanism. Four studies reporting reticular sorbents with high C_3_H_4_ selectivity are discussed below.

In 2017, B. Chen and colleagues accomplished C_3_H_4_/C_3_H_6_ separation for the first time using a flexible yet robust MOF, **ELM‐12** [Cu(bpy)_2_(OTf)_2_] (OTf^−^ = trifluoromethanesulfonate) [438]. The strong binding affinity and pore confinement for C_3_H_4_ result in high uptake capacity and selectivity, as demonstrated by neutron powder diffraction studies and density functional theory calculations, to achieve C_3_H_6_ purity of >99.9998%.

**FIGURE 13:**
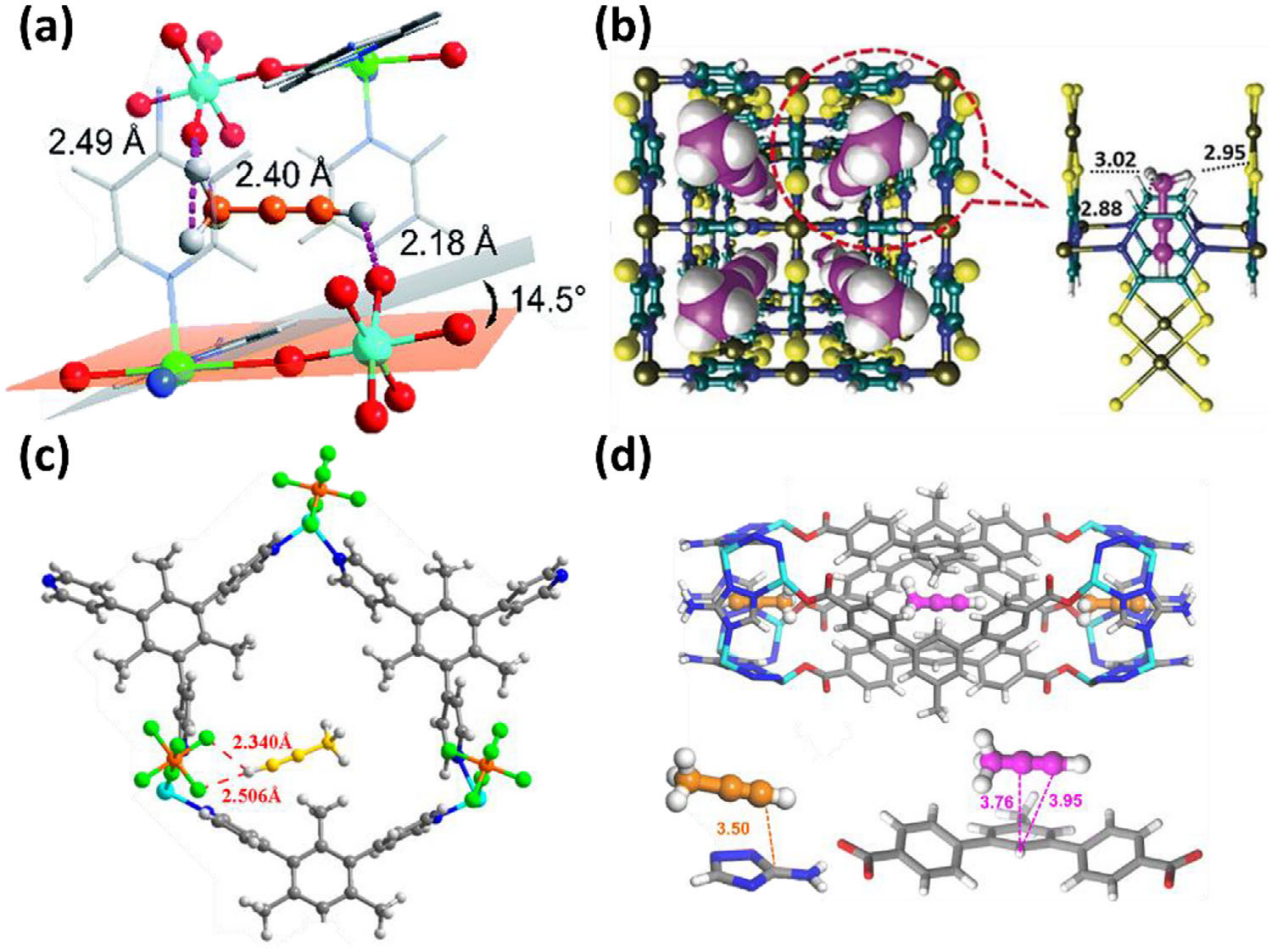
Examples of top‐performing reticular sorbents for C_3_H_4_/C_3_H_6_ separation with high selectivity: (a) **TIFSIX‐14‐Cu‐i** [432] with feed ratio, temperature, and total pressure set at 1:1, 298 K, and 1 bar, respectively. (b) **NKMOF‐1‐Cu** [428] with feed ratio, temperature, and total pressure set at 0.5:99, 298 K, and 1 bar, respectively. (c) **FJI‐W1** [431] with feed ratio, temperature, and total pressure set at 1:99, 298 K, and 1 bar, respectively. (d) **BUT‐306** [430] with feed ratio, temperature, and total pressure set at 1:1, 298 K, and 1 bar, respectively. (Reprinted with permission from ref. [432]: Copyright 2018, The Royal Society of Chemistry; ref. [428]: Copyright 2019, Wiley‐VCH GmbH; ref. [431]: Copyright 2022, American Chemical Society; ref. [430]: Copyright 2020, The Royal Society of Chemistry.).

Flexible MOFs that exhibit stepwise adsorption isotherm profiles can theoretically offer high (even infinite) separation selectivity. However, their practical utility in separating gas mixtures often falls significantly short of the theoretical values derived from pure‐component sorption isotherms. This discrepancy is due to the unpredictable effects of exposure to gas mixtures. In 2018, H. Xing's group reported a flexible HUM, **TIFSIX‐14‐Cu‐i** (also termed as **ZU‐13**, TIFSIX = TiF_6_^2−^, 14 = 4,4′‐azobipyridine, i = interpenetrated) with an anion‐pillared structure and ultramicroporous pore diameter of 3.4 Å [432]. **TIFSIX‐14‐Cu‐i** featured an ultra‐low C_3_H_4_ threshold pressure of 500 ppm, an important requirement to produce polymer‐grade C_3_H_6_ as the effluent. **TIFSIX‐14‐Cu‐i** demonstrated exceptionally high C_3_H_4_/C_3_H_6_ IAST selectivity of 355 for separating 1/99 C_3_H_4_/C_3_H_6_ mixtures, achieving an adsorption capacity of 1280 mL/g and C_3_H_6_ purity exceeding 99.9999%, as evidenced by breakthrough results. Density functional theory calculations revealed that the underlying mechanism for C_3_H_4_/C_3_H_6_ separation in **TIFSIX‐14‐Cu‐i** involves hydrogen bonding and Van der Waals interactions with the C3 sorbates (Figure 13a).

In 2019, Y. Peng et al. reported two isostructural HUMs, **NKMOF‐1‐Ni** and **NKMOF‐1‐Cu** (Cu [M(pdt)_2_] (pdt = pyrazine‐2,3‐dithiol, M = Cu, Ni)) with pore dimensions of *ca*. 5.7 × 5.7 Å^2^ [428]. High IAST selectivities of 1217.8 and 859.5 for the separation of C_3_H_4_/C_3_H_6_ at a v/v ratio of 0.5/99 were observed in **NKMOF‐1‐Ni** and **NKMOF‐1‐Cu**, respectively. Gas‐loaded single‐crystal structures provided insight into the separation mechanism, which involves hydrogen bonding and π···π interactions (Figure 13b). In 2021, the same research group reported an isostructural material, **NKMOF‐11** (Ni [Cu(pdt)_2_], pdt = pyrazine‐2,3‐dithiol). Half of the nickel (Ni) atoms in **NKMOF‐1‐Ni** were replaced by copper (Cu) atoms in **NKMOF‐11**, resulting in the same pore dimensions and high IAST selectivity of 1074 for C_3_H_4_/C_3_H_6_ at a v/v ratio of 1/99. Due to the high affinity for C_3_H_4_, the selectivity for C_3_H_4_/C_3_H_6_ at a v/v ratio of 1/99 can reach as high as 1388. Structural analysis indicated that. once again, hydrogen bonding and π···π interactions are crucial to the C_3_H_4_‐selective binding.

In 2022, M. Hong and colleagues reported a hybrid microporous material, **FJI‐W1** ([Cu_3_(TMTPB)_4_(SiF_6_)_3_]*_n_*, TMTPB = 1,3,5‐trimethyl‐2,4,6‐tris(4‐pyridyl)benzene) with pore dimensions of 8 × 8 Å^2^ [431]. The IAST selectivity for a binary C_3_H_4_/C_3_H_6_ mixture at a v/v ratio of 1/99 in **FJI‐W1** was calculated to be 593, with dynamic breakthrough experiments lasting up to 600 minutes per gram. Computational studies revealed that the separation of C_3_H_4_/C_3_H_6_ was facilitated by hydrogen bonding with C_3_H_4_ forming strong C─H···F hydrogen bonds with SiF_6_^2−^ in the irregular hexagonal channels (Figure 13c).

In 2022, J. R. Li and co‐workers reported two isoreticular ultramicroporous Zn(II)‐MOFs, **BUT‐305** (Zn_2_(ATZ)_2_(TPDC), H_2_TPDC = [1,1′:3′,1′′‐terphenyl]‐4,4′′‐dicarboxylic acid, HATZ = 3‐amino‐1,2,4‐triazole) and **BUT‐306** (Zn_2_(ATZ)_2_(MeTPDC), H_2_MeTPDC = 5′‐methyl‐ [1,1′:3′,1′′‐terphenyl]‐4,4′′‐dicarboxylic acid) [430]. A pore diameter of 3 Å was observed for **BUT‐306**. High hydrolytic stability and hydrophobicity enabled breakthrough experiments on a binary C_3_H_4_/C_3_H_6_ gas mixture (v/v = 1/1) to be conducted with pre‐saturated water vapor, resulting in an IAST selectivity of 636. The single‐crystal structure of C_3_H_4_‐loaded BUT‐306 revealed that adsorbed C_3_H_4_ molecules lie in the center of the pores and interact with pore walls through multiple weak C*^δ^* ^−^···C*^δ^* ^+^ dipole–dipole interactions and C─H···π interactions (Figure 13d).

#### 3.3.2 C_3_H_6_‐Selective Separation

##### 3.3.2.1 C_3_H_6_/C_3_H_8_ Separation

C_3_H_6_ is predominantly produced through steam cracking of naphtha or dry gas refining, and it is invariably mixed with minor impurities such as C_3_H_8_. Currently, the petrochemical industry relies almost entirely on cryogenic distillation for C_3_H_6_ purification. However, because C_3_H_6_ and C_3_H_8_ have very similar physicochemical properties (Table 2), cryogenic distillation is both inefficient and energy‐intensive. Adsorptive separation using reticular porous materials has been explored to achieve C_3_H_6_ purification from C_3_H_6_/C_3_H_8_ mixtures under ambient conditions. Table 11 presents the leading reticular sorbents reported to date for C_3_H_6_/C_3_H_8_ separation, listed in decreasing order of adsorption selectivity. The sorbents with high selectivity were found to exhibit pore sizes ranging from 3 to 7 Å. Pore size was observed to influence C_3_H_6_‐selective sorption. Interestingly, the uptake at 298 K and 1 bar for C_3_H_6_ did not correlate with pore size/surface area. Six reports of reticular sorbents with high selectivity are discussed below.

In 2009, C_3_H_6_/C_3_H_8_ separation was reported by Lamia et al. using a simulated moving bed (SMB) process conducted on the prototypal high surface area MOF **HKUST‐1** [500]. The observed C_3_H_6_/C_3_H_8_ separation was attributed to UMCs and π‐complexation between the vacant *s*‐orbital of Cu(II) cations and the electron‐rich π orbitals in C_3_H_6_. In the same year, J. Li et al. reported the first example of the kinetic separation of C_3_H_6_/C_3_H_8_, based on their different diffusion rates in Zn(2‐cim)_2_ (2‐cim = 2‐chloroimidazole), which has a pore diameter of 3.26 Å [468].

In 2017, J. Li and co‐workers reported two structurally related microporous MOFs, **Zn(ox)_0.5_(trz)** (ox = oxalate, trz = 1,2,4‐triazole) and **Zn(ox)_0.5_(atrz)** (atrz = 3‐amino‐1,2,4‐triazole) with pore sizes of only 2.9 and 3.3 Å, respectively [458]. Single crystal X‐ray data revealed that the pore channels in both MOFs exhibit a zig‐zag shape. As a result, due to the optimal pore size and the zig‐zag‐shaped ultramicroporous 1D channels, a significant difference in the diffusivity of C_3_H_6_ and C_3_H_8_ in **Zn(ox)_0.5_(trz)** at 323 K was achieved, leading to a kinetic separation factor of 1565.

**FIGURE 14:**
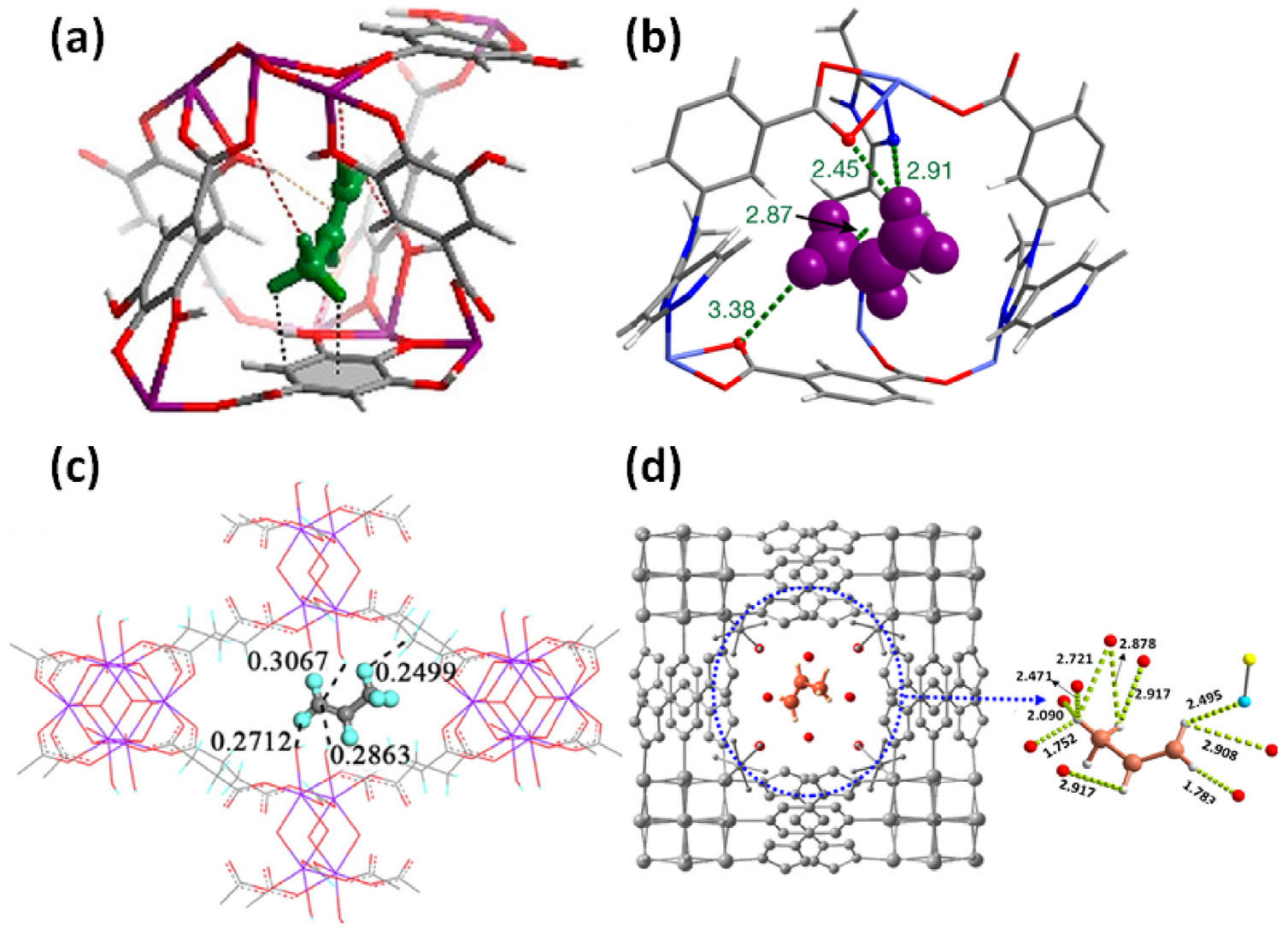
Top‐performing reticular sorbents for C_3_H_6_/C_3_H_8_ separation: (a) **Co‐gallate** [462], (b) **JNU‐3a** [461], (c) **MIP‐203** [460], and (d) **NTU‐85‐WNT** [457] with feed ratio, temperature, and total pressure set at 1:1, 298 K, and 1 bar, respectively. (Reprinted with permission from ref. [462]: Copyright 2020, American Chemical Society; ref. [461]: Copyright 2021, Springer Nature; ref. [460]: Copyright 2022, The Chemical Industry and Engineering Society of China, and Chemical Industry Press Co., Ltd. All rights reserved; ref. [457]: Copyright 2023, American Chemical Society.).

In 2020, B. Chen, W. Zhou, and co‐workers reported an ultramicroporous cobalt gallate MOF (**Co‐gallate**, [Co(gallate)]*_n_*) for highly C_3_H_6_‐selective sieving of C_3_H_6_ from C_3_H_8_ at 298 K [462]. The optimal pore structure of Co‐gallate enabled confinement of C_3_H_6_ while excluding the slightly larger C_3_H_8_, as demonstrated in the neutron diffraction crystal structure of **Co‐gallate⊃0.38C_3_D_6_** (Figure 14a). The IAST selectivity for equimolar C_3_H_6_/C_3_H_8_ separation was calculated to be 330 at 298 K. This high separation performance was subsequently supported by gas sorption isotherms and column breakthrough experiments, resulting in high purity C_3_H_6_ (97.7%). Structural analysis indicated that hydrogen bonding, O‐H···π interactions, and van der Waals forces resulted in selective binding.

The potential separation ability of flexible MOFs has also been reported, but the rational design of flexibility in MOFs for dynamic molecular sieving remains a challenge. In 2021, D. Li et al. reported a flexible MOF, **JNU‐3a** (JNU = Jinan University, Co(MPTBDC)_2_, MPTBDC = 5‐(3‐methyl‐5‐(pyridin‐4‐yl)‐4H‐1,2,4‐triazol‐4‐yl)‐1,3‐benzenedicarboxylate), exhibiting a molecular sieving effect for C_3_H_6_/C_3_H_8_ separation [461]. **JNU‐3a** features one‐dimensional channels with embedded molecular pockets that open to C_3_H_6_ and C_3_H_8_ at significantly different pressures (Figure 14b). The dynamic nature of these pockets is demonstrated by the single‐crystal‐to‐single‐crystal transformation of **JNU‐3a** when exposed to an atmosphere of C_3_H_6_ or C_3_H_8_. **JNU‐3a** exhibited a high IAST selectivity of 513 at 298 K and produced high‐purity C_3_H_6_ (>99.5%). The underlying separation mechanism, involving orthogonal array‐like dynamic molecular sieving, enabled both a large separation capacity and rapid adsorption‐desorption kinetics. In 2022, two flexible Zr‐based MOFs (**MIP‐202** and **MIP‐203**) were prepared with C4 linkers and studied for C_3_H_6_/C_3_H_8_ separation [460]. The temperature‐induced and guest‐induced flexibility of C_3_H_6_‐selective **MIP‐203** resulted in an IAST selectivity of 551.4 at 303 K. Computational studies provided insight into binding sites, which were found to involve H‐bonding and electrostatic interactions (Figure 14c). O‐H···π H‐bonding formed by the ‐OH group in the MBB metal cluster and sp^2^ carbon atoms from C_3_H_6_ play a key role in the experimentally observed high C_3_H_6_/C_3_H_8_ selectivity of **MIP‐203**.

In 2023, J. Bai and co‐workers reported a super tetrahedral‐cluster Cu_10_O_13_‐based MOF (**NTU‐85‐WNT**) and studied its C_3_H_6_/C_3_H_8_ separation performance. With pore dimensions of 4.57 × 4.57 Å^2^ [457], **NTU‐85‐WNT** recorded IAST selectivity of 1570 at 298 K for C_3_H_6_/C_3_H_8_. The C_3_H_6_ selectivity was credited to a new mechanism involving initial framework expansion and subsequent contraction of the confined water nanotubes (∼4.5 Å) exclusively triggered by C_3_H_6_ adsorption (Figure 14d). Thanks to the robust nature of the framework, the water nanotubes were restored by soaking the MOF in water.

#### 3.3.3 C_3_H_8_‐Selective Separation (Inverse Separation)

##### 3.3.3.1 C_3_H_8_/C_3_H_6_ Separation

Compared to C_3_H_6_‐selective adsorption from a C_3_H_6_/C_3_H_8_ mixture, the preferential removal of C_3_H_8_ impurities could be more practically useful, as there would be no need for a C_3_H_6_ desorption process. The subtle structural differences between C_3_H_6_ and C_3_H_8_ mean only slight differences in size and shape, rendering the development of C_3_H_8_‐selective materials a challenge. The use of C_3_H_8_‐selective CNs as adsorbents for the one‐step purification of C_3_H_6_ from C_3_H_8_/C_3_H_6_ binary mixtures is still in its early stages, with only a limited amount of literature available. In 2013, Bohme et al. reported separation of C_3_H_8_/C_3_H_6_ by **CPO‐27‐Co** (**Co_2_(dhtp)**, dhtp = dihydroxyterephthalate) and **ZIF‐8** (Zn(MIM)_2_, MIM = 2‐methylimidazole) [716]. Preferential sorption of C_3_H_8_ over C_3_H_6_ was observed in **ZIF‐8**, thanks to its nonpolar pore environment, which lacks any specific interaction with C_3_H_6_. Although these initial experiments indicated promising potential for C_3_H_8_‐selective adsorbents, there is room to improve capacities and selectivities. Table 12 presents the leading reticular sorbents reported for C_3_H_8_/C_3_H_6_ separation, arranged in decreasing order of adsorption selectivity. Similar to C_2_H_6_/C_2_H_4_ separation, C_3_H_8_/C_3_H_6_ selectivity is generally not as high as the reverse pair (C_3_H_6_/C_3_H_8_), and pore size does not seem to have a significant influence on C_3_H_8_ selectivity. The pore sizes of the highly C_3_H_8_‐selective sorbents are mostly within the range of 4–12 Å. The relatively large pore sizes mean that the uptake of C_3_H_8_ can be high (up to 8.73 mmolg^−1^). The separation mechanism relies on non‐covalent interactions, with *Q*_st_ values between C_3_H_8_ and the frameworks ranging from 31 to 66 kJ/mol. Seven studies reporting reticular sorbents with high selectivity are discussed below.

**FIGURE 15:**
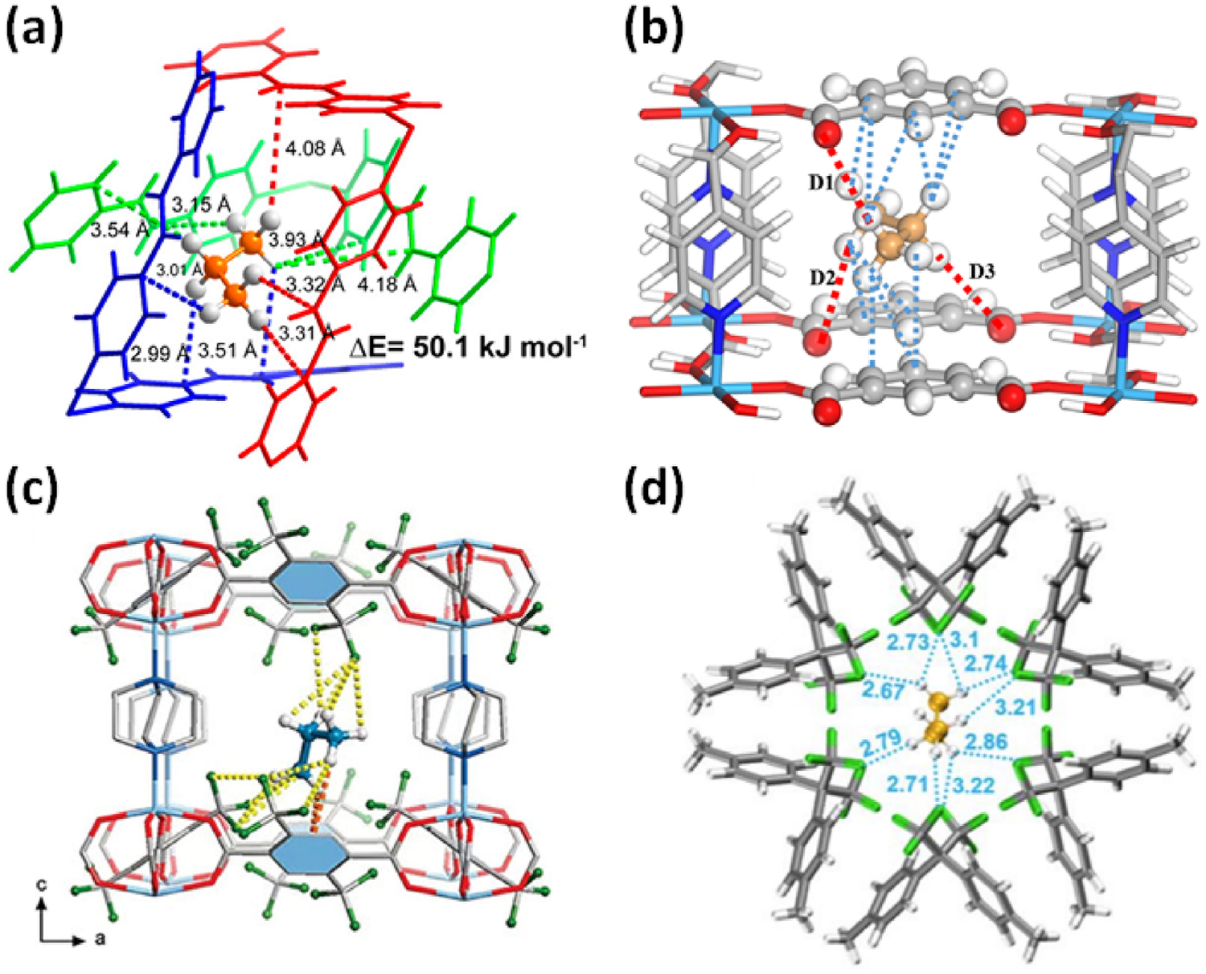
Top‐performing reticular sorbents for C_3_H_6_/C_3_H_8_ separation with high selectivity: (a) **Ni(bpe)_2_(WO_4_)** [505], (b) **PCP‐IPA** [325], (c) **FDMOF‐2** [507], and (d) **JNU‐90** [506] with feed ratio, temperature, and total pressure set at 1:1, 298 K, and 1 bar, respectively. (Reprinted with permission from ref. [505]: Copyright 2021, American Chemical Society; ref. [325]: Copyright 2021, Springer Nature; ref. [507]: Copyright 2023, Wiley‐VCH GmbH; ref. [506]: Copyright 2024, American Chemical Society.).

In 2019, H. Xing and colleagues reported a microporous HUM, **[Ni(bpe)_2_(WO_4_)]*_n_*** (bpe = 1,2‐bis(4‐pyridyl)ethylene), featuring a polycatenated molecular cage. This HUM exhibited IAST selectivity of 1.75 for an equimolar (v/v) C_3_H_8_/C_3_H_6_ mixture. Structural analysis revealed that the cage provided dense electronegative binding sites, facilitating multiple C^δ−^─H^δ+^···C^δ−^ interactions with C_3_H_8_ molecules, thereby conferring higher affinity for C_3_H_8_ over C_3_H_6_ (Figure 15a). Further, the cage exhibited shape selectivity for the oblate C_3_H_8_, while being less favourable to the relatively planar C_3_H_6_.

In 2021, a robust MOF, **Ni(ADC)(TED)_0.5_** (ADC = 9,10‐anthracenedicarboxyl, TED = 1,4‐diazabicyclo [2.2.2] octane or triethylenediamine) featuring sorbate‐induced pore confinement for C_3_H_8_ trappin, was reported to purify C_3_H_6_ [504]. **Ni(ADC)(TED)_0.5_** demonstrated excellent separation performance for C_3_H_8_/C_3_H_6_ (v/v = 1/1), with a record‐high IAST selectivity of 6.4. Structural analysis revealed that the C─H···π interactions between the aromatic rings of the MOF and the C_3_H_8_ molecules enable the observed high C_3_H_8_‐selective sorption.

In 2022, H. Xing and co‐workers reported an ultramicroporous material **PCP‐IPA**, ([Co(IPA)(DPG)]*_n_* (**PCP‐IPA**; PCP  =  porous coordination polymer, IPA  =  isophthalic acid, DPG  =  meso‐α,β‐di(4‐pyridyl) glycol)) with parallel‐aligned linearly extending isophthalic acid units along a one‐dimensional channel with dimensions of 4.7 × 5.6 Å^2^. **PCP‐IPA** was found to produce high‐purity C_3_H_6_ (99.99%) [325]. **PCP‐IPA** also achieved a separation selectivity of 2.48 and a separation potential of 1.20 mol/L for an equimolar C_3_H_8_/C_3_H_6_ binary mixture. Computational studies indicated that the periodically expanded and parallel‐aligned aromatic moieties acted as a paraffin nano‐trap, engaging the exposed hydrogen atoms of C_3_H_8_ through hydrogen bonding and van der Waals interactions. (Figure 15b).

In 2023, the incorporation of fluorinated functional groups into the confined pore space of a MOF was reported in **FDMOF‐2**, Zn_2_(BDC‐(CF_3_)_2_)_2_(DABCO) (BDC‐(CF_3_)_2_ = 2,5‐bis(trifluoromethyl) terephthalate, DABCO = 1,4‐diazabicyclo [2.2.2]octane)). **FDMOF‐2** effected C_3_H_8_‐selective separation for an equimolar C_3_H_8_/C_3_H_6_ mixture [507]. IAST selectivity of 2.18 for C_3_H_8_/C_3_H_6_ (v/v = 1/1) separation was measured for **FDMOF‐2**. SCXRD studies revealed that the tailored pore confinement in **FDMOF‐2** facilitates stronger and multiple attractive interactions (C─H···π interactions and hydrogen bonding) between the ‐CF_3_ moieties and C_3_H_8_ (Figure 15c), enabling high C_3_H_6_ purity (99.99%). The separation performance of **FDMOF‐2** was maintained even at 70% relative humidity.

Machine learning is increasingly being used fpr prediction of materials properties. In 2024, D. Li and colleagues employed computational screening methods of existing MOFs for C_3_H_8_/C_3_H_6_ separation, identifying **JNU‐90** (Zn(4,4′‐(hexafluoroisopropylidene) bis(benzoic acid)) as a promising candidate [506]. IAST selectivity of 2.7 was obtained, a number consistent with the computational results. H‐bonding and C─H···π interactions between C_3_H_8_ and the ‐CF_3_ functional groups in **JNU‐90** are thought to play a role in facilitating the observed C_3_H_8_/C_3_H_6_ selectivity. (Figure 15d).

**FIGURE 16:**
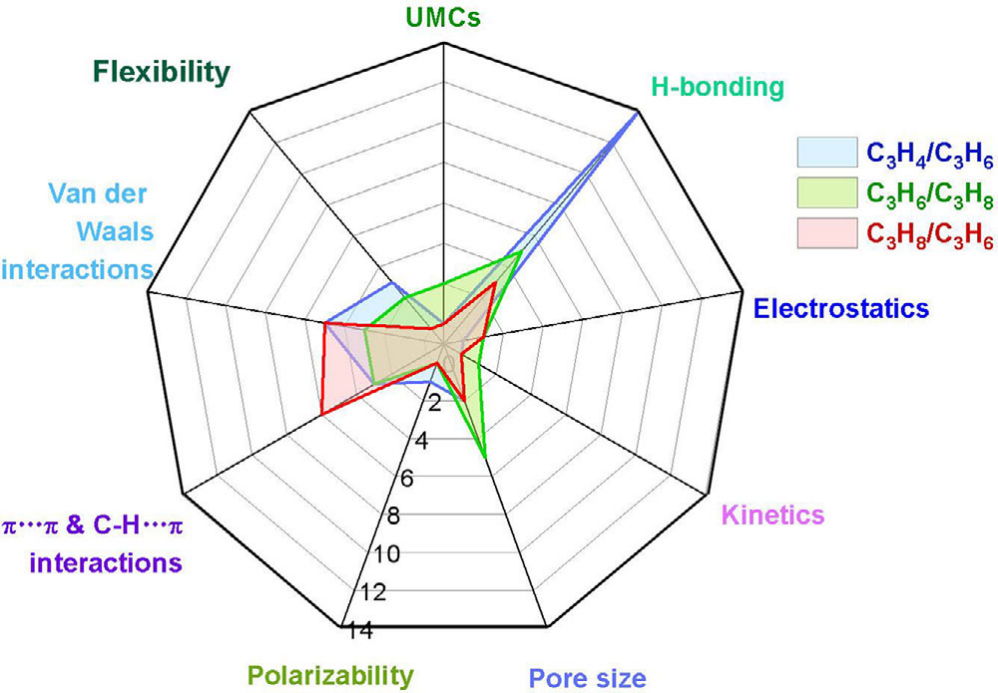
Radar plot presenting the contribution of nine separation mechanisms (and their combinations) among the 15 top‐performing reticular sorbents that exhibit binary C_3_H_4_/C_3_H_6_, C_3_H_6_/C_3_H_8,_ and C_3_H_8_/C_3_H_6_ adsorption selectivities. Each concentric enneagon represents one sorbent, while the central enneagon represents two.

For the three C3 HC binary pair separations discussed above, the distinct structural and physicochemical characteristics (of C_3_H_4_, C_3_H_6_, and C_3_H_8_) resulted in different primary separation mechanism(s) for each pair. In the case of C_3_H_4_‐selective separation over C_3_H_6_, hydrogen bonding was found to play the key role, as revealed by Figure 16. Similar to C_2_H_2_, C_3_H_4_ has relatively acidic alkyne hydrogen atoms, which can act as hydrogen bond donors to heteroatoms that can serve as hydrogen bond acceptors. For C_3_H_6_ separation over C_3_H_8_, it was found that reticular sorbents with hydrogen acceptors and appropriate pore sizes generate stronger noncovalent interactions with C_3_H_6_ than C_3_H_8_. As for C2 HCs, one‐step C_3_H_8_‐selective separation over C_3_H_6_ is more challenging. Designing sorbents with electron‐rich groups that facilitate the formation of C─H···π, π···π, and van der Waals interactions between C_3_H_8_ and the sorbents was found to be the most effective strategy.

### 3.4 C1‐C3 Multicomponent Separation

Industrial production of HCs through steam cracking of crude petroleum generates unwanted by‐products. As detailed above, the separation of binary mixtures using HC‐selective adsorbents has been addressed. However, from a practical perspective, the separation of a more complex multicomponent gas mixture is more reflective of real‐world scenarios. This necessitates a more nuanced level of molecular recognition capability in physisorbents (Table 13). To design reticular physisorbents with optimal separation abilities for multicomponent mixtures, an understanding of structure‐function relationships is needed. Table 13 presents the leading reticular sorbents reported to date for C1‐C3 multicomponent hydrocarbon separations. Sorbents for multicomponent mixtures are typically reported with single‐component selective sorption from mixtures, with one binary pair playing a dominant role. The mechanisms of C1‐C3 separation are similar to those presented for binary separations, for three types of reticular sorbents, HUMs, MOFs, and COFs, in this context.

In 2012, B. Chen, Krishna, and co‐workers reported a MOF, **[Cu_3_(H_2_L)(H_2_O)_3_**], **UTSA‐34** (UTSA = University of Texas at San Antonio; H_8_L = 1,2,4,5‐tetra(5‐isophthalate)benzene), with high separation capacity and selectivity for C2 HCs over CH_4_ [425]. The activated form of **UTSA‐34b** possesses pore cavities of 12.8 Å and high‐density Cu(II) UMCs, which facilitate a high separation capacity of 3.0 mol kg^−1^ and a selectivity of 35 for the separation of C2 HCs from CH_4_ at 298 K. In the same year, B. Chen and co‐workers utilized H_3_BTN (6,6′,6′′‐benzene‐1,3,5‐triyl‐2,2′,2′′‐trinaphthoic acid and Cd(NO_3_)_2_·4H_2_O to synthesize **Cd_3_(BTN)_2_(H_2_O)_3_(DMF)_6_** (**UTSA‐35a**) [411]. With pore dimensions of 7.7 × 5.8 Å, **UTSA‐35a** demonstrated high selectivities for multiple components, with selectivities for C_3_H_6_/CH_4_ (1/1) of 80 and C_3_H_8_/CH_4_ (1/1) of 75. Van der Waals interactions were found to enable the observed selective adsorption of higher HCs.

**FIGURE 17:**
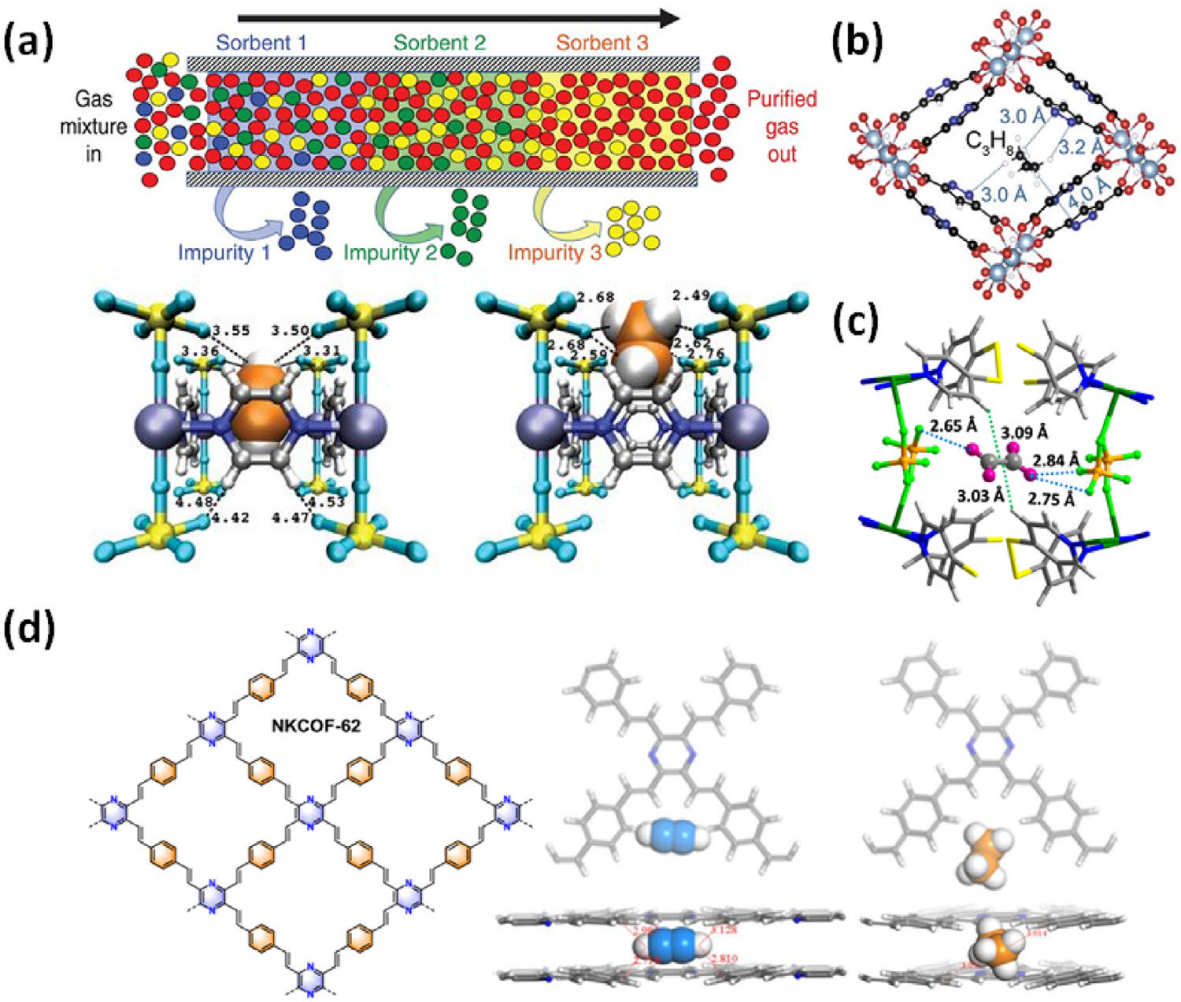
Examples of top‐performing reticular sorbents for separating multicomponent C1‐C3 gas mixtures with high selectivity: (a) **SIFSIX‐3‐Ni** [70], (b) **MOF‐303** [533], (c) **BFFOUR‐Cu‐dpds** [525], and (d) **NKCOF‐62** [413] with feed ratio, temperature, and total pressure set at 1:1, 298 K, and 1 bar, respectively. (Reprinted with permission from ref. [70]: Copyright 2019, the Authors; ref. [533]: Copyright 2022, the Authors; ref. [525]: Copyright 2024, the Authors; ref. [413]: Copyright 2023, American Chemical Society.).

In 2019, Zaworotko, Chen, and colleagues reported three ultramicroporous CN sorbents that collectively achieved one‐step purification of C_2_H_4_ from a mixture of C_2_H_2_/C_2_H_4_/C_2_H_6_/CO_2_, introducing the concept of synergistic sorbent separation technology (SSST) for multicomponent separations [70]. **SIFSIX‐3‐Ni** (SIFSIX = SiF_6_^2^–, 3 = pyrazine), **Zn‐atz‐ipa** (atz = 3‐amino‐1,2,4‐triazolate; ipa = isophthalate), and **TIFSIX‐2‐Cu‐i** (TIFSIX = TiF_6_^2^–, 2 = 4,4′‐dipyridylacetylene, i = interpenetrated) were packed in tandem in a column, with **SIFSIX‐3‐Ni** selectively removing CO_2_ from a C_2_H_2_/C_2_H_4_/C_2_H_6_/CO_2_ mixture, **TIFSIX‐2‐Cu‐i** capturing C_2_H_2_, and **Zn‐atz‐ipa** offering high inverse selectivity for C_2_H_6_. Breakthrough experiments confirmed that the SSST approach produced high‐purity C_2_H_4_ in one step from a four‐component gas mixture. Computational studies revealed that hydrogen bonding (C‐H···F) between the hydrogen atoms of HCs and TiF_6_^2−^ anions, along with electrostatic interactions between CO_2_ molecules and electronegative fluorine atoms, enabled the observed separation (Figure 17a).

J. Li and colleagues reported in 2023 a record‐high IAST selectivity of 5114 for the separation of C_3_H_8_/CH_4_ (v/v = 5:85) using **MOF‐303** (Al(OH)(PZDC), PZDC = 1‐*H*‐pyrazole‐3,5‐dicarboxylate) [533]. High surface polarity, combined with a high density of heteroatoms and an appropriate pore diameter (5‐7 Å), is the key factor that was attributed to the observed separation performance (Figure 17b). **MOF‐303** was shown to efficiently separate C_3_H_8_/C_2_H_6_/CH_4_ through ternary breakthrough experiments. Most recently, in 2024, S. S. Chen, X. Z. Guo, and colleagues reported on a paddle‐wheel Co(II)‐MOF, **FNU‐2** (Co_2_(NTA)(bpy)_2_, NTA = 4,4′,4″‐tricarboxylate triphenylamine), that purified CH_4_ from a C_3_H_8_/C_2_H_6_/CH_4_ ternary mixture, achieving a selectivity of 638.9 for C_3_H_8_/CH_4_ (v/v = 5:85) [519]. Computational studies revealed that van der Waals interactions between guest molecules and **FNU‐2** played a crucial role in influencing its separation performance. J. Wang and colleagues, also in 2024, reported that **BFFOUR‐Cu‐dpds** (Cu(dpds)_2_(BF_4_)_2_, BFFOUR  =  BF_4_^−^, dpds = 4,4′‐bipyridinedisulfide) simultaneously sieved C_2_‐C_4_ olefins from their corresponding paraffins [525]. Interlayer spaces were opened through hydrogen bonding and C─H···π sorbent‐sorbate interactions induced by the unsaturated C = C bonds in olefins (Figure 17c). **BFFOUR‐Cu‐dpds** simultaneously separated olefins from paraffins in an equimolar six‐component mixture of C_2_H_4_/C_2_H_6_/C_3_H_6_/C_3_H_8_/n‐C_4_H_8_/n‐C_4_H_10_. High‐purity C_2_H_4_ (> 99.99%) was obtained in a second column using granular porous carbons.

In 2023, Z. Zhang's group reported a robust olefin‐linked COF, **NKCOF‐62**, synthesized through the melt polymerization method using tetramethylpyrazine and terephthalaldehyde as monomers [413]. **NKCOF‐62** ushers in cost‐effective, kilogram‐scale fabrication of olefin‐linked COFs in a single pot reaction. With a pore size of 8 × 8 Å^2^, **NKCOF‐62** was found to facilitate selective adsorption of C_2_H_2_ and C_2_H_6_ over C_2_H_4_, allowing polymer‐grade C_2_H_4_ to be obtained directly from C_2_H_2_/C_2_H_6_/C_2_H_4_ (1/1/1) mixtures through a one‐step process. GCMC simulations indicated that noncovalent interactions between C2 HCs and the pore walls of **NKCOF‐62** contributed to the separation of C_2_H_2_ and C_2_H_6_ over C_2_H_4_ (Figure 17d).

**FIGURE 18:**
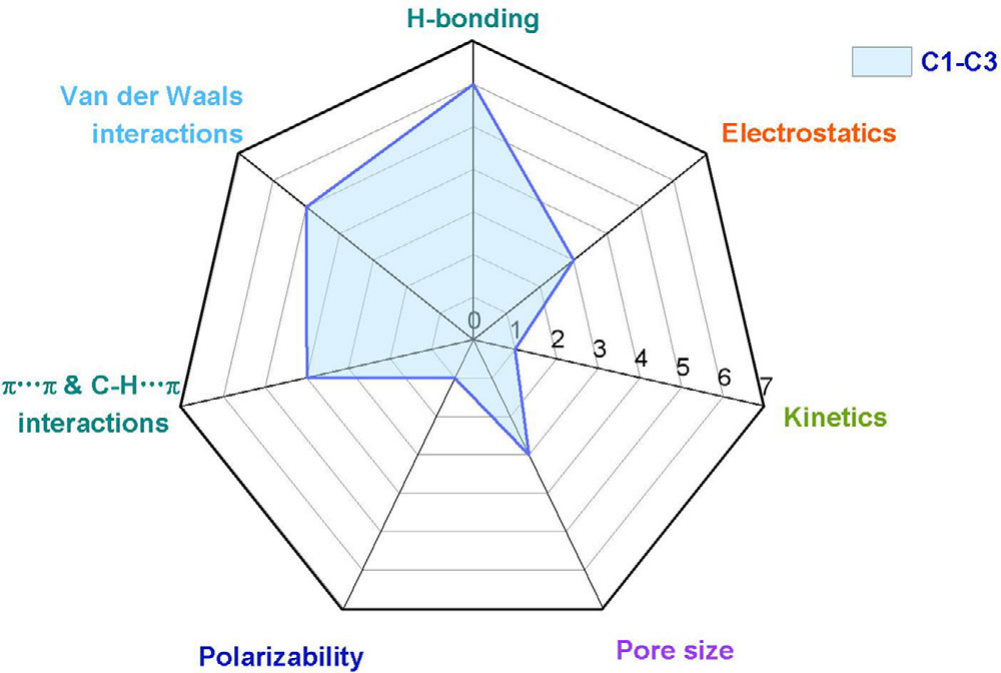
Radar plot illustrating the contribution of seven separation mechanisms (and their combinations) across the 15 top‐performing reticular sorbents, each of which demonstrates C1–C3 HC adsorption selectivities. Every concentric heptagon denotes an individual sorbent, with the central heptagon symbolizing one sorbent.

For the separation of multicomponent C1–C3 mixtures, seven mechanisms were identified among the 15 top‐performing reticular sorbents: hydrogen bonding; electrostatics; kinetics, pore size; polarizability; π···π and C─H···π interactions; and van der Waals interactions (Figure 18). This finding is consistent with the results in the sections on binary C1, C2, and C3 separations. Overall, hydrogen bonding, π···π and interactions, and van der Waals interactions are the three primary mechanisms behind the top‐performing C1‐C3 reticular sorbents.

### 3.5 C4 Separation

C4 HCs, including 1,3‐butadiene (C_4_H_6_), 1‐butene (*n*‐C_4_H_8_), isobutene (*i*‐C_4_H_8_), cis‐2‐butene (2‐C_4_H_8_), isobutane (*i*‐C_4_H_10_), and n‐butane (*n*‐C_4_H_10_) serve as raw materials for a range of products such as synthetic rubbers and elastomers, resins, detergents, fuels, plastics, and more [717]. Generally, C4 olefins are produced through fluidized catalytic cracking (FCC), steam cracking of petroleum or methanol‐to‐olefins (MTO) processes [718]. C4 olefins produced from these processes usually comprise 30%–60% C_4_H_6_, 10–30% *i*‐C_4_H_8_, 10–20% *n*‐C_4_H_8_, 2–6% 2‐C_4_H_8_, 1–3% *i*‐C_4_H_10_, and 3–10% *n*‐C_4_H_10_ [719]. However, due to their similar molecular shapes and physicochemical properties, developing adsorbents for C4 olefin separation is a challenge. Extractive distillation is the established method for obtaining high‐purity C_4_H_6_ (greater than 99.5%) and operates at high pressure (3 bar) and elevated temperatures (323 to 393 K) with tall towers (over 110 trays). This process is energy‐intensive, environmentally unfriendly, and carries the risk of C_4_H_6_ polymerization at high temperatures [720]. Reticular adsorbents with tailored pore size, shape, and controllable surface functionalization offer an opportunity to address these handicaps and develop an economically viable separation process without the need for heat (Table 14). Table 14 presents the leading reticular sorbents reported to date for C4 isomer separation, arranged in decreasing order of adsorption selectivity. The pore sizes of sorbents with high selectivity for C4 isomer separation (4–12 Å) are generally larger than those for C1–C3 sorbents, due to the larger molecular size of C4 isomers. However, an analysis of studies on sorbents for C4 separation reveals that pore size is not a key parameter in achieving the separation. Instead, noncovalent interactions, primarily hydrogen bonding, tend to play a role in the separation process. The regeneration temperatures for the foregoing C4 sorbents typically fall within the range of 323 K to 423 K. Seven studies reporting high selectivity are discussed below.

**FIGURE 19:**
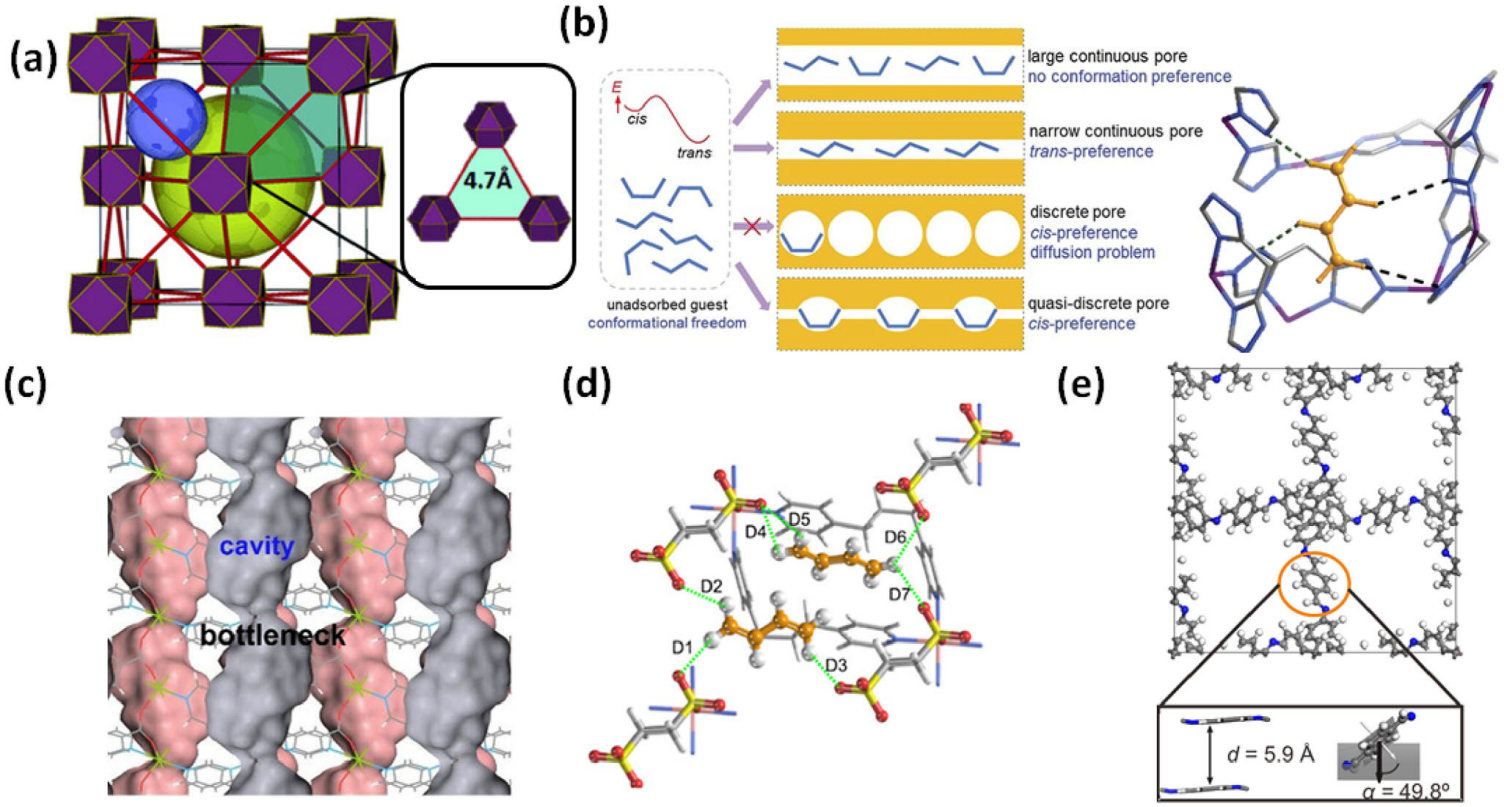
Examples of top‐performing reticular sorbents for C4 isomer separation with high selectivity: (a) **fcu‐MOF** [569], (b) **Zn_2_(btm)_2_** [82], (c) **MnINA** [577], (d) **ZU‐601** [578], and (e) **COF‐300** [583] with feed ratio, temperature, and total pressure set at 1:1, 298 K, and 1 bar, respectively. (Reprinted with permission from ref. [569]: Copyright 2015, Wiley‐VCH GmbH; ref. [82]: Copyright 2017, The American Association for the Advancement of Science; ref. [577]: Copyright 2019, Wiley‐VCH GmbH; ref. [578]: copyright 2024, American Chemical Society; ref. [583]: Copyright 2022, Institute of Process Engineering, Chinese Academy of Sciences.).

Assen et al. reported an **fcu‐MOF** with precisely tuned pore size to achieve efficient molecular sieving of *n*‐C_4_H_10_ from i‐C_4_H_10_ in 2015 [569]. This rare‐earth (RE) **fcu‐MOF** was built from 12‐connected RE hexanuclear clusters and formed well‐defined triangular windows as well as interconnected octahedral and tetrahedral cages (Figure 19a). The **Y‐fcu‐MOF** analogue ((CH_3_)_2_NH_2_)_2_ [Y_6_(μ_3_‐OH)_8_(fum)_6_(H_2_O)_6_], fum = fumarate) excluded branched paraffins from normal paraffins. Its window of approximately 4.7 Å diameter, considered a sorbate‐size cut‐off, enabled sieving of branched paraffins from normal paraffins, with *n*‐C_4_H_10_ and *i*‐C_4_H_10_ capacities of 1.97 and 0.05 mmol/g, respectively.

Conventional adsorbents typically favour adsorption of smaller, higher‐polarity C_4_H_6_ over other C4 HCs. In 2017, X. M. Chen and colleagues reported inverse selectivity for C4 HCs by **Zn_2_(btm)_2_** (H_2_btm = bis(5‐methyl‐1*H*‐1,2,4‐triazol‐3‐yl)methane), which is comprised of quasi‐discrete pores (Figure 19b) [82]. As revealed by SCXRD and computational studies, the pores of **Zn_2_(btm)_2_** can induce conformational changes in the flexible guest molecules, weakening C_4_H_6_ adsorption through a significant bending energy penalty. This allows C_4_H_6_ to be purified at 298 K (>99.5%) using a guest conformation‐controlling adsorbent that elutes C_4_H_6_ first, followed by C_4_H_10_, *n*‐C_4_H_8_, and *i*‐C_4_H_8_.

In 2019, H. Xing and colleagues reported a flexible MOF, **MnINA** (INA = isonicotinate), which features one‐dimensional pore channels with periodic cavities connected by narrow windows. **MnINA** was found to exhibit *n*‐/*i*‐C_4_H_8_ selectivity [577]. With an optimal pore size of 4.62 Å, **MnINA** exhibits steep adsorption isotherms and a high capacity of 1.79 mmol g^−1^ for *n*‐C_4_H_8_ (4.46 Å) enabled by C−H⋅⋅⋅π interactions (Figure 19c). At the same time, bottlenecks create barriers for larger *i*‐C_4_H_8_ molecules (4.84 Å) in the gate‐opening pressure range of 0‐0.1 bar. **MnINA** achieved selectivity of 327.7 (based on the initial slope ratio, *n*‐/*i*‐C_4_H_8_ v/v = 1/1) for *n*‐/*i*‐C_4_H_8_. In 2024, the same group reported two flexible sulfonate‐functionalized organic frameworks, **ED‐6‐Cu** (**ZU‐601**) and **13‐6‐Cu** (**ZU‐602**) (ED = 1,2‐ethanedisulfonate, 13 = benzene‐1,3‐disulfonate, 6 = 1,3‐di(4‐pyridyl)propane, ZU = Zhejiang University) for C4 separation [578]. Thanks to the right aperture size enabled by the flexibility of coordinated organic ligands, **ZU‐601** (6.7 × 2.2 Å^2^) and **ZU‐602** (6.8 × 2.5 Å^2^), which are pillared with different sulfonate anions, can discriminate between C4 olefin isomers. Their layered structure allows the utilization of both intra‐ and interlayer space so that **ZU‐601** exhibits a record‐high C_4_H_6_ adsorption capacity of 2.90 mmol/g (0.5 bar, 298 K) and a high uptake ratio for C_4_H_6_/*n*‐C_4_H_8_ (207), C_4_H_6_/*trans*‐C_4_H_8_ (10.1). DFT‐D calculations revealed that the C_4_H_6_ molecule within **ZU‐601** binds to the oxygen atoms of three 1,2‐ethanedisulfonate anions from adjacent layers at shorter distances than *n*‐C_4_H_8_ (Figure 19d).

In 2022, C. Zhong's team reported a flexible MOF, **Mn‐bpdc** (bpdc = 2,2′‐bipyridine‐4,4′‐dicarboxylate), with a gate‐opening effect for C_4_H_6_ separation from C4 HCs (*n*‐C_4_H_8_, *i*‐C_4_H_8_, *n*‐C_4_H_10_, *i*‐C_4_H_10_) [582]. In this study, C_4_H_6_ triggers the gate‐opening of **Mn‐bpdc** at 0.13 bar and 298 K, allowing C_4_H_6_ uptake, whereas other C4 HCs did not induce gate‐opening even at 1 bar. The selectivities of **Mn‐bpdc** for C_4_H_6_/*n*‐C_4_H_8_ and C_4_H_6_/*i*‐C_4_H_8_ reach 40.0 and 45.0, respectively, at 298 K and 1 bar, respectively. DFT calculations indicated multiple H‐bonding interactions between the one‐dimensional channel of Mn‐bpdc and C_4_H_6_, with a static adsorption energy of C_4_H_6_ of up to 74.33 kJ mol^−1^.

In 2020, H. Xing and co‐workers reported **ZU‐36‐Co** (**GeFSIX‐3‐Co**, GeFSIX = GeF_6_^2−^, 3 = pyrazine) for separating both C4 linear/branched olefins (*n*‐/*i*‐C_4_H_8_) and paraffin isomers (*n*−/*i*‐C_4_H_10_) [572]. With a pore size ranging between 3.82‐5.25 Å, **ZU‐36‐Co** adsorbs *n*‐C_4_H_8_ (2.35 mmol/g) and *n*‐C_4_H_10_ (2.20 mmol/g) while excluding *i*‐C_4_H_8_ and *i*‐C_4_H_10_. This sieving effect highlights the importance of pore window size for effective C4 gas separation. **ZU‐36‐Co** demonstrated *n*‐/*i*‐C_4_H_8_ uptake ratio of 13.8, an *n*‐/*i*‐C_4_H_10_ uptake ratio of 18.3, and IAST selectivity values of 2050 for *n*‐/*i*‐C_4_H_8_ (v/v = 1/1) and 140.4 for *n*‐/*i*‐C_4_H_10_ (v/v = 1/1). Computational studies revealed that the H‐bonding interactions between F atoms from GeF_6_^2−^ anions and *n*‐C_4_H_8_ (or *n*‐C_4_H_10_) are key to trapping linear C4 isomers while excluding branched C4 isomers.

In 2022, A. Zheng's group reported a series of COFs, **COF‐300** (dia‐c5), **COF‐320** (dia‐c9), and **azo‐COP‐2** (dia‐c5), for the separation of C4 HCs. Among these, **COF‐300** (dia‐c5) demonstrated the highest selectivity (*i*‐C_4_H_8_/*trans*‐C_4_H_8_, 38.4; *i*‐C_4_H_8_/*cis*‐C_4_H_8_, 35.8) [583]. The exceptional separation performance of **COF‐300** (dia‐c5) is attributed to its optimal interpenetration isomerism‐controlled interlayer space and the pedal motion‐altered shape of the 1D channel (Figure 19e).

**FIGURE 20:**
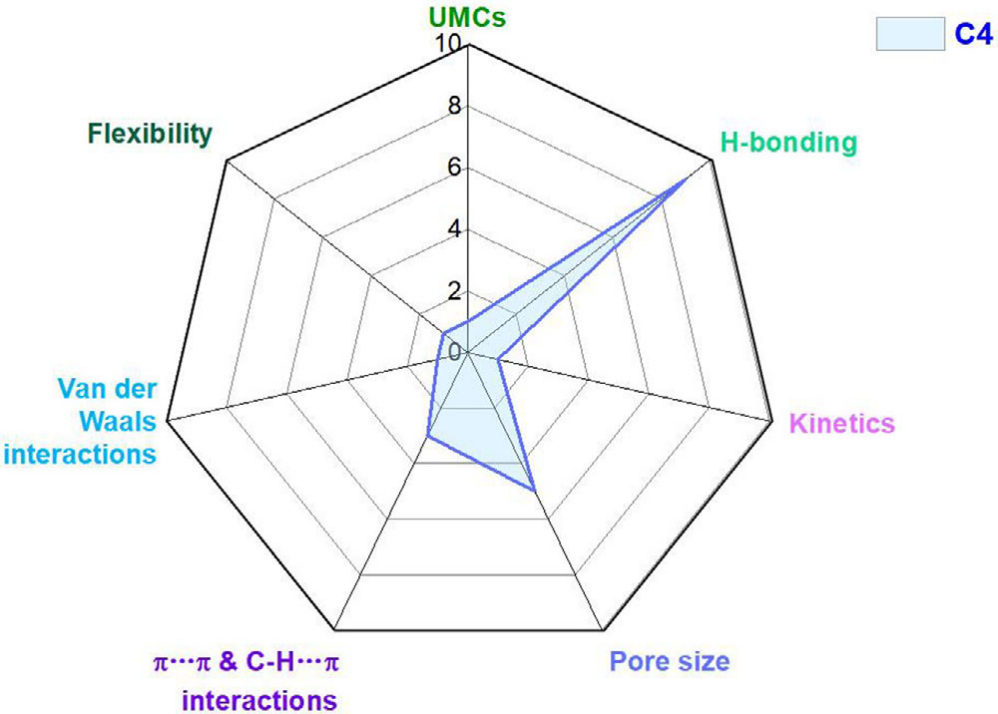
Radar plot presenting the contribution of seven separation mechanisms (and their combinations) among the 15 top‐performing reticular sorbents that exhibit binary or multiple C4 HCs adsorption selectivities. Each concentric heptagon denotes one sorbent, while the central heptagon represents two.

For the separation of C4 HCs, seven mechanisms were identified among the 15 top‐performing reticular sorbents for selective C4 adsorption. These mechanisms include UMCs, hydrogen bonding, kinetics, pore size (molecular sieving), π···π & C─H···π interactions, van der Waals interactions, and flexibility (Figure 20). Overall, pore size and hydrogen bonding were found to play prominent roles in C4 separation. The best‐performing molecular sieve exhibited nearly infinite selectivity for *n*‐C_4_H_10_/*i*‐C_4_H_10_ separation. However, achieving the precise design of an ideal pore size in sorbents remains a challenge. Hydrogen bonding interactions between C4 molecules (e.g., C_4_H_6_ and *n*‐C_4_H_10_) and reticular sorbents also enable C4 separation.

## 4 HC Separation in Vapor and/or Liquid Phase

HCs with higher molecular weights and bulkier molecular volumes typically exist as liquids at 298 K and 1 bar. While much of the focus on physisorbents has been on adsorption and purification of lower molecular weight gaseous HCs, separation of HCs in the vapor/liquid phases has also been addressed, even though it remains understudied. C6 and C8 HC isomers are the most widely studied sorbates as detailed below [721].

### 4.1 C6 Separation

Separation of C6 HCs has focused upon two subsets of industrial relevance: C6 alkane isomers (*n*‐hexane (*n*‐HEX), 3‐methylpentane (3MP), 2‐methylpentane (2MP), 2,2‐dimethylbutane (22DMB), and 2,3‐dimethylbutane (23DMB)); cyclic C6 HCs (benzene, Bz, and cyclohexane, Cy). Table 15 presents the leading reticular sorbents reported to date for the separation of these two subsets arranged in decreasing order of adsorption selectivity within each category. For C6 alkane isomers, most sorbents thus far studied favour *n*‐HEX selective sorption, presumably because of the more linear shape and smaller cross‐section of *n*‐HEX compared to its branched isomers (2MP, 3MP, 22DMB, 23DMB). The pore sizes of reticular sorbents studied for C6 alkane isomer separation range from 3 to 12 Å, with pore size being key to separation performance. For C6 cyclic HC separation, the reticular sorbents studied also exhibit pore sizes between 3 and 12 Å, but the separation mechanism primarily relies on π interactions between the sorbent and sorbate. Six studies on C6 alkane isomers and ten studies on C6 cyclic HCs have reported reticular sorbents with high selectivity and are discussed below.

#### 4.1.1 C6 Alkane HCs

C6 alkane HCs are commodities in the petrochemical industry. The quality of gasoline is related to its combustion efficiency and proclivity to detonate. To obtain high‐quality gasoline, an effective approach is to increase the degree of branching [5, 722, 723, 724, 725, 726, 727, 728]. Performance is measured by the research octane number (RON); isomers with a higher degree of branching usually possess higher RONs. The five isomers of C6 alkane HCs produced as primary products from the light naphtha isomerization process, *n*‐HEX, 3MP, 2MP, 22DMB, and 23DMB, have RONs of 24.8, 73.4, 74.5, 91.8, and 101.7, respectively [623]. Octane enhancement can be achieved by effectively separating alkane isomers with varying degrees of branching. However, the similar physicochemical properties of hexane isomers present significant challenges for C6 alkane separation. In particular, the similar boiling points of C6 alkane HCs (Table 2) make separation by conventional fractionation energy‐intensive. In this context, adsorptive separation using porous materials offers an energy‐efficient alternative to the traditional methods. The distinct linear and branched shapes of C6 alkane isomers mean that control over pore size and shape is likely impact separation performance.

Early work on C6 alkane separation was reported in 2006 by B. Chen, S. Dai, and colleagues. They studied a flexible **MOF‐508**, [(Zn(BDC)(4,4′‐bpy)_0.5_] (BDC = 1,4‐benzenedicarboxylic acid), a primitive cubic framework with one‐dimensional channels of approximately 4 × 4 Å^2^ in cross‐section [658]. Selective separation of n‐HEX from n‐HEX/2MP/22DMB mixtures was achieved using **MOF‐508** as a GC column. In addition, other HC mixtures were separated by GC, and the guest‐loaded/activated structures of **MOF‐508** were determined through SCXRD. The authors’ analysis indicated that the GC separation of C6 alkanes was based on van der Waals interactions enabled by subtle size‐ and shape‐selective matching.

**FIGURE 21:**
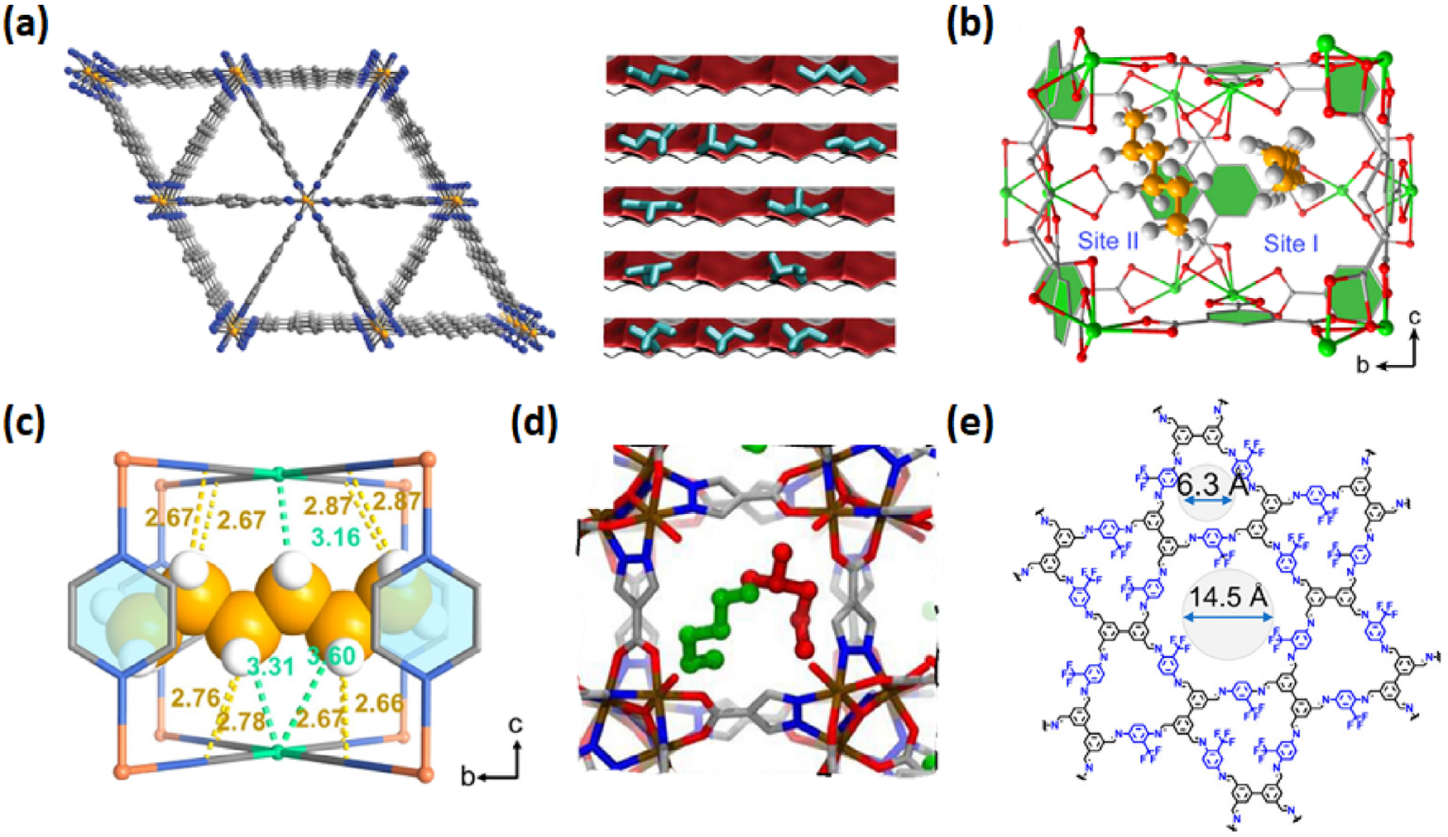
Examples of top‐performing reticular sorbents for C6 isomers’ separation with high selectivity: (a) **Fe_2_(BDP)_3_** [71] with feed ratio and temperature set at 1:1 and 298 K, respectively. (b) **UU‐200** [615] with feed ratio and temperature set at 1:1 and 298 K, respectively. (c) **CopzNi** [617] with feed ratio and temperature set at 1:1 and 303 K, respectively. (d) **MIP‐214** [651] with feed ratio and temperature set at 1:1 and 298 K, respectively. (e) **BTAPa‐CF_3_** [655] with feed ratio and temperature set at 1:1 and 298 K, respectively. (Reprinted with permission from ref. [71]: Copyright 2013, The American Association for the Advancement of Science; ref. [615]: Copyright 2022, Wiley‐VCH GmbH; ref. [617]: Copyright 2023, The Elsevier B.V.; ref. [651]: Copyright 2024, Wiley‐VCH GmbH; ref. [655]: Copyright 2024, American Chemical Society.).

In 2013, Long and co‐workers reported that self‐assembly of a rigid and nearly planar dipyrazolate linker ligand, 1,4‐benzenedipyrazolate (BDP^2−^), with Fe(III), afforded a stable 3D MOF, **Fe_2_(BDP)_3_**. **Fe_2_(BDP)_3_** features triangular channels with sharp‐angled crevices, which enabled the separation of hexane isomers according to their degree of branching (Figure 21a) [71]. The linear shape of *n*‐HEX enabled it to be selectively adsorbed from *n*‐HEX/2MP/3MP/22DMB/23DMB mixtures, driven by stronger van der Waals contacts than branched alkanes.

In 2022, D. Zhao and co‐workers reported a bismuth‐MOF synthesized from Bi^3+^ and benzene‐1,3,5‐tricarboxylic acid (H_3_BTC), **UU‐200**, that was effective for the isolation of di‐branched alkanes from their isomers by combining two mechanisms, molecular recognition and molecular sieving [615]. The auxetic structure of **UU‐200**, featuring honeycomb‐like cavities connected by narrow pore sizes (5 × 5 Å^2^) enabled rejection of dibranched alkanes while offering high capacities for linear and monobranched isomers (Figure 21b). IAST calculations highlighted **UU‐200**’s separation ability, with equimolar selectivities for *n*‐HEX/22DMB of 1 × 10^5^, for 3MP/22DMB of 3 × 10^4^, and for *n*‐HEX/3MP at 160. This was confirmed by breakthrough tests at 298 K, which produced an HC mixture with a RON >96. DFT calculations revealed that **UU‐200** binds to *n*‐HEX through multiple H‐bonding and C─H···π interactions.

In 2023, Z. Bao and colleagues reported that **M(pz) [Ni(CN)_4_]**, **MpzNi**; M = Co, Ni, pz = pyrazine, can separate ternary mixtures into individual components through molecular sieving and temperature programming [617]. The multiple UMCs and electron‐rich environment of the pore channel enabled **CopzNi** to exhibit high IAST selectivities: 420.0 for 2MP/22DMB (50/50, *v*/*v*) at 303 K; 171.7 for *n*‐HEX/2MP (50/50, *v*/v) at 363 K (Figure 21c).

In 2024, Serre and colleagues reported a multi‐cage microporous Fe(III)‐MOF, **MIP‐214** (MIP: Materials of the Institute of Porous Materials of Paris, Fe_3_(OH)(H_2_O)_2_(μ_3_‐O)(PyC)_3_, PyC = 4‐pyrazolecarboxylate) that achieved separation of a mixture of high RON di‐branched hexane and mono‐branched pentane isomers from their low RON counterparts, resulting in a product with a RON of 92 [651]. The narrow windows and large cages in **MIP‐214** were generated by using a short ditopic ligand, H_2_PyC, to replace the bdc linker in **MCF‐48**, Fe_3_(μ_3_‐O)(bdc)_3_(L^T^)_3_, bdc = 1,4‐benzenedicaboxylate (Figure 21d). Experimental data revealed that **MIP‐214** facilitates separation based on the degree of branching: linear (*n*‐HEX) ≫ mono‐branched (2MP, 3MP) ≫ di‐branched (22DMB, 23DMB). Also in 2024, X. P. Yan and colleagues reported two 2D COFs, **BTAPa‐CF_3_** and **TbPa‐CF_3_**, and studied their HC separation properties [655]. Compared to **TbPa‐CF_3_**, which has a uniform pore dimension of 14.3 × 14.3 Å^2^, **BTAPa‐CF_3_** possesses two pores of dimensions 6.3 × 6.3 and 14.5 × 14.5 Å. **BTAPa‐CF_3_** not only has a richer pore structure but also a higher density of trifluoromethyl functional groups, giving it superior performance in a bonded capillary column for *n*‐HEX‐selective separation from C6 alkane mixtures (Figure 21e).

#### 4.1.2 C6 Cyclic HCs

Separation of Bz and Cy is another challenge for the petrochemical industry, with their binary mixtures having industrial utility. Their effective separation is difficult due to their similar molecular geometries, close boiling points (353.25 K and 353.85 K, respectively), and their tendency to form azeotropic mixtures [4, 729]. Cy is a feedstock for producing cyclohexanone and cyclohexanol, which are, in turn, used in the production of nylon‐66 and nylon‐6 [5, 724]. Industrially, Cy is primarily produced by the catalytic hydrogenation of Bz, resulting in a product that is a Bz/Cy binary mixture [730, 731]. Moreover, volatile organic compounds (VOCs) like Bz are a class of toxic pollutants that contribute to both indoor and outdoor air pollution, causing environmental and health issues even at trace concentrations [732, 733, 734, 735]. Instead of using heat‐driven distillation to separate Bz/Cy mixtures, adsorptive separation with porous reticular sorbents offers an alternative approach for selectively adsorbing either Bz over Cy (Bz/Cy), or Cy over Bz (Cy/Bz). Cy adopts either a boat or chair conformation and is bulkier than Bz, a planar molecule with a narrow cross‐section and a π‐electron cloud. MOF adsorbents typically adsorb Bz over Cy due to stronger binding affinities driven by host‐guest interactions, including π···complexation, π···π interactions, and H···π interactions [62, 679, 687]. Examples of Cy/Bz separation are relatively rare, their separation mechanisms primarily relying on van der Waals interactions between the reticular sorbents and Cy as well as appropriate pore size or shape [85].

In 2007, the first example of Bz‐selective adsorption in MOFs was reported by J. S. Chang and co‐workers with **MIL‐101(Cr)**, (Cr_3_F(H_2_O)_2_O [bdc)_2_]_3_, bdc = 1,4‐benzene dicarboxylate) [736]. High Bz uptake of 16.7 mmol·g^−^^1^ at P/P_0_ = 0.5 was observed, surpassing that of porous materials like mesoporous silica **SBA‐15** (3.0 mmol·g^−^^1^), **HZSM‐5 zeolite** (1.9 mmol·g^−^^1^), and commercial **activated carbon** (8.0 mmol/g). In the same year, S. Kitagawa's group reported Bz‐selective sorption over Cy by a flexible **[Zn(μ_4_‐TCNQ‐TCNQ)bpy]** (TCNQ = 7,7,8,8‐Tetracyano‐*p*‐quinodimethane) [62]. The undulating channel in the reticular sorbent enabled CH···π interactions between the host framework and the guest molecules.

**FIGURE 22:**
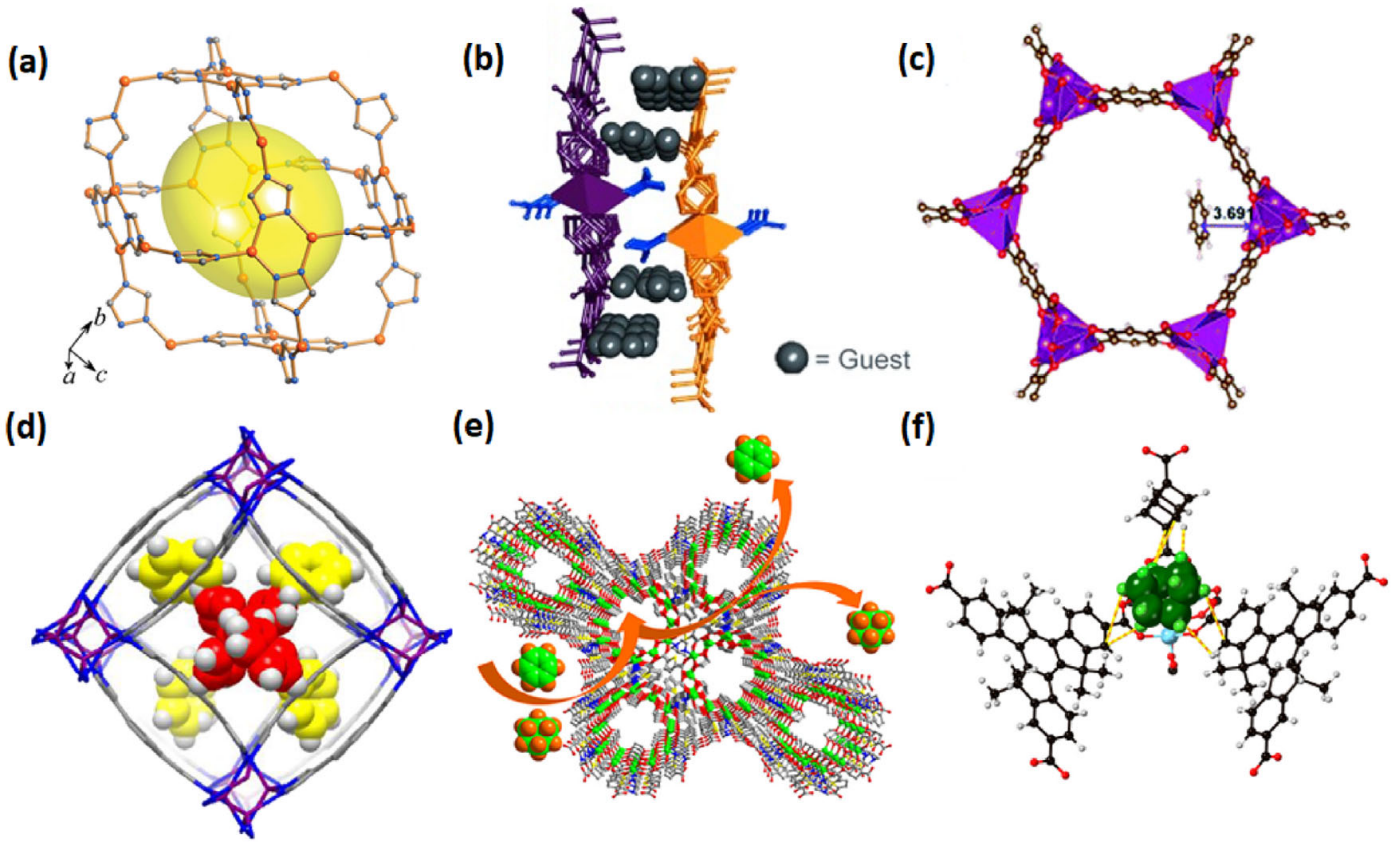
Examples of top‐performing reticular sorbents for C6 isomers separation with high selectivity: (a) **MAF‐2** [682] (b) **Cu‐1‐NO_3_** [683] (c) **Mn‐MOF‐74** [679] (d) **BUT‐55** [695] (e) **Zn_12_(tdc)_6_(glycolate)_6_(dabco)_3_** [737] (f) **CUB‐30** [680] with feed ratio and temperature set at 1:1 and 298 K, respectively. (Reprinted with permission from ref. [682]: Copyright 2008, American Chemical Society; ref. [683]: Copyright 2015, Wiley‐VCH GmbH; ref. [679]: Copyright 2016, The Royal Society of Chemistry; ref. [695]: Copyright 2022, The Author(s); ref. [737]: Copyright 2019, American Chemical Society; ref. [680]: Copyright 2024, Wiley‐VCH GmbH.).

In 2008, X. M. Chen reported a flexible MOF **[Cu(etz)]_n_**, **MAF‐2** (Hetz = 3,5‐diethyl‐1,2,4‐triazole) that exhibited efficient Bz/Cy separation [682]. Structural analysis revealed that its flexible framework distorted to allow Bz molecules to diffuse through the altered apertures while Cy did not. As a result, **MAF‐2** readily adsorbed high amounts of Bz (206 mg/g) but only surface adsorption of Cy (9 mg/g at P/P_0_ = 0.96). The strong interaction between the **MAF‐2** framework and Bz was attributed to the π system and C(*sp* ^2^)‐H moieties of Bz (Figure 22a). In 2015, Ghosh and colleagues reported another flexible MOF, **Cu‐1‐NO_3_** (CuL_2_(NO_3_)_2_), which achieved Bz/Cy separation enabled by a structural transformation from a 1D porous phase to a 2D non‐porous phase [683]. **Cu‐1‐NO_3_** was synthesized based on a flexible neutral amide‐based N‐donor ligand. This guest‐induced structural transformation enabled Bz‐selective adsorption via C─H⋯π and π⋯π interactions generated between aromatic rings of **Cu‐1‐NO_3_** and Bz (Figure 22b).

In 2011, Y. Chen and co‐workers reported a 9‐connected trinuclear cluster‐based MOF, **Ni_3_(OH)(Ina)_3_(BDC)_1.5_** (Ina = isonicotinate and BDC = 1,4‐benzenedicarboxylate), for Bz/Cy separation [676] that adsorbs more Bz (22.60%) than Cy (1.40%). Bz selectivity was attributed to π⋯π interactions between Bz and the phenyl ring of the BDC linker, as well as the two types of cages in **Ni_3_(OH)(Ina)_3_(BDC)_1.5_**.

In 2016, S. Mukherjee et al. reported the sorption properties of a family of MOFs with UMCs, **M‐MOF‐74** (M_2_(dobdc) (dobdc^4−^ = 2,5‐dioxido‐1,4‐benzenedicarboxylate) or **CPO‐27‐M** or **M_2_(dhtp)** (dhtp = 2,5‐dihydroxyterephthalate), where M = Mg, Mn, Fe, Co, Ni, Cu, Zn) [679]. In this work, π‐complexation triggered Lewis acid‐base interactions between the UMCs of MOFs and Bz. **M‐MOF‐74** marked the first report of utilizing UMCs to enable Bz/Cy separation (Figure 22c).

In 2022, J. R. Li, Zaworotko, and colleagues reported a family of double‐walled MOFs, **BUT‐53** to **BUT‐58** (BUT = Beijing University of Technology) comprised of Co^2+^ or Zn^2+^ ions and ditopic pyrazolate (dipyrazolate) ligands. Bz uptakes at 298 K ranged from 2.47 to 3.28 mmol g^−^^1^ at pressures below 10 Pa [695]. In particular, **BUT‐55** (Co(BDP), BDP = H_2_BDP  =  1,4‐di(1*H*‐pyrazol‐4‐yl)benzene), a supramolecular isomer of the MOF Co(BDP), captured trace levels of Bz, producing an air stream with Bz content below acceptable limits. Importantly, **BUT‐55** also performed under humid conditions. An exceptionally high breakthrough time of 8000 h/g and Bz uptake capacity of 2.14 mmol/g were recorded for **BUT‐55** under both dry conditions and 50% RH. Its breakthrough time and capacity were reduced to 6000 h/g and 1.61 mmol/g at 80% RH, respectively. The performance of **BUT‐55** can be understood through the crystal structure of the Bz‐loaded phase (**Bz@BUT‐55**) and DFT calculations, which revealed that C─H⋯X interactions are responsible for the strong binding of Bz in a confined space suitable for Bz (Figure 22d).

In 2019, Cy/Bz separation was reported by Macreadie and co‐workers through employing the ditopic cubane‐1,4‐dicarboxylate linker (cdc) to prepare **CUB‐5** [Zn_4_O(cdc)_3_, cdc = cubane‐1,4‐dicarboxylate], an analogue of **MOF‐5** [73]. The high Bz/Cy selectivity of **CUB‐5** was found to be enabled by non‐covalent interactions between the methine groups of the cubane linker and Bz. **MOF‐5** was observed to exhibit Cy/Bz selectivity. DFT‐D3 calculations were employed to elucidate the driving factor behind this Cy/Bz selectivity and showed multiple interactions between the **MOF‐5** walls and Cy, compared to Bz driven by Cy altering its conformation to maximize interactions with the pore.

Another example of Cy/Bz separation was reported by Dybtsev and co‐workers in 2019. **[Zn_12_(tdc)_6_(glycolate)_6_(dabco)_3_]** (H_2_tdc = thiophene‐2,5‐dicarboxylic acid, glycolate = 1,2‐pentanediol, dabco = 1,4‐diazo [2.2.2] bicyclooctane), which features an alkyl chain, demonstrated adsorption selectivity for Cy/Bz of 2.5:1 in liquids and 5:1 in vapors [737]. van der Waals interactions between Cy and alkyl chains were found to contribute to this selective adsorption of Cy over Bz (Figure 22e).

In 2020, Hill and colleagues reported separation of Cy over Bz using **CUB‐30** ([Zn_4_O(hmtt)_4/3_(bpdc)_1/2_(cdc)_1/2_], hmtt = 5,5′,10,10′,15,15′‐hexamethyltruxene‐2,7,12‐tricarboxylate; bpdc = biphenyl‐4,4′‐dicarboxylate; cdc = cubane‐1,4‐dicarboxylate) and **MUF‐77** ([Zn_4_O(hmtt)_4/3_(bpdc)_1/2_(bdc)_1/2_], bdc = benzene‐1,4‐dicarboxylate) [680]. The preference for Cy adsorption in **CUB‐30** compared to **MUF‐77** at low partial pressures was attributed to the pore environment of the smaller dodecahedral pore (Figure 22f). Based on N_2_ sorption isotherms, the theoretical pore size distribution of **MUF‐77** and **CUB‐30**are 8, 18, 21 Å and 8.2, 17.5, 20.5 Å, respectively. Additionally, breakthrough curves were simulated to assess the capability of **CUB‐30** to separate Cy and Bz under dynamic conditions [196, 738]. The results indicated that **CUB‐30** can efficiently trap Cy under dynamic conditions.

**FIGURE 23:**
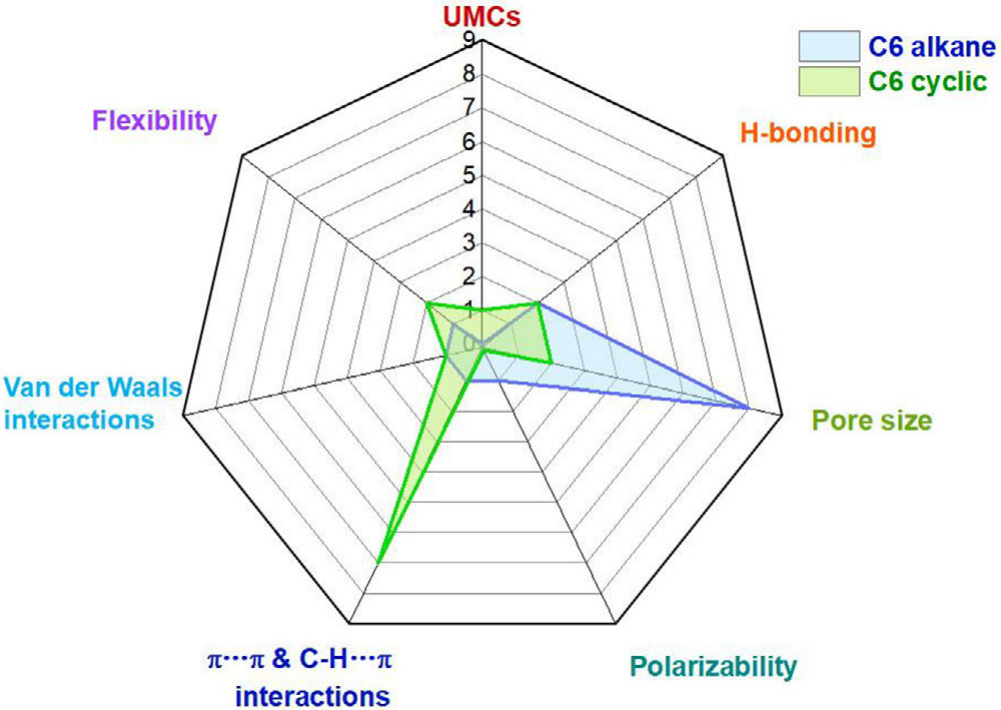
Radar plot illustrating the contribution of seven separation mechanisms (and their combinations) among the ten top‐performing reticular sorbents that exhibit binary or multiple C6 HC adsorption selectivities. Each concentric heptagon corresponds to a different sorbent, with the central heptagon representing one sorbent.

For the separation of C6 HCs, seven mechanisms were identified among the top‐performing reticular sorbents. These mechanisms include UMCs, hydrogen bonding, pore size (molecular sieving), polarizability, π···π and C─H···π interactions, van der Waals interactions, and structural flexibility, all of which contribute to C6 adsorption selectivities (Figure 23). As might have been expected, pore size plays a crucial role in C6 alkane separation, thanks to the molecular size and shape differences among C6 HCs. Specifically, *n*‐hexane, with its linear molecular shape, tends to fit better within the channels of sorbents, whereas the branched C6 alkanes tend to be excluded. For the separation of C6 cyclic HCs, π···π and C─H···π sorbate–sorbent interactions are the predominant mechanism, exploiting the difference between Bz and Cy in terms of their electronic characteristics and shapes. Specifically, Bz is planar and π‐electron‐rich, Cy is of non‐planar shape and π‐electron‐deficient.

### 4.2 C8 Aromatics Separation

C8 aromatics, which tend to be produced as mixtures which include the three xylene isomers, *para*‐xylene (PX), *ortho*‐xylene (OX) and *meta*‐xylene (MX), and ethylbenzene (EB), are each feedstocks for the synthesis of several important chemicals and polymers [4, 739]. PX is the most widely produced of the xylene isomers, accounting for 50%–60% of global xylenes production [740]. As the starting material for synthesis of terephthalic acid or dimethyl terephthalate, PX must have a purity greater than 99% for the subsequent production of polyester fiber and polyethylene terephthalate resin (PET) [741]. MX is primarily used to synthesize isophthalic acid as a co‐monomer in the production of PET‐based resin blends [742]. OX is used in the production of phthalic anhydride, which is then converted into a plasticizer [742]. EB is used in the production of polystyrene (PS) after undergoing catalytic dehydrogenation to styrene. Industrially, C8 aromatics are primarily produced through catalytic reforming of crude oil, which invariably results in the presence of other isomers [743]. Current technologies for separating C8 aromatics include distillation, crystallization, and adsorptive separation. Due to the similar boiling points and molecular sizes of the C8 aromatics, separation by distillation is costly and energy‐intensive (Table 2) [744]. Separation of aromatics through selective adsorption should be comparatively more energy‐efficient. Table 16 presents the leading reticular sorbents reported to date for the separation of the C8 aromatic isomers (OX, PX, MX, EB), arranged in decreasing order of adsorption selectivity within each category. For C8 isomer separation, more examples are reported for OX‐ or PX‐selective separation than for MX‐ or EB‐selective separation. The pore sizes for OX‐ or PX‐selective separation range between 3 and 21 Å, with pore size influencing performance. Pore size has had less influence on MX‐ or EB‐selective separation. π interactions and Van der Waals forces also play a role. Additionally, some molecular compounds with structural flexibility have shown exceptional performance, achieving record high selectivity. Eight studies reporting reticular sorbents with high selectivity are discussed below.

**TABLE 16:** C8 aromatic (EB, PX, MX, and OX) isomers binary or multicomponent separations. The following parameters are listed for comparison: pore size; BET surface area (S_BET_); single‐component gas uptakes; adsorption enthalpies (*Q*_st_); separation factor; analyzation method, regeneration temperature; and attributed mechanisms. Reticular sorbents are listed in decreasing order of binary or multicomponent separation factor for C8 isomers.

Adsorption preference	Adsorbent, network dimensionality (nD)	Pore size (Å)	Components	Separation factor^a^	C8 capacity (mmol/g)	Analyzation method	Regeneration temperature (K)	S_BET_ (m^2^/g)	Mechanism	Refs.
OX‐selective	**ZUL‐C3**, 3D (303 K)	4.5 × 5.2, 3.5 × 3.2 3.4 × 3.4, 3.6 × 4.2	OX/PX	21.8	PX: 0.17 MX: 0.29 OX: 0.68 EB: 0.47	NMR	423K with N_2_ flow	1200	C‐H···π interactions	[745]
			OX/MX	7.71						
			OX/EB	7.81						
	**CD‐MOF‐1**, 3D	7.8 × 7.8	OX/MX	6.73	—	HPLC	—	1220 [746]	Van der Waals interactions	[747]
			OX/PX	17.93						
			MX/PX	2.67						
	**CD‐MOF‐2**, 3D	7.8 × 7.8	OX/MX	4.76	—	HPLC	—	1030 [746]	Van der Waals interactions	[747]
			OX/PX	16.37						
			MX/PX	3.44						
	**CD‐MOF‐1**, 3D	21.4^b^	OX/MX	3.4	OX: 0.59 MX: 0.29 PX: 0.19	Breakthrough, GC	—	391	Pore size	[748]
			MX/PX	11.2						
			OX/PX	14.6						
	**MIL‐47**, 3D	10.5 × 11.0 [749]	PX/EB	9.7	—	Breakthrough; HPLC	Hexane flow at 298 K	930	π···π interaction	[750]
			PX/MX	2.9						
			OX/MX	2.0						
			OX/EB	10.9						
			OX/PX	1.4						
			MX/EB	4.2						
	**MIL‐53(Al)*ht***, 3D	8.5 × 8.5	PX/EB	3.1	—	Breakthrough, GC	Hexane flow at 298 K	940	Pore size, π···π interactions	[751]
			MX/PX	1.2						
			OX/MX	2.7						
			OX/EB	10.9						
			OX/PX	3.5						
			MX/EB	3.8						
	**CAU‐23**, 3D	6.3 × 7.6	OX/PX	10.4	OX: 1.90	GC	*n*‐Heptane	1243	H‐bonding	[752]
			OX/MX	7.6						
	**MIL‐53‐TDC**, 3D	8.5 × 8.5	OX/PX	3.2	OX: 1.29	Breakthrough, GC	*n*‐Heptane	1101	C‐H···π, π···π interactions	[752]
			OX/MX	5.4						
	**Co_2_(dobdc)**, 3D (306 K)	8.1 × 8.1	OX/MX	2.5	—	Breakthrough, GC	—	1410^c^	UMCs	[753]
			OX/PX	3.9						
			OX/EB	1.21						
			EB/MX	2.05						
			EB/PX	3.21						
			MX/PX	1.6						
	**MIL‐53(Fe)**, 3D (293 K)	6.8 × 21.3 (narrow phase) [754]	MX/PX	2.63	—	HPLC	—	23 [755]	π···π interaction, flexibility	[756]
			OX/MX	1.34						
			OX/PX	3.52						
	**MIL‐53(Al)**, 3D	8.5 × 8.5	PX/EB	3.3	—	HPLC	—	904	C‐H···O interactions	[757]
			MX/PX	1.52						
			OX/MX	1.93						
			PX/OX	1.5						
	**CPO‐27‐Ni**, 3D (398 K)	11 × 11	MX/PX	2	PX: 2.0 MX: 2.1 OX: 1.9	GC	—	1425	Pore shape, electrostatics	[758]
			OX/MX	1.7						
			OX/PX	3.3						
	**CPO‐27‐Ni**, 3D	11 × 11	OX/PX	3.3	PX: 0.86 OX: 2.93	Breakthrough, GC	—	1351 [759]	UMCs	[760]
	**Cu‐BTC@Fe_3_O_4_**, 3D (453 K)	9 × 9	OX/MX	3.3	OX: 3.46 MX: 1.04	Breakthrough, GC	—	562	Electrostatics	[761]
			OX/PX	2.7						
			MX/PX	1.1						
	**CMOMO‐7**, 3D (303 K)	6.6 × 8.1	OX/PX	3.1	OX: 2.3 MX: 2.1 PX: 1.9	Breakthrough	N_2_ flow at 423 K	819	C‐H···π, interactions	[762]
			MX/PX	2.5						
	**UiO‐66**, 3D (313 K)	8 × 8, 11 × 11	OX/MX	1.8	—	HPLC	—	885	Pore shape	[630]
			OX/PX	2.4						
	**SIFSIX‐1‐Cu**, 3D	8 × 8 [32]	MX/PX	2.3	OX: 1.23 MX: 1.05 PX: 0.74	Breakthrough, GC	—	1012	H‐bonding	[763]
			OX/PX	2.7						
			OX/MX	1.1						
	**COF1**, 3D	7.8 × 12.3	PX/EB	1.4	—	HPLC	—	666	Polarizability, H‐bonding	[764]
			MX/PX	1.3						
			OX/MX	2.0						
	**CAU‐13**, 3D	—	OX/PX	1.5	OX: 0.34 mol mol^−1^ PX: 0.29 mol mol^−1^ MX: 0.28 mol mol^−1^	GC	—	—	Flexibility	[765]
			OX/MX	1.9						
			PX/MX	1.3						
	**Zn(BDC)(dabco)_0.5_**, 3D(448 K)	7.5 × 7.5	MX/EB	1.15	—	Breakthrough, GC	—	—	Selective inclusion	[766]
			OX/EB	1.62						
			OX/MX	1.12						
			EB/PX	1.15						
			MX/PX	1.25						
			OX/PX	1.88						
	**MIL‐101(Cr)**, 3D	12, 16 × 14.5	PX/EB	1.64	—	HPLC	Hexane/DCM flow at 298 K	4000 [767]	π···π interaction, UMCs	[768]
			MX/PX	1.44						
			OX/MX	1.86						
			OX/PX	1.17						
	**RHO ZMOF**, 3D	18 × 18, 9 × 9	OX/PX	1.3	PX: 0.63 OX: 0.92	Breakthrough, GC	—	871 [769]	UMCs	[760]
	**HKUST‐1**, 3D	6.5 × 6.5, 10, 12, 4.6 × 4.6	OX/PX	1.3	PX: 1.20 OX: 1.63	Breakthrough, GC	—	1055 [603]	UMCs	[760]
	**HKUST‐1**, 3D (398 K)	6.5 × 6.5	MX/PX	1.1	PX: 2.8 MX: 2.4 OX: 2.8	Breakthrough, GC	—	1718	Electrostatics	[758]
			OX/MX	1.1						
			OX/PX	1.2						
	**ZIF‐76**, 3D	11.6 × 11.6, 5.4 × 5.4, 18 × 18	OX/PX	1.1	PX: 0.18 OX: 0.26	Breakthrough, GC	—	827 [770]	Polarizability	[760]
	hybrid [3]arene **H** (**Hα**), 0D	—	OX/MX	97.5%	—	NMR	—	—	C‐H···π and C‐H···O interactions	[771]
			OX/PX	97.61%						
	**MIL‐47(V)**, 3D	10.5 × 10.5	—	—	OX selective	—	—	—	π···π interactions	[772]
	**Tz‐DHTA**, 2D	25.9^b^	—	—	OX selective	GC	—	822	Pore‐filling	[773]
	**Tph‐DHTA**, 2D	17.8^b^	—	—	OX selective	GC	—	528	Pore‐filling	[773]
	**Tab‐DHTA**, 2D	20^b^	—	—	OX selective	GC	—	1137	Pore‐filling	[773]
	**TpTFMB**, 2D	15.5	OX/PX/MX/EB	—	OX selective	GC	—	964	H‐bonding	[774]
	**TpBD**, 2D	17.2	OX/PX/MX/EB	—	—	504	—	[774]
	**TpPa‐CF_3_**, 2D	14.5	OX/PX/MX/EB	—	OX selective			1306	Polarizability, pore size	[774]
	**TbTFMB**, 2D	17.8			—			500	—	[774]
	**MOF‐1**, 3D (448 K)	4, 7.5	OX/(MX+PX+EB)	—	OX selective	Vapor sorption isotherms	—	2342.5	Pore size, shape	[775]
			EB/(OX+MX+PX)							
	**MIL‐47**, 3D (448 K)	10.5 × 11.0	OX/(MX+PX+EB)	—	OX selective	Vapor sorption isotherms	—	1837.8	Pore size, shape	[775]
			EB/(OX+MX+PX)							
	**IRMOF‐1**, 3D (448 K)	15, 11	OX/(MX+PX+EB)	—	OX selective	Vapor sorption isotherms	—	3821.5	Pore size, shape	[775]
			EB/(OX+MX+PX)							
	**BTAPa‐CF_3_**, 2D	6.3 × 6.3, 14.5 × 14.5	OX/MX/PX	—	OX selective	GC	—	953	Pore size	[655]
PX‐selective	**MAF‐89**, 3D	4.2 × 4.2, 5.5 × 5.5	PX/MX	19.4	PX: 2.00	Breakthrough, GC	—	562	Kinetics, van der Waals, electrostatics	[776]
			PX/OX	221						
	**HIAM‐203**, 3D (393 K)	6^b^	PX/OX	211.2^d^	PX: 1.4 MX: 0.07 OX: 0.11	Breakthrough, GC	—	480	Kinetics, pore size, flexibility	[777]
			PX/MX	171.9^d^						
	**TPBDB**, 0D	5.4 × 9.1	PX/MX	50.6 (22.1)	—	GC, NMR	423 K	—	H‐bonding	[778]
			PX/OX	54.1 (76.1)						
			PX/EB	47.3 (49.2)						
			PX/OX/MX	60.4 (56.3)						
			PX/OX/MX/EB	85.7 (37.5)						
	**β‐ [Cu_2_(pypz)_2_]**, 3D	—	PX/MX/OX	51	—	GC	DCM (423 K)	—	Flexibility	[779]
	**sql‐1‐Co‐NCS**, 2D	4.003, 4.172, 4.397^e^	OX/MX	5.3	OX: 87.0 MX: 87.0 PX: 84.8 EB: 43.5	NMR	313 K under vacuum	—	C‐H···π interactions	[780]
			OX/PX	3.7						
			PX/MX	1.5						
			MX/EB	2.8						
			PX/EB	28.9						
	**H/ZSM5**, 3D (403‐443 K, 6–8 bar)	5.95^b^	PX/MX	25.0	OX: 0.28 MX: 0.12 PX: 1.34 EB: 0.57	Breakthrough, GC	PDEB (403‐443 K)	—	Van der Waals interactions (Lewis acid, basic binding sites)	[781]
			PX/OX	16.8						
			PX/EB	6.8						
	**NU‐2000**, 3D	7.0 × 7.0	PX/OX	20	PX: 1.14 MX: 0.29 OX: 0.06	NMR	—	570	Molecular sieving	[782]
			PX/MX	3.9						
	**sql‐4,5‐Zn**, 2D	—	PX/OX	13.69	PX: 20.7%^f^ EB: 15.9%^g^	Breakthrough, GC	—	—	C–H···π, π···π interactions	[783]
			EB/OX	16.92						
			MX/OX	4.23						
	**α‐ [Cu_2_(pypz)_2_]**, 3D	7.0 × 6.2	PX/MX/OX	16	—	GC	DCM (423 K)	—	Flexibility	[779]
	**ZSM‐5(SiO_2_/Al_2_O_3_ = 600)**, 3D	—	PX/EB	5.52	EB: 0.21 PX: 1.2	GC	—	—	Pore shape	[784]
			PX/OX	12.42						
			OX/EB	2.25						
	**MIL‐120(Al)**, 3D	5.4 × 4.7	PX/OX	11.5	PX: 0.5	GC	—	322	Pore shape, size	[785]
			PX/MX	8.3						
			PX/EB	3.6						
	**MAF‐88**, 3D	5.3 × 5.4, 6.4 × 9.6	PX/MX	6.7	PX: 2.00	Breakthrough, GC	—	—	—	[776]
			PX/OX	10.7						
	**Cu(CDC)**, 3D	5.4 × 5.4	PX/OX	10.0	PX: 1.1	GC	Methanol (423 K)	348	Pore shape	[786]
			PX/MX	7.0						
			PX/EB	5.0						
	**Li/ZSM5**, 3D (403‐443 K, 6–8 bar)	5.71^b^	PX/MX	8.2	—	Breakthrough, GC	PDEB (403‐443 K)	—	Van der Waals interactions (Lewis acid, basic binding sites)	[781]
			PX/OX	6.0						
			PX/EB	4.0						
	**DUT‐8(Cu)**, 3D	9.6 × 9.6	PX/OX	5.4	PX: 1.8	GC	—	2694	Pore size, π···alkyl interactions	[787]
			PX/MX	7.2						
			PX/EB	5.9						
	**HOF‐PX‐a**, 3D	—	PX/OX	7.2	—	NMR	—	—	C‐H···π, π···π interactions	[788]
			PX/MX	6.1						
			PX/EB	4.1						
	**UIO‐66(Zr)**, 3D	4‐5	OX/MX	7.1	—	Breakthrough	—	—	π···π interactions	[789]
			OX/PX	2.3	—	Breakthrough	—	—	π···π interactions	[789]
			PX/MX	3.1	—	Breakthrough	—	—	π···π interactions	[789]
	**Na/ZSM5**, 3D (403‐443 K, 6–8 bar)	5.56^b^	PX/MX	6.7	—	Breakthrough, GC	PDEB (403‐443 K)	—	Van der Waals interactions (Lewis acid, basic binding sites)	[781]
			PX/OX	5.5						
			PX/EB	2.0						
	**Ce(HTCPB)**, 3D (383 K)	2.25 × 2.54, 2.04 × 2.33	PX/MX	4.5	—	GC	—	378.95	Flexibility, C–H···π interactions, H‐bonding	[790]
			PX/OX	5.6						
			PX/EB	2.4						
	**nano‐zeolite K‐X**, 3D (423 K)	7.1^b^	PX/MX	5.36	—	Breakthrough, GC	Toluene at 423 K	688	Van der Waals interactions (Lewis acid, basic binding sites)	[791]
			PX/OX	2.43						
			PX/EB	3.22						
	**MAF‐X8**, 3D (433 K)	8.5 × 8.5	PX over all three	5.3	PX: 2.2	—	—	1465	Pore shape	[792]
	**K/ZSM5**, 3D (403‐443 K, 6–8 bar)	5.34^b^	PX/MX	5.1	—	Breakthrough, GC	PDEB (403‐443 K)	—	Van der Waals interaction (Lewis acid, basic binding sites)	[781]
			PX/OX	3.9						
			PX/EB	1.7						
	**MIL‐125(Ti)**, 3D	6.13 × 6.13, 12.55 × 12.55 [632]	PX/MX	4.4	—	HPLC	—	1446	Pore shape	[793]
	**BaY zeolite**, 3D (353 K)	7.0^b^	PX/OX	4.0	—	HPLC	n‐octane (353 K)	313	Molecular sieving	[794]
			PX/MX	3.9						
			PX/EB	1.8						
	**nano‐zeolite Na‐X**, 3D (423 K)	7.2^b^	PX/MX	3.73	—	Breakthrough, GC	Toluene at 423 K	697	Van der waals interactions (Lewis acid, basic binding sites)	[791]
			PX/OX	1.68						
			PX/EB	2.78						
	**CAU‐1(Al)‐NH_2_**, 3D	10 × 10 [795]	PX/MX	3.5	—	HPLC	—	1495	Pore shape	[793]
	**ZIF‐8**, 3D	11.6 × 11.6, 3.4 × 3.4	PX/OX	3.3	PX: 0.47 OX: 0.15	GC	—	739 [796]	Molecular sieving	[760]
	**MIL‐125(Ti)‐NH_2_**, 3D	12.5 × 12.5, 6.1 × 6.1	PX/MX	2.8	—	HPLC	—	1380	Pore shape	[793]
	**nano‐zeolite Li‐X**, 3D (423 K)	7.2^b^	PX/MX	2.58	—	Breakthrough, GC	Toluene at 423 K	699	Van der Waals interactions (Lewis acid, basic binding sites)	[791]
			PX/OX	1.36						
			PX/EB	1.32						
	**MIL‐140B**, 3D (323 K)	3.8 × 3.8 [667]	PX/EB	2.1^d^	—	Breakthrough, GC	323	460	Pore shape	[797]
			PX/MX	1.7^d^						
			PX/OX	1.8^d^						
	**CAU‐1‐OH**, 3D	6.0, 8.0^b^	PX/MX	1.86	PX: 4.33	Breakthrough, GC	—	1366	C–H···π interactions	[798]
	**MOF‐48**, 3D (323 K)	5.2 × 5.2 [799]	PX/EB	1.5^d^	—	Breakthrough, GC	323	195	Pore shape	[797]
			PX/MX	1.7^d^						
			PX/OX	1.7^d^						
	**nano‐zeolite H‐X**, 3D (423 K)	7.2^b^	PX/MX	1.22	—	Breakthrough, GC	Toluene at 423 K	712	Van der waals interactions (Lewis acid, basic binding sites)	[791]
			PX/OX	1.20						
			PX/EB	1.19						
	**EtP6**, 0D	6.7 × 6.7	PX/OX/MX	99.1%	—	NMR	433 K	—	π···π interactions	[800]
	**P [4]Q [1]L**, 0D	—	PX/(MX+OX+EB)	97.5%	—	NMR	—	—	C‐H···π and C‐H···O interactions	[801]
	**phenanthrene [2]arene**, 0D	—	PX/MX	81%	—	GC, NMR	423 K	—	C‐H···π and C‐H···O interactions	[802]
	**TEMPO@ZIF‐8**, 3D	7.26 × 7.26	PX/MX	93%	—	NMR	—	—	Polarizability	[803]
			PX/OX	95%						
			MX/OX	92%						
	**[Zn(Hpidba)]*_n_***, 3D	8.2‐8.7, 5.6 × 6.8	PX/MX/OX	—	PX selective	GC	—	1319	C‐H···O, π···π interactions	[804]
	**JNU‐5**, 2D	13	—	—	PX selective	GC	—	419	C‐H···π, π···π interactions	[805]
	**SCOF‐303**, 2D	8.31	—	—	PX selective	GC	—	490	π···π interactions	[806]
	**DynaMOF‐100 ([Zn_4_O(L)_3_(DMF)_2_])**, 3D	5.1^b^	—	—	PX: 2.86 MX: 0.22 OX: 0.05	GC	—	—	Pore size	[807, 808]
	**Co_2_L_2_(AzoD)_2_**, 3D	3.3 × 10.6	—	—	PX: 2.5 MX: 0 OX: 0.95	—	—	113	Pore size	[809]
	**JUC‐77**, 3D	10.8 × 7.3	—	—	PX: 3.3	—	—	976	Molecular sieving	[810]
	**[Zn(μ_4_‐L)]*_n_***, 3D	14.8 × 18.7	—	—	PX:0.02 MX: 0.01	GC, NMR	CH_3_Cl	—	Pore size, shape	[811]
	**Co‐CUK‐1**, 3D	6.8 × 6.8	—	—	PX: 2.1^f^ MX: 0^f^ OX: 0^f^ EB: 0.2^f^	—	—	730	Electrostatics, pore shape	[812]
MX‐selective	**sql‐1,3‐Co‐NCS**, 2D	7.5 × 11.5	PX/EB	9.8	OX: 37% MX: 37% PX: 37% EB: 18.7%	NMR	—	—	C–H···π, π···π stacking interactions	[813]
			MX/EB	10.8						
			OX/EB	7.9						
	**MIL‐160**, 3D	9.05 × 9.05	MX/PX	4.0	MX: 1.3	Breakthrough	—	1243	Electrostatics, π···π stacking interactions	[814]
			MX/OX	2.8						
			OX/PX	1.4						
	**MFM‐300(Fe)**, 3D	6.6 × 6.8	MX/PX	3.9^d^	—	Breakthrough, GC	—	924	Pore size, π‐π interactions, π‐O interactions	[815]
			MX/OX	1.6^d^						
			OX/PX	2.5^d^						
	**MFM‐300(V)**, 3D	6.8 × 7.0	MX/PX	3.7^d^	—	Breakthrough, GC	—	1091	Pore size, π‐π interactions, π···O interactions	[815]
			MX/OX	1.1^d^
			OX/PX	3.0^d^						
	**MFM‐300(In)**, 3D	7.2 × 7.4	MX/PX	3.5^d^	—	Breakthrough, GC	—	903	Pore size, π‐π interactions, π‐O interactions	[815]
			MX/OX	2.3^d^						
			OX/PX	1.3^d^						
	**NIIC‐30(Ph)**, 3D	2.9 × 9.6, 4 × 9.2	MX/OX	3.03 (1.94^h^)	—	NMR	—	451	C–H···O, C–H···π interactions	[816]
			PX/OX	2.97 (2.12^g^)						
			PX/MX	1.05 (1.19^g^)						
	**ZU‐61**, 3D (333 K)	7.8^b^	MX/PX	2.9	PX: 0.62 MX: 1.15 OX: 1.61	Breakthrough, GC	398K	1384	Pore shape, C–H···F interactions	[817]
	**Zn_2_(aip)_2_(bpe)**, 3D	14.7	MX/OX/EB	—	MX selective	NMR	—	125	π···π interactions	[818]
EB‐selective	**Zn‐ETTOB**, 3D	7 × 7	PX/EB	1.2	EB: 8.08 PX: 8.00 MX: 7.54 OX: 6.51	—	—	2594.9	Van der Waals interactions	[819]
			PX/MX	3.9						
			PX/OX	6.2						
			EB/MX	3.3						
			EB/OX	13.1						
			MX/OX	3.5						
	**MOF‐monoclinic**, 3D (393 K, 80kPa)	7 × 7	MX/PX	2.52	—	Breakthrough, GC	—	225	Polarizability	[820]
			EB/PX	5.17						
			OX/PX	4.55						
	**MOF‐5**, 3D (523 K, 120 kPa)	12 × 12	EB/OX	1.96	—	Breakthrough, GC	—	773	Polarizability	[820]
			EB/MX	2.34						
			EB/PX	4.14						
	**MIL‐47**, 3D (343 K)	10.5 × 11.0 [749]	EB/PX	1.83	—	Breakthrough, GC	—	—	Polarizability, π⋅⋅⋅π interactions	[821]
			EB/MX	1.41						
			EB/OX	1.39						
			MX/PX	2.07						
			OX/MX	1.17						
			OX/PX	1.01						
	**Zn_2_(aip)_2_(bpy)**, 3D	15.2	EB/MX/OX	—	EB selective	NMR	—	332	π···π interactions	[818]

^a^ Selectivity calculated from the uptake from mixtures of binary (v/v = 1/1) C8 aromatic compounds.

^b^ Pore size distribution, BET surface area calculated from the N_2_ adsorption isotherm at 77 K.

^c^ Langmuir surface area calculated from the N_2_ adsorption isotherm at 77 K.

^d^ Selectivities calculated from binary breakthrough curves.

^e^ Distances between adjacent layers.

^f^ IAST calculated uptakes for gas phases.

^g^ Gravimetric Uptake.

^h^ Vapor selectivity.

In 1989, T. Y. Yan reported the separation of PX and EB from C8 aromatics using the zeolite **ZSM‐5** (SiO_2_/Al_2_O_3_) [784]. The effective separation of PX/EB, with an uptake ratio of 5.5, was attributed to the optimal pore size and commensurate packing of PX in the cavities. In 2007, Alaerts et al. reported the first separation of xylene isomers and EB using MOFs (**MIL‐47**, VO(BDC)·(H_2_BDC)_0.75_ (BDC = 1,4‐benzenedicarboxylate)) [750]. As demonstrated by breakthrough and chromatographic experiments, **MIL‐47** exhibited high selectivity for PX/EB, OX/EB, and MX/EB, with selectivity values of 9.7, 10.9, and 4.2, respectively. The difference in π···π interactions between the C8 aromatics and the **MIL‐47** framework was determined to be key to the separation performance.

In 2016, Nair, Sholl, and co‐workers selected a series of MOFs for C8 aromatics separation using computational methods, and their performance was subsequently confirmed through experimental evaluation. Based on the results of the computational screening, four top‐performing MOF materials were identified: **MIL‐47** [VO(BDC)·(H_2_BDC)_0_._75_ (BDC = 1,4‐benzenedicarboxylate)]; **MIL‐125‐NH_2_** [Ti_8_O_8_(OH)_4_(BDC‐NH_2_)_6_]; **MIL‐140B** [ZrO(NDC)]*_n_* (NDC = 2,6‐naphthalenedicarboxylate); and **MOF‐48** [VO(DMBDC)·(H_2_DMBDC)_0_._4_ (DMBDC = 2,5‐dimethylbenzenedicarboxylate)]*_n_* [797]. At 323 K, the selectivity of **MOF‐48**, as determined from breakthrough data, was PX/EB: 1.5, PX/MX: 1.7, and PX/OX: 1.7, respectively. Computational analysis of free energy and structural characteristics revealed that PX has the lowest free energy along the corrugated channels of **MOF‐48**. This work demonstrates an approach for identifying PX‐selective reticular sorbents from the MOF database using computational methods.

**FIGURE 24:**
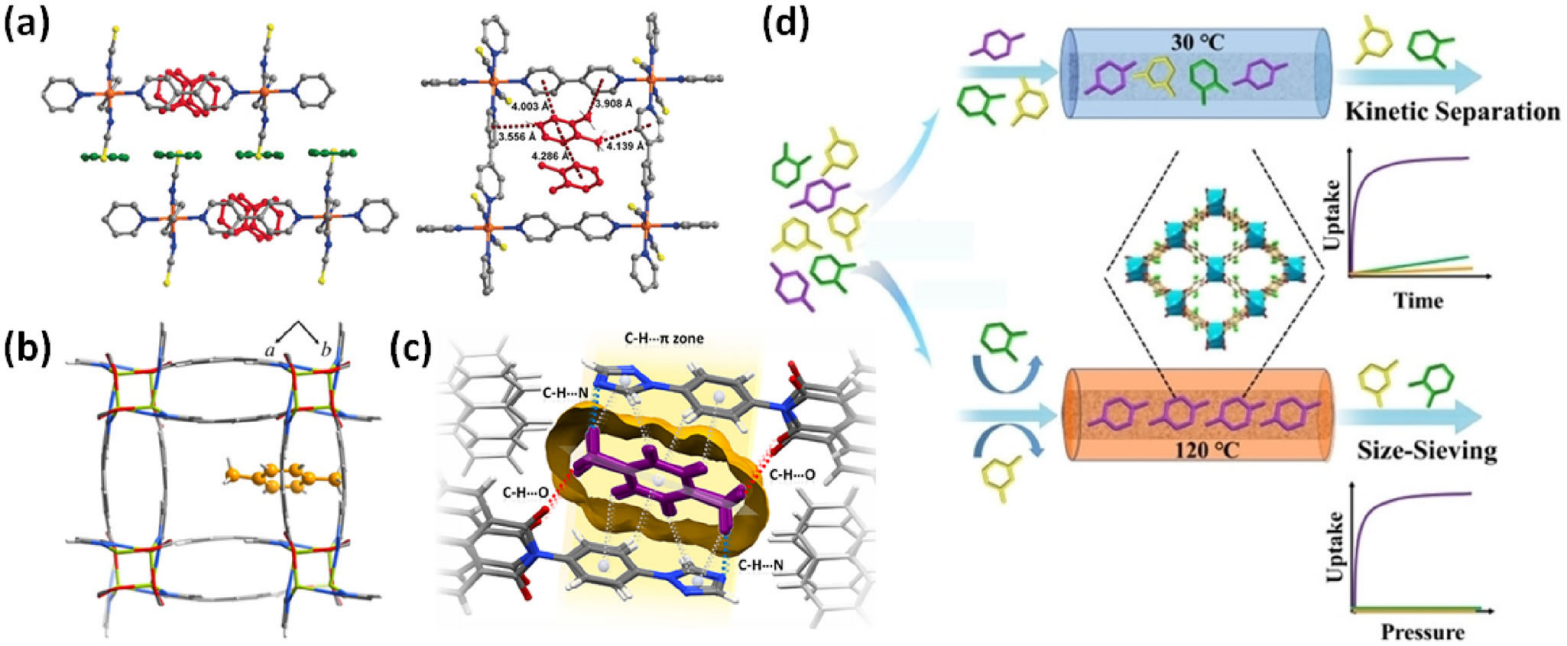
Examples of top‐performing sorbents for C8 aromatics separation with high selectivity: (a) **sql‐1‐Co‐NCS** [780], (b) **MAF‐89** [776], (c) **TPBD** [778], and (d) **HIAM‐203** [777] with feed ratio and temperature set at 1:1and 298 K, respectively. (Reprinted with permission from ref. [780]: Copyright 2019, Wiley‐VCH GmbH; ref. [776]: Copyright 2022, Science China Press and Springer‐Verlag GmbH Germany; ref. [778]: Copyright 2023, the Authors; ref. [777]: Copyright 2023, Wiley‐VCH GmbH.).

In 2019, Zaworotko and co‐workers reported a flexible **sql** topology MOF that is effective for C8 aromatics separation [780]. The non‐porous layered material [Co(bpy)_2_(NCS)_2_]_n_ (**sql‐1‐Co‐NCS**) was found to reversibly switch to large pore phases to accommodate C8 aromatics, with varying switching pressures and kinetics. High OX selectivity (S_OX/EB_ ≈ 60) and saturation capacity (>80 wt%) were observed. SCXRD analysis of xylene‐included single crystals revealed additional C─H···π interactions between the phenyl hydrogens of OX and **sql‐1‐Co‐NCS**, compared to those seen for the other isomers. These supplementary C─H···π interactions enabled the high OX‐selectivity over PX, MX, and EB and high OX uptake capacity (Figure 24a). Also in 2019, J. P. Zhang and co‐workers reported another example of a flexible CN for C8 aromatics separation, the metal‐azolate framework, **MAF‐36**. The open phase of **MAF‐36**, (**[Cu_2_(pypz)_2_]·0.5p‐xylene**, Hpypz = 4‐(1*H*‐pyrazol‐4‐yl)pyridine) features one‐dimensional channels and discrete small cavities, while a non‐porous phase was obtained upon removal of the guest molecules [779]. This guest‐induced structural flexibility plays a crucial role in C8 aromatics separation. The open phase, **α‐ [Cu_2_(pypz)_2_]**, with a pore size of 7.0 × 6.2 Å^2^, exhibited a PX/MX/OX uptake ratio of 16, while the closed phase, **β‐ [Cu_2_(pypz)_2_]**, exhibited a higher PX/MX/OX uptake ratio of 51.

In 2022, J. P. Zhang and C. T. He reported on the C8 sorption properties of two isostructural MOFs ***α*‐ [Zn(pba)]** (**MAF‐88**, H_2_pba = 4‐(1*H*‐pyrazol‐4‐yl) benzoic acid) and ***β*‐ [Zn(pba)]** (**MAF‐89**) [776]. **MAF‐88** and **MAF‐89** were found to possess similar pillared‐column structures, porosities, and high PX capacities of 2.0 mmol/g. However, due to differences in pore size and pore topology, their PX selectivities were quite different. For PX/OX and PX/MX separation, **MAF‐88**, with its narrow 1D channels (5.3 × 5.4 and 6.4 × 9.6 Å^2^), exhibited selectivities of 10.7 and 6.7, respectively. In contrast, **MAF‐89**, featuring 3D‐connected quasi‐discrete pores (4.2 × 4.2 and 5.5 × 5.5 Å^2^), exhibited selectivities of 221 and 19.4, respectively (Figure 24b). MD simulations indicated that the mechanism involved diffusion‐controlled kinetic separation. The movement trajectories showed guest molecules trapped in the 0D pores. These guest xylene molecules were found to occasionally jump to neighbouring pores through the connecting apertures. Consequently, selectivity was governed by kinetics, and attributed to the gating actions of the flexible narrow apertures.

In 2023, H. Wang and co‐workers reported another example of a flexible MOF with a temperature‐driven kinetic separation mechanisms [777]. **HIAM‐203** (Ca(chloranilate)), was found to separate PX from OX and MX isomers driven by its flexible structure. At 303 K, all three isomers are accommodated, but the adsorption kinetics of OX and MX were found to be significantly slower than that of PX (Figure 24d). At the elevated temperature of 393 K, OX and MX were excluded while PX was adsorbed.

In addition to using porous materials, molecular compounds can also be applied for C8 aromatics separation. In 2018, K. Jie et al. reported a flexible molecular compound, **EtP6** (perethylated pillar [6]arene), which separated PX with a purity of 99.1% [800]. Selectivity was found to be an intrinsic property of the **EtP6** host, resulting from its size and shape as well as flexible cavities that adapt during adsorption to trap PX in the solid state.

In 2023, Zaworotko and co‐workers reported another flexible molecular compound (**TPBD**, 4‐(1*H*‐1,2,4‐triazol‐1‐yl)‐phenyl‐1*H*‐benzo [de]isoquinoline‐1,3(2*H*)‐dione) that exhibited record‐high PX‐selective separation from PX/OX/MX/EB mixtures [778]. Binary selectivity values for PX, as determined by ^1^H NMR spectroscopy and gas chromatography, ranged from 22.4 to 108.4, setting new benchmarks for PX/MX (70.3) and PX/EB (59.9) selectivity, and achieving near‐benchmark selectivity for PX/OX (108.4) (feed ratio = 1:1, temperature: 298 K). Analysis of the C8 aromatics‐induced flexibility and PX‐loaded structure of **TPBD** revealed that it formed channels that are a particularly good shape match for PX. Multiple noncovalent interactions (C─H···π, C‐H···N, C─H···O) were observed between PX and **TPBD**. **TPBD** was the first sorbent of any class to demonstrate high across‐the‐board PX selectivity from quaternary mixtures of C8 aromatics under ambient conditions (Figure 24c).

**FIGURE 25:**
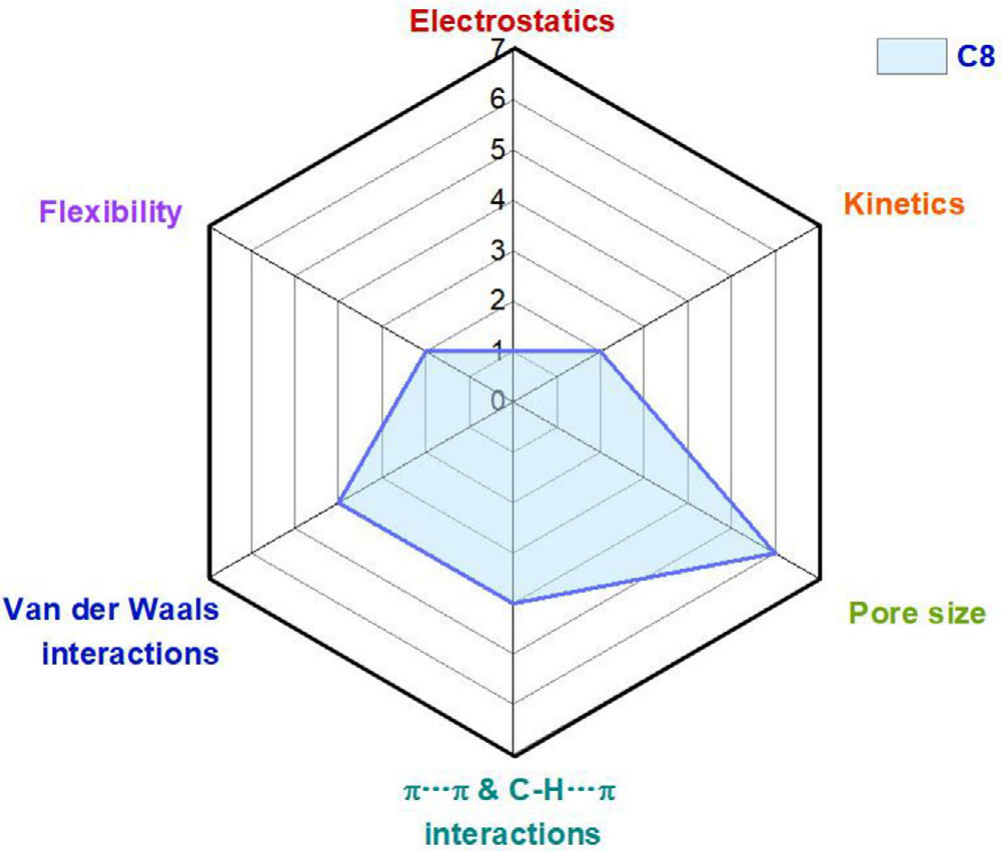
Radar plot presenting the contribution of six separation mechanisms (and their combinations) among the 15 top‐performing sorbents that exhibit binary or multiple C8 HCs adsorption selectivities. Each concentric hexagon denotes one sorbent each, while the central hexagon represents one.

For the separation of C8 HCs, six mechanisms were identified among the 15 top‐performing sorbents. The mechanisms include electrostatics, kinetics, pore size, π···π and C─H···π interactions, van der Waals interactions, and flexibility (Figure 25). Pore size was found to be the leading driver for C8 separation enabled by the smaller molecular sizes of PX and OX (Table 2). OX and PX selective sorbents are the most commonly reported, thanks to size exclusion. van der Waals and π···π and C─H···π interactions are the second most reported mechanism to drive selectivity.

## 5 Conclusions and future directions

### 5.1 We Have Come an Awfully Long Way

**Properties**: This article details the emergence over the past two decades of reticular crystalline materials as physisorbents, exemplified by MOFs and COFs, for separating and/or purifying industrially relevant HCs, including separations involving CO_2_. Overall, exceptional enhancements in performance have been reported. Whereas trace separations (removal of minor impurities at levels <1%) were generally infeasible for physisorbents as recently as 2010, as detailed herein, this is no longer the case. Today, benchmark selectivity values for several industrially relevant separations go far beyond those exhibited by traditional adsorbents like zeolites and silica. These advances are summarized in Table 17 as exemplified by seven adsorbate pairs and three entries: the existing benchmark material; the sorbent that represented a significant leap forward; the sorbent that is the current state‐of‐the‐art.

**TABLE 17:** Summary of binary selectivities and binding mechanisms (towards the more preferred sorbate) for a select few reticular sorbents, as listed below.

Adsorbent	Year	Selectivity^a^	Mechanism	Refs.
Adsorbate pair (CO_2_/N_2_)				
Zeolite 13X	2002	420^b^/562^c^	Cation···π interactions	[822]
SIFSIX‐3‐Zn	2013	1818^†^	Electrostatics (F‐CO_2_); pore size	[823]
NbOFFIVE‐1‐Ni	2016	601^b^/6528^c^	Electrostatics (F‐CO_2_); pore size	[824]
Adsorbate pair (CO_2_/CH_4_)				
Zeolite 13X	2004	790	Electrostatics	[825]
TIFSIX‐3‐Ni	2019	3501	Electrostatics (F‐CO_2_); pore size	[134]
Cu‐F‐pymo	2022	10^7^ ^c^	Molecular sieving	[128]
Adsorbate pair (C_2_H_2_/C_2_H_4_)				
Fe‐MOF‐74	2012	2.1	UMCs	[66]
SIFSIX‐2‐Cu‐i	2016	44.5	Electrostatics (C‐H∙∙∙F); pore size	[35]
UTSA‐300a	2017	10^4^ ^c^	Flexibility; H‐bonding; pore size	[159]
Adsorbate pair (C_2_H_4_/C_2_H_6_)				
Zeolite 5A	2013	4.5	Cation···π interactions	[307]
Co‐gallate	2018	52	Molecular sieving	[291]
UTSA‐280	2018	10^4^ ^c^	Molecular sieving; H‐bonding; π···π stacking; van der Waals interactions	[287]
Adsorbate pair (C_2_H_6_/C_2_H_4_)				
ZIF‐7	2010	1.6	Flexibility	[387]
MAF‐49	2015	9	H‐bonding; electrostatics	[314]
NIIC‐20‐Bu	2020	15.4	H‐bonding; C‐H···π interactions	[313]
Adsorbate pair (C_3_H_6_/C_3_H_8_)				
Co‐MOF‐74	2012	45	UMCs	[472]
UTSA‐400	2023	10^7^ ^c^	H‐bonding; van der Waals interactions	[456]
HAF‐1	2025	1.67 × 10^7^ ^c^	Molecular sieving; H‐bonding; C‐H···π interactions	[455]
Adsorbate pair (PX/OX)				
nano‐zeolite K‐X	2015	2.43	Van der Waals interactions	[791]
HIAM‐203	2023	211.2	Kinetics; pore size; flexibility	[777]
MAF‐89	2022	221	Kinetics; van der Waals interactions; electrostatics	[776]

^a^ IAST selectivity at 298 K at 1 bar.

^b^ For N_2_ mole fraction of 0.9 (CO_2_/N_2_ = 1/9).

^c^ For N_2_ mole fraction of 0.9995 (CO_2_/N_2_ = 500 ppm/0.9995 bar).

As revealed by Table 17, in some cases performance enhancements are driven by selective binding but in others molecular sieving. Highlights include the prototypal HUM, **SIFSIX‐3‐Zn**, which was the first physisorbent to offer ultrahigh CO_2_/N_2_ selectivity (∼2500:1), roughly an order of magnitude beyond the prior benchmarks zeolite 13X and **Mg‐MOF‐74** [33]. Second generation variants established that narrow pores with strongly electrostatic binding sites can capture CO_2_ at partial pressures relevant to direct air capture of CO_2_. The rigid CN **UTSA‐280** exhibited equimolar C_2_H_4_/C_2_H_6_ adsorption selectivity exceeding 4,100, producing >99% pure ethylene in the effluent stream [287]. Flexible HUMs can produce polymer‐grade propylene from propyne, with **TIFSIX‐14‐Cu‐i**, adsorbing C_3_H_4_ at a very low threshold pressure (∼500 ppm) and with an IAST selectivity of ∼355 for a 1/99 C_3_H_4_/C_3_H_6_ mixture [432].

Reticular sorbents can also capture trace gas and vapor components from more complex mixtures, including benzene removal from moist streams. Benzene is a toxic VOC and is often encountered as a minor impurity (ppm‐level) in petrochemical processes (e.g., in cyclohexane) or in air. The double‐walled CN **BUT‐55** was found to adsorb benzene at partial pressures below 10 Pa (∼100 ppm) even under humid conditions with effluent benzene levels below acceptable exposure limits. These examples underscore the extraordinary progress that has been made in HC separations with selectivity values and working capacities that could render energy‐intensive techniques (cryogenic distillation, solvent scrubbing, etc.) obsolete.

**Insight**: There is now much more understanding about the underlying structure‐function relationships that drive these strong separation performances. This insight comes in part from in situ experiments that have provided experimental observation of sorbent‐sorbate binding and also from advances in molecular modelling. Further, the inherent modularity of PCNs and COFs enables crystal engineering studies to systematically study families of related reticular sorbents to control structure/properties and further enhance performance. Several recent in situ studies have demonstrated how XRD and IR, typically coupled with computational experiments (DFT and/or GCMC), can reveal the fine details of those sorbent binding sites that enable high selectivity for a specific sorbate. For example, single‐crystal X‐ray diffraction studies have pinpointed the C_2_H_2_ binding site in the Cu(II) CN, **CPL‐1**. In **CPL‐1**, each adsorbed C_2_H_2_ molecule forms an H‐bond with a non‐coordinated framework O atom [60]. The acidic C–H on C_2_H_2_ can engage these O‐donors whereas CO_2_ (which lacks an H donor) cannot, thereby explaining **CPL‐1**’s strong C_2_H_2_/CO_2_ and C_2_H_2_/C_2_H_4_ selectivity. Similarly, Al(HCOO)_3_ (**ALF**) achieves excellent CO_2_ selectivity over C_2_H_2_ (≈6.5 × 10^5^) via directed C─H⋯O bonding [266]. In **ALF**’s ∼4.1×5.3 Å^2^ channels, in situ FTIR of CO_2_‐loaded **ALF** revealed that CO_2_ molecules bind at the electropositive C–H moiety of the formate linkers. This specific C─H⋯O interaction (along with the tight pore size) is key to the aforesaid ultrahigh CO_2_/C_2_H_2_ IAST selectivity. These examples illustrate that precise pore dimensions together with localized polar binding motifs (H‐bond donors or charged anions) can enable single sorbate discrimination.

Systematic crystal engineering studies can also reveal how even subtle composition changes, such as adding a fluorine atom to a linker and/or metal substitution, including doping, can profoundly impact sorbent performance. This is exemplified by the dramatic changes in the 195 K CO_2_ sorption profiles between [Co(bimpy)(bdc)]*_n_* (**X‐dia‐4‐Co**) and [Co(bimbz)(bdc)]*_n_* (**X‐dia‐5‐Co**) (H_2_bdc = 1,4‐benzendicarboxylic acid; bimpy = 2,5‐bis(1*H*‐imidazole‐1‐yl)pyridine; bimbz = 1,4‐bis(1*H*‐imidazole‐1‐yl)benzene) [826]. Specifically, **X‐dia‐4‐Co** showed a gradual phase transformation accompanied by a steady increase in CO_2_ uptake, whereas **X‐dia‐5‐Co** displayed a sharp switching isotherm, with the phase transition occurring at a relative pressure (*P/P*_0_) of 0.008 at 195 K, and at an absolute pressure of 3 bar at 298 K [826]. In another recent study, a Zn(II) CN, ZnFPCP, with fluorinated aromatic nanotraps was shown via DFTB/GCMC and operando FTIR to preferentially bind alkanes, yielding high C_3_H_8_/C_3_H_6_ and C_2_H_6_/C_2_H_4_ selectivities to produce ultra‐high purity (99.99%) C_3_H_6_ and C_2_H_4_. Likewise, pore tuning in **Fe‐MOF‐74** via pore‐space partitioning sharply improved C_2_H_6_/C_2_H_4_ selectivity [72]. A flexible Ni‐based diamondoid net (**X‐dia‐1‐Ni**) [54], was partially doped with Co to enhance its *inverse* C_2_H_6_/C_2_H_4_ IAST selectivity from 3.5 to 5.5 while maintaining high C_2_H_6_ uptake [316]. Even more extreme changes in properties can occur in interpenetrated vs. non‐interpenetrated variants of a CN, e.g., **SIFSIX‐2‐Cu** vs. **SIFSIX‐2‐Cu‐i**, where interpenetration means tight C_2_H_2_ binding sites with greatly enhanced performance for trace C_2_H_2_/C_2_H_4_ separation [35].

These examples span rigid ultramicroporous PCNs to soft flexible sorbents, and underscore not just the feasibility of fine‐tuning of composition, but how even small changes, typically guided by in situ insight and modelling, can significantly enhance performance. In short, there has not just been remarkable progress in terms of design (crystal engineering) and properties of reticular sorbents for HC separation, there is now insight into why these benchmark sorbents exhibit their performance.

### 5.2 How Did We Get Here?

**FIGURE 26:**
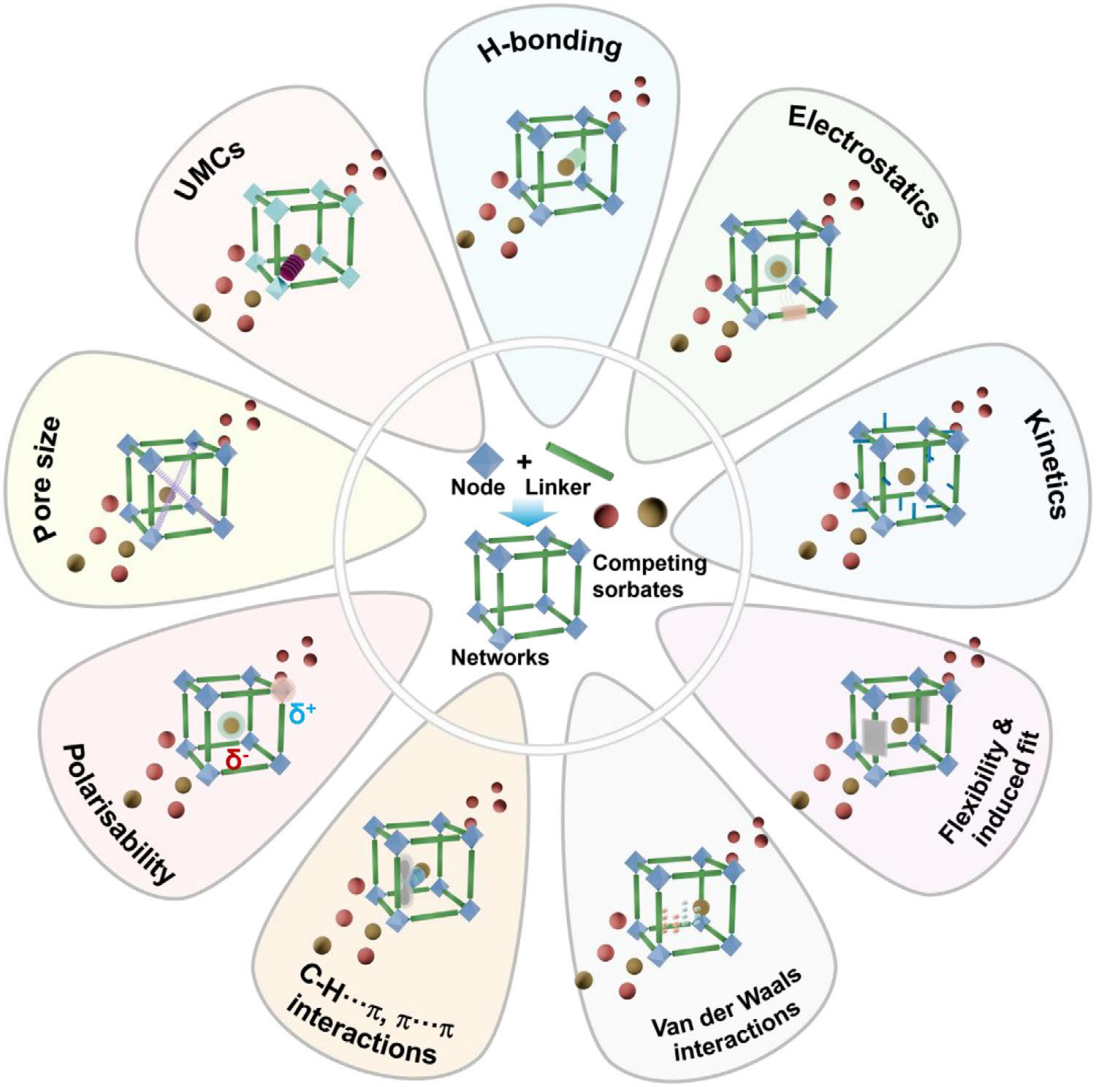
Nine mechanisms have been identified as factors that drive adsorptive HC separation by reticular materials: UMCs, H‐bonding, electrostatics, kinetics, flexibility, van der Waals interactions, C─H···π and π···π interactions, polarizability, and pore size.

That crystal engineering approaches can enable chemists to fine‐tune pore size, shape, and chemistry to achieve optimal sorbate binding has enabled a focus upon the mechanisms of sorbent‐sorbate binding to guide the next generation of reticular sorbents. As detailed herein, we and others have identified nine such mechanisms (and combinations thereof), including molecular sieving, UMCs, H‐bonding, electrostatic interactions, π···π and C─H···π interactions, van der Waals forces, framework flexibility, and kinetic sieving effects (Figure 26). Several of these are summarized below.

**Pore size really matters** as it can be the key to enabling molecular sieving, generally regarded as the most energy‐efficient approach for molecular separations [39, 130, 287]. Controlling pore size until it matches the kinetic diameter of a target molecule can enable molecular sieving, a striking example being ethylene/ethane separation by **UTSA‐280**. Two narrow channels (3.2 Å × 4.5 Å and 3.8 Å × 3.8 Å) lie between the cross‐sectional areas of C_2_H_4_ and C_2_H_6_, allowing C_2_H_4_ to enter while excluding C_2_H_6_ [287]. Consequently, **UTSA‐280** adsorbs 2.5 mmol g^−1^ of ethylene at 1 bar and 298 K but almost no ethane, translating to exceptional selectivity and >99% ethylene purity. **SIFSIX‐2‐Cu‐i** (pore ∼5.15 Å) and **SIFSIX‐3‐Zn** (∼3.84 Å) offer single‐site binding energies of ca. 45–60 kJmol^−1^ for C_2_H_2_ and CO_2_, respectively. These energies are in the sweet spot when they are strong enough to capture the target gases (C_2_H_2_ and CO_2_, respectively) when present in trace amounts, yet are not nearly as energy‐intensive as chemisorption [33, 35]. This balance can overcome the oft‐encountered *Catch‐22* of physisorption, where strong binding improves selectivity but hinders regeneration [827]. Indeed, tight binding thanks to ultramicropores enabled **SIFSIX‐3‐Zn** and its derivatives to become the first class of PCN physisorbents to exhibit selectivity consistent with that needed for DAC [33, 828]. Conversely, wider pore SIFSIX variants with the same pore chemistry exhibit much weaker binding for CO_2_ [36].

**Pore chemistry also really matters** as incorporation of open metal sites, hydrogen bonding moieties, or strong electrostatics can drive affinity for one HC over another. For instance, incorporation of Ag(I) cations into **NUS‐6(Hf)‐Ag** enables ethylene molecules to selectively bind to Ag^+^ via π‐electron donation and backbonding, affording an IAST selectivity of ∼106 for C_2_H_4_ over C_2_H_6_ [288]. Notably, this chemically modified CN maintained separation performance over repeated cycles. Likewise, UMCs in the **M‐MOF‐74** family (M = Mg, Mn, Fe, Co, Ni, Cu, Zn) were preferentially benzene over cyclohexane, marking the first use of Lewis acid–base interactions (π‐complexation) to separate aromatic/aliphatic HC isomers, viz., Bz over Cy [679]. In the same vein, electronegative SiF_6_^2^–sites favour strong binding with the more acidic C_2_H_2_ molecules (p*K*_a_ 25) vs. the less acidic C_2_H_4_ (p*K*_a_ 44) as demonstrated by **SIFSIX‐2‐Cu‐i** [35]. It is now evident that designing pore surfaces with the right functional group(s) can “programme” a reticular sorbent to favour one HC over another.

**Pore shape and topology can sometimes matter** as the spatial arrangement of pores (1D channels vs 3D cages, interpenetrated vs open frameworks) can influence diffusion rates and coadsorption, introducing kinetic selectivity in addition to thermodynamic selectivity. A good example is the separation of xylene isomers by two isostructural Zn(pba) CNs, **MAF‐88** and **MAF‐89** [776]. These CNs exhibit different pore architectures, narrow 1D channels in **MAF‐88** versus quasi‐discrete 0D cavities in **MAF‐89**. Consequently, **MAF‐89** achieves *p*‐xylene selectivities 10–20 times higher than **MAF‐88** for the same *p*/*o*/*m*‐xylene mixture. Molecular dynamics simulations support a diffusion‐controlled mechanism with the flexible narrow apertures in **MAF‐89** acting as gated traps that slow or exclude the larger isomers, whereas the more open channels of **MAF‐88** provide less discrimination. As discussed earlier (section 4.2, and Table 16), the molecular sorbent **TPBD** exhibits benchmark PX selectivity from C8 aromatic mixtures can be attributed to its structural adaptability and conformational polymorphism [778]. Despite being non‐porous, conformational polymorphs of **TPBD** (**TPBD‐αI**, **TPBD‐αII**) differ by ring orientations, each transforming to a PX–bound inclusion phase (**TPBD–PX**) upon PX exposure. Only *p*‐xylene was found to trigger the necessary induced fit pore opening while excluding *o*‐/*m*‐xylene and ethylbenzene. TPBD illustrates that pore shape can critically dictate separation outcomes. These are just two examples that illustrate how pore shape/topology can drive separation outcomes, including the harnessing of kinetic effects (diffusional “sieving”) [829].

**FIGURE 27:**
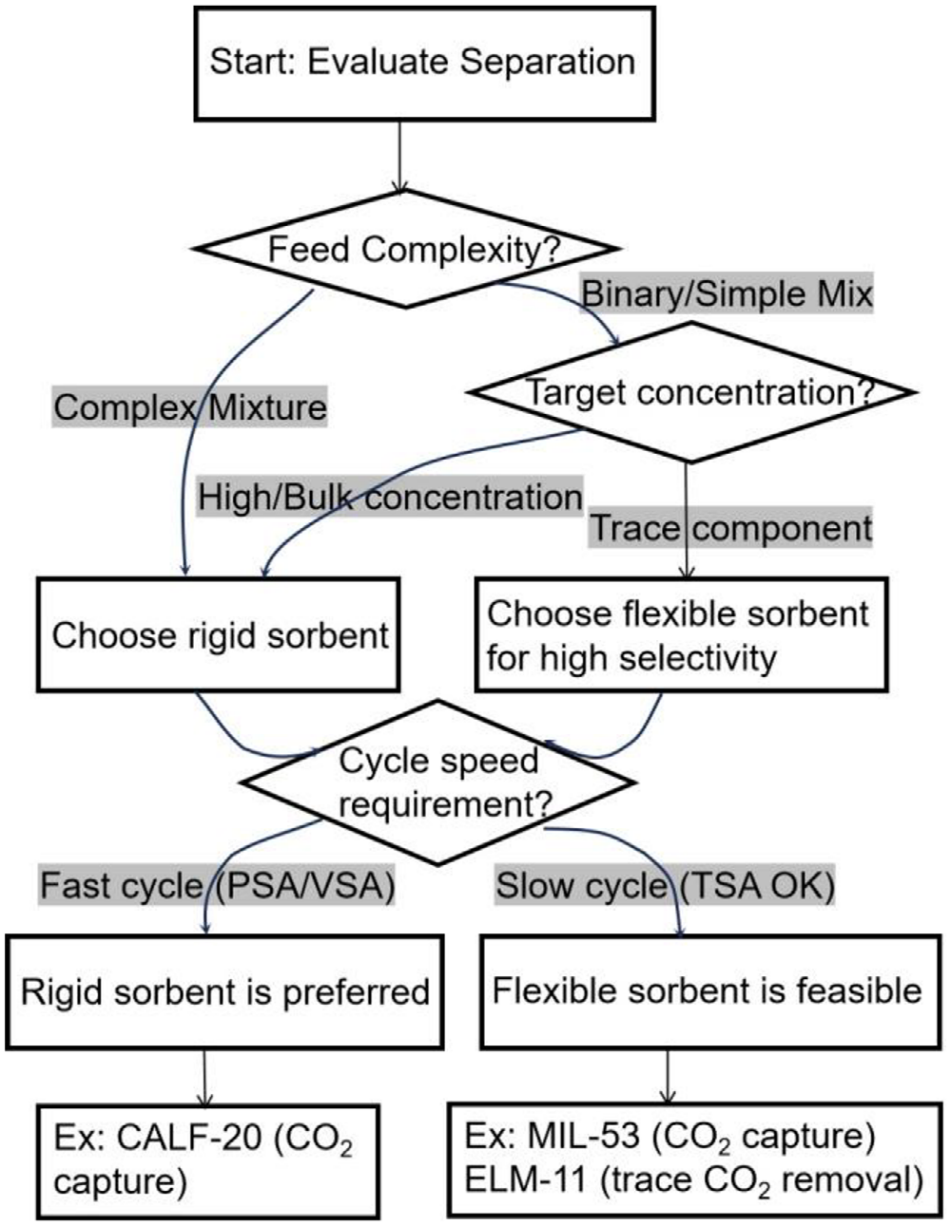
Decision framework: selecting rigid versus flexible hosts for physisorption‐based separations (Ex. = example(s)).

**Flexible frameworks** have recently emerged in the context of separations, with several studies revealing that they can exhibit induced‐fit guest binding [159]. Specifically, flexible CNs can undergo guest‐triggered structural transformations that selectively accommodate certain molecules by in effect adapting its structure to a preferred sorbate. For example, the flexible CN **JNU‐1** was found to “breathe” and reposition its UMCs to bind acetylene, achieving a C_2_H_2_/CO_2_ selectivity of ∼285 at 298 K [160]. Similarly, the HUM **TIFSIX‐14‐Cu‐i** remains essentially nonporous until C_3_H_4_ induces a structural change that enables its capture and production of C_3_H_6_ with 99.9999% purity [432]. The ability of a PCN to distort or expand only in the presence of the target sorbate confers a type of “exclusive” selectivity. Although the design rules for flexible sorbents are not yet mature, there are indications that they can offer the best of both worlds: high working capacity (since they can open up when fully loaded); high selectivity (since they remain closed to undesired sorbates) [830]. In this context, approaches like “sorbate‐selective induced fit” [237], and “double sieving” (multi‐step exclusion mechanisms) offer potential to target even the most challenging of trace separations. Nevertheless, the potential of flexible, stimuli‐responsive [831], transiently porous [832], and traditionally non‐porous sorbents, as well as derived composites, remains largely untested for HC separations. Given the distinct characteristics of rigid and flexible sorbents in adsorptive separations of light hydrocarbons, we present a decision framework to guide the choice between rigid and flexible sorbents for HC‐selective separations (Figure 27).

In summary, while a single mechanism may serve as the primary driver for the effective separation of HC mixtures, one must recognize that high selectivity and capacity are most often achieved through the synergistic combination of multiple noncovalent interactions. Rather than relying on a solitary interaction between sorbate and sorbent, the cooperative effects of van der Waals forces, hydrogen bonding, π‐π interactions, and host‐guest complementarity within porous frameworks can work in concert to enhance recognition and separation efficiency.

### 5.3 The Future in Focus: Remaining Challenges

**FIGURE 28:**
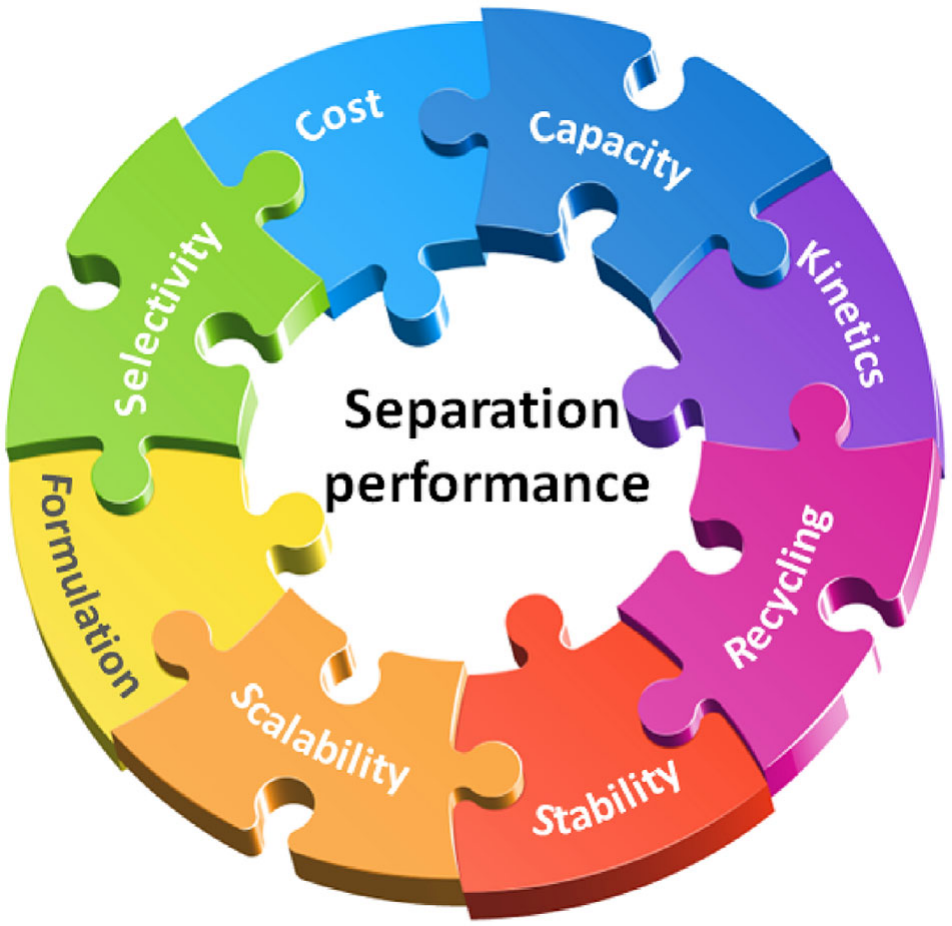
The spectrum of performance parameters that must be exhibited by a sorbent with regard to adsorptive separations and purifications, especially for commercial adoption.

Overall, future sorbents must satisfy a spectrum of performance parameters beyond selectivity alone (Figure 28). Key requirements include:

1. **High adsorption capacity** (i.e., working capacity) for the target HCs, to handle large throughputs.
2. **Fast kinetics** for both adsorption and desorption, enabling rapid cycling and high productivity.
3. **High selectivity** over competing species (ideally at relevant trace concentrations).
4. **Tolerance to humidity** and other impurities.
5. **Mild regeneration conditions**, i.e., low energy input for sorbent recycling.
6. **Processability** into industrial forms (uniform pellets, durable monoliths, etc.).
7. **Chemical, thermal, and mechanical stability** over several cycles.
8. **Scalability** of synthesis and deployment (feasible cost and volume).
9. **Multicomponent mixtures separation** including the use of tandem beds.

All of the above must be balanced when moving from laboratory demonstrations to commercial adoption. These performance parameters must be addressed from a chemist's lens in that composition can be fine‐tuned from the ground up since, as is the case for many top‐performing sorbents, this requires controlling pore chemistry and pore sizes with sub‐angstrom precision [815, 833]. In addition, it remains a challenge to predict many of the above requirements in advance of conducting an extensive range of experiments, which creates a bottleneck in that the number of PCNs being reported far outpaces our ability to test them comprehensively. This raises the issue of whether AI and predictive modelling are primed to reliably screen for functional properties and accelerate the commercial development of PCNs. It is the opinion of these authors that there is some way to go before this bottleneck is addressed. Some of the above criteria are discussed individually below.

Interference from humidity presents a major hurdle. In real‐world conditions, HC streams (especially gases from air or combustion processes) often contain water vapor, which tends to compete for adsorption sites and/or degrade some PCNs s. Further, some of the highest‐performing CNs are hydrophilic and thus prone to water adsorption. Mitigating this issue is essential unless the application itself is water capture. One solution is to design sorbents with inherent hydrophobicity and water stability – for example, by using hydrophobic linkers or high‐valence metal clusters that resist hydrolysis [834, 835]. Some robust PCNs, like **CALF‐20** and **BUT‐55**, retain their selectivity under humid conditions [74, 695], and some can even exploit pre‐adsorbed water to enhance hydrocarbon uptake via host–guest cooperation [836, 837, 838]. Still, adsorbents that are hydrophobic and offer the high selectivity values needed for trace separations remain rare for some separations and non‐existent for others [839].

Beyond binary separations, the highest volume gas mixtures (e.g., biogas, syngas, air, natural gas) are complex mixtures of varying compositions. That bespoke physisorbents have setting binary selectivity benchmarks for multiple impurities that are present in important gas mixtures (air, natural gas, C_2_ and C_3_ gases) suggests that a tandem packed fixed‐bed configuration comprising multiple sorbents, each tailored to target a specific impurity, might enable single‐step purification. Such an approach, SSST, was introduced in 2019 to produce polymer‐grade C_2_H_4_ from ternary (C_2_H_2_/C_2_H_6_/C_2_H_4_) or quaternary (CO_2_/C_2_H_2_/C_2_H_6_/C_2_H_4_) gas mixtures, using three ultramicroporous metal‐organic physisorbents, **SIFSIX‐3‐Ni** [69], **TIFSIX‐2‐Cu‐i** [36], and **Zn‐atz‐ipa** [840], in a tandem packed‐bed configuration in the right order [70]. In addition, there are now examples of single sorbents that can address multicomponent HC mixtures. **Zn‐atz‐oba** (oba = 4,4′‐oxobisbenzoate) was found to produce polymer‐grade purity (>99.95%) C_2_H_4_ from binary (1:1 for C_2_H_4_/C_2_H_6_), ternary (1:1:1 for C_2_H_2_/C_2_H_4_/C_2_H_6_) and quaternary (1:1:1:1 for C_2_H_2_/C_2_H_4_/C_2_H_6_/CO_2_) gas mixtures [419]. The Mn(II) CN, **NPU‐1** delivered C_2_H_4_ in >99.9% purity from a 1:1:1 C_2_H_2_/C_2_H_4_/C_2_H_6_ mixture in a single step [840]. Bringing this approach to a new level of complexity, **CALF‐20** was recently shown to achieve >99.99% C_2_H_4_ recovery from a seven‐component HC mixture, C_2_H_2_/C_2_H_4_/C_2_H_6_/CO_2_/C_3_H_4_/C_3_H_6_/C_3_H_8_ (0.6/62/10/0.3/0.6/26/0.5, *v*/*v*/*v*/*v*/*v*/*v*/*v*), even at 74% RH [841], whereas a tandem‐packed connection of two ultramicroporous PCNs, **MFM‐300(In)** (pore size ≈ 7.2–7.4 Å) and **MFM‐300(V)** (pore size ≈ 6.8–7 Å) enabled one‐step separation of *p*‐ and *m*‐xylene isomers from a ternary equimolar xylene mixture [815].

Another overarching challenge is scalability. To transition from lab discoveries to industrially adopted sorbents, materials must be economical to produce and compatible with standard process engineering. Unfortunately, many CNs and COFs rely on more expensive metals or complex organic linker ligands that can be difficult to synthesize on scale. In addition, shaping materials into usable forms (beads, pellets, or monoliths) without losing performance is non‐trivial. Several studies have explored shaping reticular sorbents into pellets or monoliths. In 2018, Xing's group pelletized several PCNs (**SIFSIX‐3‐Ni**, **SIFSIX‐2‐Cu‐i**, **GEFSIX‐2‐Cu‐i**, **TIFSIX‐2‐Cu‐i**) for C_2_H_2_/C_2_H_4_ separation, with **GEFSIX‐2‐Cu‐i** pellets showing an IAST selectivity of 116.4 for a 1:99 C_2_H_2_/C_2_H_4_ mixture at 298 K and 1 bar [842]. In 2021, Fairen‐Jimenez's group developed monolithic MOFs (**monoUiO‐66**, **monoUiO‐66‐NH_2_**, **monoHKUST‐1**) for CO_2_/N_2_ and CO_2_/CH_4_ separations, with **monoUiO‐66‐NH_2_** achieving a CO_2_/CH_4_ IAST selectivity of 54 for an equimolar mixture [843]. These studies underscore the main challenge in shaping MOFs for industrial use: balancing adsorption capacity with mechanical strength. Conventional binders often block pores, lowering performance. Recent strategies include ultrahigh‐loading pellets with minimal binder and binder‐free monoliths offering fast kinetics. While monoliths outperform pellets, their scalability is limited. Pelletization therefore remains the more practical route for applications such as carbon capture, provided the trade‐off between capacity and strength can be resolved by mitigating pore blockage.

Thus, an important future direction is the development of adsorbents from low‐cost, abundant, earth‐friendly precursors and simplifying their synthesis. A blueprint in this context is **CALF‐20** [74], which is comprised from low‐cost linker ligands, exhibits exceptional steam stability, and performance has been demonstrated over nearly half a million adsorption/desorption cycles. **CALF‐20**’s success in flue gas CO_2_ capture makes it a promising proof‐of‐concept for high TRL development of PCNs.

Finally, there are **design limitations** to confront. While the first generation of ultramicroporous coordination networks (Gen‐1 HUMs and related PCNs) has delivered several prototypes, they mostly belong to just a few families (e.g., Gen‐2 versions of **SIFSIX‐3‐Zn**, **Mg‐MOF‐74**). The development of new Gen‐1 materials with benchmark performance is still largely based on empirical discovery rather than design. A deeper understanding of structure–function relationships is needed to target new families of sorbents. This includes exploring understudied classes of potential sorbents that are already archived in the CSD. In addition, most research has thus far focused on rigid porous frameworks, yet flexible, stimuli‐responsive, and even nonporous or closed pore sorbents (such as molecular organic cage crystals with discrete voids, or composite materials lacking continuous porous channels but with potential to exhibit transient porosity) remain almost unstudied for HC separations. These unconventional sorbents could offer new mechanisms (e.g., gate‐opening, phase change, selective swelling). Additionally, hybrid approaches—like mixed‐matrix membranes or adsorbents integrated with catalytic sites—might address the constraints of pure adsorbents. In parallel, computational screening and machine learning may accelerate the identification of candidate sorbents that meet all of the desired criteria.

In conclusion, the strides made thus far mark what might be considered “*the end of the beginning*” for HC sorbents. Reticular chemistry and crystal engineering have brought us to the point where physisorbents can viably perform trace separations thought to be infeasible as recently as 15 years ago. The next decade will likely witness an even greater expansion of what is possible, as we leverage the insights gained so far to reach beyond this strong foundation toward more sustainable industrial separation technologies.

## Funding

Research Ireland, 16/IA/4624, IRCLA/2019/167, 21/PATH‐S/9454; European Research Council, ADG 885695); Synthesis and Solid State Pharmaceutical Centre, 12/RC/2275_P2.

## Conflicts of Interest

The authors declare no conflicts of interest.
